# The impact of comorbidities on surgical outcome and mortality in minimally invasive mitral valve surgery: a systematic review

**DOI:** 10.3389/fcvm.2025.1638217

**Published:** 2025-09-02

**Authors:** Vanessa I. T. Zwaans, Julia Stein, Simon Goecke, Leonard Pitts, Serdar Akansel, Markus Kofler, Stephan Jacobs, Volkmar Falk, Jörg Kempfert, Leonhard Wert

**Affiliations:** ^1^ Deutsches Herzzentrum der Charité, Department of Cardiothoracic and Vascular Surgery, Berlin, Germany; ^2^ Charité – Universitätsmedizin Berlin, Corporate Member of Freie Universität Berlin and Humboldt-Universität zu Berlin, Berlin, Germany; ^3^ DZHK (German Center for Cardiovascular Research), Partner Site Berlin, Berlin, Germany; ^4^ Department of Health Sciences and Technology, ETH Zürich, Zurich, Switzerland

**Keywords:** minimally invasive mitral valve repair (MIMVR), postoperative outcome cardiovascular surgery, minimally invasive surgery (includes port access, minimally invasive mitral valve surgery (MIMVS), minimally invasive mitral valve replacement

## Abstract

**Background:**

We sought to outline perioperative patient data to analyse surgical, clinical and echocardiographic outcomes and mortality of patients undergoing minimally invasive mitral valve surgery.

**Methods:**

Systematic literature research was performed in MEDLINE/PubMed according to PRISMA guidelines. Our research considered original works published until January 31, 2025. A pooled meta-analysis of studies reports early and late follow-up data of mitral valve repair for complex mitral valve regurgitation. In order to outline possible adverse events and comorbidities, we compared patients' mortality by differentiating preoperative, intraoperative and postoperative data.

**Results:**

This review analysed publications involving 222,947 patients, of which 43.4% were female and 56.6% were male, who underwent minimally invasive mitral valve surgery (MIMVS). The patients had a median age of 63.40 years (IQR: 60.42, 68.00), an average BMI of 25.1 kg/m^2^ (±7.9) and BSA of 1.7 m^2^ (±0.2). Severe mitral insufficiency was present in 86.4% of patients, 10% showed mild to moderate mitral insufficiency and 3.8% had mitral stenosis. The average EuroSCORE II showed a median value of 1.75% (IQR: 1.20, 2.95) and NYHA class III was most frequent. Comorbidities such as pulmonary hypertension were present in 35.37% of patients, diabetes mellitus in 8.57% (IQR: 4.76, 19.41), arterial hypertension was seen in 57.58% (IQR: 40.66, 68.79) with a significantly increased risk of mortality (*p* = 0.018). Coronary artery disease exhibited a prevalence of 17.41% (IQR: 10.78, 34.04), hypercholesterolaemia of 29.13% (IQR: 23.12, 49.74) and chronic kidney disease of 8.93% (IQR: 1.90, 20.00). New-onset atrial fibrillation occurred in 19.2% of patients. Besides this, 4% of patients required postoperative pacemaker implantation. Left atrial (LA) diameter decreased significantly from 50.37 mm preoperatively to 40.41%mm postoperatively (p < 0.001), LVDD was significantly reduced after MIMVS (*p* < 0.001). Mitral valve repair (75.83%) was considerably more common than replacement (21.09%). Applied techniques included annuloplasty (67.87%) and neochordal reconstruction (42.71%). Average mechanical ventilation was 540.8 min (±439.8), with a significant positive correlation between 30-day mortality and ventilation duration. In-hospital death occurred in 8 patients (±25), the average length of stay was 8.6 days (±3.9) and the mean postoperative ICU stay was 35.1 h (±15.9). Revision surgery was necessary in 4.1% of patients due to postprocedural bleeding. Postoperatively, 92% of patients showed no signs of MR, whereas 8% exhibited residual MR. Of those with residual MR, 78% continued to have mild MR, 14.9% had moderate MR and 7.1% showed severe mitral regurgitation after MIMVS.

**Conclusions:**

Postoperative mortality was associated with comorbidities like chronic kidney disease, diabetes mellitus and hypercholesterolaemia. Patients with a history of smoking, arterial hypertension or coronary artery disease showed variable risks, indicating that these factors may be associated with elevated in-hospital death or death within the first postoperative month. MIMVS shows favourable outcomes concerning echocardiographic measurements and haemodynamics such as LVEF, as well as length of hospital stay, ICU stay, postprocedural bleeding and complications such as wound infection or the need for blood transfusions.

## Introduction

Mitral valve (MV) disease occurs increasingly in patients with advanced age (1). The most critical parameters for morbidity and mortality risk include age, frailty, MV pathology and the possibility of MV repair. These modifying factors must be considered when choosing between conservative, transcatheter or surgical treatment. In order to make this decision, surgical and centre expertise is indispensable (1).

Depending on the location of the abnormality, mitral regurgitation (MR) can be divided into primary and secondary MR causes. Primary MR mainly focuses on the valve itself leading to MR due to infective endocarditis, rheumatic heart disease (RHD), connective tissue disorders, congenital malformations, drug use and mitral annular calcification. The latter often represents the degenerative pathogenesis which is most common in western countries. Mitral valve prolapse resulting from RHD is found more commonly in developing countries (2).

Secondary MR represents functional MR and is a result of LV remodelling such as mitral annular dilation and impaired LV contractility. Secondary MR can further be of ischaemic or non-ischaemic origin. Coronary artery disease leads to ischaemia of the myocardium and therefore LV dysfunction which in turn leads to ischaemic MR. Different types of cardiomyopathies (dilated, restrictive and hypertrophic) also leading to LV dysfunction constitute non-ischaemic MR. Besides this, annular dilation as a consequence of atrial fibrillation (AF) can also be the cause of non-ischaemic MR (3).

Concerning imaging modalities, echocardiography and further transoesophageal echocardiography are the key techniques to diagnose and evaluate MR but also to assess its aetiology, mechanisms, function, severity and prognosis. Furthermore, preoperative computed tomography (CT) screening and computed tomographic angiography (CTA) play a crucial role in ruling out coronary artery disease, visualising the thoracic cage as well as the thoracic and abdominal aorta and iliac arteries. In echocardiography, an effective regurgitant orifice area (EROA) ≥20 mm^2^ is associated with high mortality (4). In addition, 3D echocardiography helps to quantify regurgitant jets. The degree of MR is measured with cardiovascular magnetic resonance imaging (CMR) as a reference standard for quantifying left ventricular (LV) and left atrial (LA) volumes. Myocardial fibrosis assessed with CMR is frequent in primary MR and has been associated with sudden cardiac death and ventricular arrythmias. Currently recommended thresholds are LA diameter of ≥55 mm and left ventricular end-systolic diameter (LVESD) of ≥40 mm (5).

Guidelines of the European Society of Cardiology/European Society of Cardiothoracic Surgery (ECS/EACTS) recommend surgical treatment of chronic MR in symptomatic patients with severe primary MR. Postoperative outcomes are considered worse in the presence of ongoing LV remodelling (LVESD ≥45 mm or LVEF ≤ 60%), RV impairment, pulmonary hypertension (systolic pulmonary pressure ≥50 mmHg) and LA remodelling. Furthermore, atrial fibrillation is considered a trigger factor for early remodelling processes and is associated with worse postoperative outcomes.

There are different points of view concerning the timing of intervention. Urgent surgery in primary MR is only warranted in patients with acute severe MR due to acute papillary muscle or chordal rupture or infective endocarditis (5).

The gold standard for severe MR remains native MV repair. MV repair shows better short- and long-term outcome in direct comparison to MV replacement (6, 7). Nevertheless, it must be taken into account that surgical techniques for reconstruction remain debatable. MV repair using an undersized annuloplasty ring to regenerate leaflet coaptation is the preferred technique. In patients with risk factors for residual or recurrent MR in echocardiography, preference should be given to valve replacement over repair. MV replacement for ischaemic MR provides a more durable correction but does not show a better clinical outcome (1).

Minimally invasive mitral valve surgery (MIMVS) has been proven an appropriate alternative to conventional median sternotomy. Not only can a minimally invasive approach reduce surgical trauma, it can also promote postoperative recovery which has a beneficial impact on patients' quality of life. Due to prolonged life expectancy and in the presence of accompanying comorbidities like concomitant heart failure, minimally invasive cardiac surgery (MICS) represents a reproducible, less harmful alternative to reduce trauma and mortality (8, 9). Overall, reducing the degree of surgical trauma, minimising costs and reinforcing surgical skills continue to be the rationale of minimally invasive procedures.

The aim of this systematic review was to identify comorbidities that have an impact on MIMVS by investigating the early (failed repair, in-hospital mortality, and relevant complications) and late complications (recurrent regurgitation or redo) of minimally invasive surgery in the context of mitral valve repair or replacement.

## Methods

The literature research, concept, inclusion criteria, research question and hypothesis of this review were defined according to the Preferred Reporting Items for Systematic Reviews and Meta-Analyses (PRISMA) guidelines (10).

### Search strategies

**Figure 1:**
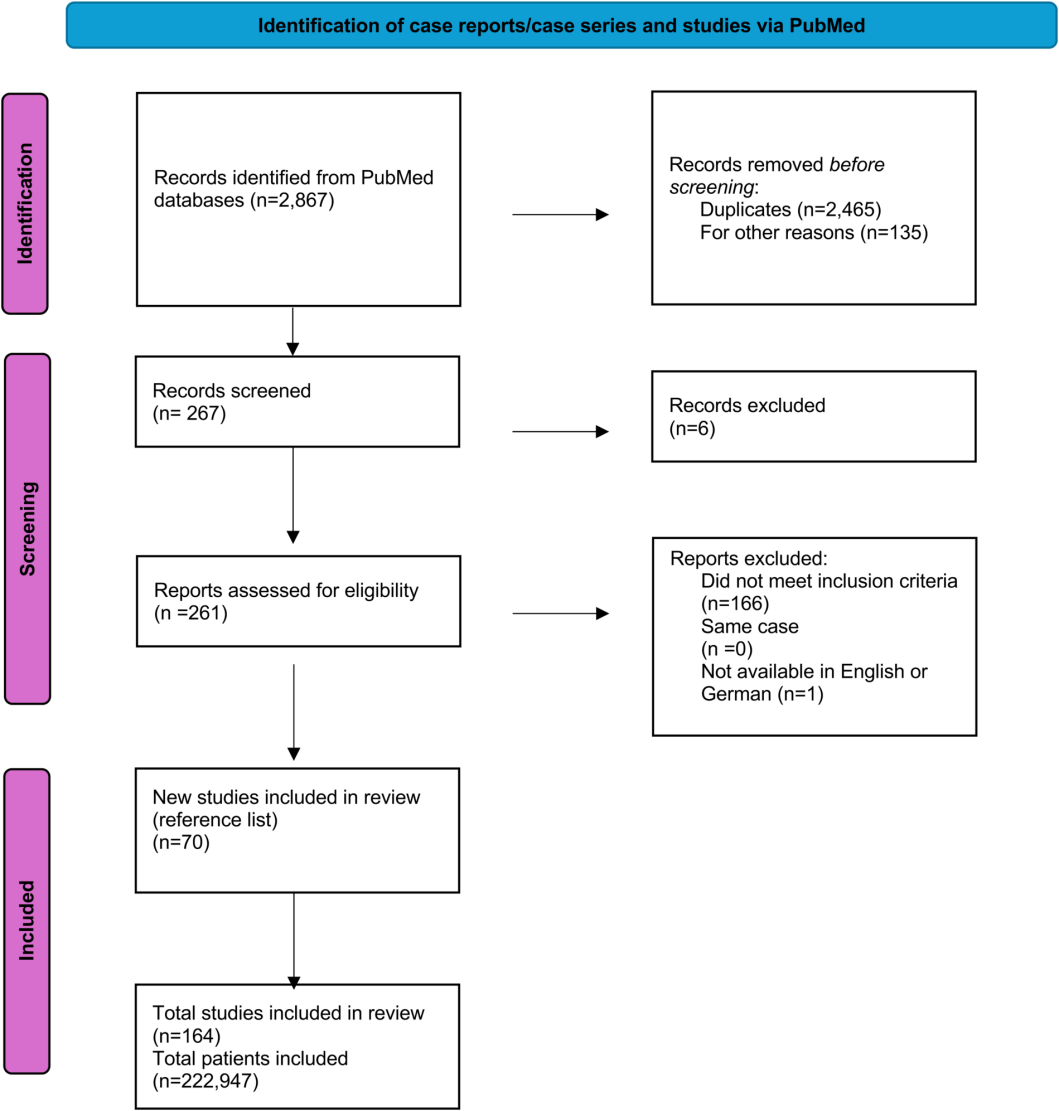
PRISMA (preferred reporting items for systematic review and meta-analysis) flowchart of the systematic literature review.

Applying the following mesh terms, systematic research on PubMed was carried out until January 31, 2025 (“Minimal invasive mitral” or “Minimally invasive cardiac surgery”) AND (“outcome”, “mitral valve”, “echocardiography”, “mitral valve repair”, “mitral valve replacement”). The results of this research were screened and included, provided they met the inclusion criteria of minimally invasive mitral valve surgery and postoperative outcome. Matching publications were then imported into the reference management software EndNote®. The first author reviewed all full texts of the included publications (Figure 1).

### Inclusion criteria

Published case reports and case series as well as retrospective, observational or randomised clinical trials (RCT) among patients diagnosed with mitral regurgitation and/or stenosis as a main indication for operative reconstruction were considered. This review included publications from the last six years (2018–2024). This also included additional interventions for mixed valve diseases (aortic, tricuspid valve insufficiency and/or stenosis, aortic dissection).

**Figure 2:**
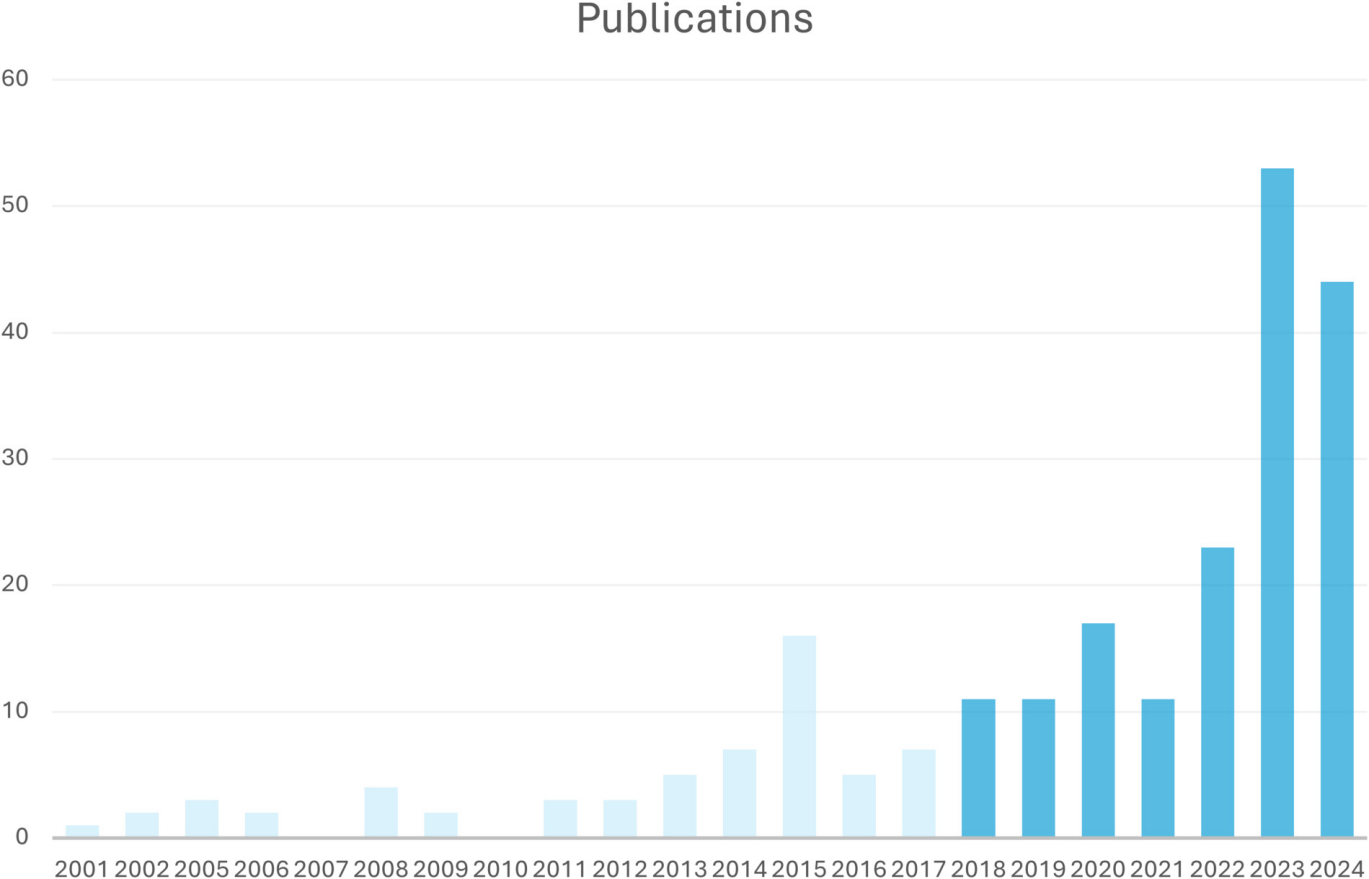
Number of included publications per year; included were publications from 2018 to 2024.

Literature not written in English or German, poorly described case reports, review articles and studies not providing enough information were excluded. Studies were excluded from the analysis if data were in a non-extractable format, duplicated or if the research was conducted in an animal model. Two assessors independently reviewed the titles and abstracts of potentially eligible studies and selected studies that met the inclusion and exclusion criteria for full-text retrieval and further examination. While the publications span a period of 23 years, the publications included in this review were from the last six years (Figure 2).

### Data extraction

The extracted data was collected by the first author and reviewed for accuracy by the last author. Data extracted included authors, title, year of publication and patient demographics (age, sex). Further data was subdivided into three groups: preoperative, perioperative and postoperative patient data, which were extracted and recorded in Microsoft Excel 2024 (Microsoft Corp., Redmond, WA, USA).

Preoperative data included NYHA class, BMI, BSA, atrial fibrillation (AF), pulmonary arterial pressure, ejection fraction (EF), degree of mitral insufficiency (MI) as well as tricuspid and aortic regurgitation. Echocardiographic data included data such as left ventricular ejection fraction (LVEF), LA dimension, mean pressure gradient (MPG), mean transvalvular velocity (MTV), mitral orifice area (MOA), LVDD and LVEDV. Since no differentiation was made for very low LVEF, all patients with very low LVEF were considered to have an LVEF of 35%. Comorbidities included chronic kidney disease (CKD), arterial hypertension, chronic obstructive pulmonary disease, diabetes mellitus (DM), coronary artery disease (CAD), history of stroke, pulmonary hypertension, hyperlipidaemia, history of smoking and a positive family history. Valvular comorbidities included, e.g., Barlow's disease, congenital mitral cleft, infective endocarditis, rheumatic valve disease, ischaemic valves, dilated cardiomyopathy and valve degeneration.

Perioperative data included aortic cross-clamp (AoX) time and duration of cardiopulmonary bypass (CPB).

Postoperative data relating to mortality, rehospitalisation, neurologic event (stroke or transient ischaemic attack), myocardial infarction, vascular complication (dissection, rupture, residual insufficiency), bleeding complication (specifically reintervention for bleeding), acute kidney injury, arrythmia, ventilator time, ICU length of stay (LoS) and hospital LoS were extracted.

### Outcomes

The aim of this systematic review was to identify comorbidities and anatomic constitutions that might influence pre- and perioperative outcome as well as postoperative 30-day mortality. These further include postoperative complications, prolonged ICU stay and the degree of residual mitral regurgitation post-reconstruction. Further aspects of analysis were cardiopulmonary bypass (CPB) and cross-clamp (CC) times, failed repair necessitating valve replacement, associated tricuspid procedures, reopening for bleeding, in-hospital mortality and total length of stay. The need for conversion to sternotomy was also recorded in the minimally invasive group as a safety endpoint.

## Results

### Literature research

Of 2,867 articles retrieved for evaluation, 2,732 including double publications met the inclusion criteria based on the abstract; of these, 261 were identified as relevant and full-text reading was performed. Ultimately, 167 articles were excluded because they did not meet the inclusion criteria or were not written in English or German or were case reports. In addition to the 94 articles extracted from PubMed, 70 articles as secondary literature items were included. See list of publications (Supplementary Table S1).

Study populations varied in quantity and not all publications provided preoperative, perioperative and postoperative data. Therefore, not every patient group was screened for all factors associated with possible adverse events in MIMVS. The main focus of the studies was also different, e.g., the cosmetic result assessed by patients' quality of life after the intervention, the duration of postoperative hospital stay, or robotic vs. non-robotic minimally invasive surgery (11–13).

After full-text evaluation, 164 articles providing individual data of 222,947 cases were included. While the publications span a period of 23 years, the publications included in this review were from the last six years (Figure 2).

The included papers were randomized trials, observational or case-control studies focusing mainly on minimally invasive mitral valve surgery and postoperative outcome. All cohorts consisted of patients with mitral valve diseases either of primary or secondary aetiology. Concerning mitral valve disease pathogenesis, eight publications focused on Barlow's disease (4, 9, 14–19). Nineteen publications focused on comparing minimally invasive valve surgery and conventional sternotomy (8, 14, 15, 20–37).

A dataset (Supplementary Table S1.1–S1.3) of 222,947 patients of mixed origin was ultimately obtained; of these, 96,682 were female and 126,265 were male.

Since each of the reviewed studies had individual endpoints, the data collection process differed, and not all details were provided by all publications (Supplementary Table S1.0–S1.3). Therefor data showing comparisons to 30 day-mortality was collected from 32,447 patients (see missings Supplementary Table S1.0–S1.3).

### Baseline patient characteristics

The median age was 63.40 years (IQR: 60.42, 68.00). The BMI for provided patient data was 25.1 kg/m^2^ (±7.9) and, where available, body surface area (BSA) was 1.7 m^2^ (±0.2).

There is no correlation between BMI and 30-day mortality *p* = 0.3914. Therefore, BMI is not be a strong predictor of 30-day mortality in this dataset. Nevertheless, a higher BMI between 24 and 28 kg/m² bears a higher risk for postoperative mortality with 3%–10%.

**Figure 3:**
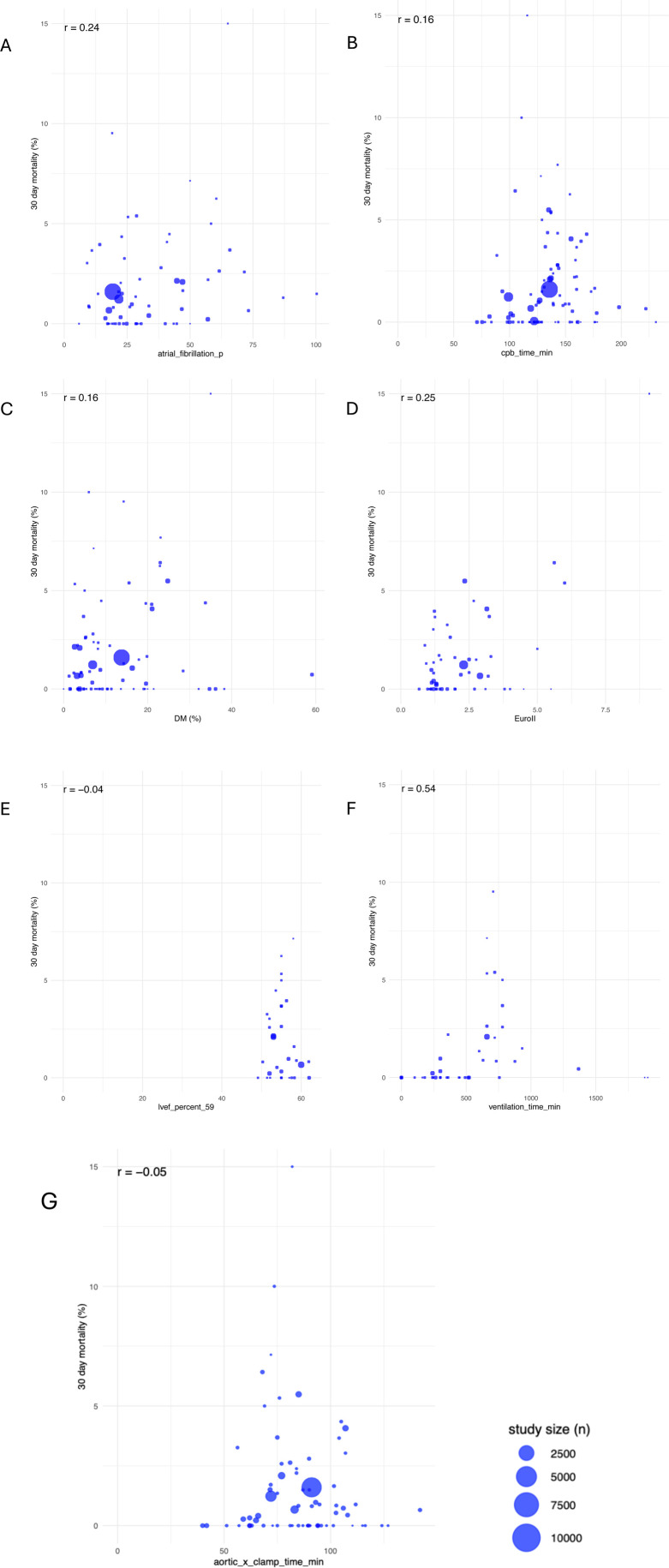
Correlations between 30-day-mortality and atrial fibrillation **(A)**, CPB **(B)**, diabetes mellitus **(C)**, euroScore II **(D)**, LVEF **(E)**, ventilation time **(F)** and aortic cross-clamp tme **(G)**; study sizes varied as seen in given legend.

The average EuroSCORE II showed a median value of 1.75% (IQR: 1.20, 2.95). As seen in Figure 3D, there is no significant positive correlation between EuroSCORE and 30-day mortality (*r* = 0.25). Nevertheless, it has to be taken into account that this score has been developed for patients undergoing cardiac surgery under cardiopulmonary bypass via sternotomy. Therefore it is not 100 percent transferable to predict hospital mortality for patients undergoing MIMVS (Figure 3B).

Considering the baseline data with a median age of 63.40 years (IQR: 60.42, 68.00) with a *p*-value of 0.0732, there is no correlation here, indicating that as age increases, so does the 30-day mortality rate.

Patients' NYHA classification was either collected as the mean overall NYHA of one patient cohort, or the number of patients of each NYHA group was listed. NYHA classes were distributed as follows: 10.5% NYHA class I, 30.4% NYHA class II, 34.9% NYHA class III, and 6.7% NYHA class IV.

Two other parameters were considered as baseline information, namely a preoperative history of atrial fibrillation and mean pulmonary arterial pressure (mPAP). The latter was measured inconsistently with a mean of 40.05 mmHg (±13.61). In this regard, an average of 35.37% of patients were diagnosed with pulmonary hypertension.

The mean incidence of preoperative atrial fibrillation was 27.61% whereas the mean incidence of postoperative new-onset AF was 19.2%. Furthermore, 4% of patients postoperatively underwent pacemaker implantation. As regards 30-day mortality, no clear pattern or trend can be identified in Figure 3A, suggesting that atrial fibrillation (AF), albeit a known risk factor, does not consistently predict 30-day mortality in a linear manner (*r* = 0.24) (Figure 3A). Compared to MIMVS, the incidence of new-onset atrial fibrillation in MVS via sternotomy is around 30%–40% and 5.3% of patients show an indication for pacemaker implantation within 30 days after surgery (38–40).

With a mean of 388.28 (±2,510.5) female patients per study, there were fewer female patients undergoing MIMVS than male patients.

**Figure 4:**
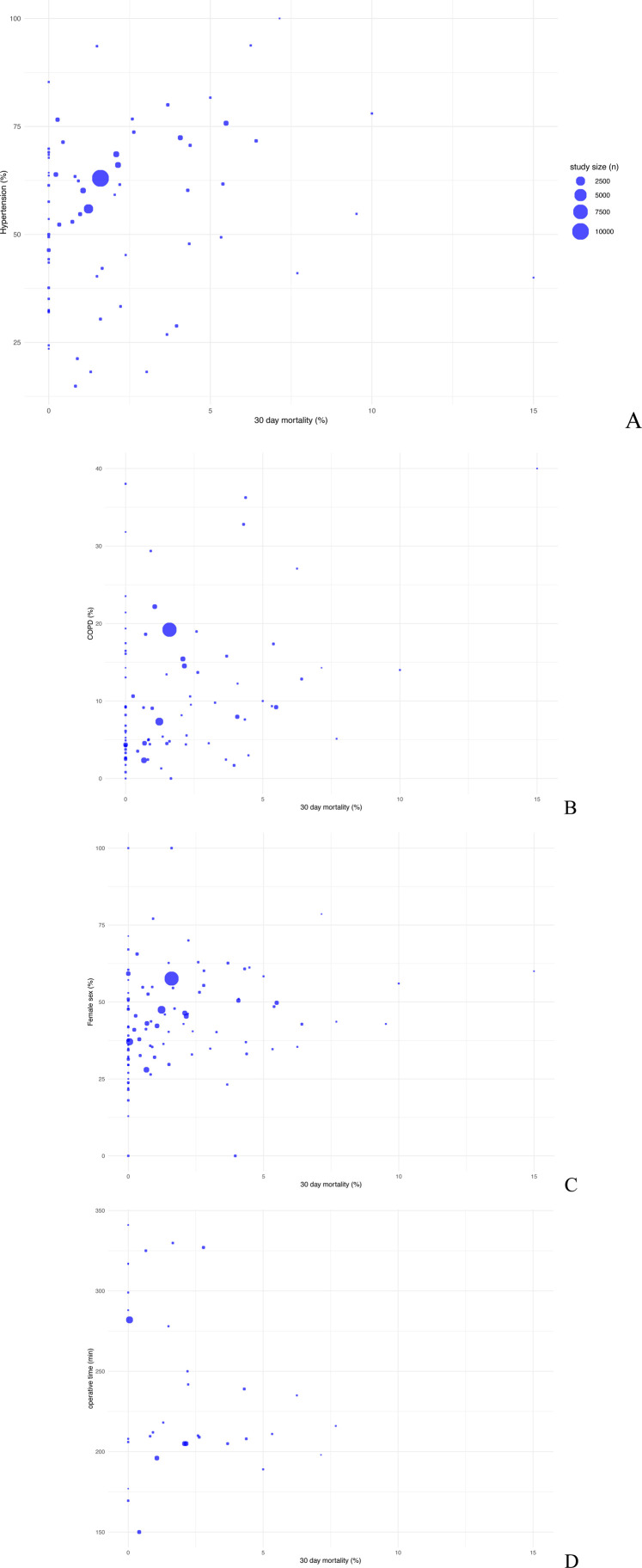
Scatter plot comparing postoperative 30-day mortality with baseline data arterial hypertension **(A)**, COPD **(B)**, female sex **(C)** and operative time **(D)**.

Considering sex distribution there is no indication for a relationship between the proportion of females and 30-day mortality. The datapoints are broadly scattered across different mortality percentages disregarding female percentage (Figure 4C).

With 86.4%, severe MI is the most common degree of MI in MIMVS, followed by 10% mild to moderate MI and, finally, 3.8% mitral stenosis. The number of patients suffering from severe mitral insufficiency preoperatively, with a mean of 86.4%, does not correlate with higher postoperative mortality risk.

Mitral valve insufficiency (MI) often coexists with tricuspid and aortic valve insufficiency.

Based on the data, 57.2% did not exhibit any tricuspid valve pathology, while 20.5% had mild tricuspid regurgitation (TR), 17.0% had moderate TR and 5.3% showed severe TR.

Overall, there was less information on aortic valve degeneration: 81.4% did not exhibit aortic valve insufficiency, while 14.5% showed mild aortic regurgitation (AR), 4.1% moderate AR and none developed severe AR resulting from MI.

Comorbidities such as chronic kidney disease (CKD) or kidney impairment, with a median of 8.93% (IQR: 1.90,20.00), correlated with a higher mortality risk. 30% of patients with CKD showed a mortality risk of up to 6%. However, the degree of association between these two factors varied.

Concerning the history of arterial hypertension, with a median prevalence of 57.58% (IQR: 40.66, 68.79), there is a no significant connection to an increased risk of mortality, which can be seen in large study sizes with more than 50% of patients suffering from high blood pressure (Figure 4A).

Other risk factors such as diabetes mellitus (DM), with a median prevalence of 8.57% (IQR: 4.76, 19.41), show a trend towards high mortality without significant correlation (*r* = 0.16). Small percentages of patients affected by DM seen in large study sizes exhibit risk factors up to 2.5%.

A similar trend can be seen with the risk factor COPD, with a median occurrence of 9.06% (IQR: 4.42, 14.52).

Low percentages of patients with coronary artery disease, with a median prevalence of 17.41% (IQR: 10.78, 34.04), have a high mortality risk with up to 6%. However, the given publications failed to define the degree of coronary artery disease and it is not known whether those patients affected by CAD had to undergo surgical or non-surgical intervention via stent implantation.

The median prevalence of patients with high lipid levels is 29.13% (IQR: 23.12, 49.74), without significant correlation to high mortality risk (Figures 3C, 4B).

Patients with a history of stroke or TIA show a median of 6.26% (IQR: 3.51, 8.01), whereas smoking history was present in 17.53% (IQR: 8.6, 30.56) of all collected cohort data.

The data tables show a prevalence for dilated cardiomyopathy with a median of 3.79% (IQR: 3.55, 4.04).

As regards the aetiology of MV disease, 13.3% of patients showed Barlow's disease, 4.8% of MIs were caused by congenital mitral cleft, 9.8% resulted from a history of infective endocarditis, 8.5% showed rheumatic valve disease and 42.3% of mitral insufficiencies resulted from degenerative valve destruction.

Laboratory examinations were carried out pre- as well as postoperatively but were not regularly recorded. Most reported laboratory values were haemoglobin, creatinine as well as creatine kinase (CK) and muscle-brain type CK (CK-MB).

**Figure 5:**
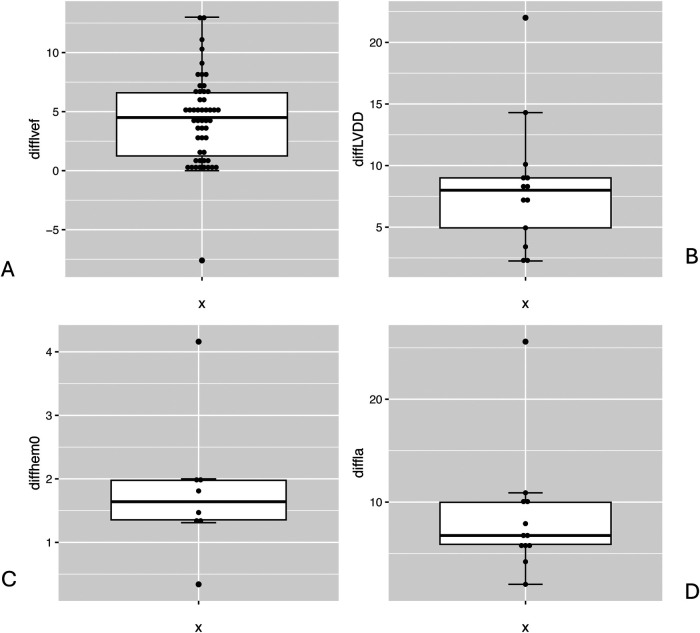
Boxplot comparison preoperative and postoperative values: LVEF (%) **(A)**, LVDD (mm) **(B)**, haemoglobin (g/dl) **(C)**, LA diameter (mm) **(D)**.

The average preoperative creatinine value was 0.97 mg/dl (±0.36), compared to 1.12 mg/dl (±0.53) postoperatively. Furthermore, average haemoglobin was 13.11 g/dl (±0.95) preoperatively vs. 10.79 g/dl (±0.88) postoperatively. There was a significant difference between the pre- and postoperative values (Figure 5C).

Preoperative CK was on average 386.25 µmol/L (±553), CK-MB was 29.55 U/L (±16.25).

### Echocardiography

Left ventricular systolic function constitutes patients' core risk profile and was collected as a patient-specific value with a mean of 55.81% (±9.93) and a median of 55.25 (IQR: 22.27, 98.90). LVEF lower than 35% is considered to have a high 30-day mortality risk of 2.5%–10%. However, a higher LVEF of 56% and above, seen especially in large study sizes, does not cross the 5% risk threshold. It must be noted that 22 publications did not differentiate patients' low left ventricular output capacity and grouped these collectively as having an LVEF under 35% (8, 9, 18). Since no differentiation was made for very low LVEF, all these patients were considered to have an LVEF of 35%. Postoperative LVEF remained overall level with a mean of 55.99% (±3.26). There is no significant correlation to 30-day mortality risk (*r* = −0.04) (Figures 3E, 5A).

Another dimension assessed by transoesophageal echocardiography (TOE) was left atrial (LA) diameter with a preoperative mean of 50.37 mm (±9.01) and a postoperative mean of 40.41 mm (±5.29). Minimally invasive mitral valve surgery also led to a significant improvement in LA dimension (Figure 5C).

Three main dimensions were assessed by TOE both pre- and postoperatively: mean mitral valve pressure gradient (MPG), mean transvalvular velocity (MTV) and mitral orifice area (MOA). The mean preoperative MPG was 2.73 mmHg (±1.29) compared to 3.13 mmHg (±0.7) postoperatively. The mean preoperative MTV, assessed only once, was measured as 1 m/s, whereas postoperative transvalvular velocity was 1.22 m/s. The mean MOA, a relevant dimension for evaluation, was 0.5 cm^2^ preoperatively and 1.22 cm^2^ (±0.62) postoperatively.

Another relevant dimension is (LVDD), which on average measured 55.25 mm (±4.79) preoperatively and 48.13 mm (±6.41) postoperatively. The difference between these values showed a significant reduction in LVDD after MIMVS (Figure 5B). Concerning haemodynamic parameters, the mean postoperative LA dimension after MIMVS was measured as 40.4 mm (±5.3) compared to 50.4 mm preoperatively (±9.0) (Figure 5C).

### Perioperative data

Numerous perioperative data was collected covering different areas such as general surgery times, use of different cardioplegia methods, valve ring sizes, and other procedures that needed to be performed additionally (Supplementary Table S1.2).

Considering perioperative data such as total operative time, CPB with a median of 135.6 min (IQR: 118.95, 154.5) and aortic cross-clamp time with a median of 84.9 min (IQR: 72.00, 96.85), there is no correlation with 30-day mortality (*r* = −0.05). Overall, a CPB time between 100 and 150 min as well as cross-clamp times of up to 90 min have a higher risk of death, with both being up to 6.0%, while durations of more than 90 min are not thought to increase the risk of mortality (*r* = 0.16) (Figures 3B,G).

An average of 11.5% underwent concomitant tricuspid valve surgery, vs. an average of 22.4% patients who underwent concomitant aortic valve replacement.

**Figure 6:**
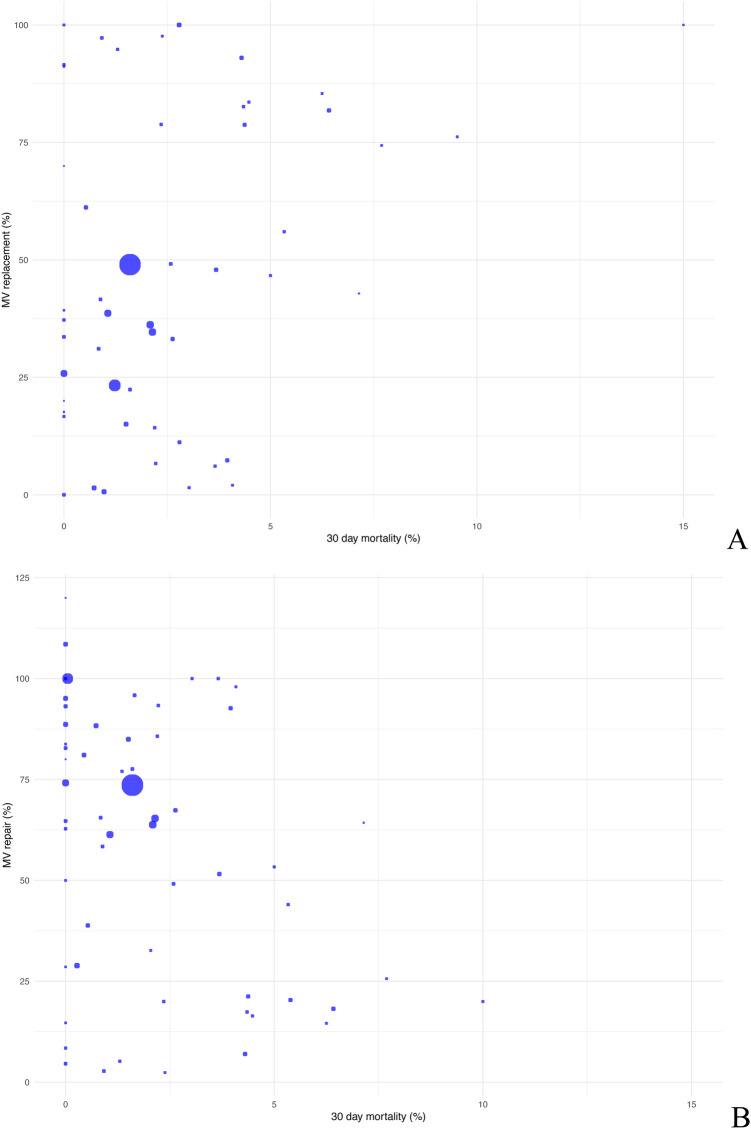
Scatter plot comparing postoperative 30-day mortality with mitral valve replacement **(A)** and mitral valve repair **(B)**.

Based on the data, 21.09% of patients underwent mitral valve replacement and 75.83% mitral valve repair. Mitral valve replacement among patient cohorts showed prevalences of up to 50% in some studies whereas MV repair was the more commonly applied method. As regards 30-day mortality, no explicit trend was observed for either of the methods. Therefore, other concomitant factors have to be taken into account. However, data for both procedures range within low mortality risks (Figures 6A,B).

Considering valve reconstruction, 42.7% of patients received neochordae as reconstructive material and 67.9% underwent annuloplasty. While 61.2% suffered from MI due to prolapse either of the anterior mitral leaflet (AML), the posterior mitral leaflet (PML) or both, and 46.6% showed rupture of a papillary muscle due to myocardial ischaemia. Alfieri plasty, also known as edge-to-edge repair, was conducted in 6.9% of patients suffering from MI. The mean mitral valve ring size was 33.03 mm (±2.26).

The average duration of surgery was 235.6 min (±59.7), CPB time was 137.6 min (±33.7) and aortic cross-clamp time was 90.2 min (±21.1).

Different types of cannulation techniques were employed during CPB but were inconsistently reported. Arterial cannulation via the ascending aorta was most common, via the femoral artery in 12.7% of cases, while 0.4% received CPB via the right axillary artery.

In this context the application of cardioplegia was recorded incoherently. Bretschneider solution was administered to 4,546 patients whereas 1,028 patients received St. Thomas cardioplegia (41, 42). Furthermore, two studies outlined the mode of application with either antegrade perfusion which was seen in an average of 2,158 patients (±3,020) or 62% besides antegrade as well as retrograde perfusion in 618 patients (±968) which made up to 24% (43, 44).

Left atrial (LA) ablation and left atrial appendage (LAA) occlusion are two commonly employed procedures for the preventive management of postoperative atrial fibrillation (AF) and its complications, as well as for the treatment of preoperative persistent or permanent AF (45). LA ablation was conducted in 11.8% of patients, whereas LAA occlusion accounted for 18.3% of perioperative procedures.

Other perioperative data was collected, such as mean periprocedural blood loss with 356.3 ml (±150.3). Additionally, 2% of MIMVS had to be converted to sternotomy.

### Postoperative data

Postoperative data revealed the most common complications after surgery as well as over the general duration of stay (Supplementary Table S1.3). Ventilation duration shows no significant but a trend towards positive correlation with increasing mortality risk (*r* = 0.54). Up to 500 min, the mortality risk stays below 2%. Crossing the threshold of 500 min, there is a correlation with up to 9% risk for postoperative death. The dataset in this review shows a mean ventilation duration of 540.8 min (±439.8) (Figure 3F).

Postoperative low cardiac output was seen in 3.7% of patients undergoing MIMVS.

Due to postprocedural bleeding, revision surgery was necessary in 4.1% of patients who underwent either MV repair or replacement. A mean of 8 patients or 1.3% (±25) died in hospital shortly after the operation. The average length of stay in hospital was 8.6 days, and the mean postoperative ICU stay was 35.1 h; of these patients, 7.8% had to be readmitted to the ICU due to complications. According to other meta-analyses, length of stay does not differ significantly, with 7.6 days for MIMVS compared with 9.4 days for MVS via conventional sternotomy (CS). Similar results are observed for ICU stay, with a length of stay of 44 h for MIMVS vs. 66 h for CS (46).

Postoperative complications such as pneumonia occurred in 1.7%, transient neurocognitive dysfunction in 4.4% of patients, of which 1.1% suffered a cerebral stroke. Postoperative myocardial infarction was seen in 0.9% of patients and wound infection in 1.5% of patients. Renal replacement therapy was necessary in 3.8%, with 1.6% requiring haemodialysis.

The majority of 92% of patients had no sign of residual MR whereas 8% did. The prevalence of residual MR was distributed as follows: 78% continued to have mild MR, 14.9% had moderate MR and 7.1% showed severe mitral regurgitation after MIMVS.

## Discussion

### Advantages and disadvantages of MIMVS

Minimally invasive techniques show several advantages such as smaller incisions, less trauma to the chest wall, reduced pain and a reduction of surgical site infections postoperatively (47, 48). As outlined in this review, postoperative complications remain apparent but overall low, for example, 1.5% of patients showed postoperative wound infection. These factors, as well as cosmetic benefits of MIMVS can have a positive effect on patients' quality of life, improving the recovery process (20).

Furthermore, with an average of 356.3 ml (±150.3) there is less blood loss compared to open-heart surgery, which decreases the need for blood transfusions during and after surgery (8, 49).

MICS techniques involve less manipulation of surrounding tissues and organs, reducing the risk of damage to structures and thereby minimising potential postoperative complications.

However, not all patients are suitable candidates for MIMVS. Factors such as the complexity of the mitral valve disease, the valve and the patient's rib cage anatomy and concomitant risk factors must be carefully evaluated to determine the appropriate approach. Overall, it is known that MICS results in a reduced risk of infection, less blood loss and pain, and a reduced hospital stay [with an average of 8.6 days (±3.9)], which also enhances postoperative rehabilitation (50).

However, disadvantages must be considered. Bypass and clamp durations tend to be 10% to 30% longer due to the more time-consuming and complex operative setup involving femoral cannulation and thoracoscopic assistance (39). Equipment and setup costs can be higher than in the CS approach, which uses endoscopic cameras or robotic systems. However, this is offset by a shorter length of hospital stay and reduced complications (38). MIMVS is not ideal for many patients, especially those with comorbidities such as severe peripheral vascular disease (22). The limited field of view requires surgical experience and bears a higher risk of complications since navigating intracardiac structures is more challenging (40). In addition, peripheral cannulation can lead to retrograde aortic dissection, groin infection and femoral vessel trauma (51).

### Is robotic assistance an added value in minimally invasive mitral valve surgery?

The surgical outcome can be sustainably improved by using robotic assistance. High-definition, three-dimensional visualisation and magnification of the surgical field enhance surgical precision. In comparison to MIMVS, the assistance of robotics allow the surgeon to make use of another degree of angle. This can facilitate a more accurate assessment and repair of the mitral valve, leading to improved surgical outcomes (52, 53).

However, it is essential that training with robotic systems remains part of a surgeon's residency in order to improve proficiency in robotic-assisted techniques and accelerate the surgeon's learning curve (54, 55). Depending on the surgeon's experience surgical outcomes can even be improved (52).

Another benefit of robotic surgery over conventional surgery are small incisions with less than five centimetres leading to minimised tissue manipulation and reduced trauma to surrounding structures, a reduction in postoperative pain and faster recovery times for patients, similar to MICS.

### What are the factors associated with failed fast track in minimally invasive mitral valve surgery?

Fast-track recovery includes early extubation, reduced ICU stay, postoperative management, and early mobilisation. The reasons why patients may not meet the criteria for fast track vary. Noteworthy haemodynamic instability that leads to ongoing need for vasopressor—support as well as respiratory instability are known factors to influence the decision for ICU admission instead of on-table extubation (13).

Comorbidities can increase the risk of both perioperative and postoperative complications, potentially delaying recovery. Additionally seen in this review, the severity and complexity of the patient's mitral valve disease, including the presence of related cardiac conditions such as atrial fibrillation with 27.61%, coronary artery disease with a median prevalence of 17.41% (IQR: 10.78, 34.04), or heart failure with 6.7% of patients registered with NYHA class IV, can also impact postoperative recovery.

Inadequate pain management, poor fluid management, or excessive intraoperative analgesia can further impair postoperative respiratory function, hindering fast-track recovery. A time-directed extubation protocol and low-dose opioid-based general anaesthesia can help alleviate these issues (42, 56, 57).

Furthermore, haemodynamic instability, including low cardiac output syndrome, seen in 3.7% of patients, arrythmias or fluid overload may result in a prolonged ICU stay (50).

Sünderman et al. showed that in direct comparison of MIMVS vs. open mitral valve surgery, the length of ICU stay was 20 h shorter in MIMVS (44 vs. 66 h), mainly due to a reduced mean length of ventilator dependence. This aligns with findings in this review outlining an average stay of 35.1 h (±15.9).

### Impact of cardioplegia in MIC-MS

The choice of cardioplegia solution depends on factors such as institutional protocols, patient characteristics and the specific requirements of the surgical procedure. In the studies assessed, two main types of cardioplegia solutions were commonly used, namely crystalloid and blood cardioplegia (24, 58–60).

Both types are efficient and the decision for either one should be adjusted to the individual patient and his or her surgical requirements. In this review the application of crystalloid solutions like Bretschneider and St. Thomas as well as the mode of application are outlined. However, the comparison of blood vs. crystalloid cardioplegia failed to demonstrate any statistically significant difference regarding myocardial infarction or death, with blood cardioplegia showing a lower incidence of postoperative low cardiac output syndrome and lower levels of creatine kinase muscle-brain release (61).

### Systematic impacts of Mi on cardiac anatomy and function

Mitral valve insufficiency affects not only the valve itself but also the heart's anatomy and physiology including other valves, such as the aortic and tricuspid valves. Furthermore, genetic conditions like Marfan syndrome, infective conditions like rheumatic heart disease or degenerative myxomatous changes often involve multiple valves simultaneously. Other conditions that have a systemic effect such as dilated cardiomyopathy, which was overall seen with a median of 3.79% (IQR: 3.55, 4.04), can also lead to cardiac and thereby to valve insufficiency. Hypertension as a common cardiac risk factor increases the afterload on the left ventricle, leading to mitral and aortic regurgitation. In the context of MR, heart failure can be caused by volume overload due to dilation of both ventricles. Consequently, the annular diameter changes, which then leads to a dysfunctional valve. Furthermore, dilation of the left ventricle leads to a change in geometry which inhibits proper coaptation of both leaflets not only of the mitral valve but also of the aortic and tricuspid valve. This is why 18.6% of patients develop mild to moderate aortic insufficiency and 42.8% additionally suffer from mild to severe tricuspid regurgitation.

As a consequence of chronic mitral regurgitation, 35.37% show pulmonary hypertension which further increases the workload on the right ventricle (62).

### Is minimally invasive mitral valve repair with artificial chords reproducible and applicable in routine surgery?

MIMVS with artificial chords, also known as artificial chordae or neochordae, has become an established technique for treating mitral valve regurgitation, particularly in degenerative mitral valve disease. The artificial sutures or chords are applied to support and stabilise the mitral valve leaflets in order to reduce mitral regurgitation.

Whether a patient´s valve is applicable for the usage of neochordae depends on different aspects such as anatomical suitability and severity of mitral regurgitation (37, 63, 64).

The use of polytetrafluoroethylene (PTFE) neochordae for posterior mitral valve prolapse, often referred to as the “loop technique”, which was applied in 42.7% of patients, is an alternative to leaflet resection in the surgical repair of mitral valve prolapse.

However, leaflet resection may be associated with a risk of leaflet tethering or restriction, which can impair valve function and potentially lead to postoperative mitral stenosis or residual regurgitation, seen in 55% of patients. Leaflet resection allows for tissue to be removed and the valve leaflet to be reshaped, however, it may result in loss of leaflet tissue and modification of valve geometry, which may impact long-term durability and function (65).

In terms of reproducibility, several aspects have to be taken into account. Surgical experience plays an important role and the learning curve is steep. Intraoperative assessment of chord length and tension is technically challenging and requires appropriate advanced imaging, preferably 3D echocardiography. In order to avoid residual MR, artificial chords must be anchored correctly. Since chordal placement currently lacks standardisation, its reproducibility is limited and highly operator-dependent. Furthermore, the limited exposure in mini-thoracotomy poses an additional challenge (12, 66, 67).

### MIC-MS and echocardiography

In MIMVS echocardiography plays a crucial role in preoperative assessment, procedural planning, intraoperative decision making and postoperative evaluation. The main echocardiographic data assessed in the reviewed studies includes mitral valve anatomy and function, with 85.2% of patients suffering from mitral insufficiency and 7.2% suffering from mitral stenosis. As regards left ventricular function, with a mean of 52.9% preoperatively and 57% postoperatively, it must be considered that postoperative LVEF was recorded inconsistently in comparison to preoperative data, thus precluding a direct comparison of outcomes. Other relevant data was pulmonary artery pressure [on average 36.7 mmHg (±17.6)] and right heart function. Further findings include possible coexistent aortic valve insufficiency and associated concomitant cardiac conditions such as AF, CAD or other valvular abnormalities (e.g., tricuspid regurgitation).

The echocardiographic parameter LVEF plays a crucial role in evaluating the surgical outcome, as surgery aims at reducing the volume overload on the left atrium. Therefore, a decrease in LA dimensions postoperatively is preferable since it also leads to a positive cardiac remodelling response (68). An enlarged LA postoperatively can induce structural changes that are mostly irreversible and increase the risk for adverse events after surgery, such as new-onset AF, which was seen in 19.2% of patients. Consequently, there is a greater risk of heart failure and thromboembolic events. Long-term outcomes may become less favourable due to ongoing or residual haemodynamic stress (21, 69). Owing to inadequate LA dimensions and new-onset AF as a result, patients might require pacemaker implantation, which was seen in 4% of patients undergoing MIMVS. This underlines the significance of perioperative LA monitoring with a view to postoperative rhythm management and anticoagulation (21).

### Endoaortic clamping in MIMVS and outcome

A commonly used technique in MIMVS is endoaortic clamping or endoaortic balloon occlusion or endoclamp. Occluding the aorta during surgery without using a cross-clamping technique reduces the risk of aortic injury, dissection or embolisation of atherosclerotic plaque.

However, endoaortic clamping requires expertise in catheterisation techniques as well as handling which involves inflating and placing the endoaortic balloon. This is reflected in the reviewed studies, since endoaortic clamping was applied in only 3% of patients (58, 70, 71).

### MIMVS and CX impairment

A possible complication of MV repair is compromised flow in the circumflex artery, which may occur in up to 1.8% of patients (61). This complication can occur during implantation of an annuloplasty ring or band, during suture annuloplasty techniques or during MV replacement surgery since the mitral valve annulus lies adjacent to the circumflex artery, especially in the posterolateral (P1/P2) region. There is controversy as to whether the coronary dominance pattern increases the risk of circumflex injury (62).

Coronary compromise can be a result of ligation or distortion of the vessels during surgery and may result in myocardial infarction. CX impairment was not commonly assessed in the reviewed studies due to its lower prevalence. However, postoperative myocardial infarction as a complication occurred in 0.9% of patients undergoing MIMVS. The precise reason for infarction, however, was not examined.

Compared to these results, CS offers full operative exposure, which allows more precise suture placement and direct visualisation of the CX course. In order to avoid CX injury due to limited access, preoperative CT angiography is indispensable to assess CX proximity to the posterior annulus (72, 73).

### Contraindications for MIMVS

With a view to the patient's aptness for MIMVS, several aspects and data have to be taken into account. This includes an assessment of the valvular and coronary artery anatomy by various imaging techniques, as well as medical comorbidities and surgical history.

Relative contraindications for a minimally invasive approach are aortic calcification, RV dysfunction or severe mitral annular calcification. Patients commonly have pulmonary comorbidities such as severe emphysema, restrictive lung disease and PH which are also critical preconditions for surgery. The Society of Thoracic Surgeons' (STS) risk calculation can be performed to evaluate the surgical risk, which includes mortality and morbidity. In the reviewed studies we saw preferential use of the EuroSCORE (European System for Cardiac Operative Risk Evaluation) for evaluating perioperative mortality. Here, an average score of 3.12 predicts a low chance of mortality associated with the surgery. Nevertheless, it must be taken into account that this score was developed for patients undergoing cardiac surgery under cardiopulmonary bypass via sternotomy. Therefore, it is not 100 percent transferable to predict hospital mortality in patients undergoing MIMVS, and the expected mortality does not confer with the observed mortality. Since preoperative LV function and ejection fraction usually overestimate the true left ventricular function in case of severe MR, severe LV dysfunction must be seen as a relative contradiction for MIMVS (74).

### Mortality in MIMVS

The mortality rate in MIMVS as assessed in this review depends on several pre- and perioperative risk factors, such as advanced age, pre-existing comorbidities such as PH or renal dysfunction and the need for concomitant procedures.

As assessed, in-hospital mortality for MIMVS rates generally range from 1% to 2% and 30-day mortality from 1% to 3%. In-hospital and 30-day mortality were both 1% in this cohort.

However, MICS approaches are generally associated with favourable outcomes compared to open-heart surgery due to lower morbidity and shorter hospital stays. There is a 67% lower in-hospital mortality rate for patients undergoing MIMVS in comparison to full sternotomy cases. Furthermore, patients undergoing MIMVS leave the ICU 26 h and the hospital 2 days earlier (75).

The correlation between aortic clamp time and mortality in MIMVS is an important consideration, as prolonged aortic cross-clamp times can be associated with an increased risk of adverse outcomes, including mortality. In this review we found an average aortic clamp time of 90.63 min (±20.05), with times ranging between 78.8 and 103.7 min (Figure 6D), which are associated with a favourable outcome (25, 76, 77).

## Conclusion

Overall, the review of the available publications on MIMVS does not provide sufficient information on the 30-day mortality rate. However, various factors associated with increased mortality risk and risk of adverse events could be determined.

Conditions and comorbidities that are often associated with high-risk mortality were outlined and analysed in this review.

Comorbidities such as chronic kidney disease, diabetes and CAD are generally associated with high mortality risk in MIMVS. However, the exact impact on postoperative death varied, suggesting that interactions with other conditions must be considered.

The overall 30-day mortality was 3%, though outcomes are likely influenced by a combination of patient-specific factors, comorbidities and perioperative complications. Multidisciplinary evaluation, careful patient selection, a precise surgical technique which requires a high level of surgical expertise and appropriate postoperative management are critical to optimising outcomes, minimising the risk of complications, including mortality, and mitigating risks in MIMVS.

Overall, a minimally invasive approach through right mini-thoracotomy showed more favourable outcomes regarding in-hospital mortality which occurred in 8 patients (±25), reintervention for bleeding with a rate of 4.1% and acute kidney injury necessitating renal replacement therapy in 3.8% of patients. Postoperative complications such as pneumonia with 1.7%, transient neurocognitive dysfunction in 4.4% of patients or cerebral stroke with an occurrence of 1.1% remain low. Conversion to sternotomy was necessary in 2% of MIMVS cases and 8% of patients showed signs of residual MR. Therefore, right mini-thoracotomy represents a safe alternative to median sternotomy for patients undergoing mitral valve surgery.

## Data Availability

The original contributions presented in the study are included in the article/Supplementary Material, further inquiries can be directed to the corresponding author.
