# Supplementary material for: The impact of comorbidities on surgical outcome and mortality in minimally invasive mitral valve surgery: a systematic review
Source: Front Cardiovasc Med. 2025 Sep 2;12:1638217. doi: 10.3389/fcvm.2025.1638217 (PMC12436284; doi:10.3389/fcvm.2025.1638217)
Supplement: Supplementary file 1 [file Table1.docx]

## Supplement

**Table 1.0:** List of publications

| No | Authors | Title | DOI | Journal | N | Date |
| --- | --- | --- | --- | --- | --- | --- |
| 1 | van Praet et al. | Factors associated with an unsuccessful fast-track course following minimally invasive surgical mitral valve repair | DOI: 10.1093/ejcts/ezac451 | European Journal of Cardio-Thoracic Surgery | 491 | 2022/09 |
| 2 | Seeburger et al. | Minimal invasive mitral valve repair for mitral regurgitation: results of 1339 consecutive patients | DOI: 10.1016/j.ejcts.2008.05.015 | European Journal of Cardio-Thoracic Surgery | 1536 | 2008/10 |
| 3 | Ntinopoulos et al. | Isolated Minimally Invasive Mitral Valve Surgery in Octogenarians: Perioperative Outcome | DOI: 10.1159/000533560 | Gerontology | 38 | 2023/08 |
| 4 | Berdajs et al. | Minimally Invasive Nonresectional Mitral Valve Repair Long-term Results | DOI: 10.1016/j.cjca.2023.03.027 | Canadian Journal of Cardiology | 365 | 2023/04 |
| 5 | Shu et al. | Attaining competency and proficiency in minimally invasive mitral valve repair: a learning curve assessment using cumulative sum analysis | DOI: 10.1186/s13019-023-02106-7 | Journal of Cardiothoracic Surgery | 150 | 2023/01 |
| 6 | De Praetere et al. | Starting minimally invasive valve surgery using endoclamp technology: safety and results of a starting surgeon | DOI: 10.1093/icvts/ivu394 | Interactive CardioVascular and Thoracic Surgery | 138 | 2015/03 |
| 7 | Cheng et al. | Minimal Invasive Thoracoscopic Mitral Valve Surgery | DOI: 10.21470/1678-9741-2020-0260 | Brazilian Journal of Cardiothoracic Surgery | 96 | 2020/10 |
| 8 | Moscarelli et al. | Sex-specific differences and postoperative outcomes of minimally invasive and sternotomy valve surgery | DOI: 10.1093/ejcts/ezab369 | European Journal of Cardio-Thoracic Surgery | 15155 (7674 MICS) | 2022/02 |
| 9 | Akowuah et al. | Minithoracotomy vs Conventional Sternotomy for Mitral Valve Repair: A Randomized Clinical Trial | DOI: 10.1001/jama.2023.7800 | JAMA | 166 | 2023/06 |
| 10 | Nasso et al. | Three-year results of repaired Barlow mitral valves via right minithoracotomy versus median sternotomy in a randomized trial | DOI: 10.1159/000357263 | Cardiology | 80 | 2014 |
| 11 | Speziale et al. | Results of mitral valve repair for Barlow disease (bileaflet prolapse) via right minithoracotomy versus conventional median sternotomy: a randomized trial | DOI: 10.1016/j.jtcvs.2010.08.033 | The Journal of Thoracic and Cardiovascular Surgery | 70 | 2011/07 |
| 12 | Tünerir et al. | An alternative, less invasive approach to median sternotomy for cardiac operations in adults: right infra-axillary minithoracotomy | DOI: 10.1177/147323000503300107 | Journal of International Medical Research | 29 | 2005/01 |
| 13 | Yildrim et al. | Left atrial strain predicts the rhythm outcome in patients with persistent atrial fibrillation undergoing left atrial cryoablation during minimally invasive mitral valve repair | DOI: 10.3389/fcvm.2024.1373310 | Frontiers in Cardiovascular medicine | 72 | 2024/03 |
| 14 | Gerber et al. | Six-year single-centre experience in minimally invasive mitral valve repair - impact of the team learning curve on in-hospital clinical outcome | DOI: 10.5114/kitp.2019.83942 | Polish Journal of Thoracic and Cardiac Surgery | 173 | 2019/03 |
| 15 | Mork et al- | Bretschneider (Custodiol®) and St. Thomas 2 Cardioplegia Solution in Mitral Valve Repair via Anterolateral Right Thoracotomy: A Propensity-Modelled Comparison | DOI: 10.1155/2019/5648051 | Mediators of inflammation | 184 | 2019/12 |
| 16 | Falk et al. | How does the use of polytetrafluoroethylene neochordae for posterior mitral valve prolapse (loop technique) compare with leaflet resection? A prospective randomized trial | DOI: 10.1016/j.jtcvs.2008.07.028 | The Journal of Thoracic and Cardiovascular Surgery | 129 | 2008/11 |
| 17 | Onnasch et al. | Five years of less invasive mitral valve surgery: from experimental to routine approach | https://journal.hsforum.com/index.php/HSF/article/view/6197/7991 | The heart surgery forum | 449 | 2002/06 |
| 18 | Ram et al. | Three-dimensional Video Assistance Improves Early Results in Minimally Invasive Mitral Valve Surgery | DOI: 10.1097/MAT.0000000000001326 | American Society for Internal Organs Journal | 152 | 2021/07 |
| 19 | Santana et al. | Outcomes of minimally invasive mitral valve surgery in patients with an ejection fraction of 35% or less | DOI: 10.1097/IMI.0b013e31828da226 | Innovations (Philadelphia) | 71 | 2013/01 |
| 20 | Feirer et al. | Non-robotic minimally invasive mitral valve repair: a 20-year single-centre experience | DOI: 10.1093/ejcts/ezac223 | European Journal of Cardio-Thoracic Surgery | 1194 | 2022/10 |
| 21 | Nakayama et al. | Early and mid-term outcomes of minimally invasive mitral valve repair via right mini-thoracotomy: 5-year experience with 129 consecutive patients | DOI: 10.1007/s11748-020-01573-2 | General Thoracic and Cardiovascular Surgery | 141 | 2021/08 |
| 22 | McClure et al. | Early and late outcomes in minimally invasive mitral valve repair: an eleven-year experience in 707 patients | DOI: 10.1016/j.jtcvs.2008.08.058 | The Journal of Thoracic and Cardiovascular Surgery | 707 | 2009/01 |
| 23 | Glauber et al. | Early and long-term outcomes of minimally invasive mitral valve surgery through right minithoracotomy: a 10-year experience in 1604 patients | DOI: 10.1186/s13019-015-0390-y | Journal of Cardiothoracic Surgery | 1604 | 2015/12 |
| 24 | McClure et al. | One thousand minimally invasive mitral valve operations: early outcomes, late outcomes, and echocardiographic follow-up | DOI: 10.1016/j.jtcvs.2012.12.070 | The Journal of Thoracic and Cardiovascular Surgery | 1000 | 2013/05 |
| 25 | Akowuah et al. | Early and Late Outcomes After Minimally Invasive Mitral Valve Repair Surgery |  | The journal of heart valve disease | 190 | 2015/07 |
| 26 | Cui et al. | Early clinical outcomes of thoracoscopic mitral valvuloplasty: a clinical experience of 100 consecutive cases | DOI: 10.21037/cdt-20-440 | Cardiovascular diagnosis and therapy | 100 | 2020/08 |
| 27 | da Rocha eSilva et al. | Barlow's Mitral Valve Disease: A Comparison of Neochordal (Loop) and Edge-To-Edge (Alfieri) Minimally Invasive Repair Techniques | DOI: 10.1016/j.athoracsur.2015.05.097 | The Annals of Thoracic Surgery | 123 | 2015/12 |
| 28 | Mazine et al. | Very high repair rate using minimally invasive surgery for the treatment of degenerative mitral insufficiency | DOI: 10.1016/j.cjca.2014.12.029 | The Canadian Journal of Cardiology | 200 | 2015/06 |
| 29 | Pojar et al. | Single-Center Experience with Minimally Invasive Mitral Operations through Right Minithoracotomy | DOI: 10.5761/atcs.oa.18-00100 | The Annals of Thoracic Surgery | 151 | 2019/02 |
| 30 | Agnino et al. | Strategy-specific durability of mitral valve repair through the video-assisted right minithoracotomy approach | DOI: 10.2459/JCM.0000000000000753 | Journal of Cardiovascular Medicine | 241 | 2019/03 |
| 31 | Faerber et al. | Minimally-invasive mitral valve repair of symmetric and asymmetric Barlow´s disease | DOI: 10.1007/s00392-021-01844-9 | Clinical Research in Cardiology | 103 | 2021/12 |
| 32 | Santana et al. | Outcomes of minimally invasive double valve surgery | DOI: 10.21037/jtd.2017.05.62 | Journal of Thoracic disease | 117 | 2017/06 |
| 33 | Santana et al. | Hybrid approach of percutaneous coronary intervention followed by minimally invasive mitral valve surgery: a 5-year single-center experience | DOI: 10.21037/jtd.2017.06.29 | Journal of Thoracic disease | 93 | 2017/06 |
| 34 | Panos et al. | Is minimally invasive mitral valve repair with artificial chords reproducible and applicable in routine surgery? | DOI: 10.1093/icvts/ivv065 | Interactive CardioVascular and Thoracic Surgery | 426 | 2015/06 |
| 35 | Moscoso-Luduena et al. | Combined Minimally Invasive Mitral Valve Surgery and Percutaneous Coronary Intervention: A Hybrid Concept for Patients with Mitral Valve and Coronary Pathologies | DOI: 10.3390/jcm12175553 | Journal of Clinical Medicine | 10 (534) | 2023/08 |
| 36 | Mihos et al. | Percutaneous Coronary Intervention Followed by Minimally Invasive Mitral Valve Surgery in Ischemic Mitral Regurgitation | DOI: 10.1097/IMI.0000000000000218 | Innovations (Philadelphia) | 31 | 2015/11 |
| 37 | Yoo et al. | Echocardiographic assessment of mitral durability in the late period following mitral valve repair: minithoracotomy versus conventional sternotomy | DOI: 10.1016/j.jtcvs.2013.05.042 | Journal of thoracic and cardiovascular surgery | 179 | 2014/05 |
| 38 | Kim et al. | Totally endoscopic mitral valve repair using a three-dimensional endoscope system: initial clinical experience in Korea | DOI: 10.21037/jtd.2019.12.126 | Journal of Thoracic disease | 40 | 2020/03 |
| 39 | Borger et al. | Minimally invasive mitral valve repair in Barlow's disease: early and long-term results | DOI: 10.1016/j.jtcvs.2013.11.030 | Journal of thoracic and cardiovascular surgery | 145 | 2014/10 |
| 40 | Pausch et al. | Early outcome of endoscopic mitral valve surgery in elderly patients: a high-volume single center experience | DOI: 10.3389/fcvm.2023.1182752 | Frontiers in Cardiovascular medicine | 91 | 2023/11 |
| 41 | van Praet et al. | Periareolar endoscopic minimally invasive cardiac surgery: postoperative scar assessment analysis | DOI: 10.1093/icvts/ivac200 | Interactive CardioVascular and Thoracic Surgery | 109 | 2022/07 |
| 42 | Brega et al. | Periareolar approach in female patients undergoing mitral and tricuspid valve surgery: An almost invisible surgical access | DOI: 10.1111/jocs.16693 | Journal of Cardiothoracic Surgery | 57 | 2022/09 |
| 43 | Faerber et al. | Right Mini-Thoracotomy for Aortic Plus Mitral with or without Tricuspid Valve Surgery | DOI: 10.1055/s-0040-1721083 | The thoracic and cardiovascular surgeon | 25 | 2022/04 |
| 44 | de Oliveira et al. | In-Hospital Outcomes of Right Minithoracotomy vs. Periareolar Access for Minimally Invasive Video-Assisted Mitral Valve Repair | DOI: 10.21470/1678-9741-2020-0507 | Brazilian Journal of Cardiothoracic Surgery | 37 | 2022/03 |
| 45 | Axtell et al. | Minimally Invasive Nonresectional Mitral Valve Repair Can Be Performed With Excellent Outcomes | DOI: 10.1016/j.athoracsur.2019.07.029 | The Annals of Thoracic Surgery | 101 | 2020/02 |
| 46 | Akansel et al. | Image-based ring size prediction for mitral valve repair | DOI: 10.1093/ejcts/ezad212 | European Journal of Cardio-Thoracic Surgery | 150 | 2023/07 |
| 47 | van Praet et al. | Single-Center Experience With a Self-Expandable Venous Cannula During Minimally Invasive Cardiac Surgery | DOI: 10.1177/15569845221131534 | Innovations (Philadelphia) | 58 | 2022/11 |
| 48 | Kofler et al. | Minimally invasive surgery versus sternotomy in native mitral valve endocarditis: a matched comparison | DOI: 10.1093/ejcts/ezab364 | European Journal of Cardio-Thoracic Surgery | 42 | 2021/12 |
| 49 | Mihos et al. | Right anterior minithoracotomy versus median sternotomy surgery for native mitral valve infective endocarditis | ? | Journal of Heart Valve Disease | 22 | 2014/05 |
| 50 | Lamelas et al. | Outcomes of minimally invasive valve surgery versus median sternotomy in patients age 75 years or greater | DOI: 10.1016/j.athoracsur.2010.09.019 | The Annals of Thoracic Surgery | 119 | 2011/01 |
| 51 | Hasde et al. | Single or Combined Valve Surgery and Concomitant Right Coronary Artery Bypass through Right Anterior Minithoracotomy Approach | DOI: 10.1055/s-0041-1731284 | Journal of thoracic and cardiovascular surgery | 28 | 2023/12 |
| 52 | Paparella et al. | Minimally invasive heart valve surgery: influence on coagulation and inflammatory response | DOI: 10.1093/icvts/ivx090 | Interactive CardioVascular and Thoracic Surgery | 20 | 2017/08 |
| 53 | Squiccinarro et al. | Mid-term results of endoscopic mitral valve repair and insights in surgical techniques for isolated posterior prolapse | DOI: 10.1186/s13019-023-02352-9 | Journal of Cardiothoracic Surgery | 309 | 2023/08 |
| 54 | Dogan et al. | Minimally Invasive Port Access Versus Conventional Mitral Valve Surgery: Prospective Randomized Study | DOI: 10.1016/j.athoracsur.2004.08.066 | The Annals of Thoracic Surgery | 20 | 2005/02 |
| 55 | Cui et al. | Dexmedetomidine Improves Lung Function by Promoting Inflammation Resolution in Patients Undergoing Totally Thoracoscopic Cardiac Surgery | DOI: 10.1155/2020/8638301 | Oxidative medicine cellular longevity | 57 | 2020/09 |
| 56 | Moscarelli et al. | A Trial of Two Anesthetic Regimes for Minimally Invasive Mitral Valve Repair | DOI: 10.1053/j.jvca.2018.01.028 | Journal of Cardiothoracic and Vascular Anaesthesia | 62 | 2018/12 |
| 57 | Samalavicius et al. | Anesthetic Management and Procedural Outcomes of Patients Undergoing Off-Pump Transapical Implantation of Artificial Chordae to Correct Mitral Regurgitation: Case Series of 76 Patients | DOI: 10.1213/ANE.0000000000002767 | Anaesthesia and Anelgesia | 76 | 2018/03 |
| 58 | Baumbach et al. | Minimally Invasive Extracorporeal Bypass in Minimally Invasive Heart Valve Operations: A Prospective Randomized Trial | DOI: 10.1016/j.athoracsur.2016.01.043 | The Annals of Thoracic Surgery | 101 | 2016/07 |
| 59 | Mo et al. | Mitral valve replacements under on-pump beating heart and lung perfusion/ventilation using a minithoracotomy: an experience with 11 cases | DOI: 10.1532/HSF98.20121013 | Heart Surgery Forum | 11 | 2012/06 |
| 60 | Mukherjee et al. | Intrathecal morphine is superior to intravenous PCA in patients undergoing minimally invasive cardiac surgery | DOI: 10.4103/0971-9784.95075 | Annals of Cardiac Anaesthesia | 61 | 2012/04 |
| 61 | Shariff et al. | Minimally Invasive Valve Surgery and Single Vessel Coronary Artery Bypass via Limited Anterior Right Thoracotomy | DOI: 10.1532/hsf.1319 | Heart Surgery Forum | 6 | 2015/12 |
| 62 | Seeburger et al. | Comparison of outcomes of minimally invasive mitral valve surgery for posterior, anterior and bileaflet prolapse | DOI: 10.1016/j.ejcts.2009.03.058 | European Journal of Cardio-Thoracic Surgery | 1230 | 2009/09 |
| 63 | Del Forno et al. | Neochordae implantation versus leaflet resection in mitral valve posterior leaflet prolapse and dilated left ventricle: a propensity score matching comparison with long-term follow-up | DOI: 10.1093/ejcts/ezad274 | European Journal of Cardio-Thoracic Surgery | 332 | 2023/10 |
| 64 | Kuntze et al. | Early and mid-term results of mitral valve repair using premeasured Gore-Tex loops ('loop technique') | DOI: 10.1016/j.ejcts.2008.01.013 | European Journal of Cardio-Thoracic Surgery | 522 | 2008/04 |
| 65 | Papadopoulos et al. | Navigating the challenges of minimally invasive mitral valve surgery: a risk analysis and learning curve evaluation | DOI: 10.1186/s13019-024-02479-3 | Journal of Cardiothoracic Surgery | 266 | 2024/01 |
| 66 | Doenst et al. | Aortic cross-clamp time correlates with mortality in the mini-mitral international registry | DOI: 10.1093/ejcts/ezad147 | European Journal of Cardio-Thoracic Surgery | 6878 | 2023/06 |
| 67 | Kakuta et al. | Long-term outcome of isolated mitral valve repair versus replacement for degenerative mitral regurgitation in propensity-matched patients | DOI: 10.1016/j.xjon.2023.12.003 | JTCVS | 1493 | 2023/12 |
| 68 | Missault et al. | Analysis of clinical outcome and postoperative organ function effects in a propensity-matched comparison between conventional and minimally invasive mitral valve surgery | DOI: 10.1111/jocs.15010 | Journal of Cardiothoracic Surgery | 143 | 2020/12 |
| 69 | Zanobini et al. | Postoperative Echocardiographic Reduction of Right Ventricular Function: Is Pericardial Opening Modality the Main Culprit? | DOI: 10.1155/2017/4808757 | Biomed Research International | 17 | 2017/05 |
| 70 | Wu et al. | Surgical repair of mitral valve prolapse through a minimal right vertical infraaxillary thoracotomy | DOI: 10.1111/j.1540-8191.2012.01499.x | Journal of Cardiac Surgery | 68 | 2012/09 |
| 71 | Heuts et al. | One-year postprocedural quality of life following mitral valve surgery: data from The Netherlands heart registration | DOI: 10.1093/icvts/ivae051 | Interdisciplinary Cardiovascular and thoracic surgery | 209 | 2024/03 |
| 72 | Olsthoorn et al. | Effect of minimally invasive mitral valve surgery compared to sternotomy on short- and long-term outcomes: a retrospective multicentre interventional cohort study based on Netherlands Heart Registration | DOI: 10.1093/ejcts/ezab507 | European Journal of Cardio-Thoracic Surgery | 725 | 2022/05 |
| 73 | Olsthoorn et al. | Minimally invasive approach compared to resternotomy for mitral valve surgery in patients with prior cardiac surgery: retrospective multicentre study based on the Netherlands Heart Registration | DOI: 10.1093/ejcts/ezac420 | European Journal of Cardio-Thoracic Surgery | 85 | 2022/10 |
| 74 | van Kampen et al. | Building a successful minimally invasive mitral valve repair program before introducing the robotic approach: The Massachusetts General Hospital experience | DOI: 10.3389/fcvm.2023.1113908 | Frontiers in Cardiovascular medicine | 261 | 2023/03 |
| 75 | Zheng et al. | Robotic vs. minimally invasive mitral valve repair: A 5-year comparison of surgical outcomes | DOI: 10.1111/jocs.16849 | Journal of Cardiac Surgery | 424 | 2022/10 |
| 76 | Downs et al. | Minimally Invasive Mitral Valve Surgery Provides Excellent Outcomes Without Increased Cost: A Multi-Institutional Analysis | DOI: 10.1016/j.athoracsur.2016.01.084 | The Annals of Thoracic Surgery | 425 | 2016/07 |
| 77 | Hawkins et al. | Minimally invasive mitral valve surgery is associated with excellent resource utilization, cost, and outcomes | DOI: 10.1016/j.jtcvs.2018.03.108 | Journal of thoracic and cardiovascular surgery | 74 | 2018/04 |
| 78 | Hage et al. | Endoscopic Mitral Repair for Degenerative Mitral Regurgitation: Effect of Disease Complexity on Short- and Mid-term Outcomes | DOI: 10.1016/j.cjco.2020.04.005 | CJC (open) | 245 | 2020/04 |
| 79 | Oezpeker et al. | Isolated annuloplasty in elderly patients with secondary mitral valve regurgitation: short- and long-term outcomes with a less invasive approach | DOI: 10.3389/fcvm.2023.1193156 | Frontiers in Cardiovascular medicine | 67 | 2023/10 |
| 80 | Oezpeker et al. | An Individualized, Less-Invasive Surgical Approach Algorithm Improves Outcome in Elderly Patients Undergoing Mitral Valve Surgery | DOI: 10.3390/jcdd10010028 | Journal of Cardiovascular development and disease | 274 | 2023/01 |
| 81 | Miceli et al. | Minimally invasive mitral valve repair through right minithoracotomy in the setting of degenerative mitral regurgitation: early outcomes and long-term follow-up | DOI: 10.3978/j.issn.2225-319X.2015.04.10 | Annals of Cardiothoracic Surgery | 703 | 2015/09 |
| 82 | Sakaguchi et al. | Minimally Invasive Mitral Valve Repair Through Right Minithoracotomy - 11-Year Single Institute Experience | DOI: 10.1253/circj.CJ-17-1319 | Circulation Journal | 387 | 2018/05 |
| 83 | Belluschi et al. | Excellent long-term results with minimally invasive edge-to-edge repair in myxomatous degenerative mitral valve regurgitation | DOI: 10.1093/icvts/ivaa048 | Interactive CardioVascular and Thoracic Surgery | 97 | 2020/07 |
| 84 | De Bonis et al. | Minimally invasive or conventional edge-to-edge repair for severe mitral regurgitation due to bileaflet prolapse in Barlow's disease: does the surgical approach have an impact on the long-term results? | DOI: 10.1093/ejcts/ezx032 | European Journal of Cardio-Thoracic Surgery | 104 | 2017/07 |
| 85 | Muneretto et al. | Results of minimally invasive, video-assisted mitral valve repair in advanced Barlow's disease with bileaflet prolapse | DOI: 10.1093/ejcts/ezu166 | European Journal of Cardio-Thoracic Surgery | 50 | 2015/01 |
| 86 | Sakaguchi et al. | Stepwise mitral valve repair for Barlow's disease via a minimally invasive approach | DOI: 10.1111/jocs.14615 | Journal of Cardiac Surgery | 292 | 2020/07 |
| 87 | Hoogma et al. | Efficacy of erector spinae plane block for minimally invasive mitral valve surgery: Results of a double-blind, prospective randomized placebo-controlled trial | DOI: 10.1016/j.jclinane.2023.111072 | Journal of Clinical Anaesthesia | 72 | 2023/06 |
|  |  |  |  |  |  |  |
| 88 | Bainbridge et al. | Percutaneous superior vena cava drainage during minimally invasive mitral valve surgery: a randomized, crossover study | DOI: 10.1053/j.jvca.2014.07.020 | Journal of Cardiothoracic and Vascular Anaesthesia | 28 | 2014/11 |
| 90 | Hamano et al. | Stress caused by minimally invasive cardiac surgery versus conventional cardiac surgery: incidence of systemic inflammatory response syndrome | DOI: 10.1007/s002680020048 | World Journal of Surgery | 21 | 2001/02 |
| 91 | Reichenspurner et al. | Video and robotic-assisted minimally invasive mitral valve surgery: a comparison of the Port-Access and transthoracic clamp techniques | DOI: 10.1016/j.athoracsur.2004.06.120 | The Annals of Thoracic Surgery | 120 | 2005/02 |
| 89 | Santana et al. | Minimally invasive papillary muscle sling placement during mitral valve repair in patients with functional mitral regurgitation | DOI: 10.1016/j.jtcvs.2013.03.006 | The Journal of Cardiovascular Surgery | 19 | 2014/01 |
| 92 | Hata et al. | A 25-year study of chordal replacement with expanded polytetrafluoroethylene in mitral valve repair† | DOI: 10.1093/icvts/ivu441 | Interactive CardioVascular and Thoracic Surgery | 224 | 2015/04 |
| 93 | Lin et al. | Early Clinical Outcomes of Thoracoscopic Mitral Valvuloplasty: The First 90 Cases | DOI: 10.1532/hsf.4807 | Heart Surgery Forum | 90 | 2022/09 |
| 94 | Mazine et al. | Minimally invasive mitral valve surgery: influence of aortic clamping technique on early outcomes | DOI: 10.1016/j.athoracsur.2013.07.015 | The Annals of Thoracic Surgery | 259 | 2013/12 |
| 95 | Casselman et al. | Endoaortic Clamping Does Not Increase the Risk of Stroke in Minimal Access Mitral Valve Surgery: A Multicenter Experience | DOI: 10.1016/j.athoracsur.2015.04.003 | The Annals of Thoracic Surgery | 500 | 2015/10 |
| 96 | Murzi et al | Minimally invasive right thoracotomy approach for mitral valve surgery in patients with previous sternotomy: a single institution experience with 173 patients | DOI: 10.1016/j.jtcvs.2014.07.108 | Journal of thoracic and cardiovascular surgery | 173 | 2014/12 |
| 97 | Murzi et al. | Antegrade and retrograde perfusion in minimally invasive mitral valve surgery with transthoracic aortic clamping: a single-institution experience with 1632 patients over 12 years | DOI: 10.1093/icvts/ivw370 | Interactive CardioVascular and Thoracic Surgery | 1632 | 2017/03 |
| 98 | Huang et al. | Early outcomes of mitral valvuloplasty by minimally invasive surgery or sternotomy | DOI: 10.1177/0218492320911756 | Asian Cardiovascular & Thoracic annals | 225 | 2020/03 |
| 99 | Balkhy et al. | A Retrospective Evaluation of Endo-Aortic Balloon Occlusion Compared to External Clamping in Minimally Invasive Mitral Valve Surgery | DOI: 10.1053/j.semtcvs.2022.11.016 | Seminars in throracic and cardiovascular surgery | 7978 | 2024/03 |
| 100 | Bentala et al. | Comparing the endo-aortic balloon and the external aortic clamp in minimally invasive mitral valve surgery | none | Interdisciplinary Cardiovascular and thoracic surgery | 340 | 2015/09 |
| 101 | Barbero et al. | Clinical Impact of the Endo-aortic Clamp for Redo Mitral Valve Surgery | DOI: 10.1007/s12265-024-10509-7 | Journal of cardiovascular tranlsational research | 216 | 2024/04 |
| 102 | Schumacher et al. | Early and long-term outcomes following redo mitral valve surgery in patients with prior minimally invasive mitral valve surgery | DOI: 10.1093/icvts/ivae042 | Interdisciplinary Cardiovascular and thoracic surgery | 187 | 2024/04 |
| 103 | Mkalaluh et al. | Early and long-term results of minimally invasive mitral valve surgery through a right mini-thoracotomy approach: a retrospective propensity-score matched analysis | DOI: 10.7717/peerj.4810 | PeerJournal | 227 | 2018/05 |
| 104 | Yasar et al. | Minimally invasive versus conventional mitral valve surgery: A propensity score matching analysis | DOI: 10.5606/tgkdc.dergisi.2023.25404 | Turk Gogus Kalp Damar Cerrahisi Dergisi | 97 | 2023/10 |
| 105 | Grant et al. | Propensity-matched analysis of minimally invasive approach versus sternotomy for mitral valve surgery | DOI: 10.1136/heartjnl-2018-314049 | Heart (British Cardiac Society) | 647 | 2019/05 |
| 106 | Lange et al,. | Right Minithoracotomy Versus Full Sternotomy for Mitral Valve Repair: A Propensity Matched Comparison | DOI: 10.1016/j.athoracsur.2016.06.055 | Annals of Thoracic Surgery | 501 | 2017/02 |
| 107 | Zhao et al. | Combined Mitral and Aortic Valve Procedure via Right Mini-Thoracotomy versus Full Median Sternotomy | DOI: 10.1536/ihj.18-186 | International heart journal | 98 | 2019/03 |
| 108 | Alkady et al. | A Simple Approach for Minimally Invasive Combined Aortic and Mitral Valve Surgery | DOI: 10.1055/s-0041-1740240 | The thoracic and cardiovascular surgeon | 72 | 2022/03 |
| 109 | Goldsstone et al. | Minimally invasive approach provides at least equivalent results for surgical correction of mitral regurgitation: a propensity-matched comparison | DOI: 10.1016/j.jtcvs.2012.09.093 | Journal of thoracic and cardiovascular surgery | 556 | 2013/03 |
| 110 | Bifulco et al. | Minimally Invasive Trans-Axillary versus Full Sternotomy Mitral Valve Repair: A Propensity Score-Matched Analysis on Mid-Term Outcomes | DOI: 10.3390/medicina60010029 | Medicina | 308 | 2023/12 |
| 111 | Malvindi et al. | Transaxillary approach enhances postoperative recovery after mitral valve surgery | DOI: 10.1093/ejcts/ezad207 | European Journal of Cardio-Thoracic Surgery | 454 | 2023/07 |
| 112 | Taghizadeh-Waghefi et al. | Safety and Efficacy of the Transaxillary Access for Minimally Invasive Mitral Valve Surgery-A Propensity Matched Competitive Analysis | DOI: 10.3390/medicina58121850 | Medicina | 160 | 2022/12 |
| 113 | Perin et al. | Use of an automated knot fastener shortens operative times in minimally invasive mitral valve repair | DOI: 10.1308/rcsann.2019.0063 | Annals of the Royal College of Surgeons of England | 108 | 2019/09 |
| 114 | Grapow et al. | Automated fastener versus manually tied knots in minimally invasive mitral valve repair: impact on operation time and short- term results | DOI: 10.1186/s13019-015-0344-4 | Journal of Cardiothoracic Surgery | 60 | 2015/11 |
| 115 | Gollmann-Tepeköylü et al. | A qualitative improvement program for minimally invasive mitral surgery: technical advancements ameliorate outcome and operative times | DOI: 10.1093/icvts/ivad030 | Interdisciplinary Cardiovascular and thoracic surgery | 1000 | 2023/03 |
| 116 | Kilic et al. | Operative Outcomes of Concomitant Minimally Invasive Mitral and Tricuspid Valve Surgery | DOI: 10.1177/1556984519864939 | Innovations (Philadelphia) | 238 | 2019/10 |
| 117 | Huang et al. | Concomitant Tricuspid Annuloplasty in Patients Undergoing Totally Endoscopic Mitral Valve Surgery: A Propensity-Score Matched Analysis | DOI: 10.1532/hsf.3675 | Heart Surgery Forum | 173 | 2021/06 |
| 118 | Ko et al. | Minimally invasive mitral valve surgery: a systematic safety analysis | DOI: 10.1136/openhrt-2020-001393 | Open Heart | 745 | 2020/10 |
| 119 | Radwan et al. | Propensity-Matched Comparison of Two Different Access Modes for Minimally Invasive Mitral Valve Surgery | DOI: 10.1053/j.semtcvs.2019.08.008 | Seminars in throracic and cardiovascular surgery | 903 | 2020/08 |
| 120 | Raanani et al. | Quality of mitral valve repair: median sternotomy versus port-access approach | DOI: 10.1016/j.jtcvs.2009.09.035 | Journal of Cardiovascular Surgery | 61 | 2010/07 |
| 121 | Barbero et al. | Endo-Aortic vs. Trans-Thoracic Clamping in Right Mini-Thoracotomy Mitral Valve Surgery: Outcome on Myocardial Protection | DOI: 10.3389/fcvm.2021.719687 | Frontiers in Cardiovascular medicine | 117 | 2021/09 |
| 122 | Malvindi et al. | del Nido and Histidine-Tryptophan-Ketoglutarate cardioplegia in minimally invasive mitral valve surgery: A propensity-Match study | DOI: 10.1177/02676591231161920 | Perfusion | 120 | 2024/05 |
| 123 | Yost et al. | Endoaortic balloon occlusion versus transthoracic cross-clamp for totally endoscopic robotic mitral valve surgery: a retrospective cohort study | DOI: 10.1007/s11701-023-01654-3 | Journal of Robotic Surgery | 113 | 2023/10 |
| 124 | Breves et al. | Ascending Aortic Endoballoon Occlusion Feasible Despite Moderately Enlarged Aorta to Facilitate Robotic Mitral Valve Surgery | DOI: 10.1097/IMI.0000000000000291 | Innovations (Philadelphia) | 196 | 2016/09 |
| 125 | Ambur et al. | Axillary Artery Access for Combined Endoaortic Balloon Occlusion and Perfusion During Robotic Mitral Valve Surgery | DOI: 10.1097/IMI.0000000000000251 | Innovations (Philadelphia) | 1 | 2016/05 |
| 126 | Ward et al. | Outcomes of peripheral perfusion with balloon aortic clamping for totally endoscopic robotic mitral valve repair | DOI: 10.1016/j.jtcvs.2014.05.035 | Journal of thoracic and cardiovascular surgery | 108 | 2014/12 |
| 127 | Haddad et al. | Echocardiographic right ventricular evaluation in cardiac surgery patients undergoing mitral valve reconstruction: a single center prospective observational study | doi: 10.21037/jtd-23-1727 | Journal of Thoracic disease | 92 | 2024/04 |
| 128 | Ertugay et al. | Fine tuning for totally endoscopic mitral valve surgery: ERAS applications | doi: 10.3389/fcvm | Frontiers in Cardiovascular medicine | 113 | 2024/10 |
| 129 | Stelzmueller et al. | Pushing boundaries in cardiac surgery: minimally invasive mitral valve repair combined with tricuspid valve repair and/or other concomitant procedures | doi: 10.3389/fcvm. | Frontiers in Cardiovascular medicine | 153 | 2024/08 |
| 130 | Pölzl et al. | Five-year outcomes of different techniques for minimally invasive mitral valve repair in Barlow's disease | doi: 10.1093/ejcts/ezae213 | European Journal of Cardio-Thoracic Surgery | 246 | 2024/06 |
| 131 | Francica et al. | Minimally Invasive Mitral Valve Surgery in Elderly Patients: Results from a Multicenter Study | DOI: 10.3390/jcm13216320 | Journal of Clinical Medicine | 238 | 2024/10 |
| 132 | Chartrain et al. | A Historical Perspective and Update on Robotic Mitral Valve Surgery | DOI: 10.3390/jcm13216375 | Journal of Clinical Medicine | 1413 | 2024/10 |
| 133 | Gardner-Hilbert et al. | Incidence of Clinical Outcomes in Minimally Invasive Valvular Surgery at the Ignacio Chávez National Institute of Cardiology | DOI: 10.7759/cureus.69859 | Cureus | 67 | 2024/09 |
| 134 | Carrara et al. | Relationship between Preoperative Red Cell Distribution Width and Prolonged Postoperative Use of Catecholamines in Minimally Invasive Mitral Valve Surgery Patients: A Retrospective Cohort Study | DOI: 10.3390/jcm13195736 | Journal of Clinical Medicine | 343 | 2024/09 |
| 135 | Barbero et al. | Comparison of Endoaortic and Transthoracic Aortic Clamping in Less-Invasive Mitral Valve Surgery | DOI: 10.1016/j.athoracsur.2017.09.054 | Annals of thoracic surgery | 500 | 2018/03 |
| 136 | Iaccarino et al. | Survival and Durability of Minimally Invasive Mitral Valve Repair: Insights from Different Repair Techniques | DOI: 10.3390/medsci12030046 | Medical Sciences | 538 | 2024/09 |
| 137 | Morimoto et al. | Efficacy and Outcomes of Para-Annular Plication in Mitral Valve Repair via Right Mini-Thoracotomy | DOI: 10.7759/cureus.67623 | Cureus | 7 | 2024/08 |
| 138 | Brown et al. | Patient-reported outcome measures after minimally invasive mitral valve surgery: The benefit may be early | DOI: 10.1016/j.xjon.2024.05.010 | Journal of Cardiovascular Surgery | 37 | 2024/05 |
| 139 | Berretta et al. | Impact of Complex Anatomy and Patient Risk Profile in Minimally Invasive Mitral Valve Surgery | DOI: 10.1016/j.athoracsur.2024.07.050 | Annals of Thoracic Surgery | 7513 | 2024/08 |
| 140 | Stock et al. | Feasibility of deescalating postoperative care in enhanced recovery after cardiac surgery | DOI: 10.3389/fcvm.2024.1412869 | Frontiers in Cardiovascular medicine | 297 | 2024/08 |
| 141 | Yoon et al. | Long-term outcomes of minimally invasive concomitant mitral and tricuspid valve surgery with surgical ablation | DOI: 10.1093/icvts/ivae146 | Interdisciplinary Cardiovascular and thoracic surgery | 360 | 2024/09 |
| 142 | Shimokawa et al. | Minimally invasive cardiac surgeries in 2021: annual report by Japanese society of minimally invasive cardiac surgery | DOI: 10.1007/s11748-024-02066-2 | General Thoracic and Cardiovascular Surgery | 2268 | 2024/07 |
| 143 | Salman et al. | Hypothermic Ventricular Fibrillation in Redo Minimally Invasive Mitral Valve Surgery: A Promising Solution for a Surgical Challenge | DOI: 10.3390/jcm13144269 | Journal of Clinical Medicine | 888 | 2024/07 |
| 144 | Franz et al. | Minimally Invasive Surgery through Right Mini-Thoracotomy for Mitral Valve Infective Endocarditis: Contraindicated or Safely Possible? | DOI: 10.3390/jcm13144182 | Journal of Clinical Medicine | 937 | 2024/07 |
| 145 | Ali et al. | Minimally Invasive Mitral Valve Surgery Using a Cold Fibrillatory Cardiac Arrest Technique in Patients With Prior Cardiac Surgery | DOI: 10.14503/THIJ-23-8167 | Texas Heart Institue Journal | 34 | 2024/07 |
| 146 | Watanabe et al. | Possible role of QRS duration in the right ventricle as a perioperative monitoring parameter for right ventricular function: a prospective cohort analysis in robotic mitral valve surgery | DOI: 10.3389/fcvm.2024.1418251 | Frontiers in Cardiovascular medicine | 94 | 2024/07 |
| 147 | Veith et al. | Different approach, similar outcomes: the impact of surgical access routes in minimally invasive cardiac surgery on enhanced recovery after surgery | DOI: 10.3389/fcvm.2024.1412829 | Frontiers in Cardiovascular medicine | 170 | 2024/07 |
| 148 | Lio et al. | Robotic Mitral Valve Repair: Impact of Experience on Results and Complex Mitral Disease Treatment | DOI: 10.3390/jcm13133744 | Journal of Clinical Medicine | 144 | 2024/06 |
| 149 | Albano et al. | Early Postoperative Complications of Robotic-assisted Versus Minimally Invasive Mitral Valve Surgery: A Propensity Score-matched Analysis | DOI: 10.1053/j.jvca.2024.05.026 | Journal of Cardiothoracic and Vascular Anaesthesia | 375 | 2024/09 |
| 150 | Dinh et al. | Concomitant tricuspid valve regurgitation repair in patients with minimally invasive mitral valve surgery: a single-centre experience in Vietnam | DOI: 10.1097/MS9.0000000000002027 | Annals of medicine and surgery | 67 | 2024/04 |
| 151 | Babliak et al. | New approach to the mitral valve through the left anterior minithoracotomy for combined valve and coronary surgical procedures | DOI: 10.1016/j.xjtc.2023.11.015 | JTCVS | 24 | 2023/12 |
| 152 | Winter et al. | Subcutaneous emphysema in patients undergoing robotic cardiac surgery: risk factors and clinical outcome | DOI: 10.1007/s11701-024-02112-4 | Journal of Robotic Surgery | 116 | 2024/11 |
| 153 | Barac et al. | Robotic versus port-access mitral repair: A propensity score analysis | DOI: 10.1111/jocs.15342 | Journal of Cardiac Surgery | 377 | 2021/04 |
| 154 | Mihaljevic et al. | Robotic repair of posterior mitral valve prolapse versus conventional approaches: potential realized | DOI: 10.1016/j.jtcvs.2010.09.008 | Journal of thoracic and cardiovascular surgery | 375 | 2011/01 |
| 155 | Rao et al. | Robotic and endoscopic mitral valve repair for degenerative disease | DOI: 10.21037/acs-2022-rmvs-28 | Annals of Cardiothoracic Surgery | 124 | 2022/11 |
| 156 | Rufa et al. | A propensity matched comparison of robotic vs.traditional minimal access approach for mitral valve repair with concomitant cryoablation | DOI: 10.21037/jtd-23-1306 | Journal of Thoracic disease | 104 | 2023/12 |
| 157 | Wei et al. | Comparison of clinical outcomes between robotic and thoracoscopic mitral valve repair | DOI: 10.21037/cdt-20-197 | Cardiovascular diagnosis and therapy | 234 | 2020/10 |
| 158 | Mori et al. | Robotic Mitral Valve Repair for Degenerative Mitral Regurgitation | DOI: 10.1016/j.athoracsur.2023.07.047 | Annals of Thoracic Surgery | 23417 | 2024/01 |
| 159 | Chitwood et al. | Robotic mitral valve repairs in 300 patients: a single-center experience | DOI: 10.1016/j.jtcvs.2008.03.053 | Journal of thoracic and cardiovascular surgery | 309 | 2008/08 |
| 160 | Larsson et al. | Biomarkers of inflammation and coagulation after minimally invasive mitral valve surgery: a prospective comparison to conventional surgery | DOI: 10.1080/14017431.2024.2347293 | Scandinavian Cardiovascular Journal | 35 | 2024/06 |
| 161 | Ahmad et al. | First experiences with automated annular suturing device in totally endoscopic aortic and mitral valve replacement | DOI: 10.1093/icvts/ivae112 | Interdisciplinary Cardiovascular and thoracic surgery | 66 | 2024/06 |
| 162 | Sawa et al. | Effect of Narrow Chest on Minimally Invasive Mitral Valve Surgery via Right Minithoracotomy | DOI: 10.1253/circj.CJ-24-0142 | Circulation Journal | 206 | 2024/05 |
| 163 | Kang et al. | Comparison of modified Del Nido and Custodiol® cardioplegia in minimally invasive mitral valve surgery | DOI: 10.1093/ejcts/ezae161 | European Journal of Cardio-Thoracic Surgery | 312 | 2024/03 |
| 164 | Silaschi et al. | Transcatheter Edge-to-Edge Mitral Valve Repair versus Minimally Invasive Mitral Valve Surgery: An Observational Study | DOI: 10.3390/jcm13051372 | Journal of Clinical Medicine | 49 | 2024/02 |
| 165 | Murphy et al. | Endoscopic robotic mitral valve surgery | DOI: 10.1016/j.jtcvs.2006.04.052 | Journal of thoracic and cardiovascular surgery | 127 | 2006/10 |
| 166 | Marchetto et al. | Results of Cryoablation for Atrial Fibrillation Concomitant With Video-Assisted Minimally Invasive Mitral Valve Surgery | DOI: 10.1053/j.semtcvs.2016.04.006 | Seminars in throracic and cardiovascular surgery | 68 | 2016/04 |
| 167 | McClure et al. | One thousand minimally invasive mitral valve operations: early outcomes, late outcomes, and echocardiographic follow-up | DOI: 10.1016/j.jtcvs.2012.12.070 | Journal of thoracic and cardiovascular surgery | 1000 | 2013/05 |
| 168 | Barbero et al. | Aortic cannulation system for minimally invasive mitral valve surgery | DOI: 10.1016/j.jtcvs.2015.02.040 | Journal of thoracic and cardiovascular surgery | 65 | 2015/06 |
| 169 | Baccanelli et al. | Risk Factors for Higher Postoperative Myocardial Injury in Minimally Invasive Mitral Valve Surgery Patients: A Cohort Study | DOI: 10.3390/jcm13061591 | Journal of Clinical Medicine | 316 | 2024/03 |
| 170 | Malvindi et al. | On-table extubation is associated with reduced intensive care unit stay and hospitalization after trans-axillary minimally invasive mitral valve surgery | DOI: 10.1093/ejcts/ezae010 | European Journal of Cardio-Thoracic Surgery | 638 | 2024/03 |
| 171 | Cocchieri et al. | Elderly patients benefit from minimally invasive mitral valve surgery: perioperative risk management matters | DOI: 10.1093/icvts/ivad211 | Interdisciplinary Cardiovascular and thoracic surgery | 131 | 2024/01 |
| 172 | Dorsey et al. | Subvalvular techniques enhanced with endoscopic robotic mitral valve repair | DOI: 10.1016/j.xjtc.2023.08.019 | Journal of thoracic and cardiovascular surgery | 1024 | 2024/09 |
| 173 | Moscarelli et al. | The Effect of Minimally Invasive Surgery and Sternotomy on Physical Activity and Quality of Life | DOI: 10.1016/j.hlc.2020.09.936 | Heart, Lung & Circulation | 50 | 2021/06 |
| 174 | Zwischenberger et al. | Late Survival After Redo Mitral Operation With Minithoracotomy Compared With Sternotomy | DOI: 10.1016/j.athoracsur.2023.08.017 | Annals of Thoracic Surgery | 503 | 2024/02 |
| 175 | Burfeind et al. | Mitral surgery after prior cardiac operation: port-access versus sternotomy or thoracotomy | DOI: 10.1016/s0003-4975(02)03909-7 | Annals of Thoracic Surgery | 60 | 2002/10 |
| 176 | Woo et al. | Robotic minimally invasive mitral valve reconstruction yields less blood product transfusion and shorter length of stay | DOI: 10.1016/j.surg.2006.05.003 | Surgery | 25 | 2006/08 |
| 177 | Cresce et al. | Neurological outcomes in minimally invasive mitral valve surgery: risk factors analysis from the Mini-Mitral International Registry | DOI: 10.1093/ejcts/ezad336 | European Journal of Cardio-Thoracic Surgery | 7343 | 2023/10 |
| 178 | Yang et al. | Minimally invasive approach associated with lower resource utilization after aortic and mitral valve surgery | DOI: 10.1016/j.xjon.2023.06.007 | Journal of thoracic and cardiovascular surgery | 368 | 2023/06 |
| 179 | Ling et al. | Minimally invasive valve surgery including patients of combined simultaneous surgery: a retrospective study | DOI: 10.1186/s13019-023-02361-8 | Journal of Cardiothoracic Surgery | 29 | 2023/09 |
| 180 | Mostafa et al. | Redefining Unilateral Pulmonary Edema after Mitral Valve Surgery on Chest X-ray Imaging Using the RALE Scoring System | DOI: 10.3390/jcm12186043 | Journal of Clinical Medicine | 434 | 2023/09 |
| 181 | Barbero et al. | Minimally Invasive Surgery: Standard of Care for Mitral Valve Endocarditis | doi: 10.3390/medicina59081435. | Medicina | 92 | 2023/08 |
| 182 | Zhang et al. | Comparison of Minimally Invasive Valve Surgery: Hemi-Sternotomy vs. Right Anterolateral Thoracotomy Incision | none | Alternative Therapes in Health and Medicine | 54 | 2023/11 |
| 183 | Ceresa et al. | Virtually Wall-Less versus Standard Thin-Wall Venous Cannula in the Minimally Invasive Mitral Valve Surgery: Single-Center Experience | DOI: 10.3390/medicina59071221 | Medicina | 65 | 2023/06 |
| 184 | Passos et al. | Midterm outcomes of minimally invasive mitral valve surgery in a heterogeneous valve pathology cohort: respect or resect? | DOI: 10.21037/jtd-22-1796 | Journal of Thoracic disease | 278 | 2023/06 |
| 185 | Zheng et al. | A Study for QOL and Surgical Incision Pain in Patients Undergoing Totally Thoracoscopic Combined Aortic and Mitral Valve Replacement Surgery | DOI: 10.1532/hsf.5411 | Heart Surgery Forum | 62 | 2023/05 |
| 186 | Passos et al. | Propensity Matched Outcomes of Minimally Invasive Mitral Surgery: Does a Heart-Team Approach Eliminate Female Gender as an Independent Risk Factor? | DOI: 10.3390/jpm13060949 | Journal of personalized medicine | 302 | 2023/06 |
| 187 | Kowalewski et al. | Tricuspid intervention for less-than-severe regurgitation simultaneously with minimally invasive mitral valve surgery in patients with atrial fibrillation | DOI: 10.33963/KP.a2023.0137 | Kardiologika polska | 1545 | 2023/06 |
| 188 | Ascaso et al. | Repair of mitral prolapse: comparison of thoracoscopic minimally invasive and conventional approaches | DOI: 10.1093/ejcts/ezad235 | European Journal of Cardio-Thoracic Surgery | 188 | 2023/08 |
| 189 | Ushioda et al. | Evaluating Short-Term Postoperative Outcomes in Minimally Invasive Mitral Valve Surgery for Patients with Rheumatic Disease | DOI: 10.1532/hsf.5391 | Heart Surgery Forum | 167 | 2023/04 |
| 190 | Vinzant et al. | Pectoral Fascial Plane Versus Paravertebral Blocks for Minimally Invasive Mitral Valve Surgery Analgesia | DOI: 10.1053/j.jvca.2023.02.012 | Journal of Cardiothoracic and Vascular Anaesthesia | 313 | 2023/02 |
| 191 | Glance et al. | Racial and Ethnic Disparities in Access to Minimally Invasive Mitral Valve Surgery | DOI: 10.1001/jamanetworkopen.2022.47968 | JAMA | 103753 | 2022(12 |
| 192 | Franke et al. | Robotically assisted mitral valve surgery-experience during the restart of a robotic program in Germany | DOI: 10.21037/acs-2022-rmvs-18 | Annals of Cardiothoracic Surgery | 182 | 2022/11 |
| 193 | Berretta et al. | Enhanced recovery after minimally invasive heart valve surgery: Early and midterm outcomes | DOI: 10.1016/j.ijcard.2022.11.016 | International journal of cardiology | 576 | 2023/01 |
| 194 | Petersen et al. | Antegrade axillary arterial perfusion in 3D endoscopic minimally-invasive mitral valve surgery | DOI: 10.3389/fcvm.2022.980074 | Frontiers in Cardiovascular medicine | 100 | 2022/09 |
| 195 | Olsthoorn et al. | Does concomitant tricuspid valve surgery increase the risks of minimally invasive mitral valve surgery? A multicentre comparison based on data from The Netherlands Heart Registration | DOI: 10.1111/jocs.17004 | Journal of Cardiac Surgery | 775 | 2022/12 |
| 196 | Hisatomi et al. | Minimally Invasive Valvular Surgery in the Elderly - Safety, Early Recovery, and Long-Term Outcomes | DOI: 10.1253/circj.CJ-22-0338 | Circulation Journal | 121 | 2022/10 |
| 197 | Barbero et al. | Single-Dose St. Thomas Versus Custodiol® Cardioplegia for Right Mini-thoracotomy Mitral Valve Surgery | DOI: 10.1007/s12265-022-10296-z | Journal of cardiovascular tranlsational research | 187 | 2023/02 |
| 198 | Moscarelli et al. | Retrograde Autologous Priming for Minimally Invasive Mitral Valve Surgery | DOI: 10.1053/j.jvca.2022.03.031 | Journal of Cardiothoracic and Vascular Anaesthesia | 500 | 2022/03 |
| 199 | Wang et al. | Retrograde arterial perfusion and its outcome in robotic mitral-valve surgery | DOI: 10.1016/j.asjsur.2021.10.013 | Asian Journal of Surgery | 117 | 2022/10 |
| 200 | Kanda et al. | Comparing Conscious Sedation With Regional Anesthesia Versus General Anesthesia in Minimally Invasive Mitral Valve Surgery With Right-Sided Minithoracotomy: A Retrospective Study | DOI: 10.1053/j.jvca.2021.07.005 | Journal of Cardiothoracic and Vascular Anaesthesia | 101 | 2022/02 |
| 201 | Cetinkaya et al. | Long-term results after mitral valve surgery using minimally invasive versus sternotomy approach: a propensity matched comparison of a large single-center series | DOI: 10.1186/s12872-021-02121-3 | BMC Cardiovascular Disorders | 422 | 2021/06 |
| 202 | Chen et al. | Totally endoscopic mitral valve surgery: early experience in 188 patients | DOI: 10.1186/s13019-021-01464-4 | Journal of Cardiothoracic Surgery | 188 | 2021/04 |
| 203 | Whiteley et al. | Patient-reported outcome measures after mitral valve repair: a comparison between minimally invasive and sternotomy | DOI: 10.1093/icvts/ivaa276 | Interactive CardioVascular and Thoracic Surgery | 60 | 2021/04 |
| 204 | Fujita et al. | Benefits of robotically-assisted surgery for complex mitral valve repair | DOI: 10.1093/icvts/ivaa271 | Interactive CardioVascular and Thoracic Surgery | 335 | 2021/04 |
| 205 | Cetinkaya et al. | Long-term results of endoclamping in patients undergoing minimally invasive mitral valve surgery where external aortic clamping cannot be used - a propensity matched analysis | DOI: 10.1186/s13019-020-01363-0 | Journal of Cardiothoracic Surgery | 822 | 2020/10 |
| 206 | Dokollari et al. | Learning curve predictors for minimally invasive mitral valve surgery; how far should the rabbit hole go? | DOI: 10.1111/jocs.14939 | Journal of Cardiac Surgery | 105 | 2020/11 |
| 207 | Nissen et al. | Less Invasive Mitral Surgery Versus Conventional Sternotomy Stratified by Mitral Pathology | DOI: 10.1016/j.athoracsur.2020.05.145 | Annals of Thoracic Surgery |  | 2021/03 |
| 208 | Paparella et al. | Current trends in mitral valve surgery: A multicenter national comparison between full-sternotomy and minimally-invasive approach | DOI: 10.1016/j.ijcard.2019.11.137 | International journal of cardiology | 2602 | 2020/05 |
| 209 | Williams et al. | Predictors of Stroke After Minimally Invasive Mitral Valve Surgery Without the Cross-Clamp | DOI: 10.1053/j.semtcvs.2019.09.001 | Seminars in throracic and cardiovascular surgery | 1247 | 2019/09 |
| 210 | Chen et al. | Minimally Invasive Video-assisted Mitral Valve Replacement with a Right Chest Small Incision in Patients Aged Over 65 Years | DOI: 10.21470/1678-9741-2018-0409 | Brazilian Journal of Cardiothoracic Surgery | 20 | 2019/08 |
| 211 | Lebon et al. | Myocardial Protection in Minimally Invasive Mitral Valve Surgery: Retrograde Cardioplegia Alone Using Endovascular Coronary Sinus Catheter Compared With Combined Antegrade and Retrograde Cardioplegia | DOI: 10.1053/j.jvca.2018.11.042 | Journal of Cardiothoracic and Vascular Anaesthesia | 215 | 2019/05 |
| 212 | Jiang et al. | Feasibility, safety, and short-term outcome of totally thoracoscopic mitral valve procedure | DOI: 10.1186/s13019-018-0819-1 | Journal of Cardiothoracic Surgery | 51 | 2018/12 |
| 213 | Qiu et al. | Does full sternotomy have more significant impact than the cardiopulmonary bypass time in patients of mitral valve surgery? | DOI: 10.1186/s13019-018-0719-4 | Journal of Cardiothoracic Surgery | 283 | 2018/04 |
| 214 | van der Merwe et al. | Endoscopic port access surgery for isolated atrioventricular valve endocarditis | DOI: 10.1093/icvts/ivy103 | Interactive CardioVascular and Thoracic Surgery | 66 | 2018/10 |
| 215 | Körber et al. | Acute kidney injury following percutaneous edge-to-edge vs. minimally invasive surgical mitral valve repair: incidence, predictors and prognostic value | DOI: 10.4244/EIJ-D-17-00131 | EuroIntervention | 182 | 2018/02 |
| 216 | Lebon et al. | Myocardial Protection in Mitral Valve Surgery: Comparison Between Minimally Invasive Approach and Standard Sternotomy | DOI: 10.1053/j.jvca.2017.08.040 | Journal of Cardiothoracic and Vascular Anaesthesia | 118 | 2018/04 |
| 217 | Murphy et al. | Transthoracic Aortic Clamp Technique for Port-Only Endoscopic Robotic Mitral Surgery | DOI: 10.1177/15569845231172774 | Innovations (Philadelphia) | 133 | 2023/05 |
| 218 | Yates et al. | Minimally Invasive Mitral Valve Surgery With Concomitant Cox Maze Procedure Is as Effective as a Median Sternotomy With Decreased Morbidity | DOI: 10.1177/15569845231209974 | Innovations (Philadelphia) | 144 | 2023/11 |
| 219 | Sazzad et al. | Novel Retractor–Camera System Facilitates Less Traumatic Minimally Invasive Procedures and Declutters the Operation Field | DOI: 10.1177/15569845241277487 | Innovations (Philadelphia) | 20 | 2024/09 |
| 220 | Franz et al. | Minimally Invasive Mitral Valve Surgery in the Elderly | DOI: 10.1055/s-0043-1762940 | Thoracic and Cardiovascular Surgery | 761 | 2023/03 |
| 221 | Sharma et al. | Right thoracotomy with central cannulation for valve surgery: 10 years of experience | DOI: 10.1186/s13019-024-02945-y | Journal of Cardiothoracic Surgery | 728 | 2024/10 |
| 222 | Kastengren et al. | Percutaneous Vascular Closure Device in Minimally Invasive Mitral Valve Surgery | DOI: 10.1016/j.athoracsur.2019.10.038 | Annals of thoracic surgery | 268 | 2020/07 |
| 223 | Gumus et al. | Multiple Valve Implantation Through a Minimally Invasive Approach: Comparison of Standard Median Sternotomy and Right Anterior Thoracotomy | DOI: 10.1016/j.hlc.2020.01.012 | Heart, Lung & Circulation | 20 | 2020/09 |
| 224 | Aydin et al. | Robotic Mitral Valve Surgey Combined with Left Atrial Reduction and Ablation Procedures | DOI: 10.21470/1678-9741-2018-0297 | Brazilian Journal of Cardiothoracic Surgery | 11 | 2019/06 |
| 225 | Grazioli et al. | Comparative myocardial protection of endoaortic balloon versus external clamp in minimally invasive mitral valve surgery | DOI: 10.2459/JCM.0000000000001404 | Journal of Cardiovascular Medicine | 180 | 2023/03 |

**Table 1.1:** Preoperative data; F= female, BMI= body mass index (kg/m^2^), BSA= body surface area (m^2^), AF= atrial fibrillation, MI= mitral insufficiency, MS= mitral stenosis, MR= mitral regurgitation, DV= dilated ventricle, MPG= mean pressure gradient (mmHg), MTV= mean transvalular velocity (m/s), MOA= mitral orifice area (cm^2^), veg= vegetation, thr= thrombus, Ab= abcess, fi=fibrosis, ciMR=chronic ischemic MR, CKD= chronic kidney disease, AH= arterial hypertension, psk= previous stroke, HCH= hypercholesterolemia, sh=s moking history,fh= familial history, dCM= dilated cardiomyopathy, ES= EuroSCORE (%), logES= logistic EuroSCORE (%), HB= haemoglobin (mg/dl), AAT= alanine aminotransferase (U/L), sAlb= serum albumin (g/l), Crea= Creatinine (mg/dl), CRP= C-reactive protein (mg/l), K= potassium (mmol/dl), tBili= total bilirubin (µmol/l), BD= Barlow‘s disease, CC= congenital mitral cleft, IE= infective endocarditis, RD= rheumatic disease, FD= fibroelastic deficiency, deg= degerative

|  | **N** | **age** | **F** | **BMI** | **BSA** | **NYHA mean** | **NYHA I** | **NYHA II** | **NYHA III** | **NYHA IV** | **AF** | **PAP (mmHg)** | **LVEF (%)** | **MI** | **MS** | **MR** | **MR (mean grade)** | **TR none** | **mild** | **moderate** | **severe** | **AR none** | **mild** | **moderate** | **severe** | **LA, mm** | **DV** | **MPG** | **MTV** |
| --- | --- | --- | --- | --- | --- | --- | --- | --- | --- | --- | --- | --- | --- | --- | --- | --- | --- | --- | --- | --- | --- | --- | --- | --- | --- | --- | --- | --- | --- |
| **1** | 491,00 | 62,40 | 186,00 | 26,00 | 1,90 |  |  |  | 182,00 |  | 165,00 | 111,00 | 59,30 |  |  |  |  | 304,00 | 148,00 | 36,00 | 3,00 | 441,00 | 47,00 | 3,00 | 0,00 |  |  |  |  |
| **3** | 38,00 | **82,00** | 16,00 | 24,30 |  |  |  |  | 20,00 |  |  |  | 60,00 |  |  |  |  |  |  |  |  |  |  |  |  |  |  |  |  |
| **4** | 365,00 | **61,00** | 132,00 | 25,00 |  |  |  |  | 96,00 |  | 64,00 |  |  |  |  |  |  |  |  |  |  |  |  |  |  |  |  |  |  |
| **5** | 150,00 | **48,15** | 57,00 |  |  |  |  | 37,00 | 86,00 | 27,00 | 16,00 |  | 64,91 |  |  |  |  |  |  |  |  |  |  |  |  | 43,90 |  |  |  |
| **7** | 96,00 | **49,70** | 39,00 |  |  |  |  |  | 23,00 |  | 15,00 |  | 65,60 | 74,00 | 6,00 | 74,00 |  |  |  |  |  |  |  |  |  | 44,20 |  |  |  |
| **8** | 7674,00 | **68,90** | 3633,00 |  | 1,81 |  |  |  |  |  | 1175,00 |  | 54,90 |  |  |  |  |  |  |  |  |  |  |  |  |  |  |  |  |
| **9** | 166,00 | **67,30** | 48,00 | 26,50 |  |  | 18,00 | 46,00 | 53,00 | 11,00 | 69,00 |  | 35,00 | 133,00 |  |  |  |  |  |  |  |  |  |  |  |  |  |  |  |
| **13** | 72,00 | **66,90** | 30,00 |  |  |  |  |  |  |  | 3,90 |  | 56,10 |  |  |  |  |  |  |  |  |  |  |  |  |  |  |  |  |
| **14.1** | 29,00 | **51,04** | 6,00 | 25,80 |  |  |  |  |  |  | 9,00 |  |  | 29,00 |  |  |  |  |  |  |  |  |  |  |  |  |  |  |  |
| **14.2** | 62,00 | **47,23** | 21,00 | 25,20 |  |  |  |  |  |  | 11,00 |  |  | 62,00 |  |  |  |  |  |  |  |  |  |  |  |  |  |  |  |
| **14.3** | 82,00 | **51,43** | 23,00 | 27,80 |  |  |  |  |  |  | 14,00 |  |  | 82,00 |  |  |  |  |  |  |  |  |  |  |  |  |  |  |  |
| **15.1** | 123,00 | **63,00** | 44,00 | 25,30 |  |  |  |  | 27,00 |  | 24,00 |  | 60,60 | 117,00 |  |  |  |  |  |  |  |  |  |  |  |  |  |  |  |
| **15.2** | 61,00 | **64,20** | 22,00 | 25,20 |  |  |  |  | 14,00 |  | 12,00 |  | 62,00 | 60,00 |  |  |  |  |  |  |  |  |  |  |  |  |  |  |  |
| **18.1** | 50,00 | **51,00** | 11,00 |  |  |  | 22,00 | 17,00 | 10,00 | 1,00 |  | 37,00 | 57,00 |  | 2,00 | 45,00 |  |  |  |  |  |  |  |  |  |  |  |  |  |
| **18.2** | 62,00 | **50,00** | 20,00 |  |  |  | 25,00 | 32,00 | 5,00 | 0,00 |  | 35,00 | 63,00 |  | 3,00 | 54,00 |  |  |  |  |  |  |  |  |  |  |  |  |  |
| **18.3** | 40,00 | **50,00** | 11,00 |  |  |  | 19,00 | 15,00 | 6,00 | 0,00 |  | 35,00 | 62,00 |  | 0,00 | 32,00 |  |  |  |  |  |  |  |  |  |  |  |  |  |
| **20** | 1194,00 | **55,10** | 334,00 | 24,80 |  |  |  |  |  |  | 212,00 |  | 63,90 |  |  |  |  |  |  |  |  |  |  |  |  |  |  |  |  |
| **21** | 129,00 | **63,90** | 47,00 |  |  |  |  |  | 32,00 |  | 27,00 |  | 66,90 | 117,00 |  |  |  |  |  |  |  |  |  |  |  |  |  |  |  |
| **26** | 100,00 | **49,20** | 56,00 |  |  |  |  | 26,00 | 45,00 | 29,00 |  |  | 65,60 |  | 2,00 | 90,00 |  |  |  |  |  |  |  |  |  | 44,00 |  | 1,00 |  |
| **29** | 151,00 | **63,40** | 83,00 | 27,60 |  | 2,10 | 26,00 | 77,00 | 49,00 | 0,00 | 70,00 |  | 57,50 |  |  | 111,00 |  |  |  |  |  |  |  |  |  |  |  |  |  |
| **30** | 241,00 | **61,20** | 109,00 |  |  |  |  |  | 143,00 |  |  |  |  |  |  |  |  |  |  |  |  |  |  |  |  |  |  |  |  |
| **31.1** | 71,00 | **55,00** | 17,00 |  |  |  | 10,00 | 33,00 | 26,00 | 2,00 | 17,00 |  | 63,00 | 71,00 |  |  | 3,00 |  |  |  |  |  |  |  |  | 47,00 |  |  |  |
| **31.2** | 31,00 | **64,00** | 14,00 |  |  |  | 3,00 | 10,00 | 17,00 | 1,00 | 17,00 |  | 59,00 | 27,00 |  |  | 2,90 |  |  |  |  |  |  |  |  | 47,70 |  |  |  |
| **35** | 10,00 | **75,00** | 3,00 | 29,40 |  | 3,00 |  |  |  |  | 3,00 |  | 60,00 | 10,00 |  |  |  |  |  |  |  |  |  |  |  |  |  |  |  |
| **38** | 40,00 | **55,00** | 15,00 |  | 1,68 |  |  |  | 9,00 |  | 7,00 |  | 59,70 |  |  | 37,00 |  |  |  |  |  |  |  |  |  | 47,40 |  |  |  |
| **40** | 91,00 | **77,00** | 42,00 | 24,40 |  |  |  |  |  |  | 52,00 | 50,00 | 57,60 |  |  |  |  |  |  |  |  |  |  |  |  |  |  |  |  |
| **41** | 109,00 | **58,50** | 0,00 | 25,00 | 2,02 |  | 34,00 | 54,00 | 21,00 | 0,00 | 31,00 |  | 60,00 | 83,00 | 7,00 |  |  |  |  |  |  |  |  |  |  |  |  |  |  |
| **42** | 57,00 | **56,20** | 57,00 | 22,50 | 2,10 |  | 13,00 | 23,00 | 19,00 | 2,00 |  |  | 61,00 |  |  |  |  |  |  |  |  |  |  |  |  |  |  |  |  |
| **43** | 25,00 | **72,00** | 7,00 | 29,00 |  |  | 5,00 |  | 20,00 |  |  |  | 60,00 |  |  |  |  |  |  |  |  |  |  |  |  |  |  |  |  |
| **44.1** | 21,00 | **62,33** | 3,00 |  |  |  |  |  |  |  |  |  | 57,90 |  |  |  |  |  |  |  |  |  |  |  |  |  |  |  |  |
| **44.2** | 16,00 | **61,37** | 5,00 |  |  |  |  |  |  |  |  |  | 54,62 |  |  |  |  |  |  |  |  |  |  |  |  |  |  |  |  |
| **45** | 101,00 | **58,00** | 31,00 |  |  |  |  |  | 8,00 |  | 8,00 |  | 65,00 | 101,00 |  | 101,00 |  |  |  |  |  |  |  |  |  | 44,00 |  | 3,10 |  |
| **46** | 150,00 | **60,60** | 41,00 |  | 1,97 |  | 32,00 | 75,00 | 39,00 | 1,00 | 44,00 |  | 57,50 |  |  |  |  |  |  |  |  |  |  |  |  |  |  |  |  |
| **47** | 58,00 | **61,90** | 18,00 | 25,20 | 1,90 |  | 0,00 | 30,00 | 25,00 | 3,00 | 13,00 |  |  |  | 3,00 |  |  |  |  |  |  | 12,00 | 4,00 | 0,00 | 1,00 |  |  |  |  |
| **48** | 42,00 | **56,29** | 18,00 | 25,64 |  |  |  |  | 23,00 |  | 8,00 |  | 35,00 |  |  |  |  |  |  |  |  |  |  |  |  |  |  |  |  |
| **51** | 28,00 | **71,46** | 16,00 | 27,13 |  |  | 18,00 |  | 10,00 |  | 5,00 | 41,40 | 54,72 | 8,00 | 10,00 |  |  |  |  | 7,00 |  |  | 3,00 |  |  |  |  | 4,10 |  |
| **53.1** | 309,00 | **63,00** | 99,00 | 26,20 |  |  | 50,00 | 121,00 | 130,00 | 0,00 | 83,00 | 34,40 | 56,80 |  |  |  |  |  |  |  |  |  |  |  |  |  |  |  |  |
| **53.2** | 122,00 | **58,00** | 22,00 | 25,50 |  |  | 30,00 | 38,00 | 53,00 | 0,00 | 21,00 | 33,20 | 58,30 |  |  |  |  |  |  |  |  |  |  |  |  |  |  |  |  |
| **53.3** | 88,00 | **65,00** | 26,00 | 26,30 |  |  | 10,00 | 38,00 | 38,00 | 0,00 | 18,00 | 34,00 | 58,40 |  |  |  |  |  |  |  |  |  |  |  |  |  |  |  |  |
| **55.1** | 28,00 | **49,89** | 12,00 | 22,10 |  |  |  | 26,00 | 2,00 |  | 7,00 | 39,39 | 63,89 |  |  |  |  |  |  |  |  |  |  |  |  |  |  |  |  |
| **55.2** | 29,00 | **48,04** | 11,00 | 22,40 |  |  |  | 26,00 | 3,00 |  | 5,00 | 34,31 | 62,28 |  |  |  |  |  |  |  |  |  |  |  |  |  |  |  |  |
| **56.1** | 31,00 | **68,30** | 16,00 | 24,70 |  |  | 25,00 | 6,00 |  |  |  |  | 35,00 |  |  |  |  |  |  |  |  |  |  |  |  |  |  |  |  |
| **56.2** | 31,00 | **60,70** | 18,00 | 24,90 |  |  | 27,00 | 4,00 |  |  |  |  | 35,00 |  |  |  |  |  |  |  |  |  |  |  |  |  |  |  |  |
| **57** | 76,00 | **60,00** | 24,00 | 27,00 |  |  | 4,00 | 44,00 | 27,00 | 1,00 |  |  | 57,00 | 76,00 |  |  |  |  |  |  |  |  |  |  |  |  |  |  |  |
| **63.1** | 85,00 | **63,00** | 32,00 |  | 1,83 |  |  |  | 11,00 |  | 14,00 | 37,00 | 64,50 |  |  |  |  |  |  |  |  |  |  |  |  |  |  |  |  |
| **63.2** | 247,00 | **57,00** | 61,00 |  | 1,89 |  |  |  | 29,00 |  | 44,00 | 37,00 | 62,00 |  |  |  |  |  |  |  |  |  |  |  |  |  |  |  |  |
| **65** | 266,00 | **66,60** | 79,00 |  |  |  |  |  |  |  | 61,00 |  | 59,00 | 255,00 | 11,00 |  |  |  |  |  |  |  |  |  |  |  |  |  |  |
| **66** | 6878,00 | **65,00** | 2950,00 |  |  |  |  |  | 3109,00 |  | 2366,00 |  | 35,00 |  | 481,00 | 5315,00 |  |  |  |  |  |  |  |  |  |  |  |  |  |
| **67** | 1493,00 | 65,00 | 490,00 | 25,60 |  |  | 265,00 | 546,00 | 501,00 | 181,00 | 367,00 |  | 43,70 |  |  |  |  |  |  |  |  |  |  |  |  |  |  |  |  |
| **68** | 143,00 | **65,40** | 86,00 |  |  |  | 7,00 | 58,00 | 78,00 |  | 55,00 |  | 35,00 |  |  |  |  |  |  |  |  |  |  |  |  |  |  |  |  |
| **71** | 209,00 | 65,00 | 88,00 | 25,70 |  |  |  |  |  |  |  |  |  |  |  |  |  |  |  |  |  |  |  |  |  |  |  |  |  |
| **72** | 725,00 | 64,00 | 312,00 |  |  |  |  |  |  |  |  |  | 35,00 |  |  |  |  |  |  |  |  |  |  |  |  |  |  |  |  |
| **73** | 85,00 | 70,00 | 28,00 |  |  |  |  |  |  |  |  |  | 55,00 |  |  |  |  |  |  |  |  |  |  |  |  |  |  |  |  |
| **74** | 261,00 | 64,00 | 82,00 | 25,40 |  |  |  |  |  |  |  |  |  |  |  |  |  |  |  |  |  |  |  |  |  |  |  |  |  |
| **75** | 69,00 | 58,30 | 19,00 | 27,20 |  |  | 36,00 | 20,00 | 12,00 | 1,00 | 7,00 |  | 63,00 |  |  | 69,00 |  | 1,00 | 23,00 | 10,00 | 1,00 | 32,00 | 27,00 | 10,00 | 1,00 |  |  |  |  |
| **77** | 74,00 | 61,40 | 32,00 |  |  |  |  |  |  |  | 26,00 |  | 60,00 | 72,00 | 6,00 |  |  |  |  | 13,00 |  |  |  |  |  |  |  |  |  |
| **78.1** | 100,00 | 60,00 | 32,00 | 26,00 |  |  |  |  | 34,00 |  | 30,00 |  |  |  |  | 59,00 |  |  |  |  |  |  |  |  |  |  |  |  |  |
| **78.2** | 145,00 | 63,00 | 42,00 | 27,00 |  |  |  |  | 62,00 |  | 26,00 |  |  |  |  | 104,00 |  |  |  |  |  |  |  |  |  |  |  |  |  |
| **79** | 67,00 | 74,00 | 42,00 | 24,80 |  |  |  |  | 43,30 |  | 67,20 |  | 55,00 | 15,00 |  |  |  |  |  |  |  |  |  |  |  | 85,10 |  |  |  |
| **80** | 274,00 | 74,00 | 144,00 |  | 1,80 |  |  |  | 142,00 | 9,00 | 128,00 | 14,00 | 60,00 |  |  |  |  |  |  |  |  |  |  |  |  |  |  |  |  |
| **82** | 387,00 | 56,00 | 135,00 | 22,60 | 1,66 |  | 34,00 | 50,00 | 15,00 | 2,00 | 56,00 |  | 67,50 |  |  |  |  |  |  |  |  |  |  |  |  |  |  |  |  |
| **83** | 97,00 | 35,00 | 62,00 |  |  |  | 52,00 | 39,00 | 6,00 |  | 7,00 | 30,00 | 62,90 |  |  |  |  |  |  |  |  |  |  |  |  |  |  |  |  |
| **86** | 292,00 | 48,70 |  |  | 1,68 |  | 12,00 | 16,00 | 1,00 | 0,00 | 8,00 |  | 67,80 |  |  |  |  |  |  |  |  |  |  |  |  |  |  |  |  |
| **87.1** | 36,00 | 66,00 | 10,00 | 26,00 |  |  |  |  |  |  |  |  |  |  |  |  |  |  |  |  |  |  |  |  |  |  |  |  |  |
| **87.2** | 36,00 | 65,00 | 11,00 | 25,00 |  |  |  |  |  |  |  |  |  |  |  |  |  |  |  |  |  |  |  |  |  |  |  |  |  |
| **93** | 90,00 | 49,70 | 28,00 | 22,00 |  |  |  | 23,00 | 52,00 | 15,00 | 20,00 | 60,00 | 65,60 |  | 1,00 | 76,00 |  |  |  | 12,00 |  |  |  |  |  |  |  |  |  |
| **98** | 225,00 | 47,40 | 82,00 | 23,10 |  |  |  |  |  |  |  |  | 65,50 |  |  |  |  |  |  |  |  |  |  |  |  | 45,70 |  |  |  |
| **99.1** | 1309,00 | 62,43 | 494,00 |  |  |  |  |  | 377,00 |  | 409,00 |  | 35,00 |  |  |  |  |  |  |  |  | 1071,00 |  | 238,00 |  |  |  |  |  |
| **99.2** | 6669,00 | 62,60 | 2891,00 |  |  |  |  |  | 1359,00 |  | 2028,00 |  | 35,00 |  |  |  |  |  |  |  |  | 5556,00 |  | 1113,00 |  |  |  |  |  |
| **101.1** | 49,00 | 62,40 | 21,00 | 24,00 |  |  |  |  |  |  | 11,00 | 37,00 |  |  |  |  |  |  |  | 9,00 |  |  |  |  |  |  |  |  |  |
| **101.2** | 167,00 | 66,50 | 81,00 | 24,00 |  |  |  |  |  |  | 48,00 | 40,00 |  |  |  |  |  |  |  | 61,00 |  |  |  |  |  |  |  |  |  |
| **102** | 187,00 | 66,00 | 80,00 | 25,20 |  |  |  |  |  |  |  |  | 55,00 |  | 11,00 | 77,00 |  |  |  |  |  |  |  |  |  |  |  |  |  |
| **103** | 227,00 | 62,00 | 74,00 | 25,30 |  |  |  |  |  |  |  |  | 35,00 |  |  |  |  |  |  |  |  |  |  |  |  |  |  |  |  |
| **104** | 96,00 | 47,43 | 33,50 |  | 1,84 |  |  |  |  |  |  |  | 59,98 | 86,00 | 22,00 |  |  |  |  |  |  |  |  |  |  | 49,13 |  |  |  |
| **105** | 647,00 | 62,00 | 213,00 |  |  |  | 140,00 | 210,00 | 251,00 | 46,00 |  |  | 35,00 |  |  |  |  |  |  |  |  |  |  |  |  |  |  |  |  |
| **107** | 98,00 | 58,60 | 30,00 |  |  |  | 33,00 |  | 65,00 | 0,00 | 10,00 |  |  |  |  |  |  |  |  |  |  |  |  |  |  |  |  |  |  |
| **108** | 72,00 | 52,00 | 42,00 | 27,50 |  |  |  | 23,00 | 49,00 |  | 14,00 |  | 52,30 |  |  |  |  |  |  |  |  |  |  |  |  | 55,10 |  |  |  |
| **110** | 308,00 | 62,00 | 202,00 | 24,90 | 1,86 |  |  |  |  |  | 69,00 |  | 61,40 |  |  |  |  |  |  | 69,00 |  |  |  |  |  |  |  |  |  |
| **111** | 454,00 | 64,00 | 186,00 | 25,50 | 1,87 |  |  |  | 195,00 | 130,00 | 259,00 |  | 60,00 |  |  | 592,00 |  |  | 134,00 |  |  |  |  |  |  |  |  |  |  |
| **112** | 160,00 | 66,70 | 90,00 | 25,90 |  |  |  |  | 105,00 |  | 53,00 |  | 56,10 |  |  |  |  |  |  |  |  |  |  |  |  |  |  |  |  |
| **113.1** | 56,00 | 61,00 | 17,00 | 26,00 |  |  |  |  | 16,00 |  |  |  | 35,00 |  |  |  |  |  |  |  |  |  |  |  |  |  |  |  |  |
| **113.2** | 52,00 | 60,00 | 8,00 | 25,90 |  |  |  |  | 9,00 |  |  |  | 35,00 |  |  |  |  |  |  |  |  |  |  |  |  |  |  |  |  |
| **115.1** | 484,00 | 60,79 | 395,00 | 25,03 | 1,89 |  | 118,00 | 205,00 | 156,00 | 5,00 | 354,00 |  |  |  | 34,00 | 987,00 |  | 327,00 | 25,00 | 645,00 | 3,00 |  |  |  |  |  |  |  |  |
| **115.2** | 347,00 | 60,35 | 177,00 | 24,93 | 1,90 |  | 71,00 | 160,00 | 113,00 | 3,00 | 159,00 |  |  |  | 15,00 | 477,00 |  | 132,00 | 25,00 | 325,00 | 2,00 |  |  |  |  |  |  |  |  |
| **115.3** | 221,00 | 59,27 | 117,00 | 24,82 | 1,90 |  | 37,00 | 106,00 | 76,00 | 2,00 | 112,00 |  |  |  | 8,00 | 343,00 |  | 99,00 | 25,00 | 221,00 | 2,00 |  |  |  |  |  |  |  |  |
| **116.1** | 119,00 | 72,00 | 53,00 | 27,00 |  |  | 4,00 | 49,00 | 54,00 | 12,00 |  |  | 35,00 |  |  |  |  | 85,00 | 11,00 | 22,00 | 1,00 |  |  |  |  |  |  |  |  |
| **116.2** | 119,00 | 70,00 | 62,00 | 26,00 |  |  | 4,00 | 42,00 | 52,00 | 11,00 |  |  | 35,00 |  |  |  |  | 22,00 | 22,00 | 34,00 | 41,00 |  |  |  |  |  |  |  |  |
| **117.1** | 122,00 | 60,52 | 22,58 |  |  |  |  |  |  |  |  | 45,07 | 57,07 | 50,00 | 38,00 |  |  | 6,00 | 40,00 | 66,00 | 10,00 |  |  |  |  |  |  |  |  |
| **117.2** | 51,00 | 57,25 | 22,46 |  |  |  |  |  |  |  |  | 44,84 | 56,02 | 19,00 | 20,00 |  |  | 3,00 | 15,00 | 28,00 | 5,00 |  |  |  |  |  |  |  |  |
| **118** | 745,00 | 62,90 | 327,00 | 25,40 |  |  |  |  |  |  | 272,00 |  |  |  | 30,00 | 695,00 |  |  |  |  |  |  |  |  |  |  |  |  |  |
| **119.1** | 647,00 | 61,70 | 247,00 | 25,88 |  |  |  |  |  |  |  |  | 35,00 | 604,00 | 10,00 |  |  |  |  |  |  |  |  |  |  |  |  |  |  |
| **119.2** | 256,00 | 55,20 | 138,00 | 24,43 |  |  |  |  |  |  |  |  | 35,00 | 216,00 | 9,00 |  |  |  |  |  |  |  |  |  |  |  |  |  |  |
| **121.1** | 80,00 | 55,20 | 28,00 | 24,50 |  |  |  |  |  |  |  |  | 62,20 |  |  |  |  |  |  |  |  |  |  |  |  |  |  |  |  |
| **121.2** | 37,00 | 61,80 | 11,00 | 24,20 |  |  |  |  |  |  |  |  | 62,80 |  |  |  |  |  |  |  |  |  |  |  |  |  |  |  |  |
| **122.1** | 55,00 | 62,40 | 21,00 |  |  |  |  |  |  | 18,00 | 2,00 | 30,00 | 59,00 |  |  | 51,00 |  |  |  |  |  |  |  | 10,00 |  |  |  |  |  |
| **122.2** | 55,00 | 63,20 | 20,00 |  |  |  |  |  |  | 16,00 | 4,00 | 30,00 | 61,00 |  |  | 52,00 |  |  |  |  |  |  |  | 12,00 |  |  |  |  |  |
| **123.1** | 71,00 | 65,00 | 21,00 |  |  |  |  |  |  |  |  |  | 63,00 |  | 8,00 |  |  |  | 6,00 | 7,00 | 58,00 |  |  |  |  |  |  |  |  |
| **123.2** | 42,00 | 62,00 | 17,00 |  |  |  |  |  |  |  |  |  | 62,00 |  | 0,00 |  |  |  | 3,00 | 3,00 | 36,00 |  |  |  |  |  |  |  |  |
| **127** | 92,00 | 59,10 | 37,00 | 26,80 |  |  |  |  | 39,00 |  | 22,00 | 35,60 | 55,90 |  |  |  |  |  | 61,00 | 16,00 | 9,00 |  | 21,00 |  |  | 46,70 |  |  | 1,00 |
| **128** | 113,00 | 54,70 | 62,00 | 25,20 | 1,77 |  | 20,00 | 82,00 | 11,00 |  | 38,00 |  | 51,40 |  |  | 41,00 |  |  |  |  |  |  |  |  |  |  |  |  |  |
| **129** | 153,00 | 63,00 | 63,00 |  |  |  | 19,00 | 67,00 | 64,00 | 3,00 | 112,00 | 46,50 | 60,20 |  | 6,00 | 147,00 |  |  |  |  |  |  |  |  |  |  |  |  |  |
| **130** | 246,00 | 53,00 | 62,00 |  |  |  |  |  |  |  |  |  | 60,00 |  |  | 244,00 |  |  |  |  |  |  |  |  |  |  |  |  |  |
| **131.1** | 119,00 |  | 52,00 |  | 1,60 | 1,60 |  |  |  |  | 31,00 | 40,30 | 61,30 |  | 16,00 | 103,00 |  |  |  | 47,00 |  |  |  |  |  |  |  |  |  |
| **131.2** | 119,00 |  | 60,00 |  |  |  |  |  |  |  | 28,00 | 43,50 | 60,50 |  | 8,00 | 111,00 |  |  |  | 43,00 |  |  |  |  |  |  |  |  |  |
| **132 .1** | 120,00 | 56,00 | 43,00 | 25,20 |  |  |  |  |  | 50,00 | 27,00 |  | 60,00 |  |  |  |  |  |  | 24,00 |  |  |  |  |  |  |  |  |  |
| **132 .2** | 1292,00 | 63,00 | 447,00 | 24,50 |  |  |  |  |  | 133,00 | 316,00 |  | 62,00 |  |  |  |  |  |  | 279,00 |  |  |  |  |  |  |  |  |  |
| **133** | 67,00 | 56,93 | 27,00 |  |  |  | 16,00 | 33,00 | 15,00 | 3,00 | 9,00 |  |  |  |  |  |  |  |  |  |  |  |  |  |  |  |  |  |  |
| **134.1** | 102,00 | 64,00 | 42,00 | 24,00 |  |  | 3,00 | 42,00 | 57,00 |  | 30,00 |  | 60,00 |  | 6,00 |  |  |  |  |  |  |  |  |  |  |  |  |  |  |
| **134.2** | 241,00 | 62,00 | 87,00 | 24,60 |  |  | 2,00 | 129,00 | 1088,00 | 2,00 | 46,00 |  | 63,00 |  | 6,00 |  |  |  |  |  |  |  |  |  |  |  |  |  |  |
| **135 .1** | 250,00 | 64,70 | 100,00 | 25,50 |  |  |  |  | 133,00 |  | 61,00 |  | 54,10 |  |  | 234,00 |  |  |  | 16,00 |  |  |  |  |  |  |  |  |  |
| **135.2** | 250,00 | 63,30 | 87,00 | 26,00 |  |  |  |  | 157,00 |  | 78,00 |  | 56,90 |  |  | 236,00 |  |  |  | 22,00 |  |  |  |  |  |  |  |  |  |
| **136.1** | 163,00 | 49,53 | 41,00 |  |  |  | 65,00 | 69,00 | 28,00 | 2,00 | 13,00 |  | 65,00 |  |  |  |  |  |  |  |  |  |  |  |  |  |  |  |  |
| **136.2** | 192,00 | 42,43 | 94,00 |  |  |  | 104,00 | 63,00 | 24,00 | 1,00 | 19,00 |  | 60,00 |  |  |  |  |  |  |  |  |  |  |  |  |  |  |  |  |
| **136.3** | 139,00 | 53,76 | 34,00 |  |  |  | 80,00 | 49,00 | 10,00 | 1,00 | 2,00 |  | 64,00 |  |  |  |  |  |  |  |  |  |  |  |  |  |  |  |  |
| **136.4** | 27,00 | 51,87 | 14,00 |  |  |  | 12,00 | 12,00 | 3,00 | 0,00 | 4,00 |  | 62,00 |  |  |  |  |  |  |  |  |  |  |  |  |  |  |  |  |
| **136.5** | 17,00 | 53,60 | 5,00 |  |  |  | 11,00 | 4,00 | 2,00 | 0,00 | 3,00 |  | 62,50 |  |  |  |  |  |  |  |  |  |  |  |  |  |  |  |  |
| **137** | 7,00 | 74,90 | 3,00 |  | 1,46 |  | 1,00 | 4,00 | 2,00 | 0,00 |  |  | 62,80 |  |  |  |  |  |  |  |  |  |  |  |  | 56,70 |  |  |  |
| **138** | 37,00 | 62,00 | 18,00 | 27,10 |  |  |  |  |  |  | 7,00 |  | 60,00 |  |  |  |  |  |  |  |  |  |  |  |  |  |  |  |  |
| **139.1** | 33,00 | 79,00 | 15,00 | 31,00 |  |  |  |  | 30,00 |  | 17,00 |  | 49,00 |  |  |  |  |  |  |  |  |  |  |  |  |  |  |  |  |
| **139.2** | 87,00 | 76,00 | 51,00 |  |  |  |  |  | 71,00 |  | 40,00 |  | 49,00 |  |  |  |  |  |  |  |  |  |  |  |  |  |  |  |  |
| **139.3** | 1263,00 | 61,00 | 446,00 |  |  |  |  |  | 494,00 |  | 391,00 |  | 49,00 |  |  |  |  |  |  |  |  |  |  |  |  |  |  |  |  |
| **139.4** | 3141,00 | 64,00 | 1051,00 |  |  |  |  |  | 1302,00 |  | 834,00 |  | 49,00 |  |  |  |  |  |  |  |  |  |  |  |  |  |  |  |  |
| **140** | 297,00 | 63,00 | 104,00 | 25,00 |  |  |  |  |  |  | 78,00 |  | 60,00 |  |  |  |  |  |  |  |  |  |  |  |  |  |  |  |  |
| **141** | 360,00 | 60,40 | 252,00 | 23,30 |  |  |  |  | 39,00 |  | 197,00 |  | 57,30 |  | 79,00 | 197,00 |  |  |  |  | 142,00 |  |  |  |  | 57,40 |  |  |  |
| **142.1** | 2017,00 | 60,50 | 748,00 |  | 1,65 |  |  |  |  |  |  |  |  |  |  |  |  |  |  |  |  |  |  |  |  |  |  |  |  |
| **142.2** | 251,00 | 69,60 | 139,00 |  | 1,53 |  |  |  |  |  |  |  |  |  |  |  |  |  |  |  |  |  |  |  |  |  |  |  |  |
| **143.1** | 48,00 | 70,00 | 17,00 |  |  |  | 0,00 | 14,00 | 27,00 | 2,00 | 29,00 |  | 56,00 |  | 3,00 | 43,00 |  |  |  |  |  |  |  |  |  |  |  |  |  |
| **143.2** | 840,00 | 67,00 | 381,00 |  |  |  | 36,00 | 326,00 | 343,00 | 38,00 | 376,00 |  | 60,00 |  | 50,00 | 723,00 |  |  |  |  |  |  |  |  |  |  |  |  |  |
| **144.1** | 75,00 | 60,00 | 26,00 |  |  |  |  |  |  |  | 19,00 |  | 60,00 |  | 0,00 | 4,00 |  |  |  |  |  |  |  |  |  |  |  |  |  |
| **144.2** | 862,00 | 68,00 | 400,00 |  |  |  |  |  |  |  | 405,00 |  | 60,00 |  | 57,00 | 809,00 |  |  |  |  |  |  |  |  |  |  |  |  |  |
| **145** | 34,00 | 64,00 | 18,00 |  |  | 2,20 |  |  |  |  |  |  | 51,60 |  | 11,00 | 23,00 |  |  |  |  |  |  |  |  |  |  |  |  |  |
| **146** | 94,00 | 63,00 | 34,00 | 22,00 |  |  |  |  | 5,00 |  | 18,00 |  | 63,00 |  |  |  |  |  |  |  |  |  |  |  |  |  |  |  |  |
| **147** | 170,00 | 63,00 | 60,00 | 25,00 |  |  |  |  |  |  | 58,00 |  | 60,00 |  |  |  |  |  |  |  |  |  |  |  |  |  |  |  |  |
| **148 .1** | 39,00 | 56,00 | 3,00 |  |  |  |  |  |  |  |  |  | 55,00 |  |  |  |  |  |  |  |  |  |  |  |  |  |  |  |  |
| **148.2** | 105,00 | 59,00 | 10,00 |  |  |  |  |  |  |  |  |  | 55,00 |  |  |  |  |  |  |  |  |  |  |  |  |  |  |  |  |
| **149.1** | 123,00 | 59,00 | 38,00 | 23,70 |  |  |  |  |  |  | 15,00 |  |  |  |  |  |  |  |  |  |  |  |  |  |  |  |  |  |  |
| **149.2** | 252,00 | 64,00 | 125,00 | 25,10 |  |  |  |  |  |  | 78,00 |  |  |  |  |  |  |  |  |  |  |  |  |  |  |  |  |  |  |
| **149.3** | 98,00 | 60,00 | 34,00 | 23,70 |  |  |  |  |  |  | 14,00 |  | 62,00 |  |  |  |  |  |  |  |  |  |  |  |  |  |  |  |  |
| **149.4** | 98,00 | 58,00 | 24,00 | 24,80 |  |  |  |  |  |  | 10,00 |  | 60,00 |  |  |  |  |  |  |  |  |  |  |  |  |  |  |  |  |
| **150** | 67,00 | 54,50 | 41,00 |  |  |  |  |  |  |  | 28,00 |  | 58,00 |  | 22,00 | 21,00 |  |  |  |  | 44,00 |  |  |  |  |  |  |  |  |
| **151** | 24,00 | 65,50 | 6,00 | 30,00 |  |  |  |  |  |  | 7,00 |  | 39,00 | 24,00 |  |  |  |  |  |  |  |  |  |  |  |  |  |  |  |
| **152 .1** | 63,00 | 60,00 | 17,00 | 29,00 | 2,17 |  | 18,00 | 21,00 | 13,00 | 11,00 |  |  | 58,00 |  |  |  |  |  |  |  |  |  |  |  |  |  |  |  |  |
| **152.2** | 31,00 | 65,50 | 4,00 | 27,00 | 2,03 |  | 7,00 | 13,00 | 8,00 | 3,00 |  |  | 58,00 |  |  |  |  |  |  |  |  |  |  |  |  |  |  |  |  |
| **152.3** | 22,00 | 64,00 | 7,00 | 26,00 | 1,90 |  | 8,00 | 6,00 | 8,00 | 0,00 |  |  | 58,00 |  |  |  |  |  |  |  |  |  |  |  |  |  |  |  |  |
| **153.1** | 249,00 | 59,00 | 64,00 |  |  |  |  |  |  |  | 44,00 |  | 49,00 |  |  |  |  |  |  |  | 33,00 |  |  |  |  |  |  |  |  |
| **153.2** | 128,00 | 59,00 | 30,00 |  |  |  |  |  |  |  | 25,00 |  | 49,00 |  |  |  |  |  |  |  | 18,00 |  |  |  |  |  |  |  |  |
| **155.1** | 62,00 | 56,70 | 20,00 |  |  |  |  |  | 5,00 |  |  |  | 57,96 |  |  |  |  |  |  |  |  |  |  |  |  |  |  |  |  |
| **155.2** | 62,00 | 55,50 | 22,00 |  |  |  |  |  | 7,00 |  |  |  | 57,79 |  |  |  |  |  |  |  |  |  |  |  |  |  |  |  |  |
| **156.1** | 52,00 | 68,00 | 19,00 | 26,30 | 2,01 |  | 11,00 | 28,00 | 11,00 | 2,00 | 52,00 |  | 45,00 | 52,00 |  | 3,00 |  |  | 19,00 | 8,00 | 6,00 |  |  | 1,00 |  |  |  |  |  |
| **156.2** | 52,00 | 69,00 | 19,00 | 24,85 | 1,96 |  | 17,00 | 21,00 | 12,00 | 2,00 | 52,00 |  | 45,00 | 52,00 |  | 0,00 |  |  | 14,00 | 14,00 | 9,00 |  |  | 2,00 |  |  |  |  |  |
| **157.1** | 121,00 | 45,60 | 32,00 |  |  |  |  |  | 42,00 |  | 12,00 |  | 67,10 |  | 5,00 |  |  |  |  |  |  |  |  |  |  | 44,20 |  |  |  |
| **157.2** | 113,00 | 47,70 | 40,00 |  |  |  |  |  | 34,00 |  | 11,00 |  | 65,10 |  | 4,00 |  |  |  |  |  |  |  |  |  |  | 45,20 |  |  |  |
| **158.1** | 7692,00 | 61,10 | 2423,00 | 26,39 |  |  |  |  |  |  | 1678,00 |  | 60,00 |  |  | 7252,00 |  |  |  | 860,00 | 199,00 |  |  |  |  |  |  |  |  |
| **158.2** | 15725,00 | 62,50 | 5710,00 | 26,47 |  |  |  |  |  |  | 4077,00 |  | 59,00 |  |  | 14678,00 |  |  |  | 2018,00 | 539,00 |  |  |  |  |  |  |  |  |
| **160** | 35,00 | 56,00 | 3,00 | 23,98 |  |  |  |  | 6,00 |  | 2,00 |  | 45,00 |  |  |  |  |  |  |  |  |  |  |  |  |  |  |  |  |
| **161** | 66,00 | 61,80 | 23,00 | 26,70 |  |  | 4,00 | 8,00 | 54,00 |  |  |  |  | 16,00 | 3,00 |  |  |  |  |  |  |  | 35,00 |  |  |  |  |  |  |
| **162.1** | 53,00 | 61,30 | 23,00 |  | 1,55 |  |  |  |  |  | 13,00 |  | 67,40 |  |  |  |  |  |  |  |  |  |  |  |  | 42,10 |  |  |  |
| **162 .2** | 153,00 | 68,70 | 67,00 |  | 1,55 |  |  |  |  |  | 36,00 |  | 64,10 |  |  |  |  |  |  |  |  |  |  |  |  | 46,20 |  |  |  |
| **163.1** | 156,00 | 57,00 | 46,00 | 25,60 |  |  | 69,00 | 57,00 | 28,00 | 2,00 | 13,00 |  |  |  |  |  |  |  |  |  |  |  |  |  |  |  |  |  |  |
| **163.2** | 156,00 | 57,00 | 46,00 | 25,90 |  |  | 88,00 | 51,00 | 16,00 | 1,00 | 13,00 |  |  |  |  |  |  |  |  |  |  |  |  |  |  |  |  |  |  |
| **164** | 49,00 | 70,00 | 25,00 | 26,70 |  |  |  |  |  |  | 20,00 | 45,60 | 60,50 |  |  |  |  | 29,00 | 9,00 | 2,00 | 0,00 |  |  |  |  |  |  |  |  |
| **169.1** | 48,00 | 65,00 | 22,00 | 23,90 |  |  |  |  | 33,00 |  | 11,00 |  | 60,00 |  | 3,00 |  |  |  |  |  |  |  |  |  |  |  |  |  |  |
| **169.2** | 268,00 | 63,00 | 96,00 | 24,60 |  |  |  |  | 128,00 |  | 49,00 |  | 62,00 |  | 7,00 |  |  |  |  |  |  |  |  |  |  |  |  |  |  |
| **170.1** | 282,00 | 63,00 | 106,00 | 24,90 |  |  |  |  | 88,00 |  | 70,00 | 30,00 | 61,00 |  |  | 269,00 |  |  |  |  | 68,00 |  |  |  |  |  |  |  |  |
| **170.2** | 74,00 | 64,00 | 34,00 | 24,70 |  |  |  |  | 26,00 |  | 21,00 | 30,00 | 60,00 |  |  | 68,00 |  |  |  |  | 15,00 |  |  |  |  |  |  |  |  |
| **170.3** | 160,00 | 61,00 | 55,00 | 24,90 |  |  |  |  | 52,00 |  | 35,00 | 30,00 | 62,00 |  |  | 154,00 |  |  |  |  | 39,00 |  |  |  |  |  |  |  |  |
| **170.4** | 122,00 | 64,00 | 51,00 | 24,80 |  |  |  |  | 36,00 |  | 35,00 | 30,00 | 60,00 |  |  | 115,00 |  |  |  |  | 29,00 |  |  |  |  |  |  |  |  |
| **171.1** | 85,00 | 79,00 | 57,00 | 24,70 |  |  | 3,00 | 23,00 | 59,00 | 46,00 | 38,00 |  | 25,00 |  |  |  |  |  |  |  |  |  |  |  |  |  |  |  |  |
| **171.2** | 46,00 | 78,90 | 22,00 | 25,80 |  |  | 2,00 | 16,00 | 39,00 | 28,00 | 20,00 |  | 30,00 |  |  |  |  |  |  |  |  |  |  |  |  |  |  |  |  |
| **172.1** | 512,00 | 60,26 |  | 26,00 |  |  |  |  |  |  |  |  | 60,70 |  |  |  |  |  |  |  |  |  |  |  |  |  |  |  |  |
| **172.2** | 512,00 | 62,62 |  | 25,70 |  |  |  |  |  |  |  |  | 61,10 |  |  |  |  |  |  |  |  |  |  |  |  |  |  |  |  |
| **173** | 50,00 | 73,00 | 28,00 | 27,70 |  |  |  | 20,00 | 30,00 |  |  |  |  |  |  | 10,00 |  |  |  |  |  |  | 40,00 |  |  |  |  |  |  |
| **174** | 503,00 | 63,00 | 263,00 |  |  |  |  |  |  |  | 247,00 |  | 49,00 |  |  |  |  |  |  |  | 160,00 |  |  |  |  |  |  |  |  |
| **177** | 7343,00 | 65,00 | 3104,00 |  |  |  |  |  | 3337,00 |  | 2520,00 |  | 50,00 |  | 511,00 | 6836,00 |  |  |  |  |  |  |  |  |  |  |  |  |  |
| **177.2** | 1750,00 | 65,00 | 773,00 |  |  |  |  | 820,00 |  |  | 707,00 |  | 49,00 |  | 216,00 | 1597,00 |  |  |  |  |  |  |  |  |  |  |  |  |  |
| **177.3** | 3876,00 | 64,00 | 1548,00 |  |  |  |  | 1618,00 |  |  | 1420,00 |  | 49,00 |  | 195,00 | 3631,00 |  |  |  |  |  |  |  |  |  |  |  |  |  |
| **178** | 368,00 | 66,00 | 165,00 | 27,68 |  |  |  |  | 58,00 |  | 77,00 |  | 60,00 |  |  |  |  |  |  |  |  |  |  |  |  |  |  |  |  |
| **179** | 29,00 | 58,41 | 13,00 | 23,22 |  |  |  | 7,00 | 22,00 |  |  |  | 64,00 |  |  |  |  |  |  |  |  |  |  |  |  | 45,46 |  |  |  |
| **180** | 434,00 | 68,50 | 100,00 | 25,00 |  |  |  |  |  |  |  |  |  |  |  |  |  |  |  |  |  |  |  |  |  |  |  |  |  |
| **181** | 92,00 | 61,10 | 34,00 | 24,60 |  |  |  |  | 20,00 |  | 21,00 | 38,00 | 59,00 |  |  |  |  |  |  |  |  |  |  |  |  |  |  |  |  |
| **182** | 54,00 | 56,77 | 23,00 |  | 1,62 |  | 25,00 |  | 29,00 |  |  |  |  |  |  |  |  |  |  |  |  |  |  |  |  |  |  |  |  |
| **183.1** | 42,00 | 59,16 | 24,00 |  | 1,84 |  |  |  |  |  |  |  | 60,50 |  |  |  |  |  |  |  |  |  |  |  |  |  |  |  |  |
| **183.2** | 23,00 | 60,50 | 10,00 |  | 1,65 |  |  |  |  |  |  |  | 60,50 |  |  |  |  |  |  |  |  |  |  |  |  |  |  |  |  |
| **184.1** | 82,00 | 60,90 | 19,00 |  |  |  |  |  | 15,00 |  | 9,00 |  | 64,10 |  |  | 82,00 |  |  |  | 5,00 | 2,00 |  |  |  |  |  |  |  |  |
| **184.2** | 66,00 | 63,50 | 23,00 |  |  |  |  |  | 6,00 |  | 6,00 |  | 63,10 |  |  | 66,00 |  |  |  | 6,00 | 1,00 |  |  |  |  |  |  |  |  |
| **184.3** | 17,00 | 58,20 | 4,00 |  |  |  |  |  | 2,00 |  | 1,00 |  | 64,20 |  |  | 17,00 |  |  |  | 0,00 | 0,00 |  |  |  |  |  |  |  |  |
| **185** | 62,00 | 52,10 | 23,00 | 24,30 |  |  |  |  |  |  |  |  | 63,90 |  |  |  |  |  |  |  |  |  |  |  |  |  |  |  |  |
| **186.1** | 177,00 | 62,60 | 0,00 |  |  |  |  |  | 28,00 |  | 25,00 |  |  |  | 2,00 | 175,00 |  |  |  | 10,00 | 16,00 |  |  |  |  |  |  |  |  |
| **186.2** | 125,00 | 66,80 | 125,00 |  |  |  |  |  | 37,00 |  | 27,00 |  |  |  | 6,00 | 119,00 |  |  |  | 4,00 | 13,00 |  |  |  |  |  |  |  |  |
| **187 .1** | 565,00 | 68,00 | 285,00 | 27,20 | 2,70 |  | 41,00 | 184,00 | 303,00 | 37,00 |  |  | 54,00 |  | 48,00 | 563,00 |  |  |  |  |  |  |  |  |  |  |  |  |  |
| **187.2** | 565,00 | 69,00 | 281,00 | 27,80 | 2,70 |  | 69,00 | 136,00 | 306,00 | 54,00 |  |  | 54,00 |  | 77,00 | 564,00 |  |  |  |  |  |  |  |  |  |  |  |  |  |
| **188** | 188,00 | 62,00 | 117,00 | 26,00 |  |  |  | 63,00 | 40,00 |  | 33,00 | 40,00 | 60,00 |  |  |  |  |  |  |  |  |  |  |  |  | 45,70 |  |  |  |
| **189.1** | 77,00 | 54,10 | 28,00 | 22,12 |  |  |  |  | 11,00 |  | 67,00 |  | 61,50 |  | 53,00 | 38,00 |  |  |  | 23,00 |  |  |  |  |  | 57,00 |  |  |  |
| **189 .2** | 90,00 | 52,20 | 63,00 | 22,71 |  |  |  |  | 13,00 |  | 27,00 |  | 66,80 |  | 0,00 | 90,00 |  |  |  | 8,00 |  |  |  |  |  | 50,00 |  |  |  |
| **190.1** | 123,00 | 58,70 | 36,00 | 25,80 |  |  |  |  |  |  |  |  |  |  |  |  |  |  |  |  |  |  |  |  |  |  |  |  |  |
| **190 .2** | 190,00 | 60,30 | 68,00 | 25,70 |  |  |  |  |  |  |  |  |  |  |  |  |  |  |  |  |  |  |  |  |  |  |  |  |  |
| **191.1** | 89013,00 |  | 39633,00 |  |  |  |  |  |  |  | 27240,00 |  | 20,00 |  |  |  |  |  |  |  |  |  | 12957,00 | 2675,00 | 120,00 |  |  |  |  |
| **191.2** | 10404,00 |  | 5996,00 |  |  |  |  |  |  |  | 357,00 |  | 20,00 |  |  |  |  |  |  |  |  |  | 1297,00 | 339,00 | 30,00 |  |  |  |  |
| **191.3** | 4336,00 |  | 2257,00 |  |  |  |  |  |  |  | 1316,00 |  | 20,00 |  |  |  |  |  |  |  |  |  | 720,00 | 154,00 | 11,00 |  |  |  |  |
| **192.1** | 96,00 |  |  |  |  |  |  |  |  |  |  |  |  |  |  |  |  |  |  |  |  |  |  |  |  |  |  |  |  |
| **192.2** | 86,00 | 86,00 |  |  |  |  |  |  |  |  |  |  |  |  |  |  |  |  |  |  |  |  |  |  |  |  |  |  |  |
| **193.1** | 209,00 | 69,30 | 97,00 | 26,10 |  |  |  |  | 70,00 |  | 29,00 |  | 62,20 |  | 2,00 | 49,00 |  |  |  |  |  |  | 36,00 |  |  |  |  |  |  |
| **193.2** | 367,00 | 71,00 | 167,00 | 26,70 |  |  |  |  | 133,00 |  | 60,00 |  | 61,20 |  | 5,00 | 101,00 |  |  |  |  |  |  | 57,00 |  |  |  |  |  |  |
| **194.1** | 50,00 | 74,20 | 21,00 | 26,30 |  |  |  |  |  |  | 27,00 |  | 49,00 |  |  |  |  |  |  |  |  |  |  |  |  |  |  |  |  |
| **194.2** | 50,00 | 73,90 | 21,00 | 24,40 |  |  |  |  |  |  | 13,00 |  | 55,40 |  |  |  |  |  |  |  |  |  |  |  |  |  |  |  |  |
| **195.1** | 689,00 | 64,00 | 408,00 |  |  |  |  |  |  |  |  | 25,00 | 55,00 |  |  |  |  |  |  |  |  |  |  |  |  |  |  |  |  |
| **195.2** | 86,00 | 73,00 | 52,00 |  |  |  |  |  |  |  |  | 25,00 | 55,00 |  |  | 116,00 |  |  |  |  |  |  |  | 5,00 |  |  |  |  |  |
| **196** | 121,00 | 75,80 | 66,00 | 21,90 |  |  |  |  |  |  | 57,00 |  | 63,20 |  |  |  |  |  |  |  |  |  |  |  |  |  |  |  |  |
| **197.1** | 39,00 | 68,60 | 24,00 | 25,20 |  |  |  |  |  |  |  |  | 58,50 |  |  |  |  |  |  |  |  |  |  |  |  |  |  |  |  |
| **197.2** | 148,00 | 61,40 | 51,00 | 24,40 |  |  |  |  |  |  |  |  | 62,90 |  |  |  |  |  |  |  |  |  |  |  |  |  |  |  |  |
| **198.1** | 235,00 | 63,30 | 112,00 |  | 1,82 |  |  |  |  |  |  |  | 53,00 |  |  | 235,00 |  |  |  |  |  |  |  |  |  |  |  |  |  |
| **198.2** | 265,00 | 63,10 | 135,00 |  | 1,83 |  |  |  |  |  |  |  | 53,30 |  |  | 265,00 |  |  |  |  |  |  |  |  |  |  |  |  |  |
| **199.1** | 117,00 | 56,10 | 56,00 | 24,30 | 1,68 |  |  |  |  |  |  |  | 66,50 |  |  |  |  |  |  |  |  |  |  |  |  |  |  |  |  |
| **199.2** | 47,00 | 57,40 | 18,19 | 23,20 |  |  |  |  |  |  |  |  | 65,10 |  |  |  |  |  |  |  |  |  |  |  |  |  |  |  |  |
| **199.3** | 20,00 | 52,00 | 6,00 | 20,30 |  |  |  |  |  |  |  |  | 69,20 |  |  |  |  |  |  |  |  |  |  |  |  |  |  |  |  |
| **199.4** | 26,00 | 56,70 | 17,00 | 24,50 |  |  |  |  |  |  |  |  | 65,20 |  |  |  |  |  |  |  |  |  |  |  |  |  |  |  |  |
| **199.5** | 24,00 | 56,20 | 20,00 | 29,70 |  |  |  |  |  |  |  |  | 68,30 |  |  |  |  |  |  |  |  |  |  |  |  |  |  |  |  |
| **200.1** | 14,00 | 81,60 | 10,00 | 20,80 | 1,39 |  |  |  |  |  |  |  |  |  |  |  |  |  |  |  |  |  |  |  |  |  |  |  |  |
| **200.2** | 87,00 | 61,90 | 34,00 | 22,30 | 1,60 |  |  |  |  |  |  |  |  |  |  |  |  |  |  |  |  |  |  |  |  |  |  |  |  |
| **201** | 422,00 | 63,70 | 45,30 | 25,80 |  |  |  |  | 85,10 |  | 35,30 |  | 60,00 |  | 22,00 | 418,00 |  |  |  |  |  |  |  |  |  | 56,00 |  |  |  |
| **202** | 188,00 | 54,32 | 103,00 | 23,49 |  | 2,00 |  |  |  |  |  |  | 57,79 |  | 72,00 | 78,00 |  |  |  |  |  |  |  |  |  |  |  |  |  |
| **203** | 60,00 | 66,00 | 11,00 | 26,70 |  |  |  |  | 21,00 |  |  | 60,00 | 49,00 |  |  |  |  |  |  |  |  |  |  |  |  |  |  |  |  |
| **204.1** | 169,00 | 61,00 | 63,00 |  | 1,65 |  |  |  | 9,00 |  |  |  | 62,00 |  |  |  |  |  |  |  |  |  |  |  |  | 47,00 |  |  |  |
| **204.2** | 166,00 | 55,00 | 47,00 |  | 1,70 |  |  |  | 9,00 |  |  |  | 62,00 |  |  |  |  |  |  |  |  |  |  |  |  | 47,00 |  |  |  |
| **205 .1** | 64,00 | 62,30 | 25,02 | 27,20 |  |  |  |  | 41,98 |  | 20,99 |  |  |  | 2,05 |  |  |  |  |  |  |  |  |  |  |  |  |  |  |
| **205.2** | 758,00 | 63,00 | 322,91 | 26,10 |  |  |  |  | 520,75 |  | 238,01 |  |  |  | 24,26 |  |  |  |  |  |  |  |  |  |  |  |  |  |  |
| **206** | 105,00 | 75,00 | 44,00 | 25,76 | 1,80 |  | 12,00 | 70,00 | 21,00 | 2,00 | 14,00 | 37,39 |  |  |  |  |  |  |  |  |  |  |  |  |  |  |  |  |  |
| **207** | 10238,00 | 62,80 | 5897,09 |  |  |  | 307,14 | 1617,60 | 1771,17 | 552,85 | 1975,93 | 41,30 | 59,00 |  |  |  |  |  |  |  |  |  |  |  |  |  |  |  |  |
| **208** | 2602,00 | 64,00 | 1235,00 | 30,00 |  |  |  |  |  |  | 565,00 |  | 30,00 |  |  |  |  |  |  |  |  |  |  |  |  |  |  |  |  |
| **209.1** | 39,00 | 53,00 | 17,00 |  |  |  |  |  |  |  |  |  | 55,00 |  |  |  |  |  |  |  |  |  |  |  |  |  |  |  |  |
| **209.2** | 109,00 | 62,00 | 84,00 |  |  |  |  |  |  |  |  |  | 55,00 |  |  |  |  |  |  |  |  |  |  |  |  |  |  |  |  |
| **209.3** | 160,00 | 70,00 | 53,00 |  |  |  |  |  |  |  |  |  | 40,00 |  |  |  |  |  |  |  |  |  |  |  |  |  |  |  |  |
| **209.4** | 753,00 | 64,00 | 318,00 |  |  |  |  |  |  |  |  |  | 55,00 |  |  |  |  |  |  |  |  |  |  |  |  |  |  |  |  |
| **209.5** | 186,00 | 68,00 | 113,00 |  |  |  |  |  |  |  |  |  | 55,00 |  |  |  |  |  |  |  |  |  |  |  |  |  |  |  |  |
| **210** | 20,00 | 68,30 |  | 19,10 |  |  | 3,00 | 8,00 | 9,00 | 0,00 | 12,00 | 66,50 | 51,20 |  | 18,00 | 15,00 |  |  |  | 13,00 |  |  |  |  |  | 71,20 |  |  |  |
| **211.1** | 97,00 | 60,90 | 57,00 | 26,00 |  |  |  |  | 36,00 |  |  | 47,50 | 59,60 |  |  |  |  |  |  |  |  |  |  |  |  |  |  |  |  |
| **211.2** | 118,00 | 55,70 | 71,00 | 25,20 |  |  |  |  | 32,00 |  |  | 42,40 | 61,20 |  |  |  |  |  |  |  |  |  |  |  |  |  |  |  |  |
| **212** | 51,00 | 49,00 | 38,00 | 23,30 |  | 2,70 |  |  |  |  |  | 25,00 | 58,90 |  |  | 17,00 |  |  |  |  |  |  |  |  |  |  |  |  |  |
| **213** | 283,00 | 46,70 | 108,00 | 24,50 |  | 2,10 |  |  | 43,00 |  | 85,00 |  | 61,60 |  |  |  |  |  |  |  |  |  |  |  |  |  |  |  |  |
| **214** | 66,00 | 65,50 | 25,00 | 27,60 |  |  |  |  |  |  | 4,00 |  | 49,00 |  |  |  |  |  |  | 3,00 |  |  |  |  |  |  |  |  |  |
| **215** | 182,00 | 62,60 | 71,00 | 25,50 |  |  |  |  |  |  | 66,00 |  | 30,00 |  |  | 49,00 |  |  |  |  |  |  |  |  |  |  |  |  |  |
| **216** | 118,00 | 55,70 | 47,00 | 25,00 |  |  | 11,00 | 70,00 | 29,00 | 3,00 |  | 42,40 | 61,20 |  |  |  |  |  |  |  |  |  |  |  |  |  |  |  |  |
| **217** | 133,00 | 65,50 | 45,00 | 30,00 |  |  |  |  | 55,00 |  |  | 40,00 | 45,00 |  | 2,00 | 125,00 |  |  |  |  |  |  |  |  |  |  |  |  |  |
| **218** | 144,00 | 64,70 | 75,00 | 26,70 |  |  |  |  | 65,00 |  | 76,00 |  | 58,70 |  |  |  |  |  |  |  |  |  |  |  |  | 49,00 |  |  |  |
| **219.1** | 10,00 | 56,30 |  |  |  |  |  |  |  |  | 5,00 | 42,83 | 60,00 |  | 1,00 |  |  |  |  | 4,00 |  |  |  |  |  |  |  |  |  |
| **219 .2** | 10,00 | 60,40 |  |  |  |  |  |  |  |  | 3,00 | 44,33 | 60,00 |  | 0,00 |  |  |  |  | 1,00 |  |  |  |  |  |  |  |  |  |
| **220.1** | 190,00 | 79,00 | 119,00 |  |  |  | 0,00 | 62,00 | 105,00 | 8,00 | 125,00 |  | 60,00 |  | 12,00 | 170,00 |  |  |  |  |  |  |  |  |  |  |  |  |  |
| **220.2** | 116,00 | 77,00 | 73,00 |  |  |  | 0,00 | 41,00 | 60,00 | 4,00 | 83,00 |  | 60,00 |  | 6,00 | 102,00 |  |  |  |  |  |  |  |  |  |  |  |  |  |
| **220.3** | 60,00 | 81,00 | 35,00 |  |  |  | 0,00 | 18,00 | 35,00 | 3,00 | 35,00 |  | 60,00 |  | 4,00 | 54,00 |  |  |  |  |  |  |  |  |  |  |  |  |  |
| **220.4** | 14,00 | 86,00 | 11,00 |  |  |  | 0,00 | 3,00 | 10,00 | 1,00 | 7,00 |  | 62,00 |  | 2,00 | 14,00 |  |  |  |  |  |  |  |  |  |  |  |  |  |
| **220.5** | 190,00 | 67,00 | 101,00 |  |  |  | 8,00 | 75,00 | 78,00 | 7,00 | 117,00 |  | 60,00 |  | 10,00 | 168,00 |  |  |  |  |  |  |  |  |  |  |  |  |  |
| **221** | 728,00 |  |  |  |  |  |  |  |  |  |  |  |  |  |  |  |  |  |  |  |  |  |  |  |  |  |  |  |  |
| **222.1** | 147,00 | 63,00 | 35,00 | 24,70 |  |  | 13,00 | 44,00 | 63,00 | 27,00 | 59,00 |  |  |  |  |  |  |  |  |  |  |  |  |  |  |  |  |  |  |
| **222.2** | 121,00 | 61,90 | 26,00 | 24,90 |  |  | 11,00 | 40,00 | 61,00 | 8,00 | 37,00 |  |  |  |  |  |  |  |  |  |  |  |  |  |  |  |  |  |  |
| **223** | 20,00 | 72,10 | 12,00 | 26,60 |  |  |  | 12,00 | 8,00 |  | 13,00 | 44,40 | 57,30 |  |  |  |  |  |  |  |  |  |  |  |  | 50,20 |  |  |  |
| **224** | 11,00 | 45,70 | 4,00 |  |  |  | 0,00 | 1,00 | 7,00 | 3,00 | 11,00 | 45,75 | 54,62 |  |  |  |  |  |  | 3,00 |  |  |  |  |  |  |  |  |  |
| **225.1** | 78,00 | 60,38 | 27,00 | 25,44 | 1,85 |  | 1,00 | 53,00 | 22,00 | 2,00 | 24,00 | 35,60 | 58,03 |  |  |  |  |  |  |  |  |  |  |  |  |  |  |  |  |
| **225.2** | 102,00 | 63,04 | 50,00 | 24,97 | 1,78 |  | 6,00 | 47,00 | 41,00 | 8,00 | 38,00 | 37,81 | 56,05 |  |  |  |  |  |  |  |  |  |  |  |  |  |  |  |  |
| **Ʃ** | 222947,00 |  | 96682,05 |  |  |  | 2287,14 | 8472,60 | 20580,30 | 1578,85 | 57034,34 |  |  | 2511,00 | 2313,30 | 50680,00 | 5,90 | 1008,00 | 580,00 | 4997,00 | 1504,00 | 7112,00 | 15244,00 | 4562,00 | 163,00 | 1410,29 | 0,00 | 8,20 | 1,00 |
| **mean** |  | 61,98 | 388,28 | 25,20 | 1,83 | 2,24 | 31,77 | 105,91 | 140,00 | 22,24 | 343,58 | 40,05 | 55,71 |  | 35,59 | 703,89 |  |  |  |  |  |  |  |  |  | 50,37 |  | 2,73 | 1,00 |
| **SD** |  | 7,63 | 2610,51 | 2,73 | 0,25 | 0,43 | 51,86 | 267,66 | 432,50 | 70,73 | 2154,05 | 13,61 | 9,96 |  | 90,42 | 2134,31 |  |  |  |  |  |  |  |  |  | 9,01 |  | 1,29 | 0,00 |
| **Missings** | 0.0 | 2.0 | 4.0 | 46.0 |  |  |  |  |  | 36.0 |  | 14.0 |  |  |  | 61.0 |  |  |  |  |  |  |  |  |  |  |  |  |  |

|  | **MOA** | | **LVDD, mm** | | **LVEDV** | | **Veg** | | **Thr** | | **Ab** | | **Fib** | | **ciMR** | | **CKD** | | **AH** | | **COPD** | | **DM** | | **CAD** | | **pstroke** | | **PH** | | **HCH** | | **sh** | | **fh** | | **dCM** | | **ES(%)** | | **logES(%)** | | | HB | **AT** | | **sAlb** | | | **Crea** | | **CRP** | | **K** | | **CK (µmol/ls)** | | **CKMB (ng/ml)** | | **ttBili** | | **FVC (%)** | | **FEV1 (%)** | | **BD** | | **CC** | | **IE** | | **RD** | | **FD** | | **deg** | |
| --- | --- | --- | --- | --- | --- | --- | --- | --- | --- | --- | --- | --- | --- | --- | --- | --- | --- | --- | --- | --- | --- | --- | --- | --- | --- | --- | --- | --- | --- | --- | --- | --- | --- | --- | --- | --- | --- | --- | --- | --- | --- | --- | --- | --- | --- | --- | --- | --- | --- | --- | --- | --- | --- | --- | --- | --- | --- | --- | --- | --- | --- | --- | --- | --- | --- | --- | --- | --- | --- | --- | --- | --- | --- | --- | --- | --- | --- |
| **1** |  | |  | |  | |  | |  | |  | |  | |  | | 66,00 | |  | |  | |  | | 85,00 | | 14,00 | |  | |  | |  | |  | |  | | 1,20 | |  | | | 13,30 |  | |  | | |  | |  | |  | |  | |  | |  | |  | |  | |  | |  | |  | |  | |  | |  | |
| **3** |  | |  | |  | |  | |  | |  | |  | |  | |  | | 26,00 | | 2,00 | | 3,00 | |  | |  | |  | | 15,00 | | 13,00 | |  | |  | | 3,10 | |  | | |  |  | |  | | | 1,11 | |  | |  | |  | |  | |  | |  | |  | |  | |  | |  | |  | |  | |  | |
| **4** |  | |  | |  | |  | |  | |  | |  | |  | | 8,00 | | 211,00 | | 10,00 | | 16,00 | |  | |  | |  | | 102,00 | | 30,00 | |  | |  | | 2,70 | |  | | |  |  | |  | | |  | |  | |  | |  | |  | |  | |  | |  | |  | |  | |  | |  | |  | |  | |
| **5** |  | | 49,11 | |  | |  | |  | |  | |  | |  | | 18,00 | | 46,00 | | 5,00 | | 13,00 | | 16,00 | |  | |  | |  | |  | |  | |  | | 2,56 | |  | | |  |  | |  | | |  | |  | |  | |  | |  | |  | |  | |  | | 13,00 | | 12,00 | | 12,00 | | 10,00 | | 101,00 | |  | |
| **7** |  | |  | |  | |  | |  | |  | |  | |  | | 4,00 | | 28,00 | | 6,00 | |  | | 13,00 | | 10,00 | |  | | 2661,00 | | 471,00 | |  | |  | | 3,70 | |  | | | 13,12 | 20,70 | | 41,50 | | | 0,93 | |  | |  | |  | |  | | 14,50 | |  | |  | |  | |  | | 7,00 | |  | |  | |  | |
| **8** |  | |  | |  | |  | |  | |  | |  | |  | |  | |  | | 502,00 | | 762,00 | |  | |  | |  | | 2661,00 | | 471,00 | |  | |  | | 3,70 | |  | | |  |  | |  | | |  | |  | |  | |  | |  | |  | |  | |  | |  | |  | |  | |  | |  | |  | |
| **9** |  | | 55,00 | |  | |  | |  | |  | |  | |  | | 6,00 | |  | | 14,00 | | 7,00 | |  | | 7,00 | | 30,00 | |  | |  | |  | |  | | 1,70 | |  | | |  |  | |  | | |  | |  | |  | |  | |  | |  | |  | |  | |  | |  | |  | |  | |  | |  | |
| **13** |  | |  | |  | |  | |  | |  | |  | |  | |  | | 49,00 | |  | | 10,00 | | 15,00 | |  | |  | |  | |  | |  | |  | |  | |  | | |  |  | |  | | |  | |  | |  | |  | |  | |  | |  | |  | |  | |  | |  | |  | |  | |  | |
| **14.1** |  | |  | |  | |  | |  | |  | |  | |  | |  | |  | |  | |  | |  | |  | |  | |  | |  | |  | |  | | 0,66 | |  | | |  |  | |  | | |  | |  | |  | |  | |  | |  | |  | |  | |  | |  | |  | |  | |  | |  | |
| **14.2** |  | |  | |  | |  | |  | |  | |  | |  | |  | |  | |  | |  | |  | |  | |  | |  | |  | |  | |  | | 0,67 | |  | | |  |  | |  | | |  | |  | |  | |  | |  | |  | |  | |  | |  | |  | |  | |  | |  | |  | |
| **14.3** |  | |  | |  | |  | |  | |  | |  | |  | |  | |  | |  | |  | |  | |  | |  | |  | |  | |  | |  | | 0,70 | |  | | |  |  | |  | | |  | |  | |  | |  | |  | |  | |  | |  | |  | |  | |  | |  | |  | |  | |
| **15.1** |  | |  | |  | |  | |  | |  | |  | |  | | 1,00 | | 78,00 | | 3,00 | | 3,00 | |  | |  | |  | | 24,00 | | 13,00 | | 1,00 | |  | | 1,20 | |  | | |  |  | |  | | | 0,92 | |  | |  | |  | |  | |  | |  | |  | | 15,00 | |  | | 3,00 | | 1,00 | |  | | 103,00 | |
| **15.2** |  | |  | |  | |  | |  | |  | |  | |  | | 1,00 | | 27,00 | | 3,00 | | 1,00 | |  | |  | |  | | 14,00 | | 6,00 | | 0,00 | |  | | 1,30 | |  | | |  |  | |  | | | 0,96 | |  | |  | |  | |  | |  | |  | |  | | 16,00 | |  | | 1,00 | | 0,00 | |  | | 51,00 | |
| **18.1** |  | | 56,00 | |  | |  | |  | |  | |  | |  | |  | | 16,00 | |  | | 2,00 | |  | |  | |  | |  | |  | |  | |  | |  | |  | | |  |  | |  | | |  | |  | |  | |  | |  | |  | |  | |  | |  | | 2,00 | |  | | 2,00 | |  | | 46,00 | |
| **18.2** |  | | 56,00 | |  | |  | |  | |  | |  | |  | |  | | 20,00 | |  | | 3,00 | |  | |  | |  | |  | |  | |  | |  | |  | |  | | |  |  | |  | | |  | |  | |  | |  | |  | |  | |  | |  | |  | | 0,00 | |  | | 4,00 | |  | | 58,00 | |
| **18.3** |  | | 54,00 | |  | |  | |  | |  | |  | |  | |  | | 12,00 | |  | | 2,00 | |  | |  | |  | |  | |  | |  | |  | |  | |  | | |  |  | |  | | |  | |  | |  | |  | |  | |  | |  | |  | |  | | 1,00 | |  | | 1,00 | |  | | 38,00 | |
| **20** |  | |  | |  | | 22,00 | | 1,00 | | 1,00 | | 41,00 | |  | | 1,00 | |  | | 28,00 | | 38,00 | |  | |  | |  | |  | |  | |  | |  | | 2,90 | |  | | |  |  | |  | | |  | |  | |  | |  | |  | |  | |  | |  | | 120,00 | |  | |  | |  | |  | |  | |
| **21** |  | | 54,70 | |  | |  | |  | |  | |  | |  | | 14,00 | | 66,00 | | 4,00 | | 9,00 | |  | |  | | 16,00 | |  | |  | |  | |  | | 2,10 | |  | | |  |  | |  | | |  | |  | |  | |  | |  | |  | |  | |  | | 3,00 | |  | | 12,00 | | 2,00 | |  | | 101,00 | |
| **26** |  | | 56,00 | |  | |  | |  | |  | |  | |  | |  | | 28,00 | | 6,00 | | 8,00 | | 13,00 | | 10,00 | |  | |  | |  | |  | |  | |  | |  | | |  |  | |  | | |  | |  | |  | |  | |  | |  | |  | |  | | 10,00 | | 10,00 | | 9,00 | | 2,00 | |  | | 78,00 | |
| **29** |  | |  | |  | |  | |  | |  | |  | |  | |  | | 109,00 | | 15,00 | | 17,00 | |  | |  | |  | | 70,00 | |  | |  | |  | | 2,20 | |  | | |  |  | |  | | | 0,96 | |  | |  | |  | |  | |  | |  | |  | |  | |  | |  | | 4,00 | |  | | 126,00 | |
| **30** |  | | 56,70 | | 127,40 | |  | |  | |  | |  | |  | | 12,00 | |  | |  | |  | |  | | 5,00 | | 200,00 | |  | |  | |  | |  | |  | |  | | |  |  | |  | | |  | |  | |  | |  | |  | |  | |  | |  | | 22,00 | | 1,00 | | 1,00 | | 3,00 | |  | | 223,00 | |
| **31.1** | 0,50 | | 57,80 | |  | |  | |  | |  | |  | |  | | 5,00 | | 46,00 | | 4,00 | | 3,00 | |  | | 3,00 | |  | |  | |  | |  | |  | | 1,60 | |  | | |  |  | |  | | |  | |  | |  | |  | |  | |  | |  | |  | |  | |  | |  | |  | |  | |  | |
| **31.2** | 0,50 | | 50,80 | |  | |  | |  | |  | |  | |  | | 4,00 | | 22,00 | | 3,00 | | 3,00 | |  | | 5,00 | |  | |  | |  | |  | |  | | 4,20 | |  | | |  |  | |  | | |  | |  | |  | |  | |  | |  | |  | |  | |  | |  | |  | |  | |  | |  | |
| **35** |  | |  | |  | |  | |  | |  | |  | |  | | 4,00 | | 9,00 | |  | | 3,00 | |  | |  | | 0,00 | | 7,00 | | 1,00 | |  | |  | | 8,10 | |  | | |  |  | |  | | |  | |  | |  | |  | |  | |  | |  | |  | |  | |  | |  | |  | |  | |  | |
| **38** |  | | 57,60 | |  | |  | |  | |  | |  | |  | | 3,00 | | 13,00 | | 0,00 | | 3,00 | |  | | 3,00 | |  | |  | |  | |  | |  | |  | |  | | |  |  | |  | | |  | |  | |  | |  | |  | |  | |  | |  | |  | |  | | 1,00 | | 0,00 | |  | | 39,00 | |
| **40** |  | |  | |  | |  | |  | |  | |  | |  | |  | | 56,00 | | 4,00 | | 10,00 | | 25,00 | | 6,00 | |  | |  | |  | |  | |  | |  | |  | | | 13,20 |  | |  | | | 1,00 | |  | |  | |  | |  | |  | |  | |  | |  | |  | |  | |  | |  | |  | |
| **41** |  | |  | |  | |  | |  | |  | |  | |  | | 8,00 | |  | | 10,00 | |  | | 3,00 | |  | |  | |  | |  | |  | |  | | 0,67 | |  | | |  |  | |  | | |  | |  | |  | |  | |  | |  | |  | |  | |  | |  | | 5,00 | |  | |  | | 93,00 | |
| **42** |  | |  | |  | |  | |  | |  | |  | |  | |  | | 20,00 | | 1,00 | | 2,00 | |  | |  | | 23,00 | | 9,00 | | 5,00 | |  | |  | | 2,00 | |  | | |  |  | |  | | |  | |  | |  | |  | |  | |  | |  | |  | |  | |  | | 2,00 | |  | |  | |  | |
| **43** |  | |  | |  | |  | |  | |  | |  | |  | |  | | 21,00 | | 1,00 | | 2,00 | |  | |  | | 17,00 | |  | |  | |  | |  | | 3,58 | |  | | |  |  | |  | | |  | |  | |  | |  | |  | |  | |  | |  | |  | |  | | 2,00 | |  | |  | |  | |
| **44.1** |  | |  | |  | |  | |  | |  | |  | |  | |  | |  | |  | |  | |  | |  | |  | |  | |  | |  | |  | | 4,03 | |  | | |  |  | |  | | |  | |  | |  | |  | |  | |  | |  | |  | |  | |  | |  | |  | |  | |  | |
| **44.2** |  | |  | |  | |  | |  | |  | |  | |  | |  | |  | |  | |  | |  | |  | |  | |  | |  | |  | |  | | 4,60 | |  | | |  |  | |  | | |  | |  | |  | |  | |  | |  | |  | |  | |  | |  | |  | |  | |  | |  | |
| **45** |  | | 53,00 | |  | |  | |  | |  | |  | |  | |  | | 37,00 | | 2,00 | | 3,00 | | 1,00 | |  | |  | | 35,00 | | 25,00 | |  | |  | |  | |  | | |  |  | |  | | | 0,91 | |  | |  | |  | |  | |  | |  | |  | |  | |  | |  | |  | |  | |  | |
| **46** |  | | 54,80 | |  | |  | |  | |  | |  | |  | |  | | 85,00 | | 6,00 | | 4,00 | | 26,00 | |  | |  | | 47,00 | |  | |  | |  | |  | |  | | |  |  | |  | | |  | |  | |  | |  | |  | |  | |  | |  | |  | |  | |  | |  | |  | |  | |
| **47** |  | |  | |  | |  | |  | |  | |  | |  | | 3,00 | |  | | 3,00 | | 2,00 | |  | |  | |  | |  | |  | |  | |  | | 1,78 | |  | | |  |  | |  | | |  | |  | |  | |  | |  | |  | |  | |  | |  | |  | | 2,00 | |  | |  | |  | |
| **48** |  | |  | |  | |  | |  | |  | |  | |  | |  | | 23,00 | |  | | 6,00 | | 7,00 | |  | |  | |  | |  | |  | |  | |  | |  | | |  |  | |  | | | 1,24 | |  | |  | |  | |  | |  | |  | |  | |  | |  | | 8,00 | |  | |  | |  | |
| **51** |  | |  | |  | |  | |  | |  | |  | |  | |  | | 15,00 | | 6,00 | | 9,00 | |  | | 1,00 | |  | | 16,00 | |  | |  | |  | |  | |  | | |  |  | |  | | | 0,95 | |  | |  | |  | |  | |  | |  | |  | |  | |  | |  | |  | |  | |  | |
| **53.1** |  | |  | |  | |  | |  | |  | |  | |  | | 32,00 | | 169,00 | | 28,00 | | 27,00 | |  | |  | |  | | 73,00 | |  | |  | |  | | 1,12 | |  | | |  |  | |  | | |  | |  | |  | |  | |  | |  | |  | |  | |  | |  | | 5,00 | |  | |  | | 42,00 | |
| **53.2** |  | |  | |  | |  | |  | |  | |  | |  | |  | | 61,00 | | 10,00 | | 8,00 | |  | | 0,00 | |  | | 25,00 | |  | |  | |  | | 0,98 | |  | | |  |  | |  | | |  | |  | |  | |  | |  | |  | |  | |  | |  | |  | |  | |  | |  | |  | |
| **53.3** |  | |  | |  | |  | |  | |  | |  | |  | |  | | 54,00 | | 6,00 | | 10,00 | |  | | 0,00 | |  | | 20,00 | |  | |  | |  | | 1,11 | |  | | |  |  | |  | | |  | |  | |  | |  | |  | |  | |  | |  | |  | |  | |  | |  | |  | |  | |
| **55.1** |  | |  | |  | |  | |  | |  | |  | |  | |  | | 2,00 | |  | | 2,00 | |  | |  | |  | |  | |  | |  | |  | |  | |  | | | 12,48 | 22,68 | |  | | | 0,81 | |  | |  | |  | |  | |  | |  | |  | |  | |  | |  | |  | |  | |  | |
| **55.2** |  | |  | |  | |  | |  | |  | |  | |  | |  | | 3,00 | |  | | 1,00 | |  | |  | |  | |  | |  | |  | |  | |  | |  | | | 12,62 | 20,50 | |  | | | 0,81 | |  | |  | |  | |  | |  | |  | |  | |  | |  | |  | |  | |  | |  | |
| **56.1** |  | |  | |  | |  | |  | |  | |  | |  | |  | | 8,00 | |  | |  | |  | | 1,00 | |  | | 4,00 | | 1,00 | | 6,00 | |  | |  | |  | | |  |  | |  | | | 0,89 | |  | |  | |  | |  | |  | |  | |  | |  | |  | |  | |  | |  | | 31,00 | |
| **56.2** |  | |  | |  | |  | |  | |  | |  | |  | |  | | 6,00 | |  | |  | |  | | 0,00 | |  | | 6,00 | | 1,00 | | 4,00 | |  | |  | |  | | |  |  | |  | | | 0,78 | |  | |  | |  | |  | |  | |  | |  | |  | |  | |  | |  | |  | | 30,00 | |
| **57** |  | | 59,20 | |  | |  | |  | |  | |  | |  | |  | | 41,00 | | 3,00 | | 2,00 | |  | |  | | 10,00 | |  | |  | |  | |  | | 1,23 | |  | | |  |  | |  | | |  | |  | |  | |  | |  | |  | |  | |  | |  | |  | |  | |  | |  | |  | |
| **63.1** |  | | 61,00 | |  | |  | |  | |  | |  | |  | |  | | 34,00 | |  | |  | | 9,00 | |  | |  | |  | |  | |  | |  | |  | |  | | |  |  | |  | | |  | |  | |  | |  | |  | |  | |  | |  | |  | |  | |  | |  | |  | |  | |
| **63.2** |  | | 63,00 | |  | |  | |  | |  | |  | |  | |  | | 64,00 | |  | |  | | 21,00 | |  | |  | |  | |  | |  | |  | |  | |  | | |  |  | |  | | |  | |  | |  | |  | |  | |  | |  | |  | |  | |  | |  | |  | |  | |  | |
| **65** |  | |  | |  | |  | |  | |  | |  | |  | |  | |  | | 12,00 | |  | |  | | 12,00 | |  | |  | |  | |  | |  | | 2,50 | |  | | |  |  | |  | | |  | |  | |  | |  | |  | |  | |  | |  | |  | |  | | 16,00 | |  | |  | |  | |
| **66** |  | |  | |  | |  | |  | |  | |  | |  | | 3914,00 | | 3618,00 | | 620,00 | | 612,00 | | 876,00 | |  | | 2620,00 | | 1985,00 | |  | |  | |  | | 1,30 | |  | | |  |  | |  | | |  | |  | |  | |  | |  | |  | |  | |  | |  | |  | | 290,00 | | 527,00 | |  | | 4362,00 | |
| **67** |  | |  | |  | |  | |  | |  | |  | |  | | 62,00 | | 748,00 | | 252,00 | | 140,00 | | 227,00 | | 131,00 | | 579,00 | | 462,00 | | 91,00 | |  | |  | |  | |  | | | 13,90 |  | |  | | | 0,99 | |  | |  | |  | |  | |  | |  | |  | |  | |  | | 8,00 | |  | |  | |  | |
| **68** |  | |  | |  | |  | |  | |  | |  | |  | | 2,00 | |  | |  | | 10,00 | |  | | 6,00 | |  | |  | |  | |  | |  | |  | |  | | |  |  | |  | | |  | | 1,30 | |  | |  | | 13,30 | |  | |  | |  | |  | |  | |  | |  | |  | |  | |
| **71** |  | |  | |  | |  | |  | |  | |  | |  | | 1,00 | |  | | 8,00 | | 6,00 | |  | |  | | 12,00 | |  | |  | |  | |  | | 2,76 | |  | | |  |  | |  | | |  | |  | |  | |  | |  | |  | |  | |  | |  | |  | | 0,00 | |  | |  | |  | |
| **72** |  | |  | |  | |  | |  | |  | |  | |  | | 1,00 | |  | | 33,00 | | 30,00 | |  | |  | |  | |  | |  | |  | |  | |  | |  | | |  |  | |  | | |  | |  | |  | |  | |  | |  | |  | |  | |  | |  | | 1,00 | |  | |  | |  | |
| **73** |  | |  | |  | |  | |  | |  | |  | |  | |  | |  | | 9,00 | | 7,00 | |  | |  | |  | |  | |  | |  | |  | |  | |  | | |  |  | |  | | |  | |  | |  | |  | |  | |  | |  | |  | |  | |  | | 1,00 | |  | |  | |  | |
| **74** |  | |  | |  | |  | |  | |  | |  | |  | |  | | 121,00 | | 11,00 | | 4,00 | |  | | 6,00 | |  | |  | |  | |  | |  | |  | |  | | |  |  | |  | | |  | |  | |  | |  | |  | |  | |  | |  | | 29,00 | |  | |  | |  | |  | |  | |
| **75** |  | |  | |  | |  | |  | |  | |  | |  | |  | | 37,00 | | 1,00 | | 2,00 | |  | | 1,00 | |  | | 40,00 | | 5,00 | |  | |  | |  | |  | | |  |  | |  | | |  | |  | |  | |  | |  | |  | |  | |  | |  | |  | | 0,00 | |  | |  | |  | |
| **77** |  | |  | |  | |  | |  | |  | |  | |  | |  | |  | | 6,00 | | 9,00 | | 7,00 | |  | |  | |  | |  | | 2,00 | |  | |  | |  | | |  |  | |  | | |  | |  | |  | |  | |  | |  | |  | |  | |  | |  | | 4,00 | | 7,00 | |  | | 63,00 | |
| **78.1** |  | |  | |  | |  | |  | |  | |  | |  | |  | |  | | 2,00 | | 3,00 | | 4,00 | |  | |  | |  | |  | |  | |  | |  | |  | | |  |  | |  | | |  | |  | |  | |  | |  | |  | |  | |  | |  | |  | |  | |  | |  | |  | |
| **78.2** |  | |  | |  | |  | |  | |  | |  | |  | |  | |  | | 12,00 | | 7,00 | | 12,00 | |  | |  | |  | |  | |  | |  | |  | |  | | |  |  | |  | | |  | |  | |  | |  | |  | |  | |  | |  | |  | |  | |  | |  | |  | |  | |
| **79** |  | | 54,00 | |  | |  | |  | |  | |  | |  | |  | | 62,70 | | 9,00 | |  | |  | |  | |  | | 37,30 | | 4,50 | |  | |  | | 2,76 | |  | | |  |  | |  | | | 0,95 | |  | |  | |  | |  | |  | |  | |  | |  | |  | |  | |  | |  | |  | |
| **80** |  | |  | |  | |  | |  | |  | |  | |  | |  | | 145,00 | | 51,00 | | 162,00 | |  | |  | |  | | 79,00 | | 14,00 | |  | |  | | 2,20 | |  | | |  |  | |  | | |  | |  | |  | |  | |  | |  | |  | |  | |  | |  | |  | |  | |  | |  | |
| **82** |  | |  | |  | |  | |  | |  | |  | |  | |  | | 144,00 | |  | | 22,00 | |  | | 11,00 | |  | | 65,00 | | 108,00 | |  | |  | |  | |  | | |  |  | |  | | | 0,86 | |  | |  | |  | |  | |  | |  | |  | |  | |  | | 13,00 | |  | |  | | 348,00 | |
| **83** |  | | 57,70 | | 130,30 | |  | |  | |  | |  | |  | | 0,00 | | 1,00 | |  | | 0,00 | |  | |  | |  | |  | |  | |  | |  | |  | |  | | |  |  | |  | | |  | |  | |  | |  | |  | |  | |  | |  | |  | |  | |  | |  | |  | |  | |
| **86** |  | |  | |  | |  | |  | |  | |  | |  | | 0,00 | | 5,00 | | 2,00 | | 0,00 | |  | | 0,00 | |  | | 2,00 | | 4,00 | |  | |  | |  | |  | | |  |  | |  | | |  | |  | |  | |  | |  | |  | |  | |  | |  | |  | |  | |  | |  | |  | |
| **87.1** |  | |  | |  | |  | |  | |  | |  | |  | |  | |  | |  | |  | |  | |  | | 14,00 | |  | |  | |  | |  | | 1,40 | |  | | |  |  | |  | | |  | |  | |  | |  | |  | |  | |  | |  | |  | |  | |  | |  | |  | |  | |
| **87.2** |  | |  | |  | |  | |  | |  | |  | |  | |  | |  | |  | |  | |  | |  | | 16,00 | |  | |  | |  | |  | | 1,40 | |  | | |  |  | |  | | |  | |  | |  | |  | |  | |  | |  | |  | |  | |  | |  | |  | |  | |  | |
| **93** |  | |  | |  | | 6,00 | |  | |  | |  | |  | |  | | 16,00 | | 3,00 | | 6,00 | | 2,00 | |  | |  | |  | |  | |  | |  | |  | |  | | |  |  | |  | | |  | |  | |  | |  | |  | |  | |  | |  | |  | | 7,00 | | 6,00 | | 5,00 | |  | | 71,00 | |
| **98** |  | | 56,00 | |  | |  | |  | |  | |  | |  | |  | |  | |  | |  | |  | |  | |  | |  | |  | | 6,00 | |  | |  | |  | | |  |  | |  | | |  | |  | |  | |  | |  | |  | |  | |  | | 28,00 | | 20,00 | | 20,00 | | 13,00 | |  | | 166,00 | |
| **99.1** |  | |  | |  | |  | |  | |  | |  | |  | |  | |  | |  | | 36,00 | |  | | 116,00 | |  | |  | |  | |  | |  | |  | |  | | |  |  | |  | | |  | |  | |  | |  | |  | |  | |  | |  | |  | |  | |  | |  | |  | |  | |
| **99.2** |  | |  | |  | |  | |  | |  | |  | |  | |  | |  | |  | | 184,00 | |  | | 624,00 | |  | |  | |  | |  | |  | |  | |  | | |  |  | |  | | |  | |  | |  | |  | |  | |  | |  | |  | |  | |  | |  | |  | |  | |  | |
| **101.1** |  | |  | |  | |  | |  | |  | |  | |  | |  | | 29,00 | | 4,00 | | 4,00 | | 9,00 | | 5,00 | |  | |  | |  | |  | |  | | 5,00 | |  | | |  |  | |  | | |  | |  | |  | |  | |  | |  | |  | |  | |  | |  | | 6,00 | | 1,00 | |  | | 11,00 | |
| **101.2** |  | |  | |  | |  | |  | |  | |  | |  | |  | | 103,00 | | 29,00 | | 26,00 | | 39,00 | | 20,00 | |  | |  | |  | |  | |  | | 6,00 | |  | | |  |  | |  | | |  | |  | |  | |  | |  | |  | |  | |  | |  | |  | | 17,00 | | 15,00 | |  | | 41,00 | |
| **102** |  | |  | |  | |  | |  | |  | |  | |  | | 56,00 | | 134,00 | | 24,00 | | 43,00 | | 30,00 | | 13,00 | | 70,00 | |  | |  | | 7,00 | | 8,00 | | 5,62 | |  | | |  |  | |  | | |  | |  | |  | |  | |  | |  | |  | |  | |  | |  | | 13,00 | |  | |  | |  | |
| **103** |  | |  | |  | |  | |  | |  | |  | |  | |  | | 162,00 | | 8,00 | | 32,00 | |  | | 3,00 | | 100,00 | | 107,00 | | 71,00 | |  | |  | |  | |  | | |  |  | |  | | |  | |  | |  | |  | |  | |  | |  | |  | |  | |  | |  | |  | |  | |  | |
| **104** |  | |  | |  | |  | |  | |  | |  | |  | | 4,00 | | 10,00 | | 10,00 | | 6,00 | |  | | 4,00 | |  | |  | | 17,00 | |  | |  | |  | |  | | |  |  | |  | | |  | |  | |  | |  | |  | |  | |  | |  | |  | |  | |  | | 30,00 | |  | |  | |
| **105** |  | |  | |  | |  | |  | |  | |  | |  | | 6,00 | | 294,00 | |  | | 33,00 | | 48,00 | |  | |  | |  | | 50,00 | |  | |  | | 5,20 | |  | | |  |  | |  | | |  | |  | |  | |  | |  | |  | |  | |  | |  | |  | |  | |  | |  | |  | |
| **107** |  | | 57,80 | |  | |  | |  | |  | |  | |  | | 7,00 | | 23,00 | | 2,00 | | 9,00 | | 2,00 | | 7,00 | |  | |  | | 36,00 | |  | |  | | 4,00 | |  | | |  |  | |  | | |  | |  | |  | |  | |  | |  | |  | |  | |  | |  | |  | | 62,00 | |  | | 27,00 | |
| **108** |  | | 62,50 | |  | |  | |  | |  | |  | |  | | 4,00 | |  | | 5,00 | | 8,00 | |  | | 6,00 | |  | |  | |  | |  | |  | | 1,82 | |  | | |  |  | |  | | |  | |  | |  | |  | |  | |  | |  | |  | |  | |  | |  | | 68,00 | |  | | 4,00 | |
| **110** |  | |  | |  | |  | |  | |  | |  | |  | |  | | 161,00 | |  | | 21,00 | | 19,00 | |  | | 104,00 | | 84,00 | | 54,00 | |  | |  | | 1,13 | |  | | | 13,70 |  | |  | | |  | |  | |  | |  | |  | |  | |  | |  | |  | |  | |  | |  | |  | |  | |
| **111** |  | |  | |  | |  | |  | |  | |  | |  | |  | | 290,00 | |  | |  | | 54,00 | | 27,00 | |  | | 305,00 | | 77,00 | |  | |  | | 1,30 | |  | | | 13,50 |  | |  | | |  | |  | |  | |  | |  | |  | |  | |  | |  | |  | |  | |  | |  | |  | |
| **112** |  | |  | |  | |  | |  | |  | |  | |  | |  | |  | | 11,00 | | 25,00 | | 32,00 | | 39,00 | | 93,00 | |  | | 25,00 | |  | |  | | 1,90 | |  | | |  |  | |  | | |  | |  | |  | |  | |  | |  | |  | |  | |  | |  | |  | |  | |  | | 116,00 | |
| **113.1** |  | |  | |  | |  | |  | |  | |  | |  | | 4,00 | | 17,00 | | 4,00 | | 0,00 | |  | | 1,00 | |  | | 12,00 | | 6,00 | |  | |  | | 3,10 | |  | | |  |  | |  | | |  | |  | |  | |  | |  | |  | |  | |  | |  | | 1,00 | | 1,00 | |  | |  | | 46,00 | |
| **113.2** |  | |  | |  | |  | |  | |  | |  | |  | | 1,00 | | 14,00 | | 5,00 | | 5,00 | |  | | 2,00 | |  | | 12,00 | | 2,00 | |  | |  | | 5,40 | |  | | |  |  | |  | | |  | |  | |  | |  | |  | |  | |  | |  | |  | | 0,00 | | 0,00 | |  | |  | | 50,00 | |
| **115.1** |  | |  | |  | |  | |  | |  | |  | |  | |  | | 463,00 | | 32,00 | | 39,00 | | 27,00 | |  | | 47,00 | | 276,00 | | 79,00 | |  | |  | | 1,96 | |  | | |  |  | |  | | |  | |  | |  | |  | |  | |  | |  | |  | |  | |  | | 11,00 | | 97,00 | |  | | 756,00 | |
| **115.2** |  | |  | |  | |  | |  | |  | |  | |  | |  | | 229,00 | | 17,00 | | 16,00 | | 15,00 | |  | | 17,00 | | 139,00 | | 44,00 | |  | |  | | 1,80 | |  | | |  |  | |  | | |  | |  | |  | |  | |  | |  | |  | |  | |  | |  | | 4,00 | | 35,00 | |  | | 390,00 | |
| **115.3** |  | |  | |  | |  | |  | |  | |  | |  | |  | | 169,00 | | 12,00 | | 12,00 | | 7,00 | |  | | 8,00 | | 105,00 | | 34,00 | |  | |  | | 1,75 | |  | | |  |  | |  | | |  | |  | |  | |  | |  | |  | |  | |  | |  | |  | | 4,00 | | 22,00 | |  | | 278,00 | |
| **116.1** |  | |  | |  | |  | |  | |  | |  | |  | | 7,00 | | 62,00 | | 15,00 | | 13,00 | | 12,00 | | 16,00 | |  | |  | |  | | 2,00 | |  | |  | |  | | |  |  | |  | | | 1,00 | |  | |  | |  | |  | |  | |  | |  | |  | |  | | 10,00 | | 5,00 | |  | | 99,00 | |
| **116.2** |  | |  | |  | |  | |  | |  | |  | |  | | 3,00 | | 65,00 | | 16,00 | | 13,00 | | 16,00 | | 13,00 | |  | |  | |  | | 7,00 | |  | |  | |  | | |  |  | |  | | | 1,00 | |  | |  | |  | |  | |  | |  | |  | |  | |  | | 5,00 | | 11,00 | |  | | 89,00 | |
| **117.1** |  | | 57,80 | |  | |  | |  | |  | |  | |  | |  | | 27,00 | |  | |  | |  | |  | |  | |  | |  | |  | |  | |  | |  | | |  |  | |  | | |  | |  | |  | |  | |  | |  | |  | |  | |  | |  | |  | |  | |  | |  | |
| **117.2** |  | | 58,29 | |  | |  | |  | |  | |  | |  | |  | | 14,00 | |  | |  | |  | |  | |  | |  | |  | |  | |  | |  | |  | | |  |  | |  | | |  | |  | |  | |  | |  | |  | |  | |  | |  | |  | |  | |  | |  | |  | |
| **118** |  | |  | |  | |  | |  | |  | |  | |  | |  | |  | | 55,00 | | 39,00 | |  | | 31,00 | | 264,00 | |  | |  | | 16,00 | |  | |  | |  | | |  |  | |  | | |  | |  | |  | |  | |  | |  | |  | |  | |  | |  | | 14,00 | | 16,00 | |  | | 534,00 | |
| **119.1** |  | |  | |  | |  | |  | |  | |  | |  | |  | |  | | 74,00 | | 64,00 | |  | |  | |  | |  | |  | |  | |  | |  | |  | | |  |  | |  | | |  | |  | |  | |  | |  | |  | |  | |  | |  | |  | | 0,00 | |  | |  | | 8,00 | |
| **119.2** |  | |  | |  | |  | |  | |  | |  | |  | |  | |  | | 16,00 | | 11,00 | |  | |  | |  | |  | |  | |  | |  | |  | |  | | |  |  | |  | | |  | |  | |  | |  | |  | |  | |  | |  | |  | |  | | 1,00 | |  | |  | | 7,00 | |
| **121.1** |  | |  | |  | |  | |  | |  | |  | |  | |  | |  | |  | |  | |  | |  | |  | |  | |  | |  | |  | | 4,00 | |  | | |  |  | |  | | | 0,90 | |  | |  | |  | |  | |  | |  | |  | |  | |  | |  | |  | |  | |  | |
| **121.2** |  | |  | |  | |  | |  | |  | |  | |  | |  | |  | |  | |  | |  | |  | |  | |  | |  | |  | |  | | 3,00 | |  | | |  |  | |  | | | 0,90 | |  | |  | |  | |  | |  | |  | |  | |  | |  | |  | |  | |  | |  | |
| **122.1** |  | |  | |  | |  | |  | |  | |  | | 2,00 | | 11,00 | | 31,00 | |  | | 1,00 | | 2,00 | | 0,00 | | 14,00 | | 9,00 | | 9,00 | |  | |  | | 1,30 | |  | | |  |  | |  | | |  | |  | |  | |  | |  | |  | |  | |  | |  | |  | |  | |  | |  | |  | |
| **122.2** |  | |  | |  | |  | |  | |  | |  | | 0,00 | | 7,00 | | 31,00 | |  | | 2,00 | | 3,00 | | 2,00 | | 17,00 | | 8,00 | | 14,00 | |  | |  | | 1,30 | |  | | |  |  | |  | | |  | |  | |  | |  | |  | |  | |  | |  | |  | |  | |  | |  | |  | |  | |
| **123.1** |  | |  | |  | |  | |  | |  | |  | |  | | 1,00 | | 49,00 | | 27,00 | | 6,00 | | 49,00 | |  | |  | |  | | 26,00 | |  | |  | |  | |  | | |  |  | |  | | |  | |  | |  | |  | |  | |  | |  | |  | |  | |  | |  | |  | |  | |  | |
| **123.2** |  | |  | |  | |  | |  | |  | |  | |  | | 0,00 | | 19,00 | | 4,00 | | 3,00 | | 19,00 | |  | |  | |  | | 18,00 | |  | |  | |  | |  | | |  |  | |  | | |  | |  | |  | |  | |  | |  | |  | |  | |  | |  | |  | |  | |  | |  | |
| **127** | 0,50 | |  | |  | |  | |  | |  | |  | |  | | 6,00 | |  | | 9,00 | |  | | 6,00 | |  | | 60,00 | |  | | 31,00 | |  | |  | | 1,70 | |  | | |  |  | |  | | | 0,90 | |  | |  | |  | |  | |  | |  | |  | |  | |  | | 9,00 | |  | |  | | 1,00 | |
| **128** |  | |  | |  | |  | |  | |  | |  | |  | |  | |  | |  | |  | |  | |  | |  | |  | |  | |  | |  | |  | |  | | | 13,00 |  | | 43,90 | | | 0,82 | |  | |  | |  | |  | |  | |  | |  | |  | |  | |  | |  | |  | |  | |
| **129** |  | |  | |  | |  | |  | |  | |  | |  | | 89,00 | |  | | 14,00 | | 2,00 | |  | | 9,00 | |  | |  | | 32,00 | |  | |  | | 3,20 | | 4,50 | | |  |  | |  | | |  | |  | |  | |  | |  | |  | |  | |  | |  | |  | | 4,00 | |  | |  | |  | |
| **130** |  | |  | |  | |  | |  | |  | |  | |  | |  | | 94,00 | | 12,00 | | 10,00 | |  | |  | |  | | 58,00 | | 20,00 | |  | |  | | 0,80 | |  | | |  |  | |  | | |  | |  | |  | |  | |  | |  | |  | |  | |  | |  | |  | |  | |  | |  | |
| **131.1** |  | |  | |  | |  | |  | |  | |  | |  | |  | |  | | 6,00 | | 5,00 | |  | | 8,00 | |  | |  | |  | |  | |  | | 2,50 | |  | | |  |  | |  | | | 1,00 | |  | |  | |  | |  | |  | |  | |  | |  | |  | |  | |  | |  | |  | |
| **131.2** |  | |  | |  | |  | |  | |  | |  | |  | |  | |  | | 11,00 | | 11,00 | |  | | 6,00 | |  | |  | |  | |  | |  | | 2,70 | |  | | |  |  | |  | | | 1,08 | |  | |  | |  | |  | |  | |  | |  | |  | |  | |  | |  | |  | |  | |
| **132 .1** |  | |  | |  | |  | |  | |  | |  | | 3,00 | | 0,00 | | 50,00 | |  | | 2,00 | |  | |  | |  | |  | |  | |  | |  | |  | |  | | |  |  | |  | | |  | |  | |  | |  | |  | |  | |  | |  | |  | |  | | 0,00 | |  | |  | | 109,00 | |
| **132 .2** |  | |  | |  | |  | |  | |  | |  | | 36,00 | | 2,00 | | 653,00 | |  | | 74,00 | |  | |  | |  | |  | |  | |  | |  | |  | |  | | |  |  | |  | | |  | |  | |  | |  | |  | |  | |  | |  | |  | |  | | 35,00 | |  | |  | | 1123,00 | |
| **133** |  | |  | |  | |  | |  | |  | |  | | 3,00 | | 1,00 | | 27,00 | |  | | 12,00 | | 7,00 | |  | | 3,00 | |  | |  | |  | |  | | 1,47 | |  | | |  |  | |  | | |  | |  | |  | |  | |  | |  | |  | |  | |  | |  | |  | |  | |  | |  | |
| **134.1** |  | |  | |  | |  | |  | |  | |  | |  | |  | | 69,00 | | 11,00 | | 4,00 | |  | |  | |  | |  | | 16,00 | |  | |  | | 1,10 | |  | | | 14,00 |  | |  | | | 0,92 | |  | |  | |  | |  | |  | |  | |  | |  | |  | |  | |  | |  | |  | |
| **134.2** |  | |  | |  | |  | |  | |  | |  | |  | |  | | 128,00 | | 17,00 | | 14,00 | |  | |  | |  | |  | | 32,00 | |  | |  | | 0,81 | |  | | | 14,10 |  | |  | | | 0,89 | |  | |  | |  | |  | |  | |  | |  | |  | |  | |  | |  | |  | |  | |
| **135 .1** |  | |  | |  | |  | |  | |  | |  | |  | |  | |  | |  | |  | |  | |  | |  | |  | |  | |  | |  | | 5,00 | |  | | |  |  | |  | | |  | |  | |  | |  | |  | |  | |  | |  | |  | |  | |  | |  | |  | |  | |
| **135.2** |  | |  | |  | |  | |  | |  | |  | |  | |  | |  | |  | |  | |  | |  | |  | |  | |  | |  | |  | | 5,70 | |  | | |  |  | |  | | |  | |  | |  | |  | |  | |  | |  | |  | |  | |  | |  | |  | |  | |  | |
| **136.1** |  | |  | |  | |  | |  | |  | |  | |  | |  | |  | |  | |  | |  | |  | |  | |  | |  | |  | |  | |  | |  | | |  |  | |  | | |  | |  | |  | |  | |  | |  | |  | |  | |  | |  | |  | |  | |  | |  | |
| **136.2** |  | |  | |  | |  | |  | |  | |  | |  | |  | |  | |  | |  | |  | |  | |  | |  | |  | |  | |  | |  | |  | | |  |  | |  | | |  | |  | |  | |  | |  | |  | |  | |  | |  | |  | |  | |  | |  | |  | |
| **136.3** |  | |  | |  | |  | |  | |  | |  | |  | |  | |  | |  | |  | |  | |  | |  | |  | |  | |  | |  | |  | |  | | |  |  | |  | | |  | |  | |  | |  | |  | |  | |  | |  | |  | |  | |  | |  | |  | |  | |
| **136.4** |  | |  | |  | |  | |  | |  | |  | |  | |  | |  | |  | |  | |  | |  | |  | |  | |  | |  | |  | |  | |  | | |  |  | |  | | |  | |  | |  | |  | |  | |  | |  | |  | |  | |  | |  | |  | |  | |  | |
| **136.5** |  | |  | |  | |  | |  | |  | |  | |  | |  | |  | |  | |  | |  | |  | |  | |  | |  | |  | |  | |  | |  | | |  |  | |  | | |  | |  | |  | |  | |  | |  | |  | |  | |  | |  | |  | |  | |  | |  | |
| **137** |  | | 53,00 | |  | |  | |  | |  | |  | |  | | 6,00 | | 7,00 | | 2,00 | | 1,00 | |  | |  | |  | | 3,00 | |  | |  | |  | |  | |  | | |  |  | | 39,00 | | |  | |  | |  | |  | |  | |  | |  | |  | |  | |  | |  | |  | |  | |  | |
| **138** |  | |  | |  | |  | |  | |  | |  | |  | |  | | 9,00 | | 1,00 | | 2,00 | | 8,00 | | 6,00 | |  | |  | | 1,00 | |  | |  | |  | |  | | |  |  | |  | | |  | |  | |  | |  | |  | |  | |  | |  | |  | |  | |  | |  | |  | |  | |
| **139.1** |  | |  | |  | |  | |  | |  | |  | |  | |  | |  | | 5,00 | | 3,00 | |  | |  | | 26,00 | |  | |  | |  | |  | | 10,90 | |  | | |  |  | |  | | |  | |  | |  | |  | |  | |  | |  | |  | | 9,00 | |  | |  | |  | | 11,00 | |  | |
| **139.2** |  | |  | |  | |  | |  | |  | |  | |  | |  | |  | | 16,00 | | 21,00 | |  | |  | | 58,00 | |  | |  | |  | |  | | 11,70 | |  | | |  |  | |  | | |  | |  | |  | |  | |  | |  | |  | |  | | 4,00 | |  | |  | |  | | 31,00 | |  | |
| **139.3** |  | |  | |  | |  | |  | |  | |  | |  | |  | |  | | 85,00 | | 77,00 | |  | |  | | 452,00 | |  | |  | |  | |  | | 1,00 | |  | | |  |  | |  | | |  | |  | |  | |  | |  | |  | |  | |  | | 340,00 | |  | |  | |  | | 505,00 | |  | |
| **139.4** |  | |  | |  | |  | |  | |  | |  | |  | |  | |  | | 203,00 | | 191,00 | |  | |  | | 1111,00 | |  | |  | |  | |  | | 1,10 | |  | | |  |  | |  | | |  | |  | |  | |  | |  | |  | |  | |  | | 319,00 | |  | |  | |  | | 1238,00 | |  | |
| **140** |  | |  | |  | |  | |  | |  | |  | |  | | 39,00 | |  | | 31,00 | | 24,00 | | 57,00 | | 26,00 | |  | |  | |  | |  | |  | | 1,10 | |  | | |  |  | |  | | |  | |  | |  | |  | |  | |  | |  | |  | |  | |  | | 6,00 | |  | |  | |  | |
| **141** |  | | 52,90 | |  | |  | |  | |  | |  | |  | |  | | 100,00 | | 23,00 | | 51,00 | |  | | 39,00 | |  | |  | |  | |  | |  | |  | |  | | |  |  | |  | | |  | |  | |  | |  | |  | |  | |  | |  | |  | |  | |  | |  | |  | |  | |
| **142.1** |  | |  | |  | |  | |  | |  | |  | |  | |  | |  | |  | |  | |  | |  | |  | |  | |  | |  | |  | |  | |  | | |  |  | |  | | |  | |  | |  | |  | |  | |  | |  | |  | |  | |  | | 95,00 | |  | |  | |  | |
| **142.2** |  | |  | |  | |  | |  | |  | |  | |  | |  | |  | |  | |  | |  | |  | |  | |  | |  | |  | |  | |  | |  | | |  |  | |  | | |  | |  | |  | |  | |  | |  | |  | |  | |  | |  | | 39,00 | |  | |  | |  | |
| **143.1** |  | |  | |  | |  | |  | |  | |  | | 1,00 | | 22,00 | | 45,00 | | 13,00 | | 11,00 | |  | | 3,00 | | 32,00 | | 40,00 | | 12,00 | | 3,00 | |  | |  | |  | | |  |  | |  | | |  | |  | |  | |  | |  | |  | |  | |  | |  | |  | | 2,00 | |  | |  | |  | |
| **143.2** |  | |  | |  | |  | |  | |  | |  | | 12,00 | | 133,00 | | 555,00 | | 122,00 | | 22,00 | |  | | 69,00 | | 368,00 | | 372,00 | | 184,00 | | 65,00 | |  | |  | |  | | |  |  | |  | | |  | |  | |  | |  | |  | |  | |  | |  | |  | |  | | 41,00 | |  | |  | |  | |
| **144.1** |  | |  | |  | |  | |  | |  | |  | |  | | 16,00 | | 37,00 | | 7,00 | | 2,00 | | 11,00 | | 20,00 | | 14,00 | | 22,00 | | 13,00 | |  | |  | |  | |  | | |  |  | |  | | |  | |  | |  | |  | |  | |  | |  | |  | |  | |  | |  | |  | |  | |  | |
| **144.2** |  | |  | |  | |  | |  | |  | |  | |  | | 148,00 | | 591,00 | | 133,00 | | 33,00 | | 250,00 | | 54,00 | | 399,00 | | 401,00 | | 190,00 | |  | |  | |  | |  | | |  |  | |  | | |  | |  | |  | |  | |  | |  | |  | |  | |  | |  | |  | |  | |  | |  | |
| **145** |  | |  | |  | |  | |  | |  | |  | |  | | 2,00 | | 29,00 | | 8,00 | | 13,00 | | 17,00 | | 8,00 | |  | |  | |  | |  | |  | |  | |  | | |  |  | |  | | |  | |  | |  | |  | |  | |  | |  | |  | |  | |  | |  | |  | |  | |  | |
| **146** |  | |  | |  | |  | |  | |  | |  | |  | |  | |  | | 19,00 | |  | |  | |  | |  | |  | |  | |  | |  | |  | |  | | |  |  | |  | | |  | |  | |  | |  | |  | |  | |  | |  | |  | |  | |  | |  | |  | |  | |
| **147** |  | |  | |  | |  | |  | |  | |  | |  | | 20,00 | |  | | 21,00 | | 13,00 | | 32,00 | | 15,00 | |  | |  | |  | |  | |  | | 1,00 | |  | | |  |  | |  | | |  | |  | |  | |  | |  | |  | |  | |  | |  | |  | |  | |  | |  | |  | |
| **148 .1** |  | |  | |  | |  | |  | |  | |  | |  | |  | | 22,00 | | 1,00 | | 1,00 | |  | |  | |  | |  | |  | |  | |  | | 0,70 | |  | | |  |  | |  | | |  | |  | |  | |  | |  | |  | |  | |  | | 3,00 | |  | |  | |  | |  | |  | |
| **148.2** |  | |  | |  | |  | |  | |  | |  | |  | |  | | 56,00 | | 5,00 | | 3,00 | |  | |  | |  | |  | |  | |  | |  | | 0,80 | |  | | |  |  | |  | | |  | |  | |  | |  | |  | |  | |  | |  | | 10,00 | |  | |  | |  | |  | |  | |
| **149.1** |  | |  | |  | |  | |  | |  | |  | |  | |  | | 56,00 | |  | | 1,00 | | 4,00 | |  | |  | |  | |  | |  | |  | | 0,89 | |  | | |  |  | |  | | |  | |  | |  | |  | |  | |  | |  | |  | |  | |  | | 0,00 | |  | |  | |  | |
| **149.2** |  | |  | |  | |  | |  | |  | |  | |  | |  | | 147,00 | |  | | 10,00 | | 19,00 | |  | |  | |  | |  | |  | |  | | 1,00 | |  | | |  |  | |  | | |  | |  | |  | |  | |  | |  | |  | |  | |  | |  | | 4,00 | |  | |  | |  | |
| **149.3** |  | |  | |  | |  | |  | |  | |  | |  | |  | | 47,00 | |  | | 1,00 | | 4,00 | | 4,00 | |  | |  | |  | |  | |  | | 0,94 | |  | | |  |  | |  | | |  | |  | |  | |  | |  | |  | |  | |  | |  | |  | |  | |  | |  | |  | |
| **149.4** |  | |  | |  | |  | |  | |  | |  | |  | |  | | 56,00 | |  | | 1,00 | | 3,00 | | 4,00 | |  | |  | |  | |  | |  | | 0,71 | |  | | |  |  | |  | | |  | |  | |  | |  | |  | |  | |  | |  | |  | |  | |  | |  | |  | |  | |
| **150** |  | |  | |  | |  | |  | |  | |  | |  | | 11,00 | |  | | 2,00 | | 6,00 | |  | |  | |  | |  | | 20,00 | |  | |  | | 2,67 | |  | | |  |  | |  | | |  | |  | |  | |  | |  | |  | |  | |  | |  | |  | |  | |  | |  | |  | |
| **151** |  | |  | |  | |  | |  | |  | |  | |  | |  | |  | |  | | 4,00 | | 22,00 | |  | |  | |  | |  | |  | |  | |  | |  | | |  |  | |  | | |  | |  | |  | |  | |  | |  | |  | |  | |  | |  | |  | |  | |  | |  | |
| **152 .1** |  | |  | |  | |  | |  | |  | |  | |  | |  | | 44,00 | | 11,00 | | 12,00 | |  | | 5,00 | |  | |  | | 30,00 | |  | |  | |  | |  | | |  |  | |  | | | 0,98 | |  | |  | |  | |  | |  | |  | | 102,00 | |  | |  | |  | |  | |  | |  | |
| **152.2** |  | |  | |  | |  | |  | |  | |  | |  | |  | | 21,00 | | 6,00 | | 6,00 | |  | | 4,00 | |  | |  | | 10,00 | |  | |  | |  | |  | | |  |  | |  | | | 0,93 | |  | |  | |  | |  | |  | |  | | 96,00 | |  | |  | |  | |  | |  | |  | |
| **152.3** |  | |  | |  | |  | |  | |  | |  | |  | |  | | 14,00 | | 7,00 | | 3,00 | |  | | 1,00 | |  | |  | | 9,00 | |  | |  | |  | |  | | |  |  | |  | | | 0,96 | |  | |  | |  | |  | |  | |  | | 103,00 | |  | |  | |  | |  | |  | |  | |
| **153.1** |  | |  | |  | |  | |  | |  | |  | |  | |  | |  | |  | | 7,00 | | 4,00 | |  | |  | |  | |  | |  | |  | |  | |  | | |  |  | |  | | |  | |  | |  | |  | |  | |  | |  | |  | |  | |  | |  | |  | |  | | 225,00 | |
| **153.2** |  | |  | |  | |  | |  | |  | |  | |  | |  | |  | |  | | 8,00 | | 3,00 | |  | |  | |  | |  | |  | |  | |  | |  | | |  |  | |  | | |  | |  | |  | |  | |  | |  | |  | |  | |  | |  | |  | |  | |  | | 115,00 | |
| **155.1** |  | |  | |  | |  | |  | |  | |  | |  | | 0,00 | |  | |  | | 3,00 | |  | |  | |  | |  | |  | |  | |  | |  | |  | | |  |  | |  | | |  | |  | |  | |  | |  | |  | |  | |  | |  | |  | |  | |  | |  | |  | |
| **155.2** |  | |  | |  | |  | |  | |  | |  | |  | | 1,00 | |  | |  | | 3,00 | |  | |  | |  | |  | |  | |  | |  | |  | |  | | |  |  | |  | | |  | |  | |  | |  | |  | |  | |  | |  | |  | |  | |  | |  | |  | |  | |
| **156.1** |  | |  | |  | |  | |  | |  | |  | | 0,00 | | 43,00 | | 42,00 | | 2,00 | | 2,00 | |  | | 1,00 | |  | | 17,00 | |  | |  | |  | | 2,76 | |  | | |  |  | |  | | | 1,10 | |  | |  | |  | |  | |  | |  | |  | |  | |  | |  | |  | |  | |  | |
| **156.2** |  | |  | |  | |  | |  | |  | |  | | 2,00 | | 42,00 | | 45,00 | | 2,00 | | 2,00 | |  | | 2,00 | |  | | 22,00 | |  | |  | |  | | 3,49 | |  | | |  |  | |  | | | 1,05 | |  | |  | |  | |  | |  | |  | |  | |  | |  | |  | |  | |  | |  | |
| **157.1** |  | | 51,10 | |  | |  | |  | |  | |  | |  | | 1,00 | | 18,00 | | 6,00 | | 5,00 | |  | | 3,00 | |  | |  | |  | |  | |  | |  | |  | | |  |  | |  | | |  | |  | |  | |  | |  | |  | |  | |  | |  | | 9,00 | | 10,00 | | 7,00 | |  | | 95,00 | |
| **157.2** |  | | 52,10 | |  | |  | |  | |  | |  | |  | | 2,00 | | 24,00 | | 5,00 | | 7,00 | |  | | 5,00 | |  | |  | |  | |  | |  | |  | |  | | |  |  | |  | | |  | |  | |  | |  | |  | |  | |  | |  | |  | | 5,00 | | 15,00 | | 4,00 | |  | | 80,00 | |
| **158.1** |  | |  | |  | |  | |  | |  | |  | | 210,00 | |  | | 4296,00 | | 53,00 | | 499,00 | |  | | 163,00 | |  | |  | |  | |  | |  | |  | |  | | |  |  | |  | | | 0,98 | |  | |  | |  | |  | |  | |  | |  | |  | |  | |  | |  | |  | |  | |
| **158.2** |  | |  | |  | |  | |  | |  | |  | | 722,00 | |  | | 9472,00 | | 272,00 | | 1370,00 | |  | | 436,00 | |  | |  | |  | |  | |  | |  | |  | | |  |  | |  | | | 0,97 | |  | |  | |  | |  | |  | |  | |  | |  | |  | |  | |  | |  | |  | |
| **160** |  | |  | |  | |  | |  | |  | |  | |  | |  | | 11,00 | | 0,00 | | 1,00 | |  | |  | | 6,00 | |  | |  | |  | |  | | 0,78 | |  | | |  |  | |  | | | 0,80 | |  | |  | |  | |  | |  | |  | |  | |  | |  | |  | |  | |  | |  | |
| **161** |  | |  | |  | |  | |  | |  | |  | |  | | 4,00 | | 38,00 | |  | |  | | 29,00 | |  | |  | |  | |  | |  | |  | |  | |  | | |  |  | |  | | |  | |  | |  | |  | |  | |  | |  | |  | |  | |  | | 0,00 | |  | |  | |  | |
| **162.1** |  | | 52,60 | |  | |  | |  | |  | |  | |  | | 9,00 | | 24,00 | | 2,00 | | 5,00 | |  | |  | |  | |  | |  | |  | |  | | 2,70 | |  | | |  |  | |  | | |  | |  | |  | |  | |  | |  | |  | |  | | 3,00 | |  | | 7,00 | | 5,00 | |  | | 35,00 | |
| **162 .2** |  | | 54,10 | |  | |  | |  | |  | |  | |  | | 40,00 | | 76,00 | | 8,00 | | 26,00 | |  | |  | |  | |  | |  | |  | |  | | 3,08 | |  | | |  |  | |  | | |  | |  | |  | |  | |  | |  | |  | |  | | 4,00 | |  | | 8,00 | | 18,00 | |  | | 90,00 | |
| **163.1** |  | |  | |  | |  | |  | |  | |  | |  | | 156,00 | | 108,00 | | 2,00 | | 8,00 | | 7,00 | | 3,00 | | 39,00 | | 46,00 | | 29,00 | |  | |  | |  | |  | | |  |  | |  | | | 0,82 | |  | |  | | 1,70 | |  | |  | |  | |  | |  | |  | |  | |  | |  | |  | |
| **163.2** |  | |  | |  | |  | |  | |  | |  | |  | | 156,00 | | 90,00 | | 4,00 | | 6,00 | | 7,00 | | 1,00 | | 38,00 | | 66,00 | | 15,00 | |  | |  | |  | |  | | |  |  | |  | | | 0,85 | |  | |  | | 2,00 | |  | |  | |  | |  | |  | |  | |  | |  | |  | |  | |
| **164** |  | | 50,40 | |  | |  | |  | |  | |  | |  | |  | |  | | 6,00 | |  | | 4,00 | | 2,00 | |  | |  | |  | |  | |  | |  | | 1,80 | | |  |  | |  | | | 1,00 | |  | |  | |  | |  | |  | |  | |  | |  | |  | |  | |  | |  | |  | |
| **169.1** |  | |  | |  | |  | |  | |  | |  | |  | |  | | 32,00 | | 4,00 | | 3,00 | |  | |  | |  | |  | |  | |  | |  | | 0,96 | |  | | | 14,10 |  | |  | | |  | |  | |  | | 0,86 | |  | |  | |  | |  | |  | |  | |  | |  | |  | |  | |
| **169.2** |  | |  | |  | |  | |  | |  | |  | |  | |  | | 151,00 | | 22,00 | | 12,00 | |  | |  | |  | |  | |  | |  | |  | | 0,90 | |  | | | 14,10 |  | |  | | |  | |  | |  | | 0,91 | |  | |  | |  | |  | |  | |  | |  | |  | |  | |  | |
| **170.1** |  | |  | |  | |  | |  | |  | |  | |  | |  | |  | | 7,00 | |  | |  | |  | |  | |  | |  | |  | |  | | 1,20 | |  | | |  |  | |  | | |  | |  | |  | |  | |  | |  | |  | |  | |  | |  | |  | |  | |  | | 250,00 | |
| **170.2** |  | |  | |  | |  | |  | |  | |  | |  | |  | |  | | 4,00 | |  | |  | |  | |  | |  | |  | |  | |  | | 1,20 | |  | | |  |  | |  | | |  | |  | |  | |  | |  | |  | |  | |  | |  | |  | |  | |  | |  | | 63,00 | |
| **170.3** |  | |  | |  | |  | |  | |  | |  | |  | |  | |  | | 6,00 | |  | |  | |  | |  | |  | |  | |  | |  | | 1,10 | |  | | |  |  | |  | | |  | |  | |  | |  | |  | |  | |  | |  | |  | |  | |  | |  | |  | | 149,00 | |
| **170.4** |  | |  | |  | |  | |  | |  | |  | |  | |  | |  | | 1,00 | |  | |  | |  | |  | |  | |  | |  | |  | | 1,30 | |  | | |  |  | |  | | |  | |  | |  | |  | |  | |  | |  | |  | |  | |  | |  | |  | |  | | 101,00 | |
| **171.1** |  | |  | |  | |  | |  | |  | |  | |  | |  | | 32,00 | | 14,00 | | 3,00 | |  | |  | |  | |  | |  | |  | |  | | 3,80 | |  | | |  |  | |  | | |  | |  | |  | |  | |  | |  | |  | |  | |  | |  | |  | |  | |  | |  | |
| **171.2** |  | |  | |  | |  | |  | |  | |  | |  | |  | | 20,00 | | 6,00 | | 4,00 | |  | |  | |  | |  | |  | |  | |  | | 4,00 | |  | | |  |  | |  | | |  | |  | |  | |  | |  | |  | |  | |  | |  | |  | |  | |  | |  | |  | |
| **172.1** |  | |  | |  | |  | |  | |  | |  | |  | |  | |  | |  | |  | |  | |  | |  | |  | |  | |  | |  | |  | |  | | |  |  | |  | | |  | |  | |  | |  | |  | |  | |  | |  | |  | |  | |  | |  | |  | |  | |
| **172.2** |  | |  | |  | |  | |  | |  | |  | |  | |  | |  | |  | |  | |  | |  | |  | |  | |  | |  | |  | |  | |  | | |  |  | |  | | |  | |  | |  | |  | |  | |  | |  | |  | |  | |  | |  | |  | |  | |  | |
| **173** |  | |  | |  | |  | |  | |  | |  | |  | |  | | 39,00 | | 7,00 | | 3,00 | |  | | 4,00 | |  | |  | |  | |  | |  | |  | |  | | |  |  | |  | | |  | |  | |  | |  | |  | |  | |  | |  | |  | |  | |  | |  | |  | |  | |
| **174** |  | |  | |  | |  | |  | |  | |  | |  | | 13,00 | |  | |  | |  | | 186,00 | |  | |  | |  | |  | |  | |  | |  | |  | | |  |  | |  | | |  | |  | |  | |  | |  | |  | |  | |  | |  | |  | | 48,00 | | 110,00 | |  | | 48,00 | |
| **177** |  | |  | |  | |  | |  | |  | |  | |  | | 83,00 | | 3896,00 | | 662,00 | | 653,00 | | 1001,00 | |  | | 2804,00 | |  | | 883,00 | |  | |  | | 1,31 | |  | | |  |  | |  | | |  | |  | |  | |  | |  | |  | |  | |  | |  | |  | | 272,00 | |  | |  | |  | |
| **177.2** |  | |  | |  | |  | |  | |  | |  | |  | |  | | 965,00 | | 199,00 | | 201,00 | | 237,00 | | 193,00 | | 564,00 | |  | | 252,00 | |  | |  | | 1,58 | |  | | |  |  | |  | | |  | |  | |  | |  | |  | |  | |  | |  | |  | |  | | 79,00 | |  | |  | |  | |
| **177.3** |  | |  | |  | |  | |  | |  | |  | |  | |  | | 2243,00 | | 287,00 | | 265,00 | | 485,00 | | 218,00 | | 1490,00 | |  | | 422,00 | |  | |  | | 1,10 | |  | | |  |  | |  | | |  | |  | |  | |  | |  | |  | |  | |  | |  | |  | | 124,00 | |  | |  | |  | |
| **178** |  | |  | |  | |  | |  | |  | |  | |  | | 7,00 | | 277,00 | | 28,00 | | 74,00 | |  | | 40,00 | |  | | 179,00 | | 145,00 | |  | |  | |  | |  | | |  |  | |  | | |  | |  | |  | |  | |  | |  | |  | |  | |  | |  | |  | |  | |  | |  | |
| **179** |  | | 51,77 | |  | |  | |  | |  | |  | |  | |  | | 16,00 | |  | | 3,00 | |  | |  | |  | | 1,00 | | 8,00 | |  | |  | |  | |  | | |  |  | |  | | |  | |  | |  | |  | |  | |  | |  | |  | |  | |  | |  | |  | |  | |  | |
| **180** |  | |  | |  | |  | |  | |  | |  | |  | |  | |  | |  | |  | |  | |  | |  | |  | |  | |  | |  | |  | |  | | |  |  | |  | | |  | |  | |  | |  | |  | |  | |  | |  | |  | |  | |  | |  | |  | |  | |
| **181** |  | |  | |  | |  | |  | |  | |  | |  | | 12,00 | | 44,00 | | 7,00 | | 18,00 | |  | |  | |  | |  | |  | |  | |  | |  | | 15,00 | | |  |  | |  | | |  | |  | |  | |  | |  | |  | |  | |  | |  | |  | | 92,00 | |  | |  | |  | |
| **182** |  | |  | |  | |  | |  | |  | |  | |  | |  | |  | |  | |  | |  | |  | |  | |  | |  | |  | |  | |  | |  | | |  |  | |  | | |  | |  | |  | |  | |  | |  | |  | |  | |  | |  | |  | |  | |  | |  | |
| **183.1** |  | |  | |  | |  | |  | |  | |  | |  | |  | |  | |  | |  | |  | |  | |  | |  | |  | |  | |  | |  | |  | | | 12,15 |  | |  | | | 0,82 | |  | |  | |  | |  | |  | |  | |  | | 23,00 | |  | |  | | 19,00 | |  | |  | |
| **183.2** |  | |  | |  | |  | |  | |  | |  | |  | |  | |  | |  | |  | |  | |  | |  | |  | |  | |  | |  | |  | |  | | | 10,52 |  | |  | | | 0,82 | |  | |  | |  | |  | |  | |  | |  | | 10,00 | |  | |  | | 13,00 | |  | |  | |
| **184.1** |  | |  | |  | |  | |  | |  | |  | |  | |  | | 22,00 | | 2,00 | |  | | 8,00 | | 4,00 | |  | |  | |  | |  | |  | | 1,25 | |  | | |  |  | |  | | |  | |  | |  | |  | |  | |  | |  | |  | | 14,00 | |  | | 2,00 | |  | |  | | 82,00 | |
| **184.2** |  | |  | |  | |  | |  | |  | |  | |  | |  | | 12,00 | | 3,00 | |  | | 9,00 | | 0,00 | |  | |  | |  | |  | |  | | 1,19 | |  | | |  |  | |  | | |  | |  | |  | |  | |  | |  | |  | |  | | 12,00 | |  | | 0,00 | |  | |  | | 65,00 | |
| **184.3** |  | |  | |  | |  | |  | |  | |  | |  | |  | | 4,00 | | 1,00 | |  | | 2,00 | | 0,00 | |  | |  | |  | |  | |  | | 1,03 | |  | | |  |  | |  | | |  | |  | |  | |  | |  | |  | |  | |  | | 4,00 | |  | | 0,00 | |  | |  | | 17,00 | |
| **185** |  | |  | |  | |  | |  | |  | |  | |  | |  | | 42,00 | |  | | 10,00 | |  | |  | |  | |  | |  | |  | |  | |  | |  | | |  |  | |  | | |  | |  | |  | |  | |  | |  | |  | |  | |  | |  | |  | |  | |  | |  | |
| **186.1** |  | |  | |  | |  | |  | |  | |  | |  | |  | | 51,00 | | 3,00 | |  | | 31,00 | | 7,00 | |  | |  | |  | |  | |  | | 1,23 | |  | | |  |  | |  | | |  | |  | |  | |  | |  | |  | |  | |  | | 26,00 | |  | |  | |  | |  | | 174,00 | |
| **186.2** |  | |  | |  | |  | |  | |  | |  | |  | |  | | 38,00 | | 6,00 | |  | | 16,00 | | 4,00 | |  | |  | |  | |  | |  | | 1,98 | |  | | |  |  | |  | | |  | |  | |  | |  | |  | |  | |  | |  | | 39,00 | |  | |  | |  | |  | | 118,00 | |
| **187 .1** |  | |  | |  | |  | |  | |  | |  | |  | | 105,00 | | 409,00 | | 45,00 | | 119,00 | | 71,00 | |  | | 175,00 | | 242,00 | |  | |  | |  | | 3,15 | |  | | |  |  | |  | | |  | |  | |  | |  | |  | |  | |  | |  | |  | |  | |  | |  | |  | |  | |
| **187.2** |  | |  | |  | |  | |  | |  | |  | |  | | 113,00 | | 428,00 | | 52,00 | | 140,00 | | 101,00 | |  | | 161,00 | | 252,00 | |  | |  | |  | | 2,34 | |  | | |  |  | |  | | |  | |  | |  | |  | |  | |  | |  | |  | |  | |  | |  | |  | |  | |  | |
| **188** |  | | 57,00 | |  | |  | |  | |  | |  | | 3,00 | | 4,00 | | 41,00 | | 7,00 | | 9,00 | |  | | 5,00 | |  | |  | | 22,00 | |  | |  | | 2,31 | |  | | |  |  | |  | | | 0,97 | |  | |  | |  | |  | |  | |  | |  | |  | |  | |  | |  | |  | |  | |
| **189.1** |  | | 53,00 | |  | |  | |  | |  | |  | |  | | 3,00 | | 14,00 | | 1,00 | | 11,00 | |  | | 5,00 | |  | | 21,00 | | 2,00 | |  | |  | | 0,93 | |  | | |  |  | |  | | |  | |  | |  | |  | |  | |  | |  | |  | |  | |  | |  | |  | |  | |  | |
| **189 .2** |  | | 59,00 | |  | |  | |  | |  | |  | |  | | 6,00 | | 30,00 | | 5,00 | | 3,00 | |  | | 1,00 | |  | | 13,00 | | 2,00 | |  | |  | | 0,88 | |  | | |  |  | |  | | |  | |  | |  | |  | |  | |  | |  | |  | |  | |  | |  | |  | |  | |  | |
| **190.1** |  | |  | |  | |  | |  | |  | |  | | 1,00 | | 2,00 | | 35,00 | |  | | 2,00 | | 10,00 | | 5,00 | |  | |  | |  | |  | |  | |  | |  | | |  |  | |  | | |  | |  | |  | |  | |  | |  | |  | |  | |  | |  | |  | |  | |  | |  | |
| **190 .2** |  | |  | |  | |  | |  | |  | |  | | 1,00 | | 5,00 | | 62,00 | |  | | 4,00 | | 11,00 | | 9,00 | |  | |  | |  | |  | |  | |  | |  | | |  |  | |  | | |  | |  | |  | |  | |  | |  | |  | |  | |  | |  | |  | |  | |  | |  | |
| **191.1** |  | |  | |  | |  | |  | |  | |  | |  | | 1433,00 | |  | | 3472,00 | |  | |  | | 7443,00 | |  | |  | |  | |  | |  | |  | |  | | |  |  | |  | | |  | |  | |  | |  | |  | |  | |  | |  | |  | |  | | 9909,00 | | 6284,00 | |  | |  | |
| **191.2** |  | |  | |  | |  | |  | |  | |  | |  | | 241,00 | |  | | 649,00 | |  | |  | | 1485,00 | |  | |  | |  | |  | |  | |  | |  | | |  |  | |  | | |  | |  | |  | |  | |  | |  | |  | |  | |  | |  | | 1731,00 | | 1462,00 | |  | |  | |
| **191.3** |  | |  | |  | |  | |  | |  | |  | |  | | 96,00 | |  | | 172,00 | |  | |  | | 478,00 | |  | |  | |  | |  | |  | |  | |  | | |  |  | |  | | |  | |  | |  | |  | |  | |  | |  | |  | |  | |  | | 608,00 | | 721,00 | |  | |  | |
| **192.1** |  | |  | |  | |  | |  | |  | |  | |  | |  | |  | |  | |  | |  | |  | |  | |  | |  | |  | |  | |  | |  | | |  |  | |  | | |  | |  | |  | |  | |  | |  | |  | |  | |  | |  | |  | |  | |  | |  | |
| **192.2** |  | |  | |  | |  | |  | |  | |  | |  | |  | |  | |  | |  | |  | |  | |  | |  | |  | |  | |  | |  | |  | | |  |  | |  | | |  | |  | |  | |  | |  | |  | |  | |  | |  | |  | |  | |  | |  | |  | |
| **193.1** |  | |  | |  | |  | |  | |  | |  | |  | | 40,00 | | 142,00 | | 17,00 | | 40,00 | | 23,00 | | 18,00 | | 33,00 | | 98,00 | | 24,00 | |  | |  | | 1,10 | |  | | | 13,30 |  | |  | | |  | |  | |  | |  | |  | |  | |  | |  | |  | |  | |  | |  | |  | |  | |
| **193.2** |  | |  | |  | |  | |  | |  | |  | |  | | 125,00 | | 281,00 | | 39,00 | | 72,00 | | 74,00 | | 24,00 | | 64,00 | | 189,00 | | 67,00 | |  | |  | | 1,30 | |  | | | 13,10 |  | |  | | |  | |  | |  | |  | |  | |  | |  | |  | |  | |  | |  | |  | |  | |  | |
| **194.1** |  | |  | |  | |  | |  | |  | |  | |  | |  | | 32,00 | |  | | 7,00 | |  | |  | |  | | 12,00 | | 6,00 | |  | |  | | 3,99 | |  | | |  |  | |  | | |  | |  | |  | |  | |  | |  | |  | |  | |  | |  | | 4,00 | |  | |  | |  | |
| **194.2** |  | |  | |  | |  | |  | |  | |  | |  | |  | | 35,00 | |  | | 2,00 | |  | |  | |  | | 12,00 | | 2,00 | |  | |  | | 1,67 | |  | | |  |  | |  | | |  | |  | |  | |  | |  | |  | |  | |  | |  | |  | | 1,00 | |  | |  | |  | |
| **195.1** |  | |  | |  | |  | |  | |  | |  | | 3,00 | |  | |  | | 30,00 | | 26,00 | |  | |  | |  | |  | |  | |  | |  | |  | | 2,90 | | |  |  | |  | | | 3,60 | |  | |  | |  | |  | |  | |  | |  | |  | |  | |  | |  | |  | |  | |
| **195.2** |  | |  | |  | |  | |  | |  | |  | | 0,00 | |  | |  | | 8,00 | | 9,00 | |  | |  | |  | |  | |  | |  | |  | |  | | 5,50 | | |  |  | |  | | |  | |  | |  | |  | |  | |  | |  | |  | |  | |  | |  | |  | |  | |  | |
| **196** |  | |  | |  | |  | |  | |  | |  | |  | |  | | 51,00 | | 0,00 | | 24,00 | |  | |  | |  | | 35,00 | |  | |  | | 4,00 | | 3,30 | |  | | |  |  | |  | | | 1,00 | |  | |  | |  | |  | |  | |  | |  | |  | |  | | 4,00 | | 11,00 | |  | | 78,00 | |
| **197.1** |  | |  | |  | |  | |  | |  | |  | |  | | 5,00 | |  | | 3,00 | | 6,00 | |  | |  | |  | |  | |  | |  | |  | | 9,70 | |  | | |  |  | |  | | |  | |  | |  | | 986,50 | | 38.4 | |  | |  | |  | |  | |  | |  | |  | |  | |  | |
| **197.2** |  | |  | |  | |  | |  | |  | |  | |  | | 2,00 | |  | | 3,00 | | 5,00 | |  | |  | |  | |  | |  | |  | |  | | 3,90 | |  | | |  |  | |  | | |  | |  | |  | | 1325,50 | | 45,80 | |  | |  | |  | |  | |  | |  | |  | |  | |  | |
| **198.1** |  | |  | |  | |  | |  | |  | |  | |  | |  | |  | | 10,00 | | 85,00 | | 15,00 | |  | |  | |  | |  | |  | |  | | 1,70 | |  | | |  |  | |  | | | 0,88 | |  | |  | |  | |  | |  | |  | |  | |  | |  | |  | |  | |  | |  | |
| **198.2** |  | |  | |  | |  | |  | |  | |  | |  | |  | |  | | 7,00 | | 92,00 | | 16,00 | |  | |  | |  | |  | |  | |  | | 1,50 | |  | | |  |  | |  | | | 0,88 | |  | |  | |  | |  | |  | |  | |  | |  | |  | |  | |  | |  | |  | |
| **199.1** |  | |  | |  | |  | |  | |  | |  | |  | |  | |  | |  | |  | |  | |  | |  | |  | |  | |  | |  | | 1,40 | |  | | |  |  | |  | | |  | |  | |  | |  | |  | |  | |  | |  | |  | |  | | 11,00 | | 24,00 | |  | |  | |
| **199.2** |  | |  | |  | |  | |  | |  | |  | |  | |  | |  | |  | |  | |  | |  | |  | |  | |  | |  | |  | | 1,30 | |  | | |  |  | |  | | |  | |  | |  | |  | |  | |  | |  | |  | |  | |  | |  | |  | |  | |  | |
| **199.3** |  | |  | |  | |  | |  | |  | |  | |  | |  | |  | |  | |  | |  | |  | |  | |  | |  | |  | |  | | 1,50 | |  | | |  |  | |  | | |  | |  | |  | |  | |  | |  | |  | |  | |  | |  | |  | |  | |  | |  | |
| **199.4** |  | |  | |  | |  | |  | |  | |  | |  | |  | |  | |  | |  | |  | |  | |  | |  | |  | |  | |  | | 1,90 | |  | | |  |  | |  | | |  | |  | |  | |  | |  | |  | |  | |  | |  | |  | |  | |  | |  | |  | |
| **199.5** |  | |  | |  | |  | |  | |  | |  | |  | |  | |  | |  | |  | |  | |  | |  | |  | |  | |  | |  | | 0,80 | |  | | |  |  | |  | | |  | |  | |  | |  | |  | |  | |  | |  | |  | |  | |  | |  | |  | |  | |
| **200.1** |  | |  | |  | |  | |  | |  | |  | |  | | 2,00 | | 9,00 | | 2,00 | | 3,00 | | 3,00 | | 4,00 | |  | | 4,00 | | 2,00 | |  | |  | |  | |  | | | 11,00 |  | |  | | | 0,99 | |  | |  | |  | |  | |  | |  | |  | |  | |  | |  | |  | |  | |  | |
| **200.2** |  | |  | |  | |  | |  | |  | |  | |  | | 2,00 | | 43,00 | | 14,00 | | 9,00 | | 3,00 | | 7,00 | |  | | 24,00 | | 20,00 | |  | |  | |  | |  | | | 11,60 |  | |  | | | 0,77 | |  | |  | |  | |  | |  | |  | |  | |  | |  | |  | |  | |  | |  | |
| **201** |  | | 55,00 | |  | |  | |  | |  | |  | | 1,20 | | 0,90 | | 48,80 | | 12,30 | | 6,40 | | 6,90 | | 5,90 | | 9,00 | | 8,80 | |  | |  | |  | |  | | 7,30 | | |  |  | |  | | |  | |  | |  | |  | |  | |  | |  | |  | |  | |  | | 16,00 | |  | |  | | 399,00 | |
| **202** |  | | 59,13 | |  | |  | |  | |  | |  | |  | |  | |  | |  | |  | |  | |  | |  | |  | |  | |  | |  | |  | |  | | |  |  | |  | | |  | |  | |  | |  | |  | |  | |  | |  | |  | |  | |  | |  | |  | |  | |
| **203** |  | |  | |  | |  | |  | |  | |  | |  | |  | | 22,00 | |  | | 3,00 | |  | |  | |  | |  | |  | |  | |  | |  | | 2,71 | | |  |  | |  | | |  | |  | |  | |  | |  | |  | |  | |  | |  | |  | |  | |  | |  | |  | |
| **204.1** |  | | 57,00 | |  | |  | |  | |  | |  | |  | |  | | 68,00 | |  | | 6,00 | |  | |  | |  | | 45,00 | |  | |  | |  | | 1,44 | |  | | |  |  | |  | | | 0,89 | |  | |  | |  | |  | |  | |  | | 69,00 | | 19,00 | |  | |  | |  | |  | |  | |
| **204.2** |  | | 59,00 | |  | |  | |  | |  | |  | |  | |  | | 71,00 | |  | | 12,00 | |  | |  | |  | | 43,00 | |  | |  | |  | | 0,95 | |  | | |  |  | |  | | | 0,83 | |  | |  | |  | |  | |  | |  | | 69,00 | | 1,00 | |  | |  | |  | |  | |  | |
| **205 .1** |  | |  | |  | |  | |  | |  | |  | |  | |  | | 41,98 | | 9,02 | | 9,98 | | 12,03 | |  | | 6,98 | | 16,00 | |  | |  | |  | |  | | 2,80 | | |  |  | |  | | | 0,90 | |  | |  | |  | |  | |  | |  | |  | |  | |  | | 4,03 | |  | |  | | 62,98 | |
| **205.2** |  | |  | |  | |  | |  | |  | |  | |  | | 3,79 | | 416,14 | | 88,69 | | 50,03 | | 50,79 | |  | | 107,64 | | 159,18 | |  | |  | |  | |  | | 3,10 | | |  |  | |  | | | 0,90 | |  | |  | |  | |  | |  | |  | |  | |  | |  | | 12,13 | |  | |  | | 63,00 | |
| **206** |  | |  | |  | |  | |  | |  | |  | |  | |  | | 71,00 | | 17,00 | | 15,00 | |  | | 2,00 | | 18,00 | | 40,00 | | 22,00 | | 17,00 | |  | |  | | 2,72 | | | 13,80 |  | |  | | |  | |  | |  | |  | |  | |  | |  | |  | |  | |  | |  | |  | |  | |  | |
| **207** |  | | 35,00 | |  | |  | |  | |  | |  | |  | |  | | 6449,94 | | 1965,70 | | 1412,84 | |  | |  | |  | |  | | 4402,34 | |  | |  | |  | |  | | |  |  | |  | | |  | |  | |  | |  | |  | |  | |  | |  | |  | |  | |  | | 1013,56 | |  | | 7432,79 | |
| **208** |  | |  | |  | |  | |  | |  | |  | |  | | 66,00 | | 1455,00 | | 191,00 | | 180,00 | | 127,00 | | 19,00 | |  | | 839,00 | |  | |  | |  | | 2,30 | |  | | |  |  | |  | | |  | |  | |  | |  | |  | |  | |  | |  | |  | |  | |  | |  | |  | |  | |
| **209.1** |  | |  | |  | |  | |  | |  | |  | |  | |  | | 16,00 | | 2,00 | | 9,00 | |  | |  | |  | | 7,00 | |  | |  | |  | |  | |  | | |  |  | |  | | |  | |  | |  | |  | |  | |  | |  | |  | |  | |  | | 39,00 | |  | |  | |  | |
| **209.2** |  | |  | |  | |  | |  | |  | |  | |  | |  | | 68,00 | | 32,00 | | 31,00 | |  | |  | |  | | 22,00 | |  | |  | |  | |  | |  | | |  |  | |  | | |  | |  | |  | |  | |  | |  | |  | |  | |  | |  | |  | | 109,00 | |  | |  | |
| **209.3** |  | |  | |  | |  | |  | |  | |  | |  | |  | | 113,00 | | 58,00 | | 54,00 | |  | |  | |  | | 43,00 | |  | |  | |  | |  | |  | | |  |  | |  | | |  | |  | |  | |  | |  | |  | |  | |  | |  | |  | |  | |  | | 160,00 | |  | |
| **209.4** |  | |  | |  | |  | |  | |  | |  | |  | |  | | 453,00 | | 167,00 | | 123,00 | |  | |  | |  | | 133,00 | |  | |  | |  | |  | |  | | |  |  | |  | | |  | |  | |  | |  | |  | |  | |  | |  | |  | |  | |  | |  | |  | | 753,00 | |
| **209.5** |  | |  | |  | |  | |  | |  | |  | |  | |  | | 112,00 | | 61,00 | | 39,00 | |  | |  | |  | | 51,00 | |  | |  | |  | |  | |  | | |  |  | |  | | |  | |  | |  | |  | |  | |  | |  | |  | |  | |  | |  | |  | |  | |  | |
| **210** |  | | 63,50 | |  | |  | |  | |  | |  | |  | | 0,00 | | 7,00 | |  | | 5,00 | |  | |  | |  | | 7,00 | |  | |  | |  | |  | |  | | |  |  | |  | | | 1,11 | |  | |  | |  | |  | |  | |  | |  | |  | |  | |  | |  | |  | |  | |
| **211.1** |  | |  | |  | |  | |  | |  | |  | | 5,00 | |  | |  | |  | |  | |  | |  | |  | |  | |  | |  | |  | |  | |  | | |  |  | |  | | | 0,75 | |  | |  | |  | |  | |  | |  | |  | |  | |  | |  | |  | |  | |  | |
| **211.2** |  | |  | |  | |  | |  | |  | |  | | 4,00 | |  | |  | |  | |  | |  | |  | |  | |  | |  | |  | |  | |  | |  | | |  |  | |  | | | 0,77 | |  | |  | |  | |  | |  | |  | |  | |  | |  | |  | |  | |  | |  | |
| **212** |  | |  | |  | |  | |  | |  | |  | |  | |  | |  | |  | |  | | 7,00 | | 2,00 | |  | |  | |  | |  | |  | |  | |  | | |  |  | |  | | |  | |  | |  | |  | |  | |  | |  | |  | |  | |  | | 1,00 | | 27,00 | |  | |  | |
| **213** |  | |  | |  | |  | |  | |  | |  | |  | |  | | 60,00 | | 26,00 | | 56,00 | |  | |  | | 78,00 | |  | |  | |  | |  | | 4,80 | |  | | |  |  | |  | | | 0,77 | |  | |  | |  | |  | |  | |  | |  | |  | |  | |  | |  | |  | |  | |
| **214** |  | |  | |  | |  | |  | |  | |  | |  | | 13,00 | |  | | 7,00 | |  | |  | |  | | 6,00 | |  | |  | |  | | 1,00 | |  | |  | | |  |  | |  | | |  | |  | |  | |  | |  | |  | |  | |  | |  | |  | | 41,00 | |  | |  | |  | |
| **215** |  | |  | |  | |  | |  | |  | |  | | 7,00 | |  | | 127,00 | | 14,00 | | 13,00 | | 23,00 | | 6,00 | |  | |  | |  | |  | |  | |  | |  | | | 13,70 |  | |  | | | 0,90 | |  | |  | |  | |  | |  | |  | |  | |  | |  | |  | |  | |  | |  | |
| **216** |  | |  | |  | |  | |  | |  | |  | |  | |  | |  | |  | |  | |  | |  | |  | |  | |  | |  | |  | |  | |  | | | 13,90 |  | |  | | |  | |  | |  | |  | |  | |  | |  | |  | |  | |  | |  | |  | |  | |  | |
| **217** |  | |  | |  | |  | |  | |  | |  | |  | | 1,00 | | 93,00 | | 9,00 | | 15,00 | |  | | 7,00 | |  | |  | |  | |  | |  | |  | |  | | |  |  | |  | | |  | |  | |  | |  | |  | |  | |  | |  | |  | |  | | 2,00 | |  | |  | |  | |
| **218** |  | |  | |  | |  | |  | |  | |  | | 9,00 | |  | | 94,00 | | 9,00 | | 10,00 | |  | | 8,00 | |  | |  | | 41,00 | |  | |  | |  | |  | | |  |  | |  | | | 0,90 | |  | |  | |  | |  | |  | |  | |  | |  | |  | |  | |  | |  | |  | |
| **219.1** |  | |  | |  | |  | |  | |  | |  | |  | |  | |  | |  | | 2,00 | |  | |  | | 5,00 | | 5,00 | | 2,00 | |  | |  | | 4,50 | |  | | |  |  | |  | | |  | |  | |  | |  | |  | |  | |  | |  | | 2,00 | |  | |  | |  | |  | | 14,00 | |
| **219 .2** |  | |  | |  | |  | |  | |  | |  | |  | |  | |  | |  | | 0,00 | |  | |  | | 2,00 | | 2,00 | | 0,00 | |  | |  | | 5,50 | |  | | |  |  | |  | | |  | |  | |  | |  | |  | |  | |  | |  | | 1,00 | |  | |  | |  | |  | | 8,00 | |
| **220.1** |  | |  | |  | |  | |  | |  | |  | | 5,00 | | 49,00 | | 152,00 | | 30,00 | | 9,00 | | 83,00 | | 14,00 | | 109,00 | | 121,00 | | 16,00 | | 8,00 | |  | | 3,24 | |  | | | 12,80 |  | |  | | |  | |  | |  | |  | |  | |  | |  | |  | |  | |  | | 10,00 | |  | |  | |  | |
| **220.2** |  | |  | |  | |  | |  | |  | |  | | 2,00 | | 29,00 | | 89,00 | | 22,00 | | 6,00 | | 52,00 | | 11,00 | | 57,00 | | 76,00 | | 15,00 | | 6,00 | |  | |  | |  | | |  |  | |  | | |  | |  | |  | |  | |  | |  | |  | |  | |  | |  | | 6,00 | |  | |  | |  | |
| **220.3** |  | |  | |  | |  | |  | |  | |  | | 3,00 | | 17,00 | | 49,00 | | 6,00 | | 3,00 | | 26,00 | | 2,00 | | 42,00 | | 38,00 | | 1,00 | | 2,00 | |  | |  | |  | | |  |  | |  | | |  | |  | |  | |  | |  | |  | |  | |  | |  | |  | | 3,00 | |  | |  | |  | |
| **220.4** |  | |  | |  | |  | |  | |  | |  | | 0,00 | | 3,00 | | 14,00 | | 2,00 | | 1,00 | | 5,00 | | 1,00 | | 10,00 | | 7,00 | | 0,00 | | 0,00 | |  | |  | |  | | |  |  | |  | | |  | |  | |  | |  | |  | |  | |  | |  | |  | |  | | 1,00 | |  | |  | |  | |
| **220.5** |  | |  | |  | |  | |  | |  | |  | | 3,00 | | 38,00 | | 140,00 | | 26,00 | | 10,00 | | 72,00 | | 19,00 | | 101,00 | | 93,00 | | 17,00 | | 16,00 | |  | | 1,81 | |  | | | 13,60 |  | |  | | |  | |  | |  | |  | |  | |  | |  | |  | |  | |  | | 12,00 | |  | |  | |  | |
| **221** |  | |  | |  | |  | |  | |  | |  | |  | |  | |  | |  | |  | |  | |  | |  | |  | |  | |  | |  | |  | |  | | |  |  | |  | | |  | |  | |  | |  | |  | |  | |  | |  | |  | |  | |  | |  | |  | |  | |
| **222.1** |  | |  | |  | |  | |  | |  | |  | |  | |  | |  | | 9,00 | | 7,00 | |  | | 14,00 | |  | |  | |  | |  | |  | | 1,98 | |  | | |  |  | |  | | |  | |  | |  | |  | |  | |  | |  | |  | |  | |  | |  | |  | |  | |  | |
| **222.2** |  | |  | |  | |  | |  | |  | |  | |  | |  | |  | | 4,00 | | 4,00 | |  | | 8,00 | |  | |  | |  | |  | |  | | 1,43 | |  | | |  |  | |  | | |  | |  | |  | |  | |  | |  | |  | |  | |  | |  | |  | |  | |  | |  | |
| **223** |  | | 48,40 | |  | |  | |  | |  | |  | |  | |  | | 8,00 | | 8,00 | | 7,00 | |  | |  | |  | |  | |  | |  | |  | | 9,10 | |  | | |  |  | |  | | |  | |  | |  | |  | |  | |  | |  | |  | |  | |  | |  | | 14,00 | |  | | 6,00 | |
| **224** |  | | 57,60 | |  | |  | |  | |  | |  | |  | | 0,00 | | 4,00 | | 0,00 | |  | |  | | 0,00 | |  | |  | |  | |  | |  | |  | |  | | |  |  | |  | | |  | |  | |  | |  | |  | |  | |  | |  | |  | |  | |  | | 5,00 | |  | | 6,00 | |
| **225.1** |  | |  | |  | |  | |  | |  | |  | |  | |  | |  | |  | | 6,00 | |  | |  | |  | |  | |  | |  | |  | | 0,26 | |  | | |  |  | |  | | | 1,04 | |  | |  | |  | |  | |  | |  | |  | |  | |  | |  | |  | |  | |  | |
| **225.2** |  | |  | |  | |  | |  | |  | |  | |  | |  | |  | |  | | 5,00 | |  | |  | |  | |  | |  | |  | |  | | 1,97 | |  | | |  |  | |  | | | 0,91 | |  | |  | |  | |  | |  | |  | |  | |  | |  | |  | |  | |  | |  | |
| **Ʃ** | 1,50 | | 2320,40 | | 257,70 | | 28,00 | | 1,00 | | 1,00 | | 41,00 | | 1038,20 | | 7749,69 | | 45638,57 | | 11829,71 | | 9279,26 | | 5005,72 | | 12130,90 | | 12719,61 | | 13401,28 | | 8842,84 | | 168,00 | | 13,00 | |  | |  | | | 314,59 | 63,88 | | 124,40 | | | 56,32 | | 1,30 | | 0,00 | | 2317,47 | |  | | 14,50 | | 0,00 | | 439,00 | | 1099,00 | | 68,00 | | 13822,16 | | 10789,56 | | 2046,00 | | 20287,76 | |
| **mean** | 0,50 | | 55,25 | |  | |  | |  | |  | |  | | 39,93 | | 82,44 | | 288,85 | | 71,70 | | 49,68 | | 59,59 | | 120,11 | | 231,27 | | 174,04 | | 121,13 | | 9,33 | | 4,33 | | 2,48 | | 4,83 | | | 13,11 | 21,29 | | 41,47 | | | 0,97 | | 1,30 | |  | | 386,25 | | 29,55 | | 14,50 | |  | | 87,80 | | 39,25 | | 5,67 | | 170,64 | | 250,92 | | 341,00 | | 322,03 | |
| **SD** | 0,00 | | 4,73 | |  | |  | |  | |  | |  | | 142,19 | | 424,98 | | 1057,08 | | 321,53 | | 170,45 | | 154,28 | | 752,01 | | 550,38 | | 477,33 | | 522,54 | | 14,46 | | 2,87 | | 2,01 | | 3,73 | | | 0,95 | 0,98 | | 2,00 | | | 0,36 | | 0,00 | |  | | 553,03 | | 16,25 | | 0,00 | |  | | 15,54 | | 83,51 | | 5,92 | | 1107,81 | | 973,98 | | 433,37 | | 1064,56 | |
| **missings** | |  | |  | |  | |  | |  | |  | |  | | 90.0 | | 62.0 | | 33.0 | | 19.0 | | 62.0 | | 52.0 | | 80.0 | | 62.0 | | 65.0 | | 90.0 | | 98.0 | | 48.0 | |  | |  |  | | |  | |  |  | |  | |  | |  | |  | |  | |  | |  | |  | |  | |  | |  | |  | |  | |  |

**Table 1.2:** Perioperative data; pIn= periareolar incision, pCan= percutaneous cannulation, endoC= endoaortic clamping, PMLneo= PML neochords, AMLneo= AML neochords, quRes= quadrangular resection, trRes= triangular resection, AnPl= annuloplasty, OT= operative time (min), CPB= cardiopulmonary bypass time (min), AoX= aortic cross-clamp time (min), fA= femoral artery cannulation, rAA= right axillary artery cannulation, fV= femoral vein, rAbi= right atrium bicaval, Bret=Bretschneider (Custodiol), St.T= St.Thomas (Plegisol), ante= antegrade, AlP= Alfieri plasty/edge-to-edge, MVRepl= mitral valve replacement, MVR= mitral valve repair, LAAb= LA ablation, LAAo= LAA occlusion, PFOc= PFO closure, ASDc= ASD closure, Tres= Tumor resection, TVR= tricuspid valve repair, AVR= aortic valve repair, AAR= ascending aortic repair, CtS= conversion to sternotomy, BL= blood loss (ml), secRA= second repair attempt, RBCs/FFP/PLT= x transfused, LCO= low cardiac output, PVL= paravalvular leak, calAML= calcification AML, calAnn= calcification annulus, pPML= prolapsed PML, pAML= prolapsed AML, ChR= chordae rupture, ChE= chordae elongation, AnnD= annulus dilatation, rPap= ruptured papillary muscle, MRVol= mitral regurgitation volume (ml)

|  | **pIn** | **pCan** | **endoC** | **PML neo** | **AML neo** | **PML+AML neo** | **quRes** | **trRes** | **AnPl** | **OT** | **CPB** | **AoX** | **fA** | **rAA** | **fV** | **rAbi** | **SVC** | **Bret** | **St. T** | **ante** | **ante- + retro** | **AlP** | **25,00** | **28,00** | **30,00** | **32,00** | **34,00** | **36,00** |
| --- | --- | --- | --- | --- | --- | --- | --- | --- | --- | --- | --- | --- | --- | --- | --- | --- | --- | --- | --- | --- | --- | --- | --- | --- | --- | --- | --- | --- |
| **1** | 76,00 | 9,00 | 115,00 | 358,00 | 70,00 | 428,00 |  | 10,00 | 91,00 | 150,00 | 101,00 | 66,00 |  |  |  |  |  |  |  |  |  |  |  |  |  |  |  |  |
| **65.03** |  |  |  |  |  |  |  |  |  |  | 78,00 | 57,00 |  |  |  |  |  |  |  |  |  |  |  |  |  |  |  |  |
| **4** |  |  |  |  |  |  |  |  |  |  |  |  |  |  |  |  |  |  |  |  |  |  |  |  |  |  |  |  |
| **5** |  |  |  |  |  | 34,00 |  |  | 49,00 |  | 168,82 | 111,44 |  |  |  |  |  |  |  |  |  | 0,00 |  |  |  |  |  |  |
| **7** |  |  |  |  |  |  |  |  |  |  | 163,80 | 119,70 |  |  |  |  |  |  |  |  |  |  | 1,00 | 10,00 | 32,00 | 40,00 | 12,00 | 1,00 |
| **8** |  |  |  |  |  |  |  |  |  |  | 100,50 | 88,90 |  |  |  |  |  |  |  |  |  |  |  |  |  |  |  |  |
| **9** |  |  |  | 22,00 | ´ | 22,00 |  |  |  | 228,70 |  | 85,60 |  |  |  |  |  |  |  |  |  |  |  |  |  |  |  |  |
| **13** |  |  |  |  |  |  |  |  |  |  | 87,50 | 164,60 |  |  |  |  |  |  |  |  |  |  |  |  |  |  |  |  |
| **14.1** |  |  |  |  |  | 23,00 |  |  | 29,00 |  |  |  |  |  |  |  |  |  |  |  |  |  |  |  |  |  |  |  |
| **14.2** |  |  |  |  |  | 38,00 |  |  | 62,00 |  |  |  |  |  |  |  |  |  |  |  |  |  |  |  |  |  |  |  |
| **14.3** |  |  |  |  |  | 44,00 |  |  | 81,00 |  |  |  |  |  |  |  |  |  |  |  |  |  |  |  |  |  |  |  |
| **15.1** |  |  |  |  |  | 95,00 |  |  | 121,00 | 209,60 | 147,80 | 91,20 |  |  |  |  |  |  |  |  |  |  |  |  |  |  |  |  |
| **15.2** |  |  |  |  |  | 48,00 |  |  | 56,00 | 208,00 | 143,60 | 89,20 |  |  |  |  |  |  |  |  |  |  |  |  |  |  |  |  |
| **18.1** |  |  |  |  |  | 15,00 |  | 21,00 | 46,00 |  |  |  |  |  |  |  |  |  |  |  |  | 35,00 |  |  |  |  |  |  |
| **18.2** |  |  |  | 54,00 |  | 54,00 |  | 0,00 | 59,00 |  |  |  |  |  |  |  |  |  |  |  |  | 7,00 |  |  |  |  |  |  |
| **18.3** |  |  |  | 28,00 |  | 28,00 |  |  | 40,00 |  |  |  |  |  |  |  |  |  |  |  |  | 0,00 |  |  |  |  |  |  |
| **20** |  |  |  |  |  | 536,00 | 71,00 | 111,00 | 1231,00 |  | 118,90 | 83,10 |  |  |  |  |  |  |  |  |  | 85,00 |  |  |  |  |  |  |
| **21** |  |  |  |  |  | 56,00 |  | 32,00 | 127,00 | 219,00 | 144,00 | 113,00 | 98,00 | 31,00 | 115,00 |  | 14,00 |  |  |  |  | 26,00 |  |  |  |  |  |  |
| **26** |  |  |  |  |  | 76,00 |  |  | 100,00 |  | 164,40 | 119,70 |  |  |  |  |  |  |  |  |  |  |  |  |  |  |  |  |
| **29** |  |  |  |  |  |  |  |  | 90,00 | 54,90 | 140,50 | 94,80 |  |  |  |  |  |  |  |  |  |  |  |  |  |  |  |  |
| **30** |  |  | 182,00 |  |  | 60,00 |  |  | 40,00 |  | 145,60 | 104,80 |  |  |  |  |  |  |  |  |  |  |  |  |  |  |  |  |
| **31.1** |  | |  |  |  | 71,00 |  |  |  | 170,00 | 133,00 | 76,00 |  |  |  |  |  |  |  |  |  |  |  |  |  |  |  |  |
| **31.2** |  | |  |  |  |  |  |  |  | 164,00 | 126,00 | 53,00 |  |  |  |  |  |  |  |  |  |  |  |  |  |  |  |  |
| **35** |  |  |  |  |  |  |  |  |  |  |  |  |  |  |  |  |  |  |  |  |  |  |  |  |  |  |  |  |
| **38** |  |  |  |  |  | 24,00 |  | 5,00 | 39,00 |  | 176,10 | 116,30 |  |  |  |  |  |  |  |  |  |  |  |  |  |  |  |  |
| **40** |  |  |  |  |  |  |  |  |  | 250,00 | 158,00 | 84,00 | 25,00 | ´ |  |  |  |  |  |  |  | 6,00 |  |  |  |  |  |  |
| **41** | 109,00 |  | 109,00 | 17,00 | 37,00 | 54,00 |  | 0,00 | 2,00 | 169,50 | 111,50 | 68,50 |  |  |  |  |  | 91,00 |  |  |  |  |  |  |  |  |  |  |
| **42** | 57,00 |  |  |  |  | 39,00 |  |  |  |  | 123,20 | 101,30 |  |  |  |  |  |  |  |  |  |  |  |  | 1,00 | 4,00 | 3,00 | 3,00 |
| **43** |  |  |  |  |  |  |  |  |  | 230,00 | 181,00 | 106,00 |  |  |  |  |  |  |  |  |  |  |  |  |  |  |  |  |
| **44.1** |  |  |  |  |  |  |  |  |  |  | 113,52 | 81,33 |  |  |  |  |  |  |  |  |  |  |  |  |  |  |  |  |
| **44.2** |  |  |  |  |  |  |  |  |  |  | 119,31 | 82,06 |  |  |  |  |  |  |  |  |  |  |  |  |  |  |  |  |
| **45** |  |  |  |  |  |  |  |  |  |  |  |  |  |  |  |  |  |  |  |  |  |  |  |  |  |  |  |  |
| **46** |  | 43,00 | 27,00 | 125,00 | 24,00 | 149,00 |  |  | 28,00 |  | 111,30 | 71,60 | 148,00 | 2,00 |  |  |  |  |  |  |  |  |  |  |  |  |  |  |
| **47** |  |  | 44,00 | 28,00 | 6,00 | 34,00 |  | 2,00 | 8,00 |  | 107,70 | 64,50 |  |  |  |  |  |  |  |  |  |  |  |  |  |  |  |  |
| **48** |  |  |  |  |  |  |  |  |  |  |  |  |  |  |  |  |  |  |  |  |  |  |  |  |  |  |  |  |
| **51** |  |  |  |  |  |  |  |  |  |  | 117,64 | 98,11 |  |  |  |  |  |  |  |  |  |  |  |  |  |  |  |  |
| **53.1** | 309,00 |  | 27,00 | 37,00 | 9,00 | 46,00 |  |  | 49,00 |  | 126,00 | 93,00 |  |  |  |  |  | 20,00 | 280,00 |  |  | 6,00 |  |  |  |  |  |  |
| **53.2** |  | |  |  |  |  |  |  |  |  | 126,00 | 94,00 |  |  |  |  |  |  |  |  |  |  |  |  |  |  |  | 5,00 |
| **53.3** |  | |  |  |  |  |  |  |  |  | 122,00 | 90,00 |  |  |  |  |  |  |  |  |  |  |  |  |  |  |  | 9,00 |
| **55.1** |  |  |  |  |  |  |  |  |  |  | 135,86 | 92,46 |  |  |  |  |  |  |  |  |  |  |  |  |  |  |  |  |
| **55.2** |  |  |  |  |  |  |  |  |  |  | 134,71 | 85,32 |  |  |  |  |  |  |  |  |  |  |  |  |  |  |  |  |
| **56.1** |  | |  | 15,00 |  |  |  |  |  | 320,00 | 139,70 | 90,30 |  |  |  |  |  |  |  |  |  |  |  |  |  |  |  |  |
| **56.2** |  |  |  | 24,00 |  |  |  |  |  | 322,00 | 141,20 | 94,30 |  |  |  |  |  |  |  |  |  |  |  |  |  |  |  |  |
| **57** |  |  |  |  |  |  |  |  |  | 120,00 |  |  |  |  |  |  |  |  |  |  |  |  |  |  |  |  |  |  |
| **63.1** |  |  |  |  |  |  |  |  |  |  | 82,00 | 62,00 |  |  |  |  |  |  |  |  |  |  |  |  |  |  |  |  |
| **63.2** |  |  |  |  |  |  |  |  |  |  | 74,00 | 55,00 |  |  |  |  |  |  |  |  |  |  |  |  |  |  |  |  |
| **65** |  |  |  | 62,00 |  |  |  |  | 226,00 |  | 93,50 | 71,60 |  |  |  |  |  |  |  |  |  |  |  |  |  |  |  |  |
| **66** | 637,00 |  | 1095,00 |  |  |  |  |  |  |  | 135,00 | 85,00 |  |  |  |  |  | 3973,00 |  |  |  |  |  |  |  |  |  |  |
| **67** |  |  |  |  |  |  |  |  |  | 4,80 |  | 95,00 |  |  |  |  |  |  |  |  |  |  |  |  |  |  |  |  |
| **68** |  |  |  | 15,00 |  |  |  |  | 41,00 |  | 142,90 | 89,90 |  |  |  |  |  |  |  |  |  |  |  |  |  |  |  |  |
| **71** |  |  |  |  |  |  |  |  |  |  |  |  |  |  |  |  |  |  |  |  |  |  |  |  |  |  |  |  |
| **72** |  |  |  |  |  |  |  |  |  |  |  |  |  |  |  |  |  |  |  |  |  |  |  |  |  |  |  |  |
| **73** |  |  |  |  |  |  |  |  |  |  |  |  |  |  |  |  |  |  |  |  |  |  |  |  |  |  |  |  |
| **74** |  |  |  | 266,00 |  |  |  |  | 1,00 |  | 161,00 | 94,00 |  |  |  |  |  |  |  |  |  |  |  |  |  |  |  |  |
| **75** |  |  |  | 1,00 |  |  |  |  | 5,00 |  | 88,00 | 47,00 |  |  |  |  |  |  |  |  |  |  |  |  |  |  |  |  |
| **77** |  |  |  | 26,00 | ´ |  |  |  |  | 291,00 | 155,00 | 101,00 |  |  |  |  |  |  |  |  |  |  |  |  |  |  |  |  |
| **78.1** |  | | 0,00 | 88,00 |  |  |  |  | 100,00 |  | 173,00 | 121,00 |  |  |  |  |  |  |  |  |  |  |  |  |  |  |  |  |
| **78.2** |  |  | 0,00 | 121,00 |  |  |  |  | 145,00 |  | 146,00 | 104,00 |  |  |  |  |  |  |  |  |  |  |  |  |  |  |  |  |
| **79** |  |  |  |  |  |  |  |  | 67,00 |  | 173,00 | 87,00 |  |  |  |  |  |  |  |  |  |  |  |  |  |  |  |  |
| **80** |  |  |  |  |  |  |  |  |  |  | 198,00 | 106,00 |  |  |  |  |  |  |  |  |  |  |  |  |  |  |  |  |
| **82** |  |  |  |  |  |  |  |  | 24,00 | 274,00 | 174,00 | 119,00 | 342,00 | 5,00 |  |  |  |  |  |  |  |  |  |  |  |  |  |  |
| **83** |  |  |  |  |  |  |  |  |  |  | 84,00 | 61,00 |  |  |  |  |  |  |  |  |  |  |  |  |  |  |  |  |
| **86** |  |  |  | 12,00 | 14,00 | 14,00 |  | 9,00 | 1,00 | 302,00 | 212,00 | 150,00 |  |  |  |  |  |  |  |  |  |  |  |  |  |  |  |  |
| **87.1** |  |  |  |  |  |  |  |  |  | 186,00 |  |  |  |  |  |  |  |  |  |  |  |  |  |  |  |  |  |  |
| **87.2** |  |  |  |  |  |  |  |  |  | 186,00 | 1,00 |  |  |  |  |  |  |  |  |  |  |  |  |  |  |  |  |  |
| **93** |  |  |  | 64,00 |  |  |  | 3,00 | 90,00 | 296,00 | 177,10 | 114,00 |  |  |  |  |  |  |  |  |  | 1,00 |  |  |  |  |  |  |
| **98** |  |  |  |  |  |  |  |  |  |  | 153,70 | 106,80 |  |  |  |  |  |  |  |  |  |  |  |  |  |  |  |  |
| **99.1** |  |  |  |  |  |  |  |  |  |  |  |  |  |  |  |  |  |  |  |  |  |  |  |  |  |  |  |  |
| **99.2** |  |  |  |  |  |  |  |  |  |  |  |  |  |  |  |  |  |  |  |  |  |  |  |  |  |  |  |  |
| **101.1** |  |  |  |  |  |  |  |  |  |  | 131,00 |  |  |  |  |  |  |  |  |  |  |  |  |  |  |  |  |  |
| **101.2** |  |  |  |  |  |  |  |  |  |  | 137,00 |  |  |  |  |  |  |  |  |  |  |  |  |  |  |  |  |  |
| **102** | 18,00 |  |  |  |  |  |  |  |  |  | 105,00 | 68,00 |  |  |  |  |  |  |  |  |  |  |  |  |  |  |  |  |
| **103** |  |  | 5,00 |  |  |  |  |  |  |  | 178,00 | 108,00 | 218,00 |  |  |  |  |  |  |  |  |  |  |  |  |  |  |  |
| **104** |  |  |  |  |  |  |  |  |  | 220,50 | 149,94 | 95,01 |  |  |  |  |  | 72,90 | 5,20 |  |  |  |  |  |  |  |  |  |
| **105** |  |  |  |  |  |  |  |  |  |  | 166,00 |  |  |  |  |  |  |  |  |  |  |  |  |  |  |  |  |  |
| **107** |  |  |  |  |  |  |  |  |  | 240,00 | 112,20 | 79,40 |  |  |  |  |  |  |  |  |  |  |  |  |  |  |  |  |
| **108** |  |  |  |  |  |  |  |  |  | 179,00 | 135,00 | 105,00 |  |  |  |  |  |  |  |  |  |  |  |  |  |  |  |  |
| **110** |  |  |  | 123,00 |  |  |  |  |  |  | 103,00 | 62,00 |  |  |  |  |  |  |  |  |  | 20,00 |  |  |  |  |  |  |
| **111** |  |  |  | 190,00 |  |  |  |  |  |  | 99,00 | 65,00 |  |  |  |  |  |  |  |  |  | 31,00 |  |  |  |  |  |  |
| **112** |  |  |  | 83,00 | 1,00 | 1,00 |  |  |  |  |  |  |  |  |  |  |  |  |  |  |  | 4,00 |  |  |  |  |  |  |
| **113.1** |  | |  |  |  |  |  |  |  |  | 200,00 | 134,00 |  |  |  |  |  |  |  |  |  |  |  |  |  |  |  |  |
| **113.2** |  |  |  |  |  |  |  |  |  |  | 165,00 | 111,00 |  |  |  |  |  |  |  |  |  |  |  |  |  |  |  |  |
| **115.1** |  |  |  | 232,00 | 55,00 | 55,00 |  |  |  |  | 199,95 | 109,70 | 470,00 | 10,00 | 405,00 |  |  | 105,00 | 369,00 |  |  |  |  |  |  |  |  |  |
| **115.2** |  |  |  | 176,00 | 37,00 | 37,00 |  |  |  |  | 195,50 | 106,39 | 339,00 | 5,00 | 292,00 |  |  | 98,00 | 241,00 |  |  |  |  |  |  |  |  |  |
| **115.3** |  |  |  | 117,00 | 26,00 | 26,00 |  |  |  |  | 188,48 | 102,67 | 215,00 | 3,00 | 181,00 |  |  | 84,00 | 133,00 |  |  |  |  |  |  |  |  |  |
| **116.1** |  | | 92,00 |  |  |  |  |  |  |  | 137,00 | 101,00 |  |  |  |  |  |  |  |  |  |  |  |  |  |  |  |  |
| **116.2** |  |  | 90,00 |  |  |  |  |  |  |  | 169,00 | 122,00 |  |  |  |  |  |  |  |  |  |  |  |  |  |  |  |  |
| **117.1** |  | |  |  |  |  |  |  | 35,00 | 210,31 | 129,00 | 86,71 |  |  |  |  |  |  |  |  |  |  |  |  |  |  |  |  |
| **117.2** |  | |  |  |  |  |  |  | 32,00 | 233,90 | 170,80 | 111,63 |  |  |  |  |  |  |  |  |  |  |  |  |  |  |  |  |
| **118** |  |  |  | 282,00 |  |  |  |  |  |  | 122,00 | 82,00 |  |  |  |  |  |  |  |  |  |  |  |  |  |  |  |  |
| **119.1** |  |  |  |  |  |  |  |  |  |  |  |  |  |  |  |  |  |  |  |  |  |  |  |  |  |  |  |  |
| **119.2** |  |  |  |  |  |  |  |  |  |  |  |  |  |  |  |  |  |  |  |  |  |  |  |  |  |  |  |  |
| **121.1** |  |  |  |  |  |  |  |  |  |  |  | 100,00 |  |  |  |  |  | 69,00 |  |  |  |  |  |  |  |  |  |  |
| **121.2** |  |  |  |  |  |  |  |  |  |  |  | 94,00 |  |  |  |  |  | 33,00 |  |  |  |  |  |  |  |  |  |  |
| **122.1** |  |  |  |  |  |  |  |  |  |  | 102,00 | 65,00 |  |  |  |  |  |  |  |  |  |  |  |  |  |  |  |  |
| **122.2** |  | |  |  |  |  |  |  |  |  | 93,00 | 58,00 |  |  |  |  |  |  |  |  |  |  |  |  |  |  |  |  |
| **123.1** |  |  |  |  |  | 50,00 |  |  | 64,00 |  | 122,00 | 75,00 |  |  |  |  |  |  |  | 30,00 | 35,00 |  |  |  |  |  |  |  |
| **123.2** |  |  |  |  |  | 34,00 |  |  | 38,00 |  | 139,00 | 84,00 |  |  |  |  |  |  |  | 15,00 | 27,00 |  |  |  |  |  |  |  |
| **127** |  |  |  |  |  |  |  |  |  |  | 88,60 | 56,30 |  |  |  |  |  |  |  |  |  |  |  |  |  |  |  |  |
| **128** |  |  |  |  |  |  |  |  |  |  | 149,90 | 94,80 | 113,00 |  |  |  |  |  |  |  |  |  |  |  |  |  |  |  |
| **129** |  |  |  |  |  | 52,00 | 13,00 |  |  | 325,00 | 222,00 | 142,00 |  |  |  |  |  |  |  |  |  | 1,00 |  | 7,00 | 20,00 | 21,00 | 32,00 | 28,00 |
| **130** |  |  |  |  |  | 153,00 | 58,00 |  | 35,00 |  |  | 117,00 |  |  |  |  |  |  |  |  |  |  |  |  |  |  |  |  |
| **131.1** |  |  | 40,00 |  |  |  |  |  |  |  | 139,20 | 102,68 | 116,00 | 2,00 |  |  |  |  |  |  |  |  |  |  |  |  |  |  |
| **131.2** |  |  | 6,00 |  |  |  |  |  |  |  | 125,00 | 89,42 | 103,00 | 11,00 |  |  |  |  |  |  |  |  |  |  |  |  |  |  |
| **132 .1** |  |  |  |  |  |  |  |  |  |  | 153,50 | 112,00 |  |  |  |  |  |  |  |  |  |  |  |  |  |  |  |  |
| **132 .2** |  |  |  |  |  |  |  |  |  |  | 116,00 | 75,00 |  |  |  |  |  |  |  |  |  |  |  |  |  |  |  |  |
| **133** |  |  |  |  |  |  |  |  |  | 278,00 | 129,00 | 90,00 |  |  |  |  |  |  |  |  |  |  |  |  |  |  |  |  |
| **134.1** |  |  |  |  |  |  |  |  |  | 240,00 | 152,00 | 95,00 |  |  |  |  |  |  |  |  |  |  |  |  |  |  |  |  |
| **134.2** |  | |  |  |  |  |  |  |  | 229,00 | 137,00 | 86,00 |  |  |  |  |  |  |  |  |  |  |  |  |  |  |  |  |
| **135 .1** |  |  |  |  |  |  |  |  |  |  | 111,00 | 75,00 |  |  |  |  |  |  |  |  |  |  |  |  |  |  |  |  |
| **135.2** |  |  |  |  |  |  |  |  |  |  | 105,00 | 71,00 |  |  |  |  |  |  |  |  |  |  |  |  |  |  |  |  |
| **136.1** |  | |  |  |  |  |  |  |  |  | 104,00 | 81,00 |  |  |  |  |  |  |  |  |  |  |  |  |  |  |  |  |
| **136.2** |  |  |  |  |  |  |  |  |  |  | 104,00 | 74,00 |  |  |  |  |  |  |  |  |  |  |  |  |  |  |  |  |
| **136.3** |  | |  |  |  |  |  |  |  |  | 134,00 | 100,00 |  |  |  |  |  |  |  |  |  |  |  |  |  |  |  |  |
| **136.4** |  |  |  |  |  |  |  |  |  |  | 120,00 | 93,00 |  |  |  |  |  |  |  |  |  |  |  |  |  |  |  |  |
| **136.5** |  | |  |  |  |  |  |  |  |  | 159,00 | 116,00 |  |  |  |  |  |  |  |  |  |  |  |  |  |  |  |  |
| **137** |  |  |  |  |  | 7,00 |  |  |  | 297,00 | 187,00 | 126,00 |  |  |  |  |  |  |  |  |  |  |  |  |  |  |  |  |
| **138** |  |  |  |  |  |  |  |  |  |  | 118,00 | 87,00 |  |  |  |  |  |  |  |  |  |  |  |  |  |  |  |  |
| **139.1** |  |  |  |  |  | 15,00 | 1,00 |  | 21,00 |  | 152,00 | 86,00 | 28,00 | 1,00 |  |  |  |  |  |  |  | 0,00 |  |  |  |  |  |  |
| **139.2** |  |  |  |  |  | 31,00 | 12,00 |  | 51,00 |  | 152,00 | 99,00 | 72,00 | 2,00 |  |  |  |  |  |  |  | 0,00 |  |  |  |  |  |  |
| **139.3** |  |  |  |  |  | 874,00 | 36,00 |  | 1098,00 |  | 149,00 | 95,00 | 1177,00 | 13,00 |  |  |  |  |  |  |  | 54,00 |  |  |  |  |  |  |
| **139.4** |  |  |  |  |  | 2075,00 | 576,00 |  | 2856,00 |  | 133,00 | 83,00 | 2902,00 | 30,00 |  |  |  |  |  |  |  | 39,00 |  |  |  |  |  |  |
| **140** |  |  |  |  |  |  |  |  |  |  |  |  |  |  |  |  |  |  |  |  |  |  |  |  |  |  |  |  |
| **141** |  |  |  |  |  |  |  |  |  |  | 167,00 | 105,00 |  |  |  |  |  |  |  |  |  |  |  |  |  |  |  |  |
| **142.1** |  |  |  |  |  |  |  |  |  | 282,00 | 122,00 |  |  |  |  |  |  |  |  |  |  |  |  |  |  |  |  |  |
| **142.2** |  |  |  |  |  |  |  |  |  | 327,00 | 143,00 |  |  |  |  |  |  |  |  |  |  |  |  |  |  |  |  |  |
| **143.1** |  |  |  |  |  |  | 1,00 |  |  | 235,00 | 154,00 |  |  |  |  |  |  |  |  |  |  |  |  |  |  |  |  |  |
| **143.2** |  |  |  |  |  |  | 40,00 |  |  | 205,00 | 137,00 |  |  |  |  |  |  |  |  |  |  |  |  |  |  |  |  |  |
| **144.1** |  |  |  |  |  |  |  |  |  | 211,00 | 137,00 | 76,00 |  |  |  |  |  |  |  |  |  |  |  |  |  |  |  |  |
| **144.2** |  |  |  |  |  |  |  |  |  | 205,00 | 136,00 | 77,00 |  |  |  |  |  |  |  |  |  |  |  |  |  |  |  |  |
| **145** |  |  |  |  |  |  |  |  |  |  | 160,20 |  |  |  |  |  |  |  |  |  |  |  |  |  |  |  |  |  |
| **146** |  |  |  |  |  |  |  |  |  |  | 188,00 |  |  |  |  |  |  |  |  |  |  |  |  |  |  |  |  |  |
| **147** |  |  |  |  |  |  |  |  |  |  |  |  |  |  |  |  |  |  |  |  |  |  |  |  |  |  |  |  |
| **148 .1** | 4,00 | 11,00 | 28,00 |  |  | 24,00 | 4,00 | 16,00 | 0,00 |  | 155,00 | 112,00 | 39,00 | 0,00 |  |  |  |  |  |  |  |  |  |  |  |  |  |  |
| **148.2** | 10,00 | 105,00 | 0,00 |  |  | 15,00 | 31,00 | 70,00 | 5,00 |  | 121,00 | 68,00 | 104,00 | 1,00 |  |  |  |  |  |  |  |  |  |  |  |  |  |  |
| **149.1** |  |  |  |  |  |  |  |  |  | 255,00 | 163,00 | 89,00 |  |  |  |  |  |  |  |  |  |  |  |  |  |  |  |  |
| **149.2** |  |  |  |  |  |  |  |  |  | 235,00 | 140,00 | 100,00 |  |  |  |  |  |  |  |  |  |  |  |  |  |  |  |  |
| **149.3** |  |  |  |  |  |  |  |  |  | 259,00 | 165,00 | 91,00 |  |  |  |  |  |  |  |  |  |  |  |  |  |  |  |  |
| **149.4** |  |  |  |  |  |  |  |  |  | 235,00 | 137,00 | 99,00 |  |  |  |  |  |  |  |  |  |  |  |  |  |  |  |  |
| **150** |  |  |  |  |  |  |  |  |  |  |  |  |  |  |  |  |  |  |  |  |  |  |  |  |  |  |  |  |
| **151** |  |  |  |  |  | 1,00 |  |  | 10,00 | 341,00 | 231,00 | 127,00 |  |  |  |  |  |  |  |  |  | 2,00 |  |  |  |  |  |  |
| **152 .1** |  |  |  |  |  |  |  |  |  | 299,00 | 108,00 | 81,00 |  |  |  |  |  |  |  |  |  |  |  |  |  |  |  |  |
| **152.2** |  |  |  |  |  |  |  |  |  | 288,00 | 128,00 | 82,00 |  |  |  |  |  |  |  |  |  |  |  |  |  |  |  |  |
| **152.3** |  |  |  |  |  |  |  |  |  | 317,00 | 122,00 | 94,00 |  |  |  |  |  |  |  |  |  |  |  |  |  |  |  |  |
| **153.1** |  |  | 48,00 |  |  | 185,00 | 13,00 |  |  |  | 195,00 | 119,00 | 37,00 |  |  |  |  |  |  |  |  | 171,00 |  |  |  |  |  |  |
| **153.2** |  |  | 33,00 |  |  | 99,00 | 1,00 |  |  |  | 275,00 | 152,00 | 4,00 |  |  |  |  |  |  |  |  | 91,00 |  |  |  |  |  |  |
| **155.1** |  |  |  |  |  | 14,00 | 30,00 |  |  |  |  |  |  |  |  |  |  |  |  |  |  | 4,00 |  |  |  |  |  |  |
| **155.2** |  |  |  |  |  | 19,00 | 31,00 |  |  |  |  |  |  |  |  |  |  |  |  |  |  | 5,00 |  |  |  |  |  |  |
| **156.1** |  |  |  |  |  |  |  |  |  |  | 166,00 | 101,00 |  |  |  |  |  |  |  |  |  |  |  |  |  |  |  |  |
| **156.2** |  |  |  |  |  |  |  |  |  |  | 181,00 | 99,00 |  |  |  |  |  |  |  |  |  |  |  |  |  |  |  |  |
| **157.1** |  |  |  |  |  | 20,00 | 79,00 |  |  |  | 123,80 | 84,90 |  |  |  |  |  |  |  |  |  |  |  |  |  |  |  |  |
| **157.2** |  |  |  |  |  | 67,00 | 6,00 |  |  |  | 153,20 | 111,80 |  |  |  |  |  |  |  |  |  |  |  |  |  |  |  |  |
| **158.1** |  |  | 2428,00 |  |  |  |  |  |  | 373,00 | 141,20 | 92,70 |  |  |  |  |  |  |  |  |  |  |  |  |  |  |  |  |
| **158.2** |  |  | 868,00 |  |  |  |  |  |  | 335,00 | 138,30 | 95,30 |  |  |  |  |  |  |  |  |  |  |  |  |  |  |  |  |
| **160** |  |  |  |  |  |  |  |  |  |  | 127,00 | 76,00 |  |  |  |  |  |  |  |  |  |  |  |  |  |  |  |  |
| **161** |  |  |  |  |  |  |  |  |  |  | 83,70 | 51,20 |  |  |  |  |  |  |  |  |  |  |  |  | 9,00 |  |  |  |
| **162.1** |  |  |  |  |  |  |  |  |  | 220,10 |  | 118,70 | 44,00 | 9,00 |  |  |  |  |  |  |  |  |  |  |  |  |  |  |
| **162 .2** |  |  |  |  |  |  |  |  |  | 227,80 |  | 105,80 | 104,00 | 50,00 |  |  |  |  |  |  |  |  |  |  |  |  |  |  |
| **163.1** |  |  |  |  |  |  |  |  |  |  | 133,00 | 83,00 |  |  |  |  |  |  |  |  |  |  |  |  |  |  |  |  |
| **163.2** |  |  |  |  |  |  |  |  |  |  | 131,00 | 82,00 |  |  |  |  |  |  |  |  |  |  |  |  |  |  |  |  |
| **164** |  |  |  |  |  |  |  |  |  |  |  |  |  |  |  |  |  |  |  |  |  |  |  |  |  |  |  |  |
| **169.1** |  |  |  |  |  |  |  |  |  | 266,00 | 167,00 | 117,00 |  |  |  |  |  |  |  |  |  |  |  |  |  |  |  |  |
| **169.2** |  |  |  |  |  |  |  |  |  | 223,00 | 135,00 | 86,00 |  |  |  |  |  |  |  |  |  |  |  |  |  |  |  |  |
| **170.1** |  |  |  |  |  |  |  |  |  |  | 100,00 | 62,00 |  |  |  |  |  |  |  |  |  |  |  |  |  |  |  |  |
| **170.2** |  |  |  |  |  |  |  |  |  |  | 119,00 | 75,00 |  |  |  |  |  |  |  |  |  |  |  |  |  |  |  |  |
| **170.3** |  |  |  |  |  |  |  |  |  |  | 98,00 | 62,00 |  |  |  |  |  |  |  |  |  |  |  |  |  |  |  |  |
| **170.4** |  |  |  |  |  |  |  |  |  |  | 103,00 | 63,00 |  |  |  |  |  |  |  |  |  |  |  |  |  |  |  |  |
| **171.1** |  |  |  |  |  | 56,00 | 2,00 |  |  |  | 128,70 | 84,90 |  |  |  |  |  |  |  |  |  |  |  |  |  |  |  |  |
| **171.2** |  |  |  |  |  |  |  |  |  |  | 155,90 | 124,10 |  |  |  |  |  |  |  |  |  |  |  |  |  |  |  |  |
| **172.1** |  |  |  |  |  | 100,00 |  |  |  |  |  |  |  |  |  |  |  |  |  |  |  |  |  |  |  |  |  |  |
| **172.2** |  |  |  |  |  | 190,00 |  |  |  |  |  |  |  |  |  |  |  |  |  |  |  |  |  |  |  |  |  |  |
| **173** |  |  |  |  |  |  |  |  |  |  | 110,70 | 73,60 |  |  |  |  |  |  |  |  |  |  |  |  |  |  |  |  |
| **174** |  |  |  |  |  |  |  |  |  |  | 207,00 | 103,00 | 30,00 | 207,00 |  |  |  |  |  |  |  |  |  |  |  |  |  |  |
| **177** | 695,00 | 6040,00 | 1122,00 |  |  |  |  |  |  |  | 135,00 | 84,00 | 6645,00 | 137,00 |  |  |  |  |  |  |  |  |  |  |  |  |  |  |
| **177.2** | 80,00 | 1658,00 | 65,00 |  |  |  |  |  |  |  | 140,00 | 83,00 | 1317,00 | 15,00 |  |  |  |  |  |  |  |  |  |  |  |  |  |  |
| **177.3** | 583,00 | 65,00 | 306,00 |  |  |  |  |  |  |  | 134,00 | 80,00 | 3791,00 | 39,00 |  |  |  |  |  |  |  |  |  |  |  |  |  |  |
| **178** |  |  |  |  |  |  |  |  |  |  | 65,00 | 46,00 |  |  |  |  |  |  |  |  |  |  |  |  |  |  |  |  |
| **179** |  |  |  |  |  |  |  |  |  | 230,54 | 120,24 | 78,66 |  |  |  |  |  |  |  |  |  |  |  |  |  |  |  |  |
| **180** |  |  |  |  |  |  |  |  |  | 287,00 |  |  |  |  |  |  |  |  |  |  |  |  |  |  |  |  |  |  |
| **181** |  | 51,00 | 40,00 |  |  |  |  |  |  |  | 143,00 | 105,00 | 85,00 |  |  |  |  |  |  |  |  |  |  |  |  |  |  |  |
| **182** |  |  |  |  |  |  |  |  |  |  | 110,60 | 88,90 |  |  |  |  |  |  |  |  |  |  |  |  |  |  |  |  |
| **183.1** |  |  |  |  |  |  |  |  |  |  |  | 67,85 |  |  |  |  |  |  |  |  |  |  |  |  |  |  |  |  |
| **183.2** |  |  |  |  |  |  |  |  |  |  |  | 79,60 |  |  |  |  |  |  |  |  |  |  |  |  |  |  |  |  |
| **184.1** |  |  |  |  |  |  | 82,00 |  |  |  | 160,00 | 104,00 |  |  |  |  |  |  |  |  |  |  |  |  |  |  |  |  |
| **184.2** |  |  |  |  |  | 66,00 | 0,00 |  |  |  | 159,00 | 107,00 |  |  |  |  |  |  |  |  |  |  |  |  |  |  |  |  |
| **184.3** |  |  |  |  |  | 17,00 | 17,00 |  |  |  | 177,00 | 115,00 |  |  |  |  |  |  |  |  |  |  |  |  |  |  |  |  |
| **185** |  |  |  |  |  |  |  |  |  |  | 100,00 |  |  |  |  |  |  |  |  |  |  |  |  |  |  |  |  |  |
| **186.1** |  |  |  |  |  | 60,00 | 81,00 |  |  |  | 164,00 |  |  |  |  |  |  |  |  |  |  |  |  |  |  |  |  |  |
| **186.2** |  |  |  |  |  | 30,00 | 26,00 |  |  |  | 160,00 |  |  |  |  |  |  |  |  |  |  |  |  |  |  |  |  |  |
| **187 .1** |  |  |  |  |  |  |  |  | 438,00 |  | 155,00 | 107,00 |  |  |  |  |  |  |  |  |  |  |  |  |  |  |  |  |
| **187.2** |  |  |  |  |  |  |  |  | 444,00 |  | 135,00 | 85,00 |  |  |  |  |  |  |  |  |  |  |  |  |  |  |  |  |
| **188** |  |  |  |  |  | 46,00 | 10,00 | 38,00 | 103,00 |  | 128,00 | 95,00 |  |  |  |  |  |  |  |  |  |  |  |  |  |  |  |  |
| **189.1** |  |  |  |  |  |  |  |  |  | 218,10 | 145,00 |  |  |  |  |  |  |  |  |  |  |  |  |  |  |  |  |  |
| **189 .2** |  |  |  |  |  |  |  |  |  | 241,80 | 160,00 |  |  |  |  |  |  |  |  |  |  |  |  |  |  |  |  |  |
| **190.1** |  |  |  |  |  |  |  |  |  | 222,00 |  |  |  |  |  |  |  |  |  |  |  |  |  |  |  |  |  |  |
| **190 .2** |  |  |  |  |  |  |  |  |  | 222,00 |  |  |  |  |  |  |  |  |  |  |  |  |  |  |  |  |  |  |
| **191.1** |  |  |  |  |  |  |  |  |  |  |  |  |  |  |  |  |  |  |  |  |  |  |  |  |  |  |  |  |
| **191.2** |  |  |  |  |  |  |  |  |  |  |  |  |  |  |  |  |  |  |  |  |  |  |  |  |  |  |  |  |
| **191.3** |  |  |  |  |  |  |  |  |  |  |  |  |  |  |  |  |  |  |  |  |  |  |  |  |  |  |  |  |
| **192.1** |  |  |  |  |  |  |  |  |  |  |  |  |  |  |  |  |  |  |  |  |  |  |  |  |  |  |  |  |
| **192.2** |  |  |  |  |  |  |  |  |  |  |  |  |  |  |  |  |  |  |  |  |  |  |  |  |  |  |  |  |
| **193.1** |  |  |  |  |  |  |  |  |  |  | 75,00 | 57,00 |  |  |  |  |  |  |  |  |  |  |  |  |  |  |  |  |
| **193.2** |  |  |  |  |  |  |  |  |  |  | 82,00 | 59,00 |  |  |  |  |  |  |  |  |  |  |  |  |  |  |  |  |
| **194.1** |  |  |  |  |  |  |  |  |  | 260,00 | 172,00 | 86,00 |  |  |  |  |  |  |  |  |  |  |  |  |  |  |  |  |
| **194.2** |  |  |  |  |  |  |  |  |  | 257,00 | 178,00 | 111,00 |  |  |  |  |  |  |  |  |  |  |  |  |  |  |  |  |
| **195.1** |  |  |  |  |  |  |  |  |  |  |  |  |  |  |  |  |  |  |  |  |  |  |  |  |  |  |  |  |
| **195.2** |  |  |  |  |  |  |  |  |  |  |  |  |  |  |  |  |  |  |  |  |  |  |  |  |  |  |  |  |
| **196** |  |  |  |  |  |  |  |  |  | 329,80 | 176,20 | 101,60 |  |  |  |  |  |  |  |  |  |  |  |  |  |  |  |  |
| **197.1** |  |  | 12,00 |  |  |  |  |  |  |  | 103,00 | 78,10 |  |  |  |  |  |  |  |  |  |  |  |  |  |  |  |  |
| **197.2** |  |  | 79,00 |  |  |  |  |  |  |  | 116,00 | 100,50 |  |  |  |  |  |  |  |  |  |  |  |  |  |  |  |  |
| **198.1** |  |  |  |  |  |  |  |  |  |  | 70,80 | 39,90 |  |  |  |  |  |  |  |  |  |  |  |  |  |  |  |  |
| **198.2** |  |  |  |  |  |  |  |  |  |  | 75,40 | 41,80 |  |  |  |  |  |  |  |  |  |  |  |  |  |  |  |  |
| **199.1** |  |  |  |  |  |  |  |  |  |  | 131,00 | 71,90 |  |  |  |  |  |  |  |  |  |  |  |  |  |  |  |  |
| **199.2** |  |  |  |  |  |  |  |  |  |  | 132,30 | 73,60 |  |  |  |  |  |  |  |  |  |  |  |  |  |  |  |  |
| **199.3** |  |  |  |  |  |  |  |  |  |  | 128,10 | 68,10 |  |  |  |  |  |  |  |  |  |  |  |  |  |  |  |  |
| **199.4** |  |  |  |  |  |  |  |  |  |  | 134,00 | 75,40 |  |  |  |  |  |  |  |  |  |  |  |  |  |  |  |  |
| **199.5** |  |  |  |  |  |  |  |  |  |  | 127,90 | 68,30 |  |  |  |  |  |  |  |  |  |  |  |  |  |  |  |  |
| **200.1** |  |  |  |  |  |  |  |  |  | 177,00 | 141,00 | 72,80 |  |  |  |  |  |  |  |  |  |  |  |  |  |  |  |  |
| **200.2** |  |  |  |  |  |  |  |  |  | 206,00 | 154,00 | 95,60 |  |  |  |  |  |  |  |  |  |  |  |  |  |  |  |  |
| **201** |  |  |  |  |  | 186,00 | 93,00 |  | 377,00 |  | 133,00 | 80,00 |  |  |  |  |  |  |  |  |  |  |  |  |  |  |  |  |
| **202** |  |  |  |  |  |  |  |  |  |  | 156,23 | 102,55 |  |  |  |  |  |  |  |  |  |  |  |  |  |  |  |  |
| **203** |  |  |  |  |  |  |  |  |  |  |  |  |  |  |  |  |  |  |  |  |  |  |  |  |  |  |  |  |
| **204.1** |  |  |  |  |  | 41,00 |  | 47,00 | 169,00 | 201,00 | 125,00 | 87,00 |  |  |  |  |  |  |  |  |  |  |  |  |  |  |  |  |
| **204.2** |  |  |  |  |  | 0,00 |  | 137,00 | 166,00 | 228,00 | 121,00 | 88,00 |  |  |  |  |  |  |  |  |  |  |  |  |  |  |  |  |
| **205 .1** |  |  |  |  |  |  |  | 3,97 | 90,50 |  |  |  |  |  |  |  |  |  |  |  |  |  |  |  |  |  |  |  |
| **205.2** |  |  |  |  |  |  |  | 0,00 | 96,80 |  |  |  |  |  |  |  |  |  |  |  |  |  |  |  |  |  |  |  |
| **206** |  |  |  |  |  |  |  |  |  |  | 158,72 | 114,48 |  |  |  |  |  |  |  |  |  |  |  |  |  |  |  |  |
| **207** |  |  | 911,18 |  |  | 3521,87 |  |  |  |  | 135,60 | 91,10 | 8599,92 | 255,95 | 9234,68 | 890,71 |  |  |  | 6429,46 | 2293,31 | 685,95 |  |  |  |  |  |  |
| **208** |  |  |  |  |  |  |  |  |  |  | 99,00 | 72,00 |  |  |  |  |  |  |  |  |  |  |  |  |  |  |  |  |
| **209.1** |  |  |  |  |  |  |  |  |  | 216,00 | 143,00 |  | 39,00 | 0,00 |  |  |  |  |  |  |  |  |  |  |  |  |  |  |
| **209.2** |  |  |  |  |  |  |  |  |  | 212,00 | 139,00 |  | 107,00 | 1,00 |  |  |  |  |  |  |  |  |  |  |  |  |  |  |
| **209.3** |  |  |  |  |  |  |  |  |  | 208,00 | 134,00 |  | 148,00 | 8,00 |  |  |  |  |  |  |  |  |  |  |  |  |  |  |
| **209.4** |  |  |  |  |  |  |  |  |  | 196,00 | 127,00 |  | 737,00 | 8,00 |  |  |  |  |  |  |  |  |  |  |  |  |  |  |
| **209.5** |  |  |  |  |  |  |  |  |  | 239,00 | 169,00 |  | 179,00 | 6,00 |  |  |  |  |  |  |  |  |  |  |  |  |  |  |
| **210** |  |  |  |  |  |  |  |  |  | 148,20 | 73,20 | 50,10 |  |  |  |  |  |  |  |  |  |  |  |  |  |  |  |  |
| **211.1** |  |  |  |  |  |  |  |  |  |  | 108,40 | 86,00 |  |  |  |  |  |  |  |  |  |  |  |  |  |  |  |  |
| **211.2** |  |  |  |  |  |  |  |  |  |  | 146,20 | 115,90 |  |  |  |  |  |  |  |  | 118,00 |  |  |  |  |  |  |  |
| **212** |  |  |  |  |  | 9,00 |  |  |  | 249,00 | 144,00 | 80,00 |  |  |  |  |  |  |  |  |  |  |  |  |  |  |  |  |
| **213** |  |  |  |  |  |  |  |  |  |  | 99,00 | 65,00 |  |  |  |  |  |  |  |  |  |  |  |  |  |  |  |  |
| **214** |  |  |  |  |  |  | 7,00 |  |  |  | 167,00 | 113,00 |  |  |  |  |  |  |  |  |  |  |  |  |  |  |  |  |
| **215** |  |  |  |  |  |  |  |  |  | 206,80 |  |  |  |  |  |  |  |  |  |  |  |  |  |  |  |  |  |  |
| **216** |  |  |  |  |  |  |  |  |  | 266,60 | 146,20 | 115,90 |  |  |  |  |  |  |  |  |  |  |  |  |  |  |  |  |
| **217** |  |  |  | 74,00 | 15,00 | 89,00 | 10,00 |  | 124,00 |  |  |  |  |  |  |  |  |  |  |  |  |  |  |  |  |  |  |  |
| **218** |  |  |  |  |  |  |  |  |  |  | 217,00 | 110,00 |  |  |  |  |  |  |  |  |  |  |  |  |  |  |  |  |
| **219.1** |  |  | 1,00 |  |  |  |  |  |  | 317,00 | 163,70 | 110,30 |  |  |  |  |  |  |  |  |  |  |  |  |  |  |  |  |
| **219 .2** |  |  | 1,00 |  |  |  |  |  |  | 316,50 | 178,60 | 90,60 |  |  |  |  |  |  |  |  |  |  |  |  |  |  |  |  |
| **220.1** |  |  |  |  |  | 62,00 | 7,00 |  |  | 205,00 | 132,00 | 75,00 |  |  |  |  |  |  |  |  |  |  |  |  |  |  |  |  |
| **220.2** |  |  |  |  |  | 32,00 | 5,00 |  |  | 210,00 | 137,00 | 77,00 |  |  |  |  |  |  |  |  |  |  |  |  |  |  |  |  |
| **220.3** |  |  |  |  |  | 22,00 | 2,00 |  |  | 189,00 | 129,00 | 69,00 |  |  |  |  |  |  |  |  |  |  |  |  |  |  |  |  |
| **220.4** |  |  |  |  |  | 8,00 | 0,00 |  |  | 198,00 | 128,00 | 72,00 |  |  |  |  |  |  |  |  |  |  |  |  |  |  |  |  |
| **220.5** |  |  |  |  |  | 89,00 | 6,00 |  |  | 209,00 | 144,00 | 81,00 |  |  |  |  |  |  |  |  |  |  |  |  |  |  |  |  |
| **221** |  |  |  |  |  |  |  |  |  |  | 64,10 | 43,60 |  |  |  |  |  |  |  |  |  |  |  |  |  |  |  |  |
| **222.1** |  |  |  |  |  |  |  |  |  |  |  |  |  |  |  |  |  |  |  |  |  |  |  |  |  |  |  |  |
| **222.2** |  |  |  |  |  |  |  |  |  |  |  |  |  |  |  |  |  |  |  |  |  |  |  |  |  |  |  |  |
| **223** |  |  |  |  |  |  |  |  |  |  | 115,80 | 82,00 |  |  |  |  |  |  |  |  |  |  |  |  |  |  |  |  |
| **224** |  |  |  |  |  |  |  |  |  |  | 147,88 | 105,75 |  |  |  |  |  |  |  |  |  |  |  |  |  |  |  |  |
| **225.1** |  |  |  |  |  |  |  |  |  | 227,22 | 138,53 | 95,79 |  |  |  |  |  |  |  |  |  |  |  |  |  |  |  |  |
| **225.2** |  |  |  |  |  |  |  |  |  | 252,77 | 147,00 | 108,59 |  |  |  |  |  |  |  |  |  |  |  |  |  |  |  |  |
| **Ʃ** | 2578,00 | 7982,00 | 7774,18 | 2640,00 | 294,00 | 10466,87 | 1351,00 | 504,97 | 9301,30 |  |  |  | 28375,92 | 851,95 | 10227,68 | 890,71 | 14,00 | 4545,90 | 1028,20 | 6474,46 | 2473,31 | 1273,95 | 1,00 | 17,00 | 62,00 | 65,00 | 47,00 | 46,00 |
| **mean** |  |  |  |  |  |  |  |  |  | 235,29 | 137,56 | 90,18 |  |  |  |  |  |  |  |  |  |  |  |  |  |  |  |  |
| **SD** |  |  |  |  |  |  |  |  |  | 59,67 | 33,74 | 21,10 |  |  |  |  |  |  |  |  |  |  |  |  |  |  |  |  |
| **missings** |  |  |  |  |  |  |  |  |  | 66.0 | 13.0 | 29.0 |  |  |  |  |  |  |  |  |  |  |  |  |  |  |  |  |

|  | **MVRepl** | **MVR** | **LAAb** | **LAAo** | **PFOc** | **ASDc** | **Tres** | **TVR** | **AVR** | **AAR** | **MVR size** | **CTS** | **BL** | **secRA** | **RBCs** | **FFPs** | **PLTs** | **SAM** | **LCO** | **PVL** | **cal AML** | **calAnn** | **pPML** | **pAML** | **pAML+PML** | **ChR** | **ChE** | **cleft** | **AnnD** | **rPap** | **MRVol** | **A1** | **A2** | **A3** | **P1** | **P2** | **P3** |
| --- | --- | --- | --- | --- | --- | --- | --- | --- | --- | --- | --- | --- | --- | --- | --- | --- | --- | --- | --- | --- | --- | --- | --- | --- | --- | --- | --- | --- | --- | --- | --- | --- | --- | --- | --- | --- | --- |
| **1** |  |  | 136,00 | 12,00 | 48,00 |  |  |  |  |  |  | 1,00 |  | 12,00 | 69,00 |  | 30,00 |  | 1,00 |  |  |  |  |  |  |  |  |  |  |  |  |  |  |  |  |  |  |
| **3** |  | 19,00 |  |  |  |  |  |  |  |  |  |  |  |  | 0,00 |  | 0,00 |  |  |  |  |  |  |  |  |  |  |  |  |  |  |  |  |  |  |  |  |
| **4** |  |  |  |  |  |  |  |  |  |  |  |  |  |  |  |  |  |  |  |  |  |  |  |  |  |  |  |  |  |  |  |  |  |  |  |  |  |
| **5** |  |  |  |  |  |  |  |  |  |  |  |  |  |  |  |  |  |  |  |  |  |  | 74,00 | 54,00 | 128,00 |  |  | 2,00 |  |  |  | 13,00 | 24,00 | 17,00 | 19,00 | 47,00 | 8,00 |
| **7** |  |  |  |  |  |  |  |  |  |  |  | 0,00 | 285,30 |  | 1,20 | 2,90 |  |  |  |  |  |  |  |  |  |  |  |  |  |  |  | 7,00 | 15,00 | 4,00 | 6,00 | 21,00 | 9,00 |
| **8** | 850,00 | 2619,00 |  |  |  |  |  | 312,00 | 4205,00 |  |  |  |  |  | 1440,00 |  |  |  |  |  |  |  |  |  |  |  |  |  |  |  |  |  |  |  |  |  |  |
| **9** | 8,00 |  | 21,00 |  |  |  |  | 2,00 |  |  | 31,50 | 9,00 |  |  | 44,00 |  |  |  |  |  |  |  | 114,00 | 14,00 | 128,00 |  |  |  |  |  |  |  |  |  |  |  |  |
| **13** | 52,00 |  |  |  |  |  |  |  |  |  |  |  |  |  |  |  |  |  |  |  |  |  |  |  |  |  |  |  |  |  |  |  |  |  |  |  |  |
| **14.1** |  |  | 2,00 | 5,00 | 2,00 |  |  |  |  |  |  |  |  |  | 4,00 |  |  |  |  |  |  |  |  |  |  |  |  |  |  |  |  |  |  |  | 1,00 | 1,00 |  |
| **14.2** |  |  | 5,00 | 5,00 | 5,00 |  |  |  |  |  |  |  |  |  | 5,00 |  |  |  |  |  |  |  |  |  |  |  |  |  |  |  |  |  |  |  | 3,00 | 4,00 |  |
| **14.3** |  |  | 9,00 | 11,00 | 10,00 |  |  |  |  |  |  |  |  |  | 5,00 |  |  |  |  |  |  |  |  |  |  |  |  |  |  |  |  |  |  |  | 2,00 | 6,00 |  |
| **15.1** |  |  |  |  |  |  |  |  |  |  | 34,00 |  |  |  |  |  |  |  |  |  |  | 10,00 |  |  |  |  |  |  | 11,00 |  |  |  |  |  |  |  |  |
| **15.2** |  |  |  |  |  |  |  |  |  |  | 36,80 |  |  |  |  |  |  |  |  |  |  | 5,00 |  |  |  |  |  |  | 23,00 |  |  |  |  |  |  |  |  |
| **18.1** |  |  |  |  |  | 3,00 |  | 0,00 |  |  |  |  | 203,00 |  |  |  |  |  |  |  |  |  |  |  |  |  |  |  |  |  |  |  |  |  |  |  |  |
| **18.2** |  |  |  |  |  | 0,00 |  | 0,00 |  |  |  |  | 253,00 |  |  |  |  |  |  |  |  |  |  |  |  |  |  |  |  |  |  |  |  |  |  |  |  |
| **18.3** |  |  |  |  |  | 0,00 |  | 1,00 |  |  |  |  | 177,00 |  |  |  |  |  |  |  |  |  |  |  |  |  |  |  |  |  |  |  |  |  |  |  |  |
| **20** |  |  | 78,00 |  | 72,00 | 6,00 |  | 66,00 |  |  |  | 20,00 |  |  | 129,00 | 124,00 | 24,00 |  |  |  |  |  | 634,00 | 23,00 | 657,00 | 752,00 | 587,00 |  | 1084,00 |  |  |  |  |  |  |  |  |
| **21** |  |  | 23,00 | 9,00 |  | 5,00 |  | 20,00 |  |  | 28,90 | 0,00 |  |  | 25,00 |  |  |  | 0,00 |  |  |  | 59,00 | 17,00 | 76,00 |  |  |  |  |  |  |  |  |  |  |  |  |
| **26** |  |  |  | 14,00 |  | 2,00 |  | 2,00 |  |  |  |  |  |  | 2,20 |  |  | 0,00 |  |  |  |  | 46,00 | 38,00 | 84,00 |  |  |  |  |  |  | 5,00 | 4,00 | 2,00 | 2,00 | 23,00 | 16,00 |
| **29** | 11,00 | 140,00 | 66,00 |  |  | 9,00 |  | 56,00 |  |  | 29,30 | 1,00 |  |  |  |  |  |  |  |  |  |  |  |  |  |  |  |  |  |  | 0,90 |  |  |  |  |  |  |
| **30** |  |  | 3,00 |  | 3,00 | 3,00 |  | 24,00 |  |  |  |  |  |  | 48,00 |  |  |  |  |  |  |  | 131,00 | 10,00 | 141,00 | 79,00 |  |  |  |  |  |  |  |  |  |  |  |
| **31.1** |  |  | 15,00 | 14,00 | 57,00 |  |  | 11,00 |  |  | 35,00 |  |  |  |  |  |  |  |  |  | ´ |  |  |  |  |  |  | 6,00 |  |  | 66,60 | 1,00 | 2,00 | 1,30 | 1,10 | 2,40 | 1,20 |
| **31.2** |  |  | 11,00 | 11,00 | 23,00 |  |  | 15,00 |  |  | 33,00 |  |  |  |  |  |  |  |  |  | ´ |  |  |  |  |  |  | 4,00 |  |  | 65,30 |  |  |  |  |  |  |
| **35** |  |  |  |  |  |  |  |  |  |  |  |  |  |  |  |  |  |  |  |  |  |  |  |  |  |  |  |  |  |  |  |  |  |  |  |  |  |
| **38** |  |  | 7,00 |  |  |  |  | 9,00 |  | 1,00 |  | 1,00 |  |  |  |  |  |  | 0,00 |  |  |  | 15,00 | 8,00 | 23,00 |  |  |  |  |  |  |  |  |  |  |  |  |
| **40** | 13,00 | 78,00 |  |  |  |  |  | 10,00 |  |  | 32,00 |  |  |  |  |  |  |  | 5,00 |  |  |  |  |  |  |  |  |  |  |  |  |  |  |  |  |  |  |
| **41** | 0,00 | 5,00 | 29,00 | 5,00 | 8,00 |  |  | 12,00 |  |  |  |  |  |  | 12,00 |  | 7,00 |  | 1,00 |  | 6,00 | 101,00 | 76,00 | 21,00 | 97,00 |  |  | 5,00 |  |  |  |  |  |  |  |  |  |
| **42** |  |  |  |  |  |  |  |  |  |  |  |  |  |  |  |  |  |  |  |  |  |  |  |  |  |  |  | 18,00 |  |  |  |  |  | 1,00 |  | 3,00 |  |
| **43** | 6,00 | 18,00 |  |  |  |  |  | 8,00 | 25,00 |  |  |  |  |  | 13,00 |  |  |  |  |  |  |  |  |  |  |  |  |  |  |  |  |  |  |  |  |  |  |
| **44.1** |  |  |  |  |  |  |  |  |  |  |  | 0,00 |  |  | 0,00 |  |  |  |  |  |  |  |  |  |  |  |  |  |  |  |  |  |  |  |  |  |  |
| **44.2** |  |  |  |  |  |  |  |  |  |  |  | 0,00 |  |  | 0,00 |  |  |  |  |  |  |  |  |  |  |  |  |  |  |  |  |  |  |  |  |  |  |
| **45** |  |  |  |  |  |  |  |  |  |  |  |  |  |  | 7,00 |  |  |  |  |  |  |  |  |  |  |  |  |  |  |  |  |  |  |  |  |  |  |
| **46** |  |  | 36,00 |  |  | 14,00 |  | 8,00 |  |  |  | 0,00 |  |  |  |  |  |  |  |  |  |  | 100,00 | 28,00 | 128,00 |  |  | 28,00 |  |  |  |  |  |  |  |  |  |
| **47** | 18,00 | 36,00 | 13,00 | 0,00 | 2,00 |  |  |  |  |  |  |  |  |  | 13,00 |  | 11,00 |  |  |  |  |  |  |  |  |  |  | 14,00 |  |  |  |  |  |  |  |  |  |
| **48** | 32,00 |  |  |  |  |  |  |  |  |  |  |  |  |  | 1,00 | 0,00 | 0,00 |  | 2,00 |  |  |  |  |  |  |  |  |  |  |  |  |  |  |  |  |  |  |
| **51** | 11,00 | 8,00 | 5,00 |  |  |  |  | 7,00 | 14,00 |  |  |  |  |  |  |  |  |  |  |  |  |  |  |  |  |  |  |  |  |  |  |  |  |  |  |  |  |
| **53.1** | 2,00 |  | 21,00 | 11,00 | 6,00 |  |  | 37,00 |  |  |  | 1,00 |  |  | 6,00 |  | 4,00 |  |  |  |  |  | 48,00 | 32,00 | 80,00 |  |  | 27,00 |  |  |  |  |  |  |  |  |  |
| **53.2** |  |  | 6,00 | 0,00 | 4,00 |  |  |  |  |  | 34,10 | 1,00 |  |  | 2,00 |  | 2,00 |  |  |  |  |  |  |  |  |  |  | 6,00 |  |  |  |  |  |  |  |  |  |
| **53.3** |  |  | 8,00 | 3,00 | 1,00 |  |  |  |  |  | 33,90 | 0,00 |  |  | 2,00 |  | 0,00 |  |  |  |  |  |  |  |  |  |  | 11,00 |  |  |  |  |  |  |  |  |  |
| **55.1** | 4,00 | 24,00 |  |  |  |  |  |  |  |  |  |  |  |  |  |  |  |  |  |  |  |  |  |  |  |  |  |  |  |  |  |  |  |  |  |  |  |
| **55.2** | 5,00 | 24,00 |  |  |  |  |  |  |  |  |  |  |  |  |  |  |  |  |  |  |  |  |  |  |  |  |  |  |  |  |  |  |  |  |  |  |  |
| **56.1** |  |  |  |  |  |  |  |  |  |  |  | 0,00 |  |  |  |  |  |  |  |  |  |  |  |  |  |  |  |  |  |  |  |  |  |  |  |  |  |
| **56.2** |  |  |  |  |  |  |  |  |  |  |  | 1,00 |  |  |  |  |  |  |  |  |  |  |  |  |  |  |  |  |  |  |  |  |  |  |  |  |  |
| **57** |  |  |  |  |  |  |  |  |  |  |  |  | 500,00 |  | 4,00 | 2,00 | 1,00 |  |  |  |  |  | 68,00 | 3,00 | 71,00 |  |  |  |  |  |  |  |  |  |  |  |  |
| **63.1** |  |  | 10,00 |  |  |  |  | 21,00 |  |  | 35,00 |  |  |  |  |  |  |  |  |  |  |  |  |  |  |  |  |  |  |  |  |  |  |  |  |  |  |
| **63.2** |  |  | 6,00 |  |  |  |  | 16,00 |  |  | 35,00 |  |  |  |  |  |  |  |  |  |  |  |  |  |  |  |  |  |  |  |  |  |  |  |  |  |  |
| **65** | 40,00 | 226,00 | 55,00 | 57,00 |  | 42,00 |  | 20,00 |  |  |  | 3,00 |  |  |  |  |  |  |  |  |  |  |  |  |  |  |  |  |  |  |  |  |  |  |  |  |  |
| **66** | 1653,00 | 5224,00 | 1272,00 | 964,00 |  |  |  | 1140,00 | 128,00 | 10,00 |  | 128,00 |  |  |  |  |  |  | 234,00 |  |  |  |  |  |  |  |  |  |  |  |  |  |  |  |  |  |  |
| **67** |  |  | 29,00 |  |  |  |  | 27,00 |  |  |  |  |  |  | 252,00 | 177,00 | 155,00 |  |  |  |  |  |  |  |  |  |  |  |  |  |  |  |  |  |  |  |  |
| **68** | 16,00 |  |  |  |  |  |  |  |  |  |  |  | 860,00 |  | 0,00 |  |  |  |  |  |  |  | 68,00 | 9,00 | 77,00 |  |  |  |  |  |  |  |  |  |  |  |  |
| **71** | 44,00 | 165,00 | 21,00 |  |  | 4,00 |  | 7,00 |  |  |  |  |  |  |  |  |  |  |  |  |  |  |  |  |  |  |  |  |  |  |  |  |  |  |  |  |  |
| **72** |  |  | 100,00 |  |  | 23,00 |  | 80,00 |  |  |  |  |  |  |  |  |  |  |  |  |  |  |  |  |  |  |  |  |  |  |  |  |  |  |  |  |  |
| **73** | 67,00 | 17,00 | 4,00 |  |  | 3,00 |  | 10,00 |  |  |  |  |  |  |  |  |  |  |  |  |  |  |  |  |  |  |  |  |  |  |  |  |  |  |  |  |  |
| **74** |  |  | 14,00 | 7,00 | 33,00 | 0,00 |  |  |  |  |  | 3,00 |  |  | 23,00 |  |  |  |  |  |  |  |  |  |  |  |  |  |  |  |  |  |  |  |  |  |  |
| **75** |  |  | 5,00 | 6,00 | 8,00 |  |  | 1,00 |  |  |  | 1,00 |  |  | 2,00 | 0,00 | 0,00 |  |  |  |  |  |  |  |  |  |  | 15,00 |  |  |  |  |  |  |  |  |  |
| **77** |  | 59,00 | 22,00 | 6,00 |  |  |  | 6,00 |  |  |  | 0,00 |  |  | 6,00 |  |  |  |  |  |  |  |  |  |  |  |  |  |  |  |  |  |  |  |  |  |  |
| **78.1** |  |  | 19,00 |  | 19,00 |  |  | 12,00 |  |  |  |  |  |  |  |  |  |  |  |  |  |  |  |  |  |  |  |  |  |  |  |  |  |  |  |  |  |
| **78.2** |  |  | 9,00 |  | 20,00 |  |  | 13,00 |  |  |  |  |  |  |  |  |  |  |  |  |  |  |  |  |  |  |  |  |  |  |  |  |  |  |  |  |  |
| **79** |  |  |  | 41,80 |  |  |  | 56,70 |  |  |  |  |  |  | 3,00 |  |  |  | 4,00 |  |  |  |  |  |  |  |  |  |  |  |  |  |  |  |  |  |  |
| **80** | 4,00 | 242,00 | 48,00 |  |  |  |  | 94,00 |  |  |  | 11,00 |  |  | 0,00 |  |  |  | 9,00 | 0,00 |  | 2,00 |  |  |  |  |  |  |  |  |  |  |  |  |  |  |  |
| **82** |  |  | 78,00 |  |  | 6,00 |  | 70,00 |  |  | 30,30 |  |  |  |  |  |  |  |  |  |  |  | 230,00 | 79,00 | 309,00 |  |  |  |  |  |  |  |  |  |  |  |  |
| **83** |  |  |  |  |  | 5,00 |  | 8,00 |  |  |  |  |  |  |  |  |  |  |  |  |  |  | 7,00 | 12,00 | 19,00 |  |  |  |  |  |  |  |  |  |  |  |  |
| **86** |  |  | 8,00 |  |  |  |  | 5,00 |  |  | 34,00 |  |  |  |  |  |  |  |  |  |  |  |  |  |  |  |  |  |  |  |  |  |  |  |  |  |  |
| **87.1** |  |  |  |  |  |  |  |  |  |  |  |  |  |  |  |  |  |  |  |  |  |  |  |  |  |  |  |  |  |  |  |  |  |  |  |  |  |
| **87.2** |  |  |  |  |  |  |  |  |  |  |  |  |  |  |  |  |  |  |  |  |  |  |  |  |  |  |  |  |  |  |  |  |  |  |  |  |  |
| **93** |  |  | 20,00 | 12,00 |  | 6,00 |  | 12,00 |  |  |  | 1,00 |  |  |  |  |  | 2,00 |  |  |  |  | 52,00 | 24,00 | 76,00 |  |  | 7,00 |  | 64,00 |  | 4,00 | 12,00 | 8,00 | 8,00 | 36,00 | 8,00 |
| **98** | 0,00 | 225,00 | 1,00 |  |  |  |  |  |  |  |  |  |  |  | 9,00 | 5,00 | 0,00 |  |  |  |  |  | 68,00 | 78,00 | 146,00 |  |  |  |  |  |  |  |  |  |  |  |  |
| **99.1** | 216,00 | 4959,00 |  |  |  |  |  |  |  |  |  |  |  |  |  |  |  |  |  |  |  |  |  |  |  |  |  |  |  |  |  |  |  |  |  |  |  |
| **99.2** | 1710,00 | 4959,00 |  |  |  |  |  |  |  |  |  |  |  |  |  |  |  |  |  |  |  |  |  |  |  |  |  |  |  |  |  |  |  |  |  |  |  |
| **101.1** |  | 16,00 | 5,00 |  |  |  |  | 5,00 |  |  |  | 2,00 |  |  |  |  |  |  | 0,00 |  |  |  |  |  |  |  |  |  |  |  |  |  |  |  |  |  |  |
| **101.2** |  | 34,00 | 6,00 |  |  |  |  | 39,00 |  |  |  | 6,00 |  |  |  |  |  |  | 16,00 |  |  |  |  |  |  |  |  |  |  |  |  |  |  |  |  |  |  |
| **102** | 153,00 | 34,00 | 18,00 | 7,00 |  | 4,00 |  | 44,00 | 17,00 |  |  |  |  |  |  |  |  |  | 15,00 | 6,00 |  |  | 50,00 | 39,00 | 89,00 |  |  |  |  |  |  |  |  |  |  |  |  |
| **103** |  | 184,00 |  |  |  |  |  |  |  |  |  |  |  |  | 0,00 |  |  |  | 8,00 |  |  |  |  |  |  |  |  |  |  |  |  |  |  |  |  |  |  |
| **104** |  | 39,60 |  |  |  |  |  | 48,00 |  |  |  |  |  |  |  |  |  |  | 3,00 |  |  |  |  |  |  |  |  |  |  |  |  |  |  |  |  |  |  |
| **105** |  | 549,00 | 111,00 |  |  |  |  |  |  |  |  |  |  |  | 92,00 |  |  |  |  |  |  |  |  |  |  |  |  |  |  |  |  |  |  |  |  |  |  |
| **107** |  |  |  |  |  |  |  | 39,00 | 98,00 |  |  |  | 477,60 |  | 16,00 |  |  |  | 3,00 | 0,00 |  |  |  |  |  |  |  |  |  |  |  |  |  |  |  |  |  |
| **108** | 57,00 | 15,00 |  |  |  |  |  |  | 72,00 |  |  |  |  |  | 1,30 |  |  |  |  |  |  |  |  |  |  |  |  |  |  |  |  |  |  |  |  |  |  |
| **110** |  |  |  |  |  |  |  | 20,00 | 4,00 |  |  |  |  |  | 26,00 |  |  | 4,00 |  |  |  |  | 103,00 | 10,00 | 113,00 |  |  |  |  |  |  |  |  |  |  |  |  |
| **111** |  |  | 24,00 |  |  |  |  | 33,00 |  |  |  |  |  |  | 72,00 |  |  |  |  |  |  |  | 196,00 | 15,00 | 211,00 |  |  |  |  |  |  |  |  |  |  |  |  |
| **112** | 51,00 | 103,00 | 18,00 | 3,00 | 5,00 |  | 1,00 |  |  |  |  | 0,00 |  |  |  |  |  |  |  |  |  |  | 100,00 | 9,00 | 109,00 |  |  |  |  |  |  |  |  |  |  |  |  |
| **113.1** |  |  |  |  |  |  |  |  |  |  | 32,70 |  |  |  |  |  |  |  |  |  |  |  |  |  |  |  |  |  |  |  |  |  |  |  |  |  |  |
| **113.2** |  |  |  |  |  |  |  |  |  |  | 32,90 |  |  |  |  |  |  |  |  |  |  |  |  |  |  |  |  |  |  |  |  |  |  |  |  |  |  |
| **115.1** | 39,00 |  |  | 70,00 |  |  |  | 79,00 |  |  |  | 14,00 |  |  |  |  |  | 6,00 | 15,00 |  |  |  |  |  |  |  |  |  |  |  |  |  |  |  |  |  |  |
| **115.2** | 24,00 |  |  | 53,00 |  |  |  | 55,00 |  |  |  | 9,00 |  |  |  |  |  | 3,00 | 9,00 |  |  |  |  |  |  |  |  |  |  |  |  |  |  |  |  |  |  |
| **115.3** | 14,00 |  |  | 37,00 |  |  |  | 40,00 |  |  |  | 6,00 |  |  |  |  |  | 1,00 | 5,00 |  |  |  |  |  |  |  |  |  |  |  |  |  |  |  |  |  |  |
| **116.1** |  | 97,00 |  |  |  |  |  |  |  |  |  |  |  |  | 35,00 |  |  |  |  |  |  |  |  |  |  |  |  |  |  |  |  |  |  |  |  |  |  |
| **116.2** |  | 96,00 |  |  |  |  |  | 119,00 |  |  |  |  |  |  | 33,00 |  |  |  |  |  |  |  |  |  |  |  |  |  |  |  |  |  |  |  |  |  |  |
| **117.1** | 29,00 | 22,00 | 7,00 |  |  |  |  |  |  |  | 28,00 |  |  |  |  |  |  |  |  |  |  |  |  |  |  |  |  |  |  |  |  |  |  |  |  |  |  |
| **117.2** | 35,00 | 16,00 | 4,00 |  |  |  |  |  |  |  | 32,00 |  |  |  |  |  |  |  |  |  |  |  |  |  |  |  |  |  |  |  |  |  |  |  |  |  |  |
| **118** | 238,00 | 507,00 |  |  |  | 30,00 |  | 56,00 |  |  |  |  |  |  | 55,00 |  |  |  |  |  |  |  |  |  |  |  |  |  |  |  |  |  |  |  |  |  |  |
| **119.1** | 72,00 | 575,00 |  |  |  |  |  | 216,00 |  |  |  |  |  |  |  |  |  |  |  |  |  |  |  |  |  |  |  |  |  |  |  |  |  |  |  |  |  |
| **119.2** | 41,00 | 215,00 |  |  |  |  |  | 12,00 |  |  |  |  |  |  |  |  |  |  |  |  |  |  |  |  |  |  |  |  |  |  |  |  |  |  |  |  |  |
| **121.1** |  | 63,00 |  |  |  |  |  |  |  |  |  |  |  |  |  |  |  |  | 0,00 |  |  |  |  |  |  |  |  |  |  |  |  |  |  |  |  |  |  |
| **121.2** |  | 33,00 |  |  |  |  |  |  |  |  |  |  |  |  |  |  |  |  | 0,00 |  |  |  |  |  |  |  |  |  |  |  |  |  |  |  |  |  |  |
| **122.1** | 49,00 |  |  |  |  |  |  | 12,00 |  |  |  |  |  |  | 5,00 |  |  |  |  |  |  |  |  |  |  |  |  |  |  |  |  |  |  |  |  |  |  |
| **122.2** | 50,00 |  |  |  |  |  |  | 8,00 |  |  |  |  |  |  | 4,00 |  |  |  |  |  |  |  |  |  |  |  |  |  |  |  |  |  |  |  |  |  |  |
| **123.1** | 65,00 | 6,00 |  |  |  |  |  |  |  |  | 34,00 | 1,00 |  |  | 18,00 |  |  |  |  |  |  |  |  |  |  |  |  | 4,00 |  |  |  |  |  |  |  |  |  |
| **123.2** | 41,00 | 1,00 |  |  |  |  |  |  |  |  | 31,00 | 1,00 |  |  | 9,00 |  |  |  |  |  |  |  |  |  |  |  |  | 1,00 |  |  |  |  |  |  |  |  |  |
| **127** |  |  |  |  |  |  |  |  |  |  |  |  |  |  |  |  |  |  |  |  |  |  |  |  | 69,00 |  |  |  |  |  |  |  |  |  |  |  |  |
| **128** | 47,00 | 66,00 | 27,00 | 3,00 |  |  |  | 4,00 |  |  |  |  |  |  |  |  |  |  |  |  |  |  |  |  |  |  |  |  |  |  |  |  |  |  |  |  |  |
| **129** |  |  |  | 25,00 | 23,00 | 11,00 |  | 52,00 |  |  |  | 1,00 |  |  |  |  |  |  |  |  |  |  |  |  |  |  |  |  |  |  |  |  |  |  |  |  |  |
| **130** |  |  |  |  |  |  |  | 34,00 |  |  |  |  |  |  |  |  |  |  | 6,00 |  |  |  |  |  |  |  |  |  |  |  |  |  |  |  |  |  |  |
| **131.1** | 37,00 | 78,00 | 12,00 |  |  |  |  | 10,00 |  |  |  | 1,00 |  |  |  |  |  |  |  |  |  |  |  |  |  |  |  |  |  |  |  |  |  |  |  |  |  |
| **131.2** | 40,00 | 77,00 | 5,00 |  |  |  |  | 9,00 |  |  |  | 3,00 |  |  |  |  |  |  |  |  |  |  |  |  |  |  |  |  |  |  |  |  |  |  |  |  |  |
| **132 .1** |  | 118,00 | 24,00 | 0,00 | 0,00 |  |  | 0,00 |  |  |  |  |  |  |  |  |  |  |  |  |  |  | 88,00 | 30,00 |  |  |  |  |  |  |  |  |  |  |  |  |  |
| **132 .2** |  | 1267,00 | 268,00 | 864,00 | 264,00 |  |  | 81,00 |  |  |  |  |  |  |  |  |  |  |  |  |  |  | 838,00 | 366,00 |  |  |  |  |  |  |  |  |  |  |  |  |  |
| **133** |  |  |  |  |  |  |  |  |  | 52,00 |  |  |  |  |  |  |  |  |  |  |  |  |  |  |  |  |  |  |  |  |  |  |  |  |  |  |  |
| **134.1** | 10,00 |  |  | 7,00 |  |  |  |  |  |  |  |  |  |  |  |  |  |  |  |  |  |  |  |  |  |  |  |  |  |  |  |  |  |  |  |  |  |
| **134.2** | 8,00 |  |  | 21,00 |  |  |  |  |  |  |  |  |  |  |  |  |  |  |  |  |  |  |  |  |  |  |  |  |  |  |  |  |  |  |  |  |  |
| **135 .1** | 36,00 | 213,00 | 36,00 |  |  |  |  | 17,00 |  | 2,00 |  | 6,00 |  |  |  |  |  |  |  |  |  |  |  |  |  |  |  |  |  |  |  |  |  |  |  |  |  |
| **135.2** | 46,00 | 204,00 | 39,00 |  |  |  |  | 25,00 |  | 0,00 |  | 0,00 |  |  |  |  |  |  |  |  |  |  |  |  |  |  |  |  |  |  |  |  |  |  |  |  |  |
| **136.1** |  |  |  |  |  |  |  | 32,00 |  |  |  |  |  |  |  |  |  |  |  |  |  |  | 162,00 | 0,00 | 1,00 |  |  |  |  |  |  |  |  |  |  |  |  |
| **136.2** |  |  |  |  |  |  |  | 40,00 |  |  |  |  |  |  |  |  |  |  |  |  |  |  | 22,00 | 36,00 | 134,00 |  |  |  |  |  |  |  |  |  |  |  |  |
| **136.3** |  |  |  |  |  |  |  | 39,00 |  |  |  |  |  |  |  |  |  |  |  |  |  |  | 125,00 | 8,00 | 10,00 |  |  |  |  |  |  |  |  |  |  |  |  |
| **136.4** |  |  |  |  |  |  |  | 9,00 |  |  |  |  |  |  |  |  |  |  |  |  |  |  | 13,00 | 5,00 | 5,00 |  |  |  |  |  |  |  |  |  |  |  |  |
| **136.5** |  |  |  |  |  |  |  | 5,00 |  |  |  |  |  |  |  |  |  |  |  |  |  |  | 11,00 | 3,00 | 7,00 |  |  |  |  |  |  |  |  |  |  |  |  |
| **137** |  |  | 1,00 | 7,00 |  |  |  | 3,00 |  |  |  |  |  |  |  |  |  |  |  |  |  |  |  |  |  |  |  |  |  |  |  |  |  |  |  |  |  |
| **138** |  | 31,00 |  |  |  |  |  |  |  |  |  |  |  |  |  |  |  |  |  |  |  |  |  |  |  |  |  |  |  |  |  |  |  |  |  |  |  |
| **139.1** | 12,00 | 21,00 | 0,00 | 8,00 |  |  |  | 15,00 | 1,00 | 1,00 |  | 4,00 |  |  |  |  |  |  |  |  |  |  |  |  | 31,00 |  |  |  |  |  |  |  |  |  |  |  |  |
| **139.2** | 35,00 | 52,00 | 1,00 | 14,00 |  |  |  | 35,00 | 2,00 | 2,00 |  | 5,00 |  |  |  |  |  |  |  |  |  |  |  |  | 47,00 |  |  |  |  |  |  |  |  |  |  |  |  |
| **139.3** | 152,00 | 1111,00 | 15,00 | 199,00 |  |  |  | 182,00 | 1,00 | 1,00 |  | 23,00 |  |  |  |  |  |  |  |  |  |  |  |  | 1203,00 |  |  |  |  |  |  |  |  |  |  |  |  |
| **139.4** | 256,00 | 2884,00 | 22,00 | 351,00 |  |  |  | 342,00 | 5,00 | 5,00 |  | 49,00 |  |  |  |  |  |  |  |  |  |  |  |  | 2514,00 |  |  |  |  |  |  |  |  |  |  |  |  |
| **140** | 167,00 |  |  |  |  | 5,00 | 5,00 | 4,00 | 2,00 |  |  |  |  |  |  |  |  |  |  |  |  |  |  |  |  |  |  |  |  |  |  |  |  |  |  |  |  |
| **141** |  | 126,00 |  |  |  | 14,00 |  | 344,00 |  |  |  |  |  |  |  |  |  |  |  |  |  |  |  |  |  |  |  |  |  |  |  |  |  |  |  |  |  |
| **142.1** |  | 2017,00 | 391,00 |  |  |  |  | 376,00 | 28,00 |  |  | 15,00 |  |  |  |  |  |  |  |  |  |  |  |  |  |  |  |  |  |  |  |  |  |  |  |  |  |
| **142.2** | 251,00 |  | 98,00 |  |  |  |  | 101,00 | 13,00 |  |  | 5,00 |  |  |  |  |  |  |  |  |  |  |  |  |  |  |  |  |  |  |  |  |  |  |  |  |  |
| **143.1** | 41,00 | 7,00 | 3,00 | 6,00 |  |  |  |  |  |  |  |  |  |  |  |  |  |  |  |  |  |  |  |  | 13,00 | 8,00 |  | 0,00 | 28,00 |  |  |  |  |  |  |  |  |
| **143.2** | 291,00 | 549,00 | 166,00 | 246,00 |  |  |  |  |  |  |  |  |  |  |  |  |  |  |  |  |  |  |  |  | 506,00 | 363,00 |  | 82,00 | 418,00 |  |  |  |  |  |  |  |  |
| **144.1** | 42,00 | 33,00 |  |  |  |  |  | 4,00 |  |  |  |  |  |  |  |  |  |  |  |  |  | 0,00 |  |  | 33,00 | 26,00 |  |  | 17,00 |  |  |  |  |  |  |  |  |
| **144.2** | 312,00 | 550,00 |  |  |  |  |  | 122,00 |  |  |  |  |  |  |  |  |  |  |  |  |  | 14,00 |  |  | 513,00 | 355,00 |  |  | 435,00 |  |  |  |  |  |  |  |  |
| **145** | 31,00 | 5,00 |  |  |  |  |  | 3,00 |  |  |  |  |  |  |  |  |  |  |  | 3,00 |  |  |  |  |  |  |  |  |  |  |  |  |  |  |  |  |  |
| **146** |  |  |  |  |  |  |  |  |  |  |  |  | 550,00 |  |  |  |  |  |  |  |  |  |  |  |  |  |  |  |  |  |  |  |  |  |  |  |  |
| **147** | 153,00 | 153,00 |  |  |  | 4,00 | 4,00 | 7,00 |  |  |  |  |  |  |  |  |  |  |  |  |  |  |  |  |  |  |  |  |  |  |  |  |  |  |  |  |  |
| **148 .1** |  |  |  |  |  |  |  |  |  |  |  |  |  |  |  |  |  |  |  |  |  |  |  |  |  |  |  |  |  |  |  |  |  |  |  |  |  |
| **148.2** |  |  |  |  |  |  |  |  |  |  |  |  |  |  |  |  |  |  |  |  |  |  |  |  |  |  |  |  |  |  |  |  |  |  |  |  |  |
| **149.1** | 4,00 |  |  | 18,00 |  |  |  |  |  |  |  |  |  |  |  |  |  |  |  |  |  |  |  |  |  |  |  |  |  |  |  |  |  |  |  |  |  |
| **149.2** | 44,00 |  |  | 10,00 |  |  |  |  |  |  |  |  |  |  |  |  |  |  |  |  |  |  |  |  |  |  |  |  |  |  |  |  |  |  |  |  |  |
| **149.3** | 4,00 |  |  | 15,00 |  |  |  |  |  |  |  |  | 355,00 | 3,00 |  |  |  |  |  |  |  |  |  |  |  |  |  |  |  |  |  |  |  |  |  |  |  |
| **149.4** | 4,00 |  |  | 2,00 |  |  |  |  |  |  |  |  | 390,00 | 2,00 |  |  |  |  |  |  |  |  |  |  |  |  |  |  |  |  |  |  |  |  |  |  |  |
| **150** | 56,00 | 11,00 |  |  |  |  |  | 78,00 |  |  |  |  |  |  |  |  |  |  |  |  |  |  |  |  |  |  |  |  |  |  |  |  |  |  |  |  |  |
| **151** |  |  |  |  |  |  |  |  |  |  |  |  |  |  |  |  |  |  |  |  |  |  |  |  |  |  |  |  |  |  |  |  |  |  |  |  |  |
| **152 .1** |  |  |  |  |  |  |  |  |  |  |  | 4,00 |  |  |  |  |  |  |  |  |  |  |  |  |  |  |  |  |  |  |  |  |  |  |  |  |  |
| **152.2** |  |  |  |  |  |  |  |  |  |  |  | 1,00 |  |  |  |  |  |  |  |  |  |  |  |  |  |  |  |  |  |  |  |  |  |  |  |  |  |
| **152.3** |  |  |  |  |  |  |  |  |  |  |  | 3,00 |  |  |  |  |  |  |  |  |  |  |  |  |  |  |  |  |  |  |  |  |  |  |  |  |  |
| **153.1** |  |  | 23,00 |  |  |  |  | 45,00 |  |  |  | 3,00 |  |  |  |  |  |  |  |  |  |  |  |  | 35,00 |  |  |  |  |  |  |  |  |  |  |  |  |
| **153.2** |  |  | 7,00 |  |  |  |  | 2,00 |  |  |  | 2,00 |  |  |  |  |  |  |  |  |  |  |  |  | 20,00 |  |  |  |  |  |  |  |  |  |  |  |  |
| **155.1** |  |  |  |  |  |  |  |  |  |  |  |  |  |  |  |  |  |  |  |  |  |  |  |  |  |  |  |  |  |  |  |  |  |  |  |  |  |
| **155.2** |  |  |  |  |  |  |  |  |  |  |  |  |  |  |  |  |  |  |  |  |  |  |  |  |  |  |  |  |  |  |  |  |  |  |  |  |  |
| **156.1** | 3,00 | 48,00 | 52,00 | 28,00 |  |  |  | 13,00 | 1,00 |  |  | 1,00 |  |  |  |  |  |  |  |  |  |  |  |  |  |  |  |  |  |  |  |  |  |  |  |  |  |
| **156.2** | 0,00 | 52,00 | 52,00 | 47,00 |  |  |  | 15,00 | 0,00 |  |  | 1,00 |  |  |  |  |  |  |  |  |  |  |  |  |  |  |  |  |  |  |  |  |  |  |  |  |  |
| **157.1** |  |  |  | 0,00 | 2,00 | 3,00 | 1,00 | 0,00 |  |  |  | 1,00 | 200,80 |  | 78,00 |  |  |  |  |  |  |  |  |  |  |  |  |  |  | 0,00 |  | 2,00 | 7,00 | 10,00 | 8,00 | 61,00 | 10,00 |
| **157.2** |  |  |  | 3,00 | 0,00 | 2,00 | 2,00 | 32,00 |  |  |  | 0,00 | 212,00 |  | 59,00 |  |  |  |  |  |  |  |  |  |  |  |  |  |  | 2,00 |  | 6,00 | 12,00 | 7,00 | 3,00 | 41,00 | 7,00 |
| **158.1** | 75,00 |  | 358,00 | 2204,00 |  |  |  | 446,00 |  |  |  |  |  |  |  |  |  |  |  |  |  |  |  |  |  |  |  |  |  |  |  |  |  |  |  |  |  |
| **158.2** | 695,00 |  | 818,00 | 2136,00 |  |  |  | 1023,00 |  |  |  |  |  |  |  |  |  |  |  |  |  |  |  |  |  |  |  |  |  |  |  |  |  |  |  |  |  |
| **160** | 0,00 |  |  |  |  |  |  |  |  |  |  | 1,00 | 395,00 |  | 3,00 | 1,00 | 3,00 |  |  |  |  |  |  |  |  |  |  |  |  |  |  |  |  |  |  |  |  |
| **161** | 11,00 |  | 6,00 | 3,00 |  |  |  | 3,00 | 1,00 |  |  | 1,00 |  |  |  |  |  |  | 0,00 |  |  | 8,00 |  |  |  |  |  |  |  |  |  |  |  |  |  |  |  |
| **162.1** | 14,00 | 39,00 | 14,00 | 5,00 |  |  |  |  |  |  |  |  |  |  |  |  |  |  |  |  |  |  |  |  |  |  |  |  |  |  |  |  |  |  |  |  |  |
| **162 .2** | 40,00 | 113,00 | 30,00 | 19,00 |  |  |  |  |  |  |  |  |  |  |  |  |  |  |  |  |  |  |  |  |  |  |  |  |  |  |  |  |  |  |  |  |  |
| **163.1** |  |  |  |  |  |  |  |  |  |  |  |  |  |  | 0,40 | 0,30 | 0,10 |  |  |  |  |  |  |  |  |  |  |  |  |  |  |  |  |  |  |  |  |
| **163.2** |  |  |  |  |  |  |  |  |  |  |  |  |  |  | 0,70 | 0,20 | 0,10 |  |  |  |  |  |  |  |  |  |  |  |  |  |  |  |  |  |  |  |  |
| **164** | 1,00 | 48,00 |  | 5,00 | 6,00 |  |  | 0,00 |  |  |  | 3,00 |  |  |  |  |  |  |  |  |  |  |  |  |  |  |  |  |  |  |  |  |  |  |  |  |  |
| **169.1** | 6,00 |  |  |  |  |  |  |  |  |  |  |  | 405,00 |  | 11,00 |  |  |  |  |  |  |  |  |  |  |  |  |  |  |  |  |  |  |  |  |  |  |
| **169.2** | 10,00 |  |  |  |  |  |  |  |  |  |  |  | 330,00 |  | 47,00 |  |  |  |  |  |  |  |  |  |  |  |  |  |  |  |  |  |  |  |  |  |  |
| **170.1** |  | 250,00 |  |  |  |  |  |  |  |  |  |  |  |  | 49,00 |  |  |  |  |  |  |  |  |  |  |  |  |  |  |  |  |  |  |  |  |  |  |
| **170.2** |  | 57,00 |  |  |  |  |  |  |  |  |  |  |  |  | 25,00 |  |  |  |  |  |  |  |  |  |  |  |  |  |  |  |  |  |  |  |  |  |  |
| **170.3** |  | 149,00 |  |  |  |  |  |  |  |  |  |  |  |  | 26,00 |  |  |  |  |  |  |  |  |  |  |  |  |  |  |  |  |  |  |  |  |  |  |
| **170.4** |  | 101,00 |  |  |  |  |  |  |  |  |  |  |  |  | 23,00 |  |  |  |  |  |  |  |  |  |  |  |  |  |  |  |  |  |  |  |  |  |  |
| **171.1** |  | 85,00 | 8,00 |  |  | 10,00 |  | 21,00 | 0,00 |  |  |  |  |  |  |  |  |  |  |  |  |  |  |  |  |  |  |  |  |  |  |  |  |  |  |  |  |
| **171.2** | 46,00 |  | 2,00 |  |  | 3,00 |  | 10,00 | 1,00 |  |  |  |  |  |  |  |  |  |  |  |  |  |  |  |  |  |  |  |  |  |  |  |  |  |  |  |  |
| **172.1** |  |  |  |  |  |  |  |  |  |  |  | 7,00 |  |  |  |  |  |  |  |  |  |  |  |  |  |  |  |  |  |  |  |  |  |  |  |  |  |
| **172.2** |  |  |  |  |  |  |  |  |  |  |  | 3,00 |  |  |  |  |  |  |  |  |  |  |  |  |  |  |  |  |  |  |  |  |  |  |  |  |  |
| **173** |  | 10,00 |  |  |  |  |  |  | 40,00 |  |  |  |  |  |  |  |  |  |  |  |  |  |  |  |  |  |  |  |  |  |  |  |  |  |  |  |  |
| **174** | 382,00 | 121,00 | 16,00 |  |  |  |  | 115,00 |  |  |  |  |  |  | 2,00 |  |  |  |  |  |  |  |  |  |  |  |  |  |  |  |  |  |  |  |  |  |  |
| **177** | 1840,00 | 5502,00 | 1301,00 | 994,00 |  |  |  | 1240,00 |  |  |  | 135,00 |  |  |  |  |  |  | 273,00 |  |  |  |  |  |  |  |  |  |  |  |  |  |  |  |  |  |  |
| **177.2** | 548,00 | 1202,00 | 250,00 | 270,00 |  |  |  |  |  |  |  | 39,00 |  |  |  |  |  |  | 126,00 |  |  |  |  |  |  |  |  |  |  |  |  |  |  |  |  |  |  |
| **177.3** | 662,00 | 3213,00 | 757,00 | 592,00 |  |  |  |  |  |  |  | 56,00 |  |  |  |  |  |  | 114,00 |  |  |  |  |  |  |  |  |  |  |  |  |  |  |  |  |  |  |
| **178** | 54,00 | 118,00 |  |  |  |  |  |  | 196,00 |  |  |  |  |  | 7,00 | 11,00 | 22,00 |  |  |  |  |  |  |  |  |  |  |  |  |  |  |  |  |  |  |  |  |
| **179** |  |  |  |  |  |  |  |  |  |  |  |  | 317,50 |  |  |  |  |  |  |  |  |  |  |  |  |  |  |  |  |  |  |  |  |  |  |  |  |
| **180** |  |  |  |  |  |  |  |  |  |  |  |  |  |  |  |  |  |  |  |  |  |  |  |  |  |  |  |  |  |  |  |  |  |  |  |  |  |
| **181** | 76,00 | 16,00 |  |  |  | 5,00 |  | 5,00 |  |  |  | 1,00 |  |  |  |  |  |  |  |  |  | 1,00 |  |  |  |  |  |  |  |  |  |  |  |  |  |  |  |
| **182** |  | 29,00 |  |  |  | 3,00 |  | 26,00 | 25,00 |  |  |  | 207,80 |  |  |  |  |  |  |  |  |  |  |  |  |  |  |  |  |  |  |  |  |  |  |  |  |
| **183.1** | 23,00 | 19,00 |  |  |  |  |  |  |  |  |  |  |  |  |  |  |  |  |  |  |  |  |  |  |  |  |  |  |  |  |  |  |  |  |  |  |  |
| **183.2** | 10,00 | 13,00 |  |  |  |  |  |  |  |  |  |  |  |  |  |  |  |  |  |  |  |  |  |  |  |  |  |  |  |  |  |  |  |  |  |  |  |
| **184.1** | 5,00 | 82,00 | 1,00 | 8,00 | 9,00 |  |  | 5,00 |  |  |  | 1,00 |  |  |  |  |  |  |  |  |  | 6,00 | 26,00 | 13,00 | 25,00 |  |  |  |  |  |  |  |  |  |  |  |  |
| **184.2** | 1,00 | 66,00 | 1,00 | 8,00 | 4,00 |  |  | 4,00 |  |  |  | 0,00 |  |  |  |  |  |  |  |  |  | 9,00 | 24,00 | 12,00 | 20,00 |  |  |  |  |  |  |  |  |  |  |  |  |
| **184.3** | 3,00 | 17,00 | 0,00 | 1,00 | 2,00 |  |  | 0,00 |  |  |  | 0,00 |  |  |  |  |  |  |  |  |  | 1,00 | 9,00 | 3,00 | 4,00 |  |  |  |  |  |  |  |  |  |  |  |  |
| **185** |  |  |  |  |  |  |  |  |  |  |  |  |  |  |  |  |  |  |  |  |  |  |  |  |  |  |  |  |  |  |  |  |  |  |  |  |  |
| **186.1** | 13,00 | 164,00 | 5,00 | 20,00 | 16,00 |  |  | 8,00 |  |  |  | 0,00 |  |  |  |  |  |  |  |  |  | 18,00 | 60,00 | 38,00 | 53,00 |  |  | 82,00 |  |  |  |  |  |  |  |  |  |
| **186.2** | 28,00 | 97,00 | 7,00 | 26,00 | 16,00 |  |  | 28,00 |  |  |  | 1,00 |  |  |  |  |  |  |  |  |  | 26,00 | 27,00 | 27,00 | 55,00 |  |  | 55,00 |  |  |  |  |  |  |  |  |  |
| **187 .1** |  |  | 198,00 | 206,00 |  |  |  |  |  |  |  |  |  |  |  |  |  |  |  |  |  |  |  |  |  |  |  |  |  |  |  |  |  |  |  |  |  |
| **187.2** |  |  | 203,00 | 190,00 |  |  |  |  |  |  |  |  |  |  |  |  |  |  |  |  |  |  |  |  |  |  |  |  |  |  |  |  |  |  |  |  |  |
| **188** |  |  | 14,00 |  |  |  |  |  |  |  |  |  | 437,00 |  | 36,00 |  |  |  |  |  |  |  | 62,00 | 11,00 | 30,00 |  |  |  |  |  |  |  |  |  |  |  |  |
| **189.1** | 73,00 | 4,00 |  |  |  |  |  | 13,00 |  |  |  |  | 513,10 |  |  |  |  |  |  |  |  |  |  |  |  |  |  |  |  |  |  |  |  |  |  |  |  |
| **189 .2** | 6,00 | 84,00 |  |  |  |  |  | 5,00 |  |  |  |  | 463,30 |  |  |  |  |  |  |  |  |  |  |  |  |  |  |  |  |  |  |  |  |  |  |  |  |
| **190.1** |  |  |  |  |  |  |  |  |  |  |  |  |  |  |  |  |  |  |  |  |  |  |  |  |  |  |  |  |  |  |  |  |  |  |  |  |  |
| **190 .2** |  |  |  |  |  |  |  |  |  |  |  |  |  |  |  |  |  |  |  |  |  |  |  |  |  |  |  |  |  |  |  |  |  |  |  |  |  |
| **191.1** |  |  |  |  |  |  |  |  |  |  |  |  |  |  |  |  |  |  |  |  |  |  |  |  |  |  |  |  |  |  |  |  |  |  |  |  |  |
| **191.2** |  |  |  |  |  |  |  |  |  |  |  |  |  |  |  |  |  |  |  |  |  |  |  |  |  |  |  |  |  |  |  |  |  |  |  |  |  |
| **191.3** |  |  |  |  |  |  |  |  |  |  |  |  |  |  |  |  |  |  |  |  |  |  |  |  |  |  |  |  |  |  |  |  |  |  |  |  |  |
| **192.1** |  |  |  |  |  |  |  |  |  |  |  |  |  |  |  |  |  |  |  |  |  |  |  |  |  |  |  |  |  |  |  |  |  |  |  |  |  |
| **192.2** |  |  |  |  |  |  |  |  |  |  |  |  |  |  |  |  |  |  |  |  |  |  |  |  |  |  |  |  |  |  |  |  |  |  |  |  |  |
| **193.1** |  | 51,00 |  |  |  |  |  |  | 158,00 |  |  |  |  |  | 0,00 |  |  |  |  |  |  |  |  |  |  |  |  |  |  |  |  |  |  |  |  |  |  |
| **193.2** |  | 106,00 |  |  |  |  |  |  | 261,00 |  |  | 9,00 |  |  | 0,00 |  |  |  |  |  |  |  |  |  |  |  |  |  |  |  |  |  |  |  |  |  |  |
| **194.1** |  | 42,00 | 5,00 | 4,00 | 0,00 |  |  | 1,00 |  |  |  |  |  |  |  |  |  |  |  |  |  |  |  |  |  |  |  |  |  |  |  |  |  |  |  |  |  |
| **194.2** |  | 48,00 | 6,00 | 1,00 | 2,00 |  |  | 0,00 |  |  |  |  |  |  |  |  |  |  |  |  |  |  |  |  |  |  |  |  |  |  |  |  |  |  |  |  |  |
| **195.1** | 178,00 | 511,00 | 83,00 |  |  | 20,00 |  |  |  |  |  |  |  | 5,00 |  |  |  |  |  |  |  |  |  |  |  |  |  |  |  |  |  |  |  |  |  |  |  |
| **195.2** | 32,00 | 54,00 | 20,00 |  |  | 5,00 |  | 86,00 |  |  |  |  |  | 6,00 |  |  |  |  |  |  |  |  |  |  |  |  |  |  |  |  |  |  |  |  |  |  |  |
| **196** |  | 116,00 | 39,00 |  |  |  |  | 51,00 |  |  |  | 4,00 |  |  | 102,00 |  |  |  | 1,00 |  |  |  |  |  |  |  |  |  |  |  |  |  |  |  |  |  |  |
| **197.1** |  | 11,00 |  |  |  | 4,00 |  | 4,00 |  |  |  |  |  |  |  |  |  |  |  |  |  |  |  |  |  |  |  |  |  |  |  |  |  |  |  |  |  |
| **197.2** |  | 11,00 |  |  |  | 1,00 |  | 4,00 |  |  |  |  |  |  |  |  |  |  |  |  |  |  |  |  |  |  |  |  |  |  |  |  |  |  |  |  |  |
| **198.1** |  | 255,00 |  |  |  |  |  |  |  |  |  |  |  |  | 16,00 |  |  |  |  |  |  |  |  |  |  |  |  |  |  |  |  |  |  |  |  |  |  |
| **198.2** |  | 252,00 |  |  |  |  |  |  |  |  |  |  |  |  | 35,00 |  |  |  |  |  |  |  |  |  |  |  |  |  |  |  |  |  |  |  |  |  |  |
| **199.1** |  |  |  |  |  |  |  |  |  |  |  |  |  | 1,00 |  |  |  |  |  |  |  |  |  |  | 39,00 | 31,00 |  | 2,00 | 5,00 |  |  |  |  |  |  |  |  |
| **199.2** |  |  |  |  |  |  |  |  |  |  |  |  |  |  |  |  |  |  |  |  |  |  |  |  |  |  |  |  |  |  |  |  |  |  |  |  |  |
| **199.3** |  |  |  |  |  |  |  |  |  |  |  |  |  |  |  |  |  |  |  |  |  |  |  |  |  |  |  |  |  |  |  |  |  |  |  |  |  |
| **199.4** |  |  |  |  |  |  |  |  |  |  |  |  |  |  |  |  |  |  |  |  |  |  |  |  |  |  |  |  |  |  |  |  |  |  |  |  |  |
| **199.5** |  |  |  |  |  |  |  |  |  |  |  |  |  |  |  |  |  |  |  |  |  |  |  |  |  |  |  |  |  |  |  |  |  |  |  |  |  |
| **200.1** |  |  | 2,00 |  |  |  |  | 7,00 |  |  |  |  | 338,20 |  |  |  |  |  |  |  |  |  |  |  |  |  |  |  |  |  |  |  |  |  |  |  |  |
| **200.2** |  |  | 19,00 |  |  |  |  | 20,00 |  |  |  |  | 434,10 |  |  |  |  |  |  |  |  |  |  |  |  |  |  |  |  |  |  |  |  |  |  |  |  |
| **201** | 57,00 | 391,00 | 143,00 | 84,00 | 40,00 | 3,00 | 1,00 |  |  |  |  | 12,00 |  |  |  |  |  |  |  |  |  | 29,00 | 289,00 | 87,00 |  |  | 107,00 | 104,00 | 400,00 |  |  |  |  |  |  |  |  |
| **202** | 115,00 | 73,00 | 11,00 |  |  | 3,00 |  | 56,00 |  |  |  | 2,00 |  |  |  |  |  |  |  |  |  |  |  |  |  |  |  |  |  |  |  |  |  |  |  |  |  |
| **203** |  |  |  |  |  |  |  |  |  |  |  |  |  |  |  |  |  |  |  |  |  |  |  |  |  |  |  |  |  |  |  |  |  |  |  |  |  |
| **204.1** |  |  | 37,00 |  |  |  |  | 20,00 |  |  |  | 2,00 |  |  |  |  |  |  |  |  |  |  | 127,00 | 58,00 | 29,00 |  |  |  |  |  |  |  |  |  |  |  |  |
| **204.2** |  |  | 23,00 |  |  |  |  | 2,00 |  |  |  | 1,00 |  |  |  |  |  |  |  |  |  |  | 131,00 | 33,00 | 8,00 |  |  |  |  |  |  |  |  |  |  |  |  |
| **205 .1** |  |  |  |  |  |  |  |  |  |  |  |  |  |  |  |  |  |  |  |  |  | 3,07 |  |  |  |  |  |  | 57,92 |  |  |  |  |  |  |  |  |
| **205.2** |  |  |  |  |  |  |  |  |  |  |  |  |  |  |  |  |  |  |  |  |  | 4,98 |  |  |  |  |  |  | 63,00 |  |  |  |  |  |  |  |  |
| **206** |  | 68,00 |  |  | 2,00 |  |  |  |  |  |  | 3,00 |  |  |  |  |  |  |  |  |  |  |  |  |  |  |  |  |  |  |  |  |  |  |  |  |  |
| **207** | 5016,62 | 7535,17 |  |  |  |  |  |  |  |  |  |  |  |  |  |  |  |  |  |  |  | 102,38 |  |  |  |  |  |  |  |  |  |  |  |  |  |  |  |
| **208** | 606,00 |  |  |  |  |  |  | 225,00 |  |  |  |  |  |  | 618,00 |  |  |  | 113,00 |  |  |  |  |  |  |  |  |  |  |  |  |  |  |  |  |  |  |
| **209.1** | 29,00 | 10,00 | 2,00 | 15,00 | 8,00 |  |  | 2,00 |  |  |  |  |  |  | 25,00 |  |  |  |  |  |  |  |  |  |  |  |  |  |  |  |  |  |  |  |  |  |  |
| **209.2** | 106,00 | 3,00 | 16,00 | 57,00 | 8,00 |  |  | 12,00 |  |  |  |  |  |  | 43,00 |  |  |  |  |  |  |  |  |  |  |  |  |  |  |  |  |  |  |  |  |  |  |
| **209.3** | 126,00 | 34,00 | 9,00 | 75,00 | 27,00 |  |  | 28,00 |  |  |  |  |  |  | 85,00 |  |  |  |  |  |  |  |  |  |  |  |  |  |  |  |  |  |  |  |  |  |  |
| **209.4** | 291,00 | 462,00 | 118,00 | 487,00 | 144,00 |  |  | 94,00 |  |  |  |  |  |  | 215,00 |  |  |  |  |  |  |  |  |  |  |  |  |  |  |  |  |  |  |  |  |  |  |
| **209.5** | 173,00 | 13,00 | 18,00 | 65,00 | 13,00 |  |  | 37,00 |  |  |  |  |  |  | 111,00 |  |  |  |  |  |  |  |  |  |  |  |  |  |  |  |  |  |  |  |  |  |  |
| **210** |  |  |  |  |  |  |  |  |  |  |  |  | 382,20 |  |  |  |  |  |  |  |  |  |  |  |  |  |  |  |  |  |  |  |  |  |  |  |  |
| **211.1** | 17,00 | 80,00 |  |  |  |  |  |  |  |  |  |  | 187,70 |  |  |  |  |  |  |  |  |  |  |  |  |  |  |  |  |  |  |  |  |  |  |  |  |
| **211.2** | 25,00 | 93,00 |  |  |  |  |  |  |  |  |  |  | 313,30 |  |  |  |  |  |  |  |  |  |  |  |  |  |  |  |  |  |  |  |  |  |  |  |  |
| **212** | 34,00 |  | 3,00 |  |  | 7,00 | 3,00 | 28,00 |  |  |  | 1,00 |  |  |  |  |  |  |  |  |  |  |  |  |  |  |  |  |  |  |  |  |  |  |  |  |  |
| **213** |  |  |  |  |  |  |  |  |  |  |  |  |  |  |  |  |  |  |  |  |  |  |  |  |  |  |  |  |  |  |  |  |  |  |  |  |  |
| **214** | 16,00 | 14,00 | 1,00 |  | 1,00 |  |  | 5,00 |  |  |  | 6,00 |  |  |  |  |  |  | 3,00 |  |  |  |  |  |  |  |  | 3,00 |  |  |  |  |  |  |  |  |  |
| **215** |  |  |  |  |  |  |  |  |  |  |  |  |  |  |  |  |  |  |  |  |  |  |  |  |  |  |  |  |  |  |  |  |  |  |  |  |  |
| **216** |  |  |  |  |  |  |  |  |  |  |  |  | 313,30 |  |  |  |  |  |  |  |  |  |  |  |  |  |  |  |  |  |  |  |  |  |  |  |  |
| **217** |  |  |  |  |  |  |  |  |  |  |  |  |  |  |  |  |  |  |  |  |  |  | 87,00 | 13,00 | 16,00 |  |  | 2,00 | 6,00 |  |  |  |  |  |  |  |  |
| **218** | 27,00 | 117,00 |  |  |  | 5,00 |  | 6,00 |  |  |  |  |  |  | 79,00 |  |  |  |  |  |  |  |  |  |  |  |  |  |  |  |  |  |  |  |  |  |  |
| **219.1** | 7,00 | 12,00 | 5,00 | 5,00 |  |  |  | 4,00 |  |  |  |  |  |  |  |  |  |  |  |  |  |  |  |  |  |  |  |  |  |  |  |  |  |  |  |  |  |
| **219 .2** | 2,00 | 8,00 | 3,00 | 3,00 |  |  |  | 2,00 |  |  |  |  |  |  |  |  |  |  |  |  |  |  |  |  |  |  |  |  |  |  |  |  |  |  |  |  |  |
| **220.1** | 91,00 | 98,00 | 43,00 | 73,00 |  |  |  | 41,00 |  |  |  |  |  | 3,00 | 2,00 | 0,00 | 0,00 |  |  |  |  |  |  |  | 98,00 | 70,00 |  | 15,00 | 83,00 |  |  |  |  |  |  |  |  |
| **220.2** | 57,00 | 57,00 | 34,00 | 55,00 |  |  |  | 27,00 |  |  |  |  |  | 1,00 | 2,00 | 1,00 | 0,00 |  |  |  |  |  |  |  | 50,00 | 34,00 |  | 7,00 | 51,00 |  |  |  |  |  |  |  |  |
| **220.3** | 28,00 | 32,00 | 9,00 | 16,00 |  |  |  | 9,00 |  |  |  |  |  | 0,00 | 2,00 | 0,00 | 0,00 |  |  |  |  |  |  |  | 35,00 | 26,00 |  | 8,00 | 27,00 |  |  |  |  |  |  |  |  |
| **220.4** | 6,00 | 9,00 | 0,00 | 2,00 |  |  |  | 5,00 |  |  |  |  |  | 2,00 | 2,00 | 0,00 | 0,00 |  |  |  |  |  |  |  | 13,00 | 10,00 |  | 0,00 | 6,00 |  |  |  |  |  |  |  |  |
| **220.5** | 63,00 | 128,00 | 57,00 | 76,00 |  |  |  | 26,00 |  |  |  |  |  | 2,00 | 0,00 | 0,00 | 0,00 |  |  |  |  |  |  |  | 113,00 | 84,00 |  | 17,00 | 97,00 |  |  |  |  |  |  |  |  |
| **221** |  |  |  |  |  |  |  |  |  |  |  |  | 120,00 |  |  |  |  |  |  |  |  |  |  |  |  |  |  |  |  |  |  |  |  |  |  |  |  |
| **222.1** |  |  |  |  |  |  |  |  |  |  |  |  |  |  | 26,00 |  |  |  |  |  |  |  |  |  |  |  |  |  |  |  |  |  |  |  |  |  |  |
| **222.2** |  |  |  |  |  |  |  |  |  |  |  |  |  |  |  |  |  |  |  |  |  |  |  |  |  |  |  |  |  |  |  |  |  |  |  |  |  |
| **223** | 20,00 |  |  |  |  |  |  | 13,00 | 20,00 |  |  | 0,00 |  |  |  |  |  |  | 2,00 |  |  |  |  |  |  |  |  |  |  |  |  |  |  |  |  |  |  |
| **224** | 8,00 | 3,00 | 11,00 | 11,00 |  |  |  | 2,00 |  |  |  |  |  |  |  |  |  |  |  |  |  |  |  |  |  |  |  |  |  |  |  |  |  |  |  |  |  |
| **225.1** |  |  |  |  |  |  |  |  |  |  |  |  |  |  |  |  |  |  |  |  |  |  |  |  |  |  |  |  |  |  |  |  |  |  |  |  |  |
| **225.2** |  |  |  |  |  |  |  |  |  |  |  |  |  |  |  |  |  |  |  |  |  |  |  |  |  |  |  |  |  |  |  |  |  |  |  |  |  |
| **Ʃ** | 19554,62 | 53621,77 | 8356,00 | 10953,80 | 913,00 | 273,00 | 17,00 | 8872,70 | 5318,00 | 74,00 |  | 637,00 |  | 37,00 | 4223,80 | 324,40 | 259,20 | 16,00 | 968,00 | 9,00 | 6,00 | 340,43 | 4340,00 | 1266,00 | 8491,00 | 1838,00 | 694,00 | 525,00 | 2811,92 | 66,00 |  | 38,00 | 76,00 | 50,30 | 53,10 | 245,40 | 59,20 |
| **mean** | 173,05 | 454,42 |  |  |  |  |  |  |  |  | 32,67 | 8,61 | 356,34 |  |  |  |  |  | 33,38 |  |  |  |  |  |  |  |  |  |  |  | 44,27 |  |  |  |  |  |  |
| **SD** | 549,26 | 1221,55 |  |  |  |  |  |  |  |  | 2,24 | 22,79 | 150,28 |  |  |  |  |  | 69,40 |  |  |  |  |  |  |  |  |  |  |  | 30,67 |  |  |  |  |  |  |

**Table 1.3:** Postoperative data; RvB= revision for bleeding, inhD= in-hospital death, TND= transient neurocognitive dysfunction, P= pneumonia, noAF= new-onset AF, MI= myocardial infarction, WI= wound infection, HD= haemodialysis, LOS= length of stay, 30dm= 30-day mortality, ID= intubation duration (hours), V= ventilation (min), pmV= prolonged mechanical ventilation (hours), ICU(h)= ICU stay in hours, ICU(d), ICU stay in days, ICU(n)= ICU stay number of patients, reIn= reintubation after extubation, reICU= readmission to ICU, RRT= renal replacement therapy, PM= pacemaker implantation, Crea= creatinine (mg/dl), HB= haemoglobin (g/l), AAT= alanine aminotransferase (U/l), sALb= serum albumin (g/l), tBili= total bilirubin (µmol/l), CK-MB= creatine kinase MB (ng/ml), rMR0= residual MR none, rMR1= residual MR mild, rMR2= residual MR moderate, rMR3= residual MR severe, rMS= residual mitral stenosis, AVR= aortic valve regurgitation, PMG= peak mitral gradient (mmHg), LVDV= left ventricular diastolic volume (cm^2^), LoC= line of coaptation (mm)

|  | **RvB** | | **inhD** | | | **TND** | | | **P** | | | **noAF** | | | **MI** | | | | **stroke** | | | | **WI** | | | **HD** | | | | **LOS** | | | | | **30dm** | | | | **ID** | | | **V** | | | | | **pmV** | **ICU (h)** | **ICU (d)** | **ICU (n)** | **reIn** | **reICU** | **RRT** | **PM** | **IABP** | **Impella** | **ECLS** | **crea** | **HB** | **AAT** | **sAlb** | **tBili** |
| --- | --- | --- | --- | --- | --- | --- | --- | --- | --- | --- | --- | --- | --- | --- | --- | --- | --- | --- | --- | --- | --- | --- | --- | --- | --- | --- | --- | --- | --- | --- | --- | --- | --- | --- | --- | --- | --- | --- | --- | --- | --- | --- | --- | --- | --- | --- | --- | --- | --- | --- | --- | --- | --- | --- | --- | --- | --- | --- | --- | --- | --- | --- |
| **1** | 29,00 | |  | | |  | | | 17,00 | | | 17,00 | | | 4,00 | | | | 12,00 | | | |  | | |  | | | | 7,10 | | | | | 2,00 | | | |  | | |  | | | | | 142,00 |  |  | 155,00 | 19,00 | 18,00 |  |  |  |  |  |  |  |  |  |  |
| **3** | 5,00 | |  | | |  | | |  | | | 8,00 | | | 1,00 | | | | 0,00 | | | |  | | |  | | | | 9,50 | | | | | 0,00 | | | | 4,00 | | |  | | | | | 3,00 |  | 1,00 |  |  |  | 1,00 | 12,00 |  |  |  |  |  |  |  |  |
| **4** | 22,00 | | 2,00 | | |  | | | 7,00 | | | 104,00 | | |  | | | | 2,00 | | | |  | | |  | | | | 8,00 | | | | |  | | | |  | | |  | | | | |  |  | 2,00 |  |  |  | 8,00 | 7,00 |  |  |  |  |  |  |  |  |
| **5** | 0,00 | |  | | |  | | | 5,00 | | |  | | |  | | | | 1,00 | | | | 0,00 | | |  | | | | 6,64 | | | | |  | | | | 13,26 | | |  | | | | | 1,00 |  | 2,49 |  |  |  | 1,00 |  | 2,00 |  |  |  |  |  |  |  |
| **7** | 0,00 | | 1,00 | | | 0,00 | | |  | | |  | | |  | | | |  | | | |  | | |  | | | | 6,20 | | | | |  | | | | 13,20 | | |  | | | | |  |  | 2,90 |  |  |  |  |  |  |  |  | 0,89 | 11,15 | 24,20 | 35,70 | 24,60 |
| **8** |  | | 83,00 | | |  | | |  | | |  | | |  | | | | 14,00 | | | |  | | |  | | | |  | | | | |  | | | | 13,30 | | |  | | | | |  |  |  |  |  |  | 292,00 |  |  |  |  |  |  |  |  |  |
| **9** | 1,00 | | 1,00 | | |  | | |  | | | 20,00 | | | 0,00 | | | | 7,00 | | | | 7,00 | | |  | | | |  | | | | |  | | | | 11,80 | | |  | | | | | 4,00 |  | 2,60 |  |  |  | 3,00 |  |  |  |  |  |  |  |  |  |
| **13** | 3,00 | | 0,00 | | |  | | |  | | |  | | |  | | | |  | | | |  | | |  | | | |  | | | | |  | | | |  | | |  | | | | |  |  |  |  |  |  |  | 3,00 |  |  |  |  |  |  |  |  |
| **14.1** | 5,00 | |  | | |  | | |  | | |  | | |  | | | |  | | | |  | | |  | | | | 7,89 | | | | |  | | | |  | | | 388,40 | | | | |  |  |  |  |  |  |  |  |  |  |  |  |  |  |  |  |
| **14.2** | 1,00 | |  | | |  | | |  | | |  | | |  | | | |  | | | |  | | |  | | | | 7,18 | | | | |  | | | |  | | | 297,06 | | | | |  |  |  |  |  |  |  |  |  |  |  |  |  |  |  |  |
| **14.3** | 1,00 | |  | | |  | | |  | | |  | | |  | | | |  | | | |  | | |  | | | | 6,73 | | | | |  | | | |  | | | 285,04 | | | | |  |  |  |  |  |  |  |  |  |  |  |  |  |  |  |  |
| **15.1** | 3,00 | |  | | | 3,00 | | | 4,00 | | | 32,00 | | |  | | | | 0,00 | | | | 1,00 | | |  | | | | 8,50 | | | | | 1,00 | | | |  | | |  | | | | |  |  |  |  |  |  | 0,00 | 1,00 |  |  |  | 0, 978 | 11,82 |  |  |  |
| **15.2** | 0,00 | |  | | | 1,00 | | | 0,00 | | | 15,00 | | |  | | | | 1,00 | | | | 0,00 | | |  | | | | 9,00 | | | | | 0,00 | | | |  | | |  | | | | |  |  |  |  |  |  | 1,00 | 0,00 |  |  |  | 1,02 | 11,86 |  |  |  |
| **18.1** | 0,00 | |  | | |  | | |  | | |  | | | 0,00 | | | | 1,00 | | | |  | | |  | | | | 4,90 | | | | | 0,00 | | | | 6,50 | | | 300,00 | | | | |  | 21,00 |  |  |  |  | 0,50 | 0,00 |  |  |  |  |  |  |  |  |
| **18.2** | 1,00 | |  | | | 0,00 | | |  | | |  | | | 0,00 | | | | 1,00 | | | |  | | |  | | | | 4,90 | | | | | 0,00 | | | | 6,00 | | | 350,00 | | | | |  | 22,00 |  |  |  |  | 0,00 | 0,00 |  |  |  |  |  |  |  |  |
| **18.3** | 0,00 | |  | | | 0,00 | | |  | | |  | | |  | | | | 0,00 | | | |  | | |  | | | | 5,20 | | | | |  | | | |  | | | 300,00 | | | | |  | 22,00 |  |  |  |  |  |  |  |  |  |  |  |  |  |  |
| **20** | 47,00 | |  | | |  | | |  | | |  | | | 5,00 | | | | 12,00 | | | | 59,00 | | |  | | | |  | | | | | 8,00 | | | |  | | |  | | | | |  |  |  | 615,00 |  |  |  | 12,00 | 2,00 |  | 5,00 |  |  |  |  |  |
| **21** | 2,00 | | 0,00 | | |  | | | 2,00 | | | 20,00 | | |  | | | | 0,00 | | | | 2,00 | | |  | | | | 8,00 | | | | |  | | | | 9,10 | | |  | | | | |  | 48,00 |  |  |  |  | 0,00 |  |  |  |  |  |  |  |  |  |
| **26** | 2,00 | | 1,00 | | |  | | |  | | |  | | |  | | | |  | | | |  | | | 1,00 | | | | 6,20 | | | | |  | | | | 13,20 | | |  | | | | | 3,00 | 69,60 |  |  |  |  | 1,00 |  |  |  |  |  |  |  |  |  |
| **29** | 6,00 | |  | | |  | | | 10,00 | | | 65,00 | | |  | | | | 4,00 | | | | 0,00 | | |  | | | |  | | | | |  | | | |  | | |  | | | | |  |  |  |  |  |  | 3,00 |  |  |  |  |  |  |  |  |  |
| **30** | 16,00 | | 4,00 | | |  | | |  | | |  | | | 3,00 | | | | 6,00 | | | |  | | |  | | | |  | | | | |  | | | |  | | |  | | | | |  |  |  |  |  |  | 8,00 |  |  |  |  |  |  |  |  |  |
| **31.1** | 2,00 | |  | | |  | | |  | | | 3,00 | | |  | | | | 1,00 | | | | 0,00 | | | 0,00 | | | |  | | | | |  | | | |  | | |  | | | | |  |  |  |  |  |  | 0,00 |  |  |  |  |  |  |  |  |  |
| **31.2** | 0,00 | |  | | |  | | |  | | | 0,00 | | |  | | | | 0,00 | | | | 1,00 | | | 1,00 | | | |  | | | | |  | | | |  | | |  | | | | |  |  |  |  |  |  | 1,00 |  |  |  |  |  |  |  |  |  |
| **35** |  | |  | | |  | | |  | | |  | | |  | | | |  | | | |  | | |  | | | |  | | | | |  | | | |  | | |  | | | | |  |  |  |  |  |  |  |  |  |  |  |  |  |  |  |  |
| **38** | 1,00 | | 0,00 | | |  | | | 0,00 | | |  | | |  | | | | 0,00 | | | | 0,00 | | | 0,00 | | | | 7,00 | | | | | 0,00 | | | |  | | | 246,00 | | | | |  | 18,90 |  |  |  |  |  |  |  |  |  |  |  |  |  |  |
| **40** | 9,00 | |  | | |  | | |  | | |  | | | 0,00 | | | | 1,00 | | | |  | | | 3,00 | | | | 9,00 | | | | | 2,00 | | | |  | | | 360,00 | | | | |  |  | 2,00 |  |  |  |  | 6,00 | 0,00 |  | 0,00 |  |  |  |  |  |
| **41** | 5,00 | |  | | |  | | | 1,00 | | | 9,00 | | | 0,00 | | | | 0,00 | | | | 0,00 | | |  | | | |  | | | | | 0,00 | | | |  | | | 491,50 | | | | |  | 24,00 |  |  |  | 2,00 | 2,00 |  |  |  |  |  |  |  |  |  |
| **42** | 2,00 | |  | | |  | | |  | | | 6,00 | | |  | | | | 0,00 | | | |  | | |  | | | |  | | | | | 0,00 | | | |  | | | 444,00 | | | | |  |  | 2,30 |  |  |  |  | 2,00 |  |  |  |  |  |  |  |  |
| **43** | 5,00 | |  | | |  | | |  | | |  | | |  | | | | 0,00 | | | | 0,00 | | | 1,00 | | | | 16,00 | | | | |  | | | |  | | | 2700,00 | | | | |  |  |  |  |  |  |  | 2,00 | 0,00 |  |  |  |  |  |  |  |
| **44.1** |  | | 0,00 | | |  | | |  | | |  | | |  | | | |  | | | |  | | |  | | | | 4,09 | | | | |  | | | |  | | | 291,00 | | | | |  | 34,00 |  |  |  |  |  |  |  |  |  |  |  |  |  |  |
| **44.2** |  | | 0,00 | | |  | | |  | | |  | | |  | | | |  | | | |  | | |  | | | | 4,25 | | | | |  | | | |  | | | 255,00 | | | | |  | 29,62 |  |  |  |  |  |  |  |  |  |  |  |  |  |  |
| **45** | 2,00 | | 0,00 | | |  | | |  | | | 21,00 | | | 0,00 | | | | 1,00 | | | | 0,00 | | |  | | | | 4,00 | | | | |  | | | |  | | |  | | | | |  | 24,00 |  |  |  |  | 0,00 |  |  |  |  |  |  |  |  |  |
| **46** |  | | 0,00 | | |  | | |  | | |  | | |  | | | |  | | | |  | | |  | | | | 8,70 | | | | |  | | | |  | | |  | | | | |  | 41,40 |  |  |  |  |  |  |  |  |  |  |  |  |  |  |
| **47** | 2,00 | | 0,00 | | |  | | | 0,00 | | | 2,00 | | |  | | | | 1,00 | | | |  | | | 0,00 | | | | 5,00 | | | | |  | | | |  | | | 510,00 | | | | |  |  | 1,00 |  |  |  | 0,00 | 0,00 |  |  |  |  |  |  |  |  |
| **48** | 5,00 | |  | | |  | | |  | | |  | | | 0,00 | | | | 1,00 | | | |  | | | 1,00 | | | |  | | | | | 4,00 | | | |  | | | 708,00 | | | | |  |  | 1,00 |  | 2,00 |  |  | 1,00 |  |  |  |  |  |  |  |  |
| **51** | 1,00 | | 0,00 | | |  | | |  | | | 1,00 | | |  | | | |  | | | | 0,00 | | |  | | | | 6,90 | | | | | 0,00 | | | | 9,70 | | |  | | | | |  | 37,40 |  |  |  |  |  | 0,00 |  |  |  |  |  |  |  |  |
| **53.1** | 10,00 | | 1,00 | | |  | | |  | | | 46,00 | | | 1,00 | | | | 1,00 | | | |  | | | 9,00 | | | | 6,00 | | | | | 3,00 | | | |  | | | 300,00 | | | | |  | 48,00 |  |  |  |  |  | 1,00 | 1,00 |  |  |  |  |  |  |  |
| **53.2** | 4,00 | | 0,00 | | |  | | |  | | | 11,00 | | | 0,00 | | | | 0,00 | | | |  | | | 0,00 | | | | 6,00 | | | | | 0,00 | | | |  | | | 300,00 | | | | |  | 48,00 |  |  |  |  |  |  | 0,00 |  |  |  |  |  |  |  |
| **53.3** | 2,00 | | 0,00 | | |  | | |  | | | 19,00 | | | 1,00 | | | | 0,00 | | | |  | | | 6,00 | | | | 6,00 | | | | | 0,00 | | | |  | | | 360,00 | | | | |  | 48,00 |  |  |  |  |  |  | 1,00 |  |  |  |  |  |  |  |
| **55.1** |  | |  | | |  | | | 8,00 | | |  | | |  | | | |  | | | |  | | |  | | | |  | | | | |  | | | | 8,78 | | |  | | | | |  | 59,89 |  |  |  |  |  |  |  |  |  | 1,05 | 11,11 | 34,01 |  |  |
| **55.2** |  | |  | | |  | | | 4,00 | | |  | | |  | | | |  | | | |  | | |  | | | |  | | | | |  | | | | 5,31 | | |  | | | | |  | 49,41 |  |  |  |  |  |  |  |  |  | 0,93 | 10,81 | 30,39 |  |  |
| **56.1** | 0,00 | | 0,00 | | |  | | |  | | | 3,00 | | | 0,00 | | | | 0,00 | | | | 2,00 | | |  | | | | 8,00 | | | | |  | | | |  | | | 589,00 | | | | |  | 19,70 |  |  |  |  |  |  |  |  |  |  |  |  |  |  |
| **56.2** | 1,00 | | 0,00 | | |  | | |  | | | 5,00 | | | 0,00 | | | | 0,00 | | | | 0,00 | | |  | | | | 8,00 | | | | |  | | | |  | | | 497,00 | | | | |  | 20,25 |  |  |  |  |  |  |  |  |  |  |  |  |  |  |
| **57** | 2,00 | |  | | |  | | |  | | | 9,00 | | | 0,00 | | | | 0,00 | | | | 0,00 | | |  | | | | 8,00 | | | | |  | | | |  | | |  | | | | |  | 22,00 |  |  |  |  | 2,00 |  |  |  |  |  |  |  |  |  |
| **63.1** |  | | 1,00 | | |  | | |  | | | 16,00 | | |  | | | |  | | | |  | | |  | | | | 5,00 | | | | |  | | | |  | | |  | | | | |  |  |  |  |  |  |  |  | 4,00 |  |  |  |  |  |  |  |
| **63.2** |  | | 0,00 | | |  | | |  | | | 19,00 | | |  | | | |  | | | |  | | |  | | | | 5,00 | | | | |  | | | |  | | |  | | | | |  |  |  |  |  |  |  |  | 3,00 |  |  |  |  |  |  |  |
| **65** | 7,00 | |  | | |  | | |  | | |  | | | 3,00 | | | | 3,00 | | | | 0,00 | | |  | | | |  | | | | | 4,00 | | | |  | | |  | | | | |  | 20,00 |  |  |  |  |  | 4,00 |  |  |  |  |  |  |  |  |
| **66** | 380,00 | | 107,00 | | | 372,00 | | |  | | | 1005,00 | | | 54,00 | | | | 79,00 | | | | 97,00 | | | 103,00 | | | | 8,00 | | | | |  | | | | 8,00 | | |  | | | | |  | 24,00 |  |  | 225,00 |  | 366,00 | 454,00 |  |  |  |  |  |  |  |  |
| **67** | 21,00 | |  | | |  | | |  | | |  | | |  | | | |  | | | |  | | |  | | | |  | | | | |  | | | |  | | |  | | | | |  |  |  |  |  |  |  | 20,00 |  |  |  | 0,96 |  |  |  |  |
| **68** | 10,00 | |  | | |  | | | 21,00 | | | 6,00 | | |  | | | | 2,00 | | | |  | | | 3,00 | | | | 13,00 | | | | | 4,00 | | | |  | | |  | | | | | 7,00 |  | 1,30 |  |  |  | 13,00 |  |  |  |  |  |  |  |  |  |
| **71** | 11,00 | |  | | | 0,00 | | | 4,00 | | | 26,00 | | |  | | | | 2,00 | | | | 0,00 | | |  | | | |  | | | | |  | | | |  | | |  | | | | | 2,00 |  |  |  | 1,00 | 2,00 |  |  |  |  |  |  |  |  |  |  |
| **72** | 23,00 | |  | | |  | | | 15,00 | | | 162,00 | | | 3,00 | | | | 4,00 | | | |  | | |  | | | | 6,00 | | | | | 5,00 | | | |  | | |  | | | | | 6,00 |  |  |  | 3,00 | 8,00 | 3,00 |  |  |  |  |  |  |  |  |  |
| **73** | 8,00 | |  | | | 0,00 | | | 3,00 | | | 17,00 | | | 2,00 | | | | 1,00 | | | | 0,00 | | |  | | | | 7,00 | | | | | 2,00 | | | |  | | |  | | | | | 0,00 |  |  |  | 0,00 | 3,00 | 3,00 |  |  |  |  |  |  |  |  |  |
| **74** | 4,00 | | 0,00 | | |  | | | 0,00 | | | 61,00 | | |  | | | | 0,00 | | | |  | | | 0,00 | | | | 5,00 | | | | | 0,00 | | | |  | | | 264,00 | | | | | 4,00 | 25,00 |  |  | 3,00 |  | 0,00 | 1,00 |  |  |  |  |  |  |  |  |
| **75** | 1,00 | |  | | |  | | | 1,00 | | | 24,00 | | |  | | | |  | | | | 2,00 | | |  | | | | 4,00 | | | | |  | | | |  | | |  | | | | | 0,00 |  |  |  |  | 8,00 |  |  |  |  |  |  |  |  |  |  |
| **77** | 2,00 | | 0,00 | | |  | | | 1,00 | | | 15,00 | | |  | | | | 1,00 | | | | 0,00 | | | 1,00 | | | | 5,50 | | | | |  | | | | 3,70 | | |  | | | | | 4,00 |  |  |  |  | 7,00 | 1,00 |  |  |  |  |  |  |  |  |  |
| **78.1** | 3,00 | | 1,00 | | |  | | |  | | | 17,00 | | | 0,00 | | | | 0,00 | | | |  | | |  | | | | 5,00 | | | | |  | | | |  | | |  | | | | |  |  | 1,00 |  |  |  | 0,00 |  | 0,00 |  |  |  |  |  |  |  |
| **78.2** | 1,00 | | 0,00 | | |  | | |  | | | 33,00 | | | 2,00 | | | | 2,00 | | | |  | | |  | | | | 5,00 | | | | |  | | | |  | | |  | | | | |  |  | 1,00 |  |  |  | 0,00 |  | 0,00 |  |  |  |  |  |  |  |
| **79** | 5,00 | |  | | |  | | |  | | |  | | | 1,00 | | | | 0,00 | | | | 1,00 | | | 4,00 | | | | 8,00 | | | | | 1,00 | | | |  | | |  | | | | | 8,00 |  | 1,00 |  |  |  |  | 2,00 |  |  |  |  |  |  |  |  |
| **80** |  | |  | | |  | | |  | | |  | | | 0,00 | | | | 0,00 | | | | 0,00 | | | 3,00 | | | | 8,00 | | | | | 2,00 | | | |  | | |  | | | | | 30,00 |  | 1,00 |  |  |  |  | 1,00 |  |  |  |  |  |  |  |  |
| **82** | 3,00 | | 1,00 | | |  | | | 2,00 | | | 34,00 | | | 1,00 | | | | 5,00 | | | | 0,00 | | | 1,00 | | | | 14,00 | | | | |  | | | |  | | |  | | | | | 13,00 |  | 2,00 |  |  |  |  | 8,00 |  |  |  |  |  |  |  |  |
| **83** |  | |  | | |  | | |  | | |  | | |  | | | |  | | | |  | | |  | | | |  | | | | |  | | | |  | | |  | | | | |  |  |  |  |  |  |  |  |  |  |  |  |  |  |  |  |
| **86** |  | |  | | |  | | |  | | |  | | |  | | | |  | | | |  | | |  | | | |  | | | | |  | | | |  | | |  | | | | |  |  |  |  |  |  |  |  |  |  |  |  |  |  |  |  |
| **87.1** |  | |  | | | 4,00 | | | 3,00 | | | 1,00 | | |  | | | | 1,00 | | | |  | | |  | | | | 6,00 | | | | |  | | | |  | | | 62,00 | | | | |  |  | 7,00 |  |  |  |  |  |  |  |  |  |  |  |  |  |
| **87.2** |  | |  | | | 2,00 | | | 2,00 | | |  | | |  | | | | 1,00 | | | |  | | |  | | | | 8,00 | | | | |  | | | |  | | | 60,00 | | | | |  |  | 5,00 |  |  |  |  |  |  |  |  |  |  |  |  |  |
| **93** |  | |  | | |  | | | 1,00 | | |  | | |  | | | |  | | | | 3,00 | | |  | | | |  | | | | |  | | | |  | | |  | | | | |  |  |  |  |  |  | 1,00 |  |  |  |  |  |  |  |  |  |
| **98** | 0,00 | | 0,00 | | |  | | | 10,00 | | |  | | |  | | | | 0,00 | | | |  | | |  | | | | 5,90 | | | | |  | | | |  | | | 762,00 | | | | |  | 38,70 |  |  |  |  | 1,00 |  |  |  |  |  |  |  |  |  |
| **99.1** |  | |  | | |  | | |  | | |  | | |  | | | |  | | | |  | | |  | | | |  | | | | |  | | | |  | | |  | | | | |  |  |  |  |  |  |  |  |  |  |  |  |  |  |  |  |
| **99.2** |  | |  | | |  | | |  | | |  | | |  | | | |  | | | |  | | |  | | | |  | | | | |  | | | |  | | |  | | | | |  |  |  |  |  |  |  |  |  |  |  |  |  |  |  |  |
| **101.1** | 2,00 | |  | | | 2,00 | | | 2,00 | | | 10,00 | | | 0,00 | | | | 1,00 | | | | 1,00 | | | 1,00 | | | | 8,00 | | | | | 1,00 | | | |  | | | 720,00 | | | | |  |  | 2,00 |  |  |  |  | 4,00 |  |  | 0,00 |  |  |  |  |  |
| **101.2** | 11,00 | |  | | | 19,00 | | | 11,00 | | | 22,00 | | | 1,00 | | | | 3,00 | | | | 4,00 | | | 10,00 | | | | 12,00 | | | | | 9,00 | | | |  | | | 720,00 | | | | |  |  | 3,00 |  |  |  |  | 6,00 |  |  | 2,00 |  |  |  |  |  |
| **102** |  | |  | | |  | | |  | | |  | | | 3,00 | | | | 7,00 | | | |  | | | 33,00 | | | | 14,00 | | | | | 12,00 | | | |  | | |  | | | | |  |  | 1,00 |  |  |  |  | 22,00 | 3,00 |  | 2,00 |  |  |  |  |  |
| **103** | 2,00 | | 1,00 | | |  | | | 11,00 | | | 74,00 | | | 2,00 | | | | 5,00 | | | | 1,00 | | | 6,00 | | | | 13,00 | | | | | 1,00 | | | |  | | | 1367,40 | | | | |  |  | 1,00 |  |  |  |  | 1,00 |  |  | 0,00 |  |  |  |  |  |
| **104** | 9,00 | | 0,00 | | |  | | |  | | | 9,00 | | |  | | | | 0,00 | | | | 0,00 | | |  | | | | 6,00 | | | | |  | | | |  | | |  | | | | |  |  | 1,00 |  |  | 2,00 | 1,00 | 2,00 |  |  |  |  |  |  |  |  |
| **105** | 29,00 | | 12,00 | | |  | | |  | | |  | | |  | | | |  | | | |  | | | 6,00 | | | | 6,00 | | | | |  | | | |  | | |  | | | | |  |  |  |  |  |  |  |  |  |  |  |  |  |  |  |  |
| **107** | 1,00 | | 1,00 | | |  | | |  | | | 10,00 | | |  | | | | 1,00 | | | | 1,00 | | |  | | | | 6,20 | | | | |  | | | |  | | |  | | | | | 16,00 | 37,60 |  |  | 2,00 |  | 1,00 |  |  |  |  |  |  |  |  |  |
| **108** | 1,00 | | 1,00 | | |  | | |  | | |  | | |  | | | | 1,00 | | | | 1,00 | | | 1,00 | | | | 8,10 | | | | |  | | | |  | | | 258,00 | | | | |  |  | 2,70 |  |  |  |  |  |  |  |  |  |  |  |  |  |
| **110** | 1,00 | |  | | |  | | |  | | | 31,00 | | | 3,00 | | | | 1,00 | | | | 0,00 | | | 1,00 | | | | 7,00 | | | | | 1,00 | | | |  | | | 300,00 | | | | |  |  |  |  |  |  |  | 8,00 |  |  |  |  |  |  |  |  |
| **111** | 17,00 | |  | | |  | | |  | | | 78,00 | | | 6,00 | | | | 3,00 | | | | 7,00 | | |  | | | | 8,00 | | | | | 1,00 | | | |  | | | 240,00 | | | | |  |  |  |  |  |  |  | 16,00 |  |  |  |  |  |  |  |  |
| **112** | 6,00 | |  | | |  | | |  | | | 33,00 | | |  | | | | 1,00 | | | | 4,00 | | |  | | | | 11,70 | | | | |  | | | |  | | | 384,00 | | | | |  | 58,80 |  |  |  |  |  | 8,00 |  |  |  |  |  |  |  |  |
| **113.1** | 2,00 | | 0,00 | | |  | | |  | | | 9,00 | | |  | | | | 0,00 | | | |  | | |  | | | | 6,00 | | | | |  | | | |  | | |  | | | | |  |  | 3,00 |  |  |  | 1,00 |  |  |  |  |  |  |  |  |  |
| **113.2** | 4,00 | | 0,00 | | |  | | |  | | | 5,00 | | |  | | | | 0,00 | | | |  | | |  | | | | 6,00 | | | | |  | | | |  | | |  | | | | |  |  | 2,00 |  |  |  | 0,00 |  |  |  |  |  |  |  |  |  |
| **115.1** | 26,00 | | 3,00 | | |  | | |  | | |  | | |  | | | | 2,00 | | | |  | | |  | | | |  | | | | |  | | | |  | | |  | | | | |  |  |  |  |  |  |  |  |  |  |  |  |  |  |  |  |
| **115.2** | 12,00 | | 1,00 | | |  | | |  | | |  | | |  | | | | 1,00 | | | |  | | |  | | | |  | | | | |  | | | |  | | |  | | | | |  |  |  |  |  |  |  |  |  |  |  |  |  |  |  |  |
| **115.3** | 2,00 | | 0,00 | | |  | | |  | | |  | | |  | | | | 0,00 | | | |  | | |  | | | |  | | | | |  | | | |  | | |  | | | | |  |  |  |  |  |  |  |  |  |  |  |  |  |  |  |  |
| **116.1** | | | 4,00 | | | |  | | | 2,00 | | | 32,00 | | |  | | | | 2,00 | | | | 1,00 | | |  | | | |  | | | | |  | | | | |  | | |  | | |  |  |  |  |  | 4,00 |  | 1,00 |  |  |  |  |  |  |  |  |
| **116.2** |  | | 5,00 | | |  | | | 4,00 | | | 31,00 | | |  | | | | 2,00 | | | | 0,00 | | |  | | | |  | | | | |  | | | |  | | |  | | | | |  |  |  |  |  | 5,00 |  | 7,00 |  |  |  |  |  |  |  |  |
| **117.1** | 1,00 | | 1,00 | | |  | | |  | | |  | | |  | | | | 1,00 | | | | 0,00 | | |  | | | | 7,02 | | | | |  | | | |  | | |  | | | | |  | 18,51 |  |  |  |  | 2,00 |  |  |  |  |  |  |  |  |  |
| **117.2** | 1,00 | | 0,00 | | |  | | |  | | |  | | |  | | | | 0,00 | | | | 2,00 | | |  | | | | 5,45 | | | | |  | | | |  | | |  | | | | |  | 16,44 |  |  |  |  | 1,00 |  |  |  |  |  |  |  |  |  |
| **118** |  | |  | | |  | | | 11,00 | | | 111,00 | | |  | | | | 16,00 | | | |  | | |  | | | |  | | | | |  | | | |  | | |  | | | | |  |  |  |  |  |  | 6,00 | 14,00 |  |  |  |  |  |  |  |  |
| **119.1** |  | |  | | |  | | |  | | |  | | |  | | | |  | | | |  | | |  | | | |  | | | | |  | | | |  | | |  | | | | |  |  |  |  |  |  |  |  |  |  |  |  |  |  |  |  |
| **119.2** |  | |  | | |  | | |  | | |  | | |  | | | |  | | | |  | | |  | | | |  | | | | |  | | | |  | | |  | | | | |  |  |  |  |  |  |  |  |  |  |  |  |  |  |  |  |
| **121.1** | 0,00 | | 0,00 | | |  | | |  | | | 23,00 | | | 0,00 | | | | 2,00 | | | |  | | | 1,00 | | | | 7,40 | | | | |  | | | |  | | |  | | | | | 4,00 |  | 1,60 |  |  |  |  |  |  |  |  |  |  |  |  |  |
| **121.2** | 1,00 | | 2,00 | | |  | | |  | | | 12,00 | | | 0,00 | | | | 0,00 | | | |  | | | 1,00 | | | | 6,80 | | | | |  | | | |  | | |  | | | | | 1,00 |  | 1,30 |  |  |  |  |  |  |  |  |  |  |  |  |  |
| **122.1** | 4,00 | | 0,00 | | | 4,00 | | | 2,00 | | | 12,00 | | | 9,00 | | | | 1,00 | | | | 0,00 | | |  | | | | 7,00 | | | | |  | | | |  | | |  | | | | |  |  |  |  |  |  |  | 2,00 |  |  |  |  | 12,30 |  |  |  |
| **122.2** | 1,00 | | 0,00 | | | 0,00 | | | 0,00 | | | 12,00 | | | 1,00 | | | | 0,00 | | | | 1,00 | | |  | | | | 7,00 | | | | |  | | | |  | | |  | | | | |  |  |  |  |  |  |  | 1,00 |  |  |  |  | 11,90 |  |  |  |
| **123.1** | 1,00 | |  | | |  | | | 3,00 | | |  | | |  | | | | 1,00 | | | |  | | |  | | | |  | | | | | 0,00 | | | |  | | |  | | | | |  |  |  |  |  | 8,00 |  |  |  |  |  |  |  |  |  |  |
| **123.2** | 3,00 | |  | | |  | | | 3,00 | | |  | | |  | | | | 0,00 | | | |  | | |  | | | |  | | | | | 1,00 | | | |  | | |  | | | | |  |  |  |  |  | 1,00 |  |  |  |  |  |  |  |  |  |  |
| **127** | 9,00 | |  | | |  | | | 2,00 | | |  | | |  | | | |  | | | |  | | |  | | | | 11,00 | | | | | 3,00 | | | |  | | |  | | | | |  |  | 2,00 |  | 3,00 |  |  | 2,00 |  |  | 1,00 |  |  |  |  |  |
| **128** | 5,00 | |  | | | 0,00 | | | 11,00 | | | 19,00 | | |  | | | |  | | | | 4,00 | | | 1,00 | | | | 5,00 | | | | | 1,00 | | | |  | | |  | | | | |  |  | 1,00 |  |  |  |  |  |  |  |  |  |  |  |  |  |
| **129** | 10,00 | |  | | |  | | |  | | |  | | |  | | | |  | | | |  | | | 2,00 | | | |  | | | | | 1,00 | | | |  | | |  | | | | |  |  | 1,00 |  |  |  |  | 10,00 |  |  | 2,00 |  |  |  |  |  |
| **130** | 4,00 | | 2,00 | | |  | | |  | | |  | | |  | | | | 1,00 | | | |  | | | 1,00 | | | | 8,00 | | | | |  | | | | 6,00 | | |  | | | | |  |  |  |  |  |  |  |  |  |  |  |  |  |  |  |  |
| **131.1** | 3,00 | |  | | |  | | | 5,00 | | | 38,00 | | |  | | | | 2,00 | | | |  | | | 3,00 | | | |  | | | | | 1,00 | | | |  | | | 732,00 | | | | |  |  | 1,60 |  |  |  |  | 2,00 |  |  |  |  |  |  |  |  |
| **131.2** | 3,00 | |  | | |  | | | 5,00 | | | 49,00 | | |  | | | | 5,00 | | | |  | | | 3,00 | | | |  | | | | | 0,00 | | | |  | | | 756,00 | | | | |  |  | 2,07 |  |  |  |  | 5,00 |  |  |  |  |  |  |  |  |
| **132 .1** | 9,00 | | 1,00 | | |  | | |  | | |  | | |  | | | | 4,00 | | | |  | | |  | | | | 6,00 | | | | |  | | | |  | | |  | | | | | 4,00 |  |  |  |  |  | 3,00 | 3,00 |  |  |  |  |  |  |  |  |
| **132 .2** | 27,00 | | 3,00 | | |  | | |  | | |  | | |  | | | | 6,00 | | | |  | | |  | | | | 5,00 | | | | |  | | | |  | | |  | | | | | 20,00 |  |  |  |  |  | 6,00 | 21,00 |  |  |  |  |  |  |  |  |
| **133** |  | | 2,00 | | |  | | | 10,00 | | | 20,00 | | |  | | | | 0,00 | | | |  | | |  | | | | 9,50 | | | | | 1,00 | | | |  | | | 932,00 | | | | |  |  |  |  |  |  |  |  |  |  |  |  |  |  |  |  |
| **134.1** |  | |  | | |  | | |  | | |  | | |  | | | |  | | | |  | | |  | | | |  | | | | |  | | | |  | | |  | | | | |  |  |  |  |  |  |  |  | 2,00 |  |  |  |  |  |  |  |
| **134.2** |  | |  | | |  | | |  | | |  | | |  | | | |  | | | |  | | |  | | | |  | | | | |  | | | |  | | |  | | | | |  |  |  |  |  |  |  |  | 0,00 |  |  |  |  |  |  |  |
| **135 .1** | 28,00 | | 4,00 | | | 2,00 | | | 23,00 | | |  | | | 10,00 | | | | 2,00 | | | | 0,00 | | | 8,00 | | | | 9,00 | | | | |  | | | |  | | |  | | | | |  |  |  | 1,00 |  |  |  | 6,00 |  |  |  |  |  |  |  |  |
| **135.2** | 8,00 | | 2,00 | | | 3,00 | | | 15,00 | | |  | | | 3,00 | | | | 2,00 | | | | 1,00 | | | 7,00 | | | | 8,00 | | | | |  | | | |  | | |  | | | | |  |  |  | 1,00 |  |  |  | 8,00 |  |  |  |  |  |  |  |  |
| **136.1** | | | | 0,00 | |  | | |  | | | 22,00 | | | 2,00 | | | | 0,00 | | | | 1,00 | | |  | | |  | | | |  | | | | |  | | |  | | | |  | |  |  |  |  |  |  | 2,00 | 2,00 |  |  |  |  |  |  |  |  |
| **136.2** |  | | 0,00 | |  | | | |  | | | 32,00 | | | 2,00 | | | | 1,00 | | | | 3,00 | | |  | | |  | | | |  | | | | |  | | |  | | | |  | |  |  |  |  |  |  | 0,00 | 8,00 |  |  |  |  |  |  |  |  |
| **136.3** | | | 0,00 | |  | | | |  | | | 24,00 | | | 1,00 | | | | 0,00 | | | | 2,00 | | |  | | |  | | | |  | | | | |  | | |  | | | |  | |  |  |  |  |  |  | 2,00 | 10,00 |  |  |  |  |  |  |  |  |
| **136.4** |  | | 0,00 | |  | | |  | | | 6,00 | | | 0,00 | | | | 0,00 | | | | 0,00 | | |  | | |  | | | |  | | | | |  | | |  | | | |  | | |  |  |  |  |  |  | 0,00 | 1,00 |  |  |  |  |  |  |  |  |
| **136.5** | | | | 0,00 | |  | | |  | | | 4,00 | | | 0,00 | | | | 0,00 | | | | 0,00 | | |  | | |  | | | |  | | | | |  | | |  | | | |  | |  |  |  |  |  |  | 0,00 | 2,00 |  |  |  |  |  |  |  |  |
| **137** | |  |  | | | |  | | |  | | |  | | | |  | | | |  | | | |  | | | | |  | | | |  | | | | |  | | | |  | | |  |  |  |  |  |  |  |  |  |  |  |  |  |  |  |  |  |
| **138** | |  |  | | | |  | | |  | | | 7,00 | | | |  | | | | 1,00 | | | | 0,00 | | | | |  | | | | 7,00 | | | | | 0,00 | | | |  | | |  |  |  | 1,40 |  |  |  | 1,00 | 3,00 |  |  |  |  |  |  |  |  |
| **139.1** | | 5,00 | 3,00 | | | | 4,00 | | |  | | | 2,00 | | | | 1,00 | | | | 0,00 | | | | 0,00 | | | | | 2,00 | | | | 10,00 | | | | |  | | | |  | | |  | 7,00 |  | 1,29 |  |  |  | 4,00 | 1,00 |  |  |  |  |  |  |  |  |
| **139.2** | | 5,00 | 10,00 | | | | 7,00 | | |  | | | 10,00 | | | | 0,00 | | | | 2,00 | | | | 0,00 | | | | | 5,00 | | | | 12,00 | | | | |  | | | |  | | |  | 12,00 |  | 1,08 |  |  |  | 14,00 | 8,00 |  |  |  |  |  |  |  |  |
| **139.3** | | 72,00 | 14,00 | | | | 60,00 | | |  | | | 290,00 | | | | 16,00 | | | | 11,00 | | | | 12,00 | | | | | 12,00 | | | | 8,00 | | | | |  | | | |  | | |  | 92,00 |  | 0,96 |  |  |  | 72,00 | 23,00 |  |  |  |  |  |  |  |  |
| **139.4** | | 154,00 | 27,00 | | | | 172,00 | | |  | | | 504,00 | | | | 28,00 | | | | 32,00 | | | | 49,00 | | | | | 28,00 | | | | 7,00 | | | | |  | | | |  | | |  | 187,00 |  | 0,96 |  |  |  | 153,00 | 56,00 |  |  |  |  |  |  |  |  |
| **140** | |  |  | | | |  | | |  | | |  | | | |  | | | |  | | | |  | | | | |  | | | |  | | | | |  | | | |  | | |  |  |  |  |  |  |  |  |  |  |  |  |  |  |  |  |  |
| **141** | | 34,00 | 4,00 | | | |  | | | 8,00 | | | 9,00 | | | |  | | | | 8,00 | | | | 5,00 | | | | |  | | | | 10,00 | | | | |  | | | |  | | | 660,00 |  |  | 2,00 |  |  |  | 10,00 | 16,00 |  |  |  |  |  |  |  |  |
| **142.1** | | 47,00 | 5,00 | | | |  | | |  | | | 246,00 | | | | 7,00 | | | | 20,00 | | | | 2,00 | | | | | 11,00 | | | | 11,00 | | | | | 1,00 | | | |  | | |  | 31,00 |  | 2,00 |  |  |  | 32,00 |  | 19,00 |  |  |  |  |  |  |  |
| **142.2** | | 9,00 | 9,00 | | | |  | | |  | | | 49,00 | | | | 1,00 | | | | 4,00 | | | | 0,00 | | | | | 12,00 | | | | 17,00 | | | | | 7,00 | | | |  | | |  | 8,00 |  | 2,00 |  |  |  | 9,00 |  | 6,00 |  |  |  |  |  |  |  |
| **143.1** | | 10,00 | 3,00 | | | |  | | |  | | | 5,00 | | | | 0,00 | | | | 2,00 | | | |  | | | | |  | | | |  | | | | | 3,00 | | | |  | | |  |  |  |  |  |  |  | 6,00 | 7,00 |  |  | 4,00 |  |  |  |  |  |
| **143.2** | | 57,00 | 18,00 | | | |  | | |  | | | 82,00 | | | | 2,00 | | | | 16,00 | | | |  | | | | |  | | | |  | | | | | 18,00 | | | |  | | |  |  |  |  |  |  |  | 23,00 | 43,00 |  |  | 27,00 |  |  |  |  |  |
| **144.1** | | 9,00 | 3,00 | | | | 3,00 | | |  | | | 3,00 | | | | 0,00 | | | | 2,00 | | | | 4,00 | | | | | 4,00 | | | |  | | | | | 4,00 | | | |  | | | 660,00 |  |  | 1,00 |  |  |  |  | 1,00 |  |  | 2,00 |  |  |  |  |  |
| **144.2** | | 60,00 | 17,00 | | | | 26,00 | | |  | | | 87,00 | | | | 2,00 | | | | 16,00 | | | | 57,00 | | | | | 26,00 | | | |  | | | | | 18,00 | | | |  | | | 660,00 |  |  | 1,00 |  |  |  |  | 52,00 |  |  | 29,00 |  |  |  |  |  |
| **145** | |  |  | | | |  | | |  | | |  | | | |  | | | | 0,00 | | | |  | | | | |  | | | |  | | | | | 0,00 | | | |  | | |  |  |  | 2,20 |  |  |  |  |  |  |  |  |  |  |  |  |  |
| **146** | |  |  | | | |  | | |  | | |  | | | |  | | | |  | | | |  | | | | |  | | | |  | | | | |  | | | |  | | | 549,00 |  |  |  |  |  |  |  |  |  |  |  |  |  |  |  |  |
| **147** | | 3,00 |  | | | |  | | |  | | | 47,00 | | | |  | | | |  | | | | 0,00 | | | | |  | | | |  | | | | |  | | | |  | | |  |  |  |  |  |  |  |  |  |  |  |  |  |  |  |  |  |
| **148 .1** | | 0,00 | 0,00 | | | |  | | |  | | |  | | | | 1,00 | | | | 1,00 | | | |  | | | | |  | | | | 9,00 | | | | |  | | | | 7,00 | | |  |  |  | 1,00 |  |  |  |  |  |  |  |  |  |  |  |  |  |
| **148.2** | | 4,00 | 1,00 | | | |  | | |  | | |  | | | | 0,00 | | | | 0,00 | | | |  | | | | |  | | | | 8,00 | | | | |  | | | | 6,00 | | |  |  |  | 1,13 |  |  |  |  |  |  |  |  |  |  |  |  |  |
| **149.1** | |  |  | | | | 1,00 | | |  | | | 22,00 | | | | 1,00 | | | |  | | | |  | | | | |  | | | | 10,00 | | | | |  | | | |  | | | 180,00 | 9,00 |  | 1,71 |  |  |  |  |  |  |  |  |  |  |  |  |  |
| **149.2** | |  |  | | | | 3,00 | | |  | | | 12,00 | | | | 7,00 | | | |  | | | |  | | | | |  | | | | 11,00 | | | | |  | | | |  | | | 360,00 | 21,00 |  | 1,88 |  |  |  |  |  | 2,00 |  |  |  |  |  |  |  |
| **149.3** | |  |  | | | |  | | |  | | |  | | | |  | | | |  | | | |  | | | | |  | | | |  | | | | |  | | | |  | | |  |  |  |  |  |  |  |  |  |  |  |  |  |  |  |  |  |
| **149.4** | |  |  | | | |  | | |  | | |  | | | |  | | | |  | | | |  | | | | |  | | | |  | | | | |  | | | |  | | |  |  |  |  |  |  |  |  |  |  |  |  |  |  |  |  |  |
| **150** | | 1,00 |  | | | |  | | |  | | |  | | | |  | | | | 2,00 | | | |  | | | | |  | | | | 19,00 | | | | | 3,00 | | | |  | | |  |  |  |  |  |  |  | 12,00 | 3,00 |  |  |  |  |  |  |  |  |
| **151** | |  |  | | | |  | | |  | | | 10,00 | | | |  | | | |  | | | |  | | | | |  | | | | 6,54 | | | | | 0,00 | | | |  | | | 450,00 |  |  | 1,87 |  |  |  |  |  |  |  |  |  |  |  |  |  |
| **152 .1** | | 1,00 |  | | | |  | | | 0,00 | | | 17,00 | | | |  | | | | 0,00 | | | | 0,00 | | | | | 0,00 | | | | 4,00 | | | | | 0,00 | | | |  | | | 102,00 |  | 49,40 |  |  |  |  | 0,00 |  |  |  |  |  |  |  |  |  |
| **152.2** | | 0,00 |  | | | |  | | | 1,00 | | | 9,00 | | | |  | | | | 0,00 | | | | 0,00 | | | | | 0,00 | | | | 4,00 | | | | | 0,00 | | | |  | | | 0,00 |  | 46,30 |  |  |  |  | 0,00 |  |  |  |  |  |  |  |  |  |
| **152.3** | | 0,00 |  | | | |  | | | 0,00 | | | 4,00 | | | |  | | | | 0,00 | | | | 0,00 | | | | | 0,00 | | | | 4,00 | | | | | 0,00 | | | |  | | | 162,00 |  | 74,00 |  |  |  |  | 0,00 |  |  |  |  |  |  |  |  |  |
| **153.1** | | 1,00 | 1,00 | | | |  | | |  | | | 72,00 | | | |  | | | | 0,00 | | | |  | | | | |  | | | | 5,00 | | | | |  | | | |  | | |  |  |  |  |  |  |  | 3,00 | 12,00 |  |  |  |  |  |  |  |  |
| **153.2** | | 4,00 | 0,00 | | | |  | | |  | | | 27,00 | | | |  | | | | 0,00 | | | |  | | | | |  | | | | 5,00 | | | | |  | | | |  | | |  |  |  |  |  |  |  | 2,00 | 0,00 |  |  |  |  |  |  |  |  |
| **155.1** | |  |  | | | |  | | |  | | |  | | | |  | | | | 2,00 | | | | 0,00 | | | | |  | | | |  | | | | |  | | | |  | | |  | 1,00 |  |  |  |  |  |  |  |  |  |  |  |  |  |  |  |
| **155.2** | |  |  | | | |  | | |  | | |  | | | |  | | | | 3,00 | | | | 0,00 | | | | |  | | | |  | | | | |  | | | |  | | |  | 3,00 |  |  |  |  |  |  |  |  |  |  |  |  |  |  |  |
| **156.1** | | 3,00 |  | | | | 5,00 | | | 0,00 | | | 26,00 | | | | 1,00 | | | |  | | | | 1,00 | | | | |  | | | | 9,00 | | | | |  | | | |  | | | 360,00 |  | 22,00 |  |  |  |  | 0,00 | 11,00 |  |  |  |  |  |  |  |  |
| **156.2** | | 1,00 |  | | | | 5,00 | | | 3,00 | | | 23,00 | | | | 1,00 | | | |  | | | | 3,00 | | | | |  | | | | 8,00 | | | | |  | | | |  | | | 360,00 |  | 20,50 |  |  |  |  | 1,00 | 8,00 |  |  |  |  |  |  |  |  |
| **157.1** | | 0,00 |  | | | | 2,00 | | |  | | |  | | | |  | | | |  | | | | 0,00 | | | | |  | | | |  | | | | | 1,00 | | | |  | | | 876,00 |  |  |  |  |  |  |  |  |  |  |  |  |  |  |  |  |
| **157.2** | | 1,00 |  | | | | 0,00 | | |  | | |  | | | |  | | | |  | | | | 1,00 | | | | |  | | | |  | | | | | 1,00 | | | |  | | | 630,00 |  |  |  |  |  |  |  |  |  |  |  |  |  |  |  |  |
| **158.1** | |  | 51,00 | | | |  | | | 49,00 | | | 1638,00 | | | |  | | | | 81,00 | | | |  | | | | |  | | | | 4,90 | | | | |  | | | |  | | |  | 205,00 |  |  |  |  |  |  |  |  |  |  |  |  |  |  |  |
| **158.2** | |  | 179,00 | | | |  | | | 191,00 | | | 3998,00 | | | |  | | | | 199,00 | | | |  | | | | |  | | | | 5,90 | | | | |  | | | |  | | |  | 654,00 |  |  |  |  |  |  |  |  |  |  |  |  |  |  |  |
| **160** | | 1,00 |  | | | |  | | |  | | |  | | | |  | | | |  | | | |  | | | | |  | | | |  | | | | |  | | | |  | | |  |  |  |  |  |  |  |  |  |  |  |  |  |  |  |  |  |
| **161** | | 5,00 | 0,00 | | | |  | | |  | | | 11,00 | | | | 1,00 | | | | 1,00 | | | | 0,00 | | | | |  | | | | 9,00 | | | | | 0,00 | | | |  | | |  | 1,00 |  |  |  |  |  | 1,00 |  |  |  | 0,00 |  |  |  |  |  |
| **162.1** | |  |  | | | |  | | |  | | |  | | | |  | | | |  | | | |  | | | | |  | | | |  | | | | |  | | | |  | | |  |  |  |  |  |  |  |  |  |  |  |  |  |  |  |  |  |
| **162 .2** | |  |  | | | |  | | |  | | |  | | | |  | | | |  | | | |  | | | | |  | | | |  | | | | |  | | | |  | | |  |  |  |  |  |  |  |  |  |  |  |  |  |  |  |  |  |
| **163.1** | | 2,00 |  | | | |  | | |  | | | 50,00 | | | | 0,00 | | | | 1,00 | | | |  | | | | |  | | | | 10,60 | | | | |  | | | |  | | |  |  |  | 0,39 |  | 0,00 |  | 155,00 | 0,00 |  |  | 1,00 |  |  |  |  |  |
| **163.2** | | 9,00 |  | | | |  | | |  | | | 47,00 | | | | 1,00 | | | | 3,00 | | | |  | | | | |  | | | | 11,80 | | | | |  | | | |  | | |  |  |  | 0,25 |  | 1,00 |  | 156,00 | 0,00 |  |  | 0,00 |  |  |  |  |  |
| **164** | |  |  | | | |  | | |  | | |  | | | |  | | | | 1,00 | | | |  | | | | |  | | | | 23,50 | | | | | 2,00 | | | |  | | |  |  |  |  |  |  |  |  |  |  |  | 3,00 |  |  |  |  |  |
| **169.1** | |  |  | | | |  | | |  | | |  | | | |  | | | |  | | | |  | | | | |  | | | |  | | | | |  | | | |  | | |  |  |  |  |  |  |  |  |  |  |  |  |  |  |  |  |  |
| **169.2** | |  |  | | | |  | | |  | | |  | | | |  | | | |  | | | |  | | | | |  | | | |  | | | | |  | | | |  | | |  |  |  |  |  |  |  |  |  |  |  |  |  |  |  |  |  |
| **170.1** | | 6,00 |  | | | | 13,00 | | |  | | | 51,00 | | | |  | | | | 1,00 | | | |  | | | | |  | | | | 7,00 | | | | | 0,00 | | | |  | | | 0,00 |  |  | 0,96 |  |  |  |  |  |  |  |  |  |  |  |  |  |
| **170.2** | | 9,00 |  | | | | 5,00 | | |  | | | 12,00 | | | |  | | | | 0,00 | | | |  | | | | |  | | | | 9,00 | | | | | 1,00 | | | |  | | | 600,00 |  |  | 1,67 |  |  |  |  |  |  |  |  |  |  |  |  |  |
| **170.3** | | 2,00 |  | | | |  | | |  | | | 27,00 | | | |  | | | | 0,00 | | | |  | | | | |  | | | | 7,00 | | | | | 0,00 | | | |  | | | 0,00 |  |  | 0,96 |  |  |  |  |  |  |  |  |  |  |  |  |  |
| **170.4** | | 4,00 |  | | | |  | | |  | | | 24,00 | | | |  | | | | 1,00 | | | |  | | | | |  | | | | 8,00 | | | | | 0,00 | | | |  | | | 240,00 |  |  | 1,00 |  |  |  |  |  |  |  |  |  |  |  |  |  |
| **171.1** | | 0,00 |  | | | | 1,00 | | |  | | | 17,00 | | | | 2,00 | | | |  | | | |  | | | | |  | | | | 5,00 | | | | | 0,00 | | | |  | | |  |  |  | 1,00 |  |  |  | 2,00 |  |  |  |  |  |  |  |  |  |
| **171.2** | | 5,00 |  | | | | 0,00 | | |  | | | 14,00 | | | | 1,00 | | | |  | | | |  | | | | |  | | | | 7,00 | | | | | 0,00 | | | |  | | |  |  |  | 1,50 |  |  |  | 2,00 |  |  |  |  |  |  |  |  |  |
| **172.1** | | 27,00 |  | | | |  | | |  | | |  | | | |  | | | | 3,00 | | | |  | | | | |  | | | |  | | | | | 2,00 | | | |  | | |  |  |  |  |  |  |  |  |  |  |  |  |  |  |  |  |  |
| **172.2** | | 22,00 |  | | | |  | | |  | | |  | | | |  | | | | 5,00 | | | |  | | | | |  | | | |  | | | | | 4,00 | | | |  | | |  |  |  |  |  |  |  |  |  |  |  |  |  |  |  |  |  |
| **173** | | 1,00 | 0,00 | | | |  | | |  | | | 13,00 | | | |  | | | | 0,00 | | | | 0,00 | | | | |  | | | | 8,00 | | | | | 5,00 | | | |  | | |  |  |  |  |  |  |  | 2,00 |  |  |  |  |  |  |  |  |  |
| **174** | | 14,00 | 8,00 | | | |  | | |  | | | 56,00 | | | |  | | | | 15,00 | | | |  | | | | |  | | | | 7,00 | | | | |  | | | |  | | |  | 13,00 |  |  |  |  |  | 25,00 | 27,00 |  |  |  |  |  |  |  |  |
| **177** | | 420,00 | 122,00 | | | | 402,00 | | |  | | | 1064,00 | | | | 59,00 | | | |  | | | | 104,00 | | | | | 115,00 | | | | 8,00 | | | | |  | | | | 8,00 | | | 68,00 |  |  | 1,00 |  |  |  |  | 453,00 |  |  |  |  |  |  |  |  |
| **177.2** | | 95,00 | 29,00 | | | |  | | |  | | | 270,00 | | | | 12,00 | | | | 26,00 | | | | 50,00 | | | | | 35,00 | | | | 9,00 | | | | |  | | | | 8,00 | | |  |  |  | 0,96 |  |  |  |  | 53,00 |  |  |  |  |  |  |  |  |
| **177.3** | | 260,00 | 53,00 | | | |  | | |  | | | 567,00 | | | | 33,00 | | | | 53,00 | | | | 41,00 | | | | | 42,00 | | | | 8,00 | | | | |  | | | | 7,00 | | |  |  |  | 1,00 |  |  |  |  | 72,00 |  |  |  |  |  |  |  |  |
| **178** | |  |  | | | |  | | |  | | |  | | | |  | | | |  | | | |  | | | | |  | | | |  | | | | |  | | | |  | | |  |  |  | 0,63 |  |  |  |  |  |  |  |  |  |  |  |  |  |
| **179** | |  |  | | | |  | | |  | | |  | | | |  | | | |  | | | |  | | | | |  | | | | 8,25 | | | | |  | | | | 4,63 | | |  |  |  | 3,84 |  |  |  |  |  |  |  |  |  |  |  |  |  |
| **180** | |  |  | | | |  | | |  | | |  | | | |  | | | |  | | | |  | | | | |  | | | |  | | | | |  | | | |  | | |  |  |  |  |  |  |  |  |  |  |  |  |  |  |  |  |  |
| **181** | | 5,00 |  | | | |  | | |  | | |  | | | |  | | | | 1,00 | | | |  | | | | |  | | | | 7,00 | | | | | 4,00 | | | | 23,90 | | |  | 7,00 |  | 1,00 |  | 4,00 |  | 5,00 | 3,00 |  |  |  |  |  |  |  |  |
| **182** | |  |  | | | |  | | |  | | |  | | | |  | | | |  | | | |  | | | | |  | | | | 12,30 | | | | |  | | | |  | | |  |  |  |  |  |  |  |  |  |  |  |  |  |  |  |  |  |
| **183.1** | |  |  | | | |  | | |  | | |  | | | |  | | | |  | | | |  | | | | |  | | | |  | | | | |  | | | |  | | |  |  |  |  |  |  |  |  |  |  |  |  |  | 10,84 |  |  |  |
| **183.2** | |  |  | | | |  | | |  | | |  | | | |  | | | |  | | | |  | | | | |  | | | |  | | | | |  | | | |  | | |  |  |  |  |  |  |  |  |  |  |  |  |  | 10,18 |  |  |  |
| **184.1** | | 0,00 | 2,00 | | | |  | | |  | | |  | | | |  | | | | 1,00 | | | | 0,00 | | | | |  | | | | 9,70 | | | | | 3,00 | | | |  | | |  |  |  | 1,60 |  |  |  |  | 1,00 |  |  | 1,00 |  |  |  |  |  |
| **184.2** | | 1,00 | 0,00 | | | |  | | |  | | |  | | | |  | | | | 1,00 | | | | 0,00 | | | | |  | | | | 9,70 | | | | | 2,00 | | | |  | | |  |  |  | 1,40 |  |  |  |  | 2,00 |  |  | 0,00 |  |  |  |  |  |
| **184.3** | | 0,00 | 0,00 | | | |  | | |  | | |  | | | |  | | | | 1,00 | | | | 0,00 | | | | |  | | | | 9,80 | | | | | 0,00 | | | |  | | |  |  |  | 1,70 |  |  |  |  | 0,00 |  |  | 0,00 |  |  |  |  |  |
| **185** | |  |  | | | |  | | |  | | |  | | | |  | | | |  | | | |  | | | | |  | | | | 30,20 | | | | |  | | | |  | | |  |  |  | 3,50 |  |  |  |  |  |  |  |  |  |  |  |  |  |
| **186.1** | | 1,00 | 0,00 | | | |  | | |  | | | 18,00 | | | |  | | | | 4,00 | | | |  | | | | |  | | | | 10,30 | | | | | 7,00 | | | |  | | |  |  |  | 1,80 |  |  |  |  | 12,00 |  |  | 1,00 |  |  |  |  |  |
| **186.2** | | 0,00 | 3,00 | | | |  | | |  | | | 29,00 | | | |  | | | | 1,00 | | | |  | | | | |  | | | | 11,20 | | | | | 2,00 | | | |  | | |  |  |  | 2,20 |  |  |  |  | 9,00 |  |  | 2,00 |  |  |  |  |  |
| **187 .1** | |  | 5,00 | | | | 15,00 | | |  | | |  | | | |  | | | |  | | | | 12,00 | | | | | 16,00 | | | |  | | | | | 23,00 | | | |  | | |  |  |  |  |  |  |  |  |  |  |  |  |  |  |  |  |  |
| **187.2** | |  | 0,00 | | | | 13,00 | | |  | | |  | | | |  | | | |  | | | | 11,00 | | | | | 20,00 | | | |  | | | | | 31,00 | | | |  | | |  |  |  |  |  |  |  |  |  |  |  |  |  |  |  |  |  |
| **188** | | 6,00 | 2,00 | | | |  | | | 7,00 | | | 30,00 | | | |  | | | | 1,00 | | | |  | | | | |  | | | | 10,00 | | | | |  | | | |  | | | 540,00 |  |  | 1,70 |  |  |  | 3,00 | 3,00 |  |  |  |  | 10,90 |  |  |  |
| **189.1** | | 4,00 |  | | | |  | | | 2,00 | | | 25,00 | | | |  | | | |  | | | |  | | | | | 1,00 | | | | 5,30 | | | | | 1,00 | | | |  | | |  |  |  | 2,50 |  |  |  |  |  |  |  |  |  |  |  |  |  |
| **189 .2** | | 3,00 |  | | | |  | | | 2,00 | | | 18,00 | | | |  | | | |  | | | |  | | | | | 1,00 | | | | 5,50 | | | | | 2,00 | | | |  | | |  |  |  | 1,90 |  |  |  |  |  |  |  |  |  |  |  |  |  |
| **190.1** | | 0,00 |  | | | |  | | |  | | | 8,00 | | | |  | | | |  | | | | 0,00 | | | | |  | | | | 4,00 | | | | |  | | | |  | | |  |  |  | 0,90 |  |  |  |  |  |  |  |  |  |  |  |  |  |
| **190 .2** | | 7,00 |  | | | |  | | |  | | | 16,00 | | | |  | | | |  | | | | 0,00 | | | | |  | | | | 4,00 | | | | |  | | | |  | | |  |  |  | 0,90 |  |  |  |  |  |  |  |  |  |  |  |  |  |
| **191.1** | |  |  | | | |  | | |  | | |  | | | |  | | | |  | | | |  | | | | |  | | | |  | | | | |  | | | |  | | |  |  |  |  |  |  |  |  |  |  |  |  |  |  |  |  |  |
| **191.2** | |  |  | | | |  | | |  | | |  | | | |  | | | |  | | | |  | | | | |  | | | |  | | | | |  | | | |  | | |  |  |  |  |  |  |  |  |  |  |  |  |  |  |  |  |  |
| **191.3** | |  |  | | | |  | | |  | | |  | | | |  | | | |  | | | |  | | | | |  | | | |  | | | | |  | | | |  | | |  |  |  |  |  |  |  |  |  |  |  |  |  |  |  |  |  |
| **192.1** | |  |  | | | |  | | |  | | |  | | | |  | | | |  | | | |  | | | | |  | | | |  | | | | |  | | | |  | | |  |  |  |  |  |  |  |  |  |  |  |  |  |  |  |  |  |
| **192.2** | |  |  | | | |  | | |  | | |  | | | |  | | | |  | | | |  | | | | |  | | | |  | | | | |  | | | |  | | |  |  |  |  |  |  |  |  |  |  |  |  |  |  |  |  |  |
| **193.1** | | 3,00 |  | | | | 1,00 | | |  | | | 41,00 | | | |  | | | |  | | | |  | | | | |  | | | | 6,00 | | | | |  | | | | 0,00 | | |  |  |  | 1,25 |  | 1,00 |  | 2,00 |  |  |  |  |  |  |  |  |  |
| **193.2** | | 2,00 |  | | | | 6,00 | | |  | | | 49,00 | | | |  | | | | 2,00 | | | |  | | | | |  | | | | 7,00 | | | | | 1,00 | | | | 6,00 | | |  |  |  | 1,67 |  | 4,00 | 5,00 | 5,00 |  |  |  |  |  |  |  |  |  |
| **194.1** | |  |  | | | |  | | |  | | |  | | | |  | | | | 0,00 | | | |  | | | | |  | | | | 10,00 | | | | |  | | | |  | | |  |  |  | 2,90 |  |  |  |  |  |  |  |  |  |  |  |  |  |
| **194.2** | |  |  | | | |  | | |  | | |  | | | |  | | | | 0,00 | | | |  | | | | |  | | | | 7,10 | | | | |  | | | |  | | |  |  |  | 2,10 |  |  |  |  |  |  |  |  |  |  |  |  |  |
| **195.1** | | 5,00 |  | | | |  | | | 1,00 | | | 35,00 | | | |  | | | | 0,00 | | | |  | | | | |  | | | | 8,00 | | | | | 0,00 | | | |  | | |  | 2,00 |  |  |  |  | 1,00 |  |  |  |  |  |  |  |  |  |  |
| **195.2** | | 6,00 |  | | | |  | | | 3,00 | | | 13,00 | | | |  | | | | 1,00 | | | |  | | | | |  | | | | 7,00 | | | | | 0,00 | | | |  | | |  | 1,00 |  |  |  |  | 1,00 |  |  |  |  |  |  |  |  |  |  |
| **196** | | 5,00 | 3,00 | | | |  | | |  | | | 12,00 | | | |  | | | | 1,00 | | | | 0,00 | | | | | 3,00 | | | | 20,00 | | | | | 2,00 | | | | 6,00 | | |  | 10,00 |  | 0,88 |  |  |  |  | 4,00 |  |  |  |  |  |  |  |  |
| **197.1** | | 1,00 | 1,00 | | | |  | | |  | | |  | | | |  | | | | 1,00 | | | |  | | | | | 2,00 | | | | 7,00 | | | | |  | | | |  | | | 720,00 | 6,00 |  | 1,00 |  |  |  |  |  |  |  |  |  |  |  |  |  |
| **197.2** | | 1,00 | 0,00 | | | |  | | |  | | |  | | | |  | | | | 0,00 | | | |  | | | | | 1,00 | | | | 7,00 | | | | |  | | | |  | | | 660,00 | 0,00 |  | 1,00 |  |  |  |  |  |  |  |  |  |  |  |  |  |
| **198.1** | |  | 0,00 | | | |  | | |  | | |  | | | |  | | | | 0,00 | | | |  | | | | |  | | | | 8,00 | | | | | 0,00 | | | |  | | | 516,00 |  |  | 0,00 |  |  |  |  |  |  |  |  |  |  |  |  |  |
| **198.2** | |  | 0,00 | | | |  | | |  | | |  | | | |  | | | | 2,00 | | | |  | | | | |  | | | | 7,20 | | | | | 0,00 | | | |  | | | 522,00 |  |  | 2,00 |  |  |  |  |  |  |  |  |  |  |  |  |  |
| **199.1** | |  |  | | | |  | | |  | | |  | | | |  | | | | 2,00 | | | |  | | | | |  | | | |  | | | | | 2,00 | | | |  | | |  |  |  |  |  |  |  |  | 1,00 |  |  |  |  |  |  |  |  |
| **199.2** | |  |  | | | |  | | |  | | |  | | | |  | | | |  | | | |  | | | | |  | | | | 17,80 | | | | |  | | | |  | | |  |  |  | 4,20 |  |  |  |  | 1,00 |  |  |  |  |  |  |  |  |
| **199.3** | |  |  | | | |  | | |  | | |  | | | |  | | | |  | | | |  | | | | |  | | | | 24,40 | | | | |  | | | |  | | |  |  |  | 5,30 |  |  |  |  |  |  |  |  |  |  |  |  |  |
| **199.4** | |  |  | | | |  | | |  | | |  | | | |  | | | |  | | | |  | | | | |  | | | | 16,30 | | | | |  | | | |  | | |  |  |  | 2,90 |  |  |  |  |  |  |  |  |  |  |  |  |  |
| **199.5** | |  |  | | | |  | | |  | | |  | | | |  | | | |  | | | |  | | | | |  | | | | 11,40 | | | | |  | | | |  | | |  |  |  | 2,60 |  |  |  |  |  |  |  |  |  |  |  |  |  |
| **200.1** | |  |  | | | |  | | | 1,00 | | |  | | | |  | | | |  | | | |  | | | | |  | | | | 20,00 | | | | | 0,00 | | | |  | | | 0,00 |  |  | 1,45 |  |  |  | 0,00 |  |  |  |  |  | 9,53 |  |  |  |
| **200.2** | |  |  | | | |  | | | 3,00 | | |  | | | |  | | | |  | | | |  | | | | |  | | | | 15,00 | | | | | 0,00 | | | |  | | | 180,00 |  |  | 2,88 |  |  |  | 6,00 |  |  |  |  |  | 9,60 |  |  |  |
| **201** | |  |  | | | |  | | | 8,00 | | | 63,00 | | | |  | | | |  | | | | 0,00 | | | | |  | | | | 10,00 | | | | |  | | | |  | | | 600,00 |  |  | 1,08 |  |  |  |  |  |  |  |  |  |  |  |  |  |
| **202** | | 2,00 |  | | | |  | | |  | | |  | | | |  | | | |  | | | | 3,00 | | | | |  | | | |  | | | | | 1,00 | | | |  | | |  |  |  | 2,03 |  |  |  |  |  |  |  |  |  |  |  |  |  |
| **203** | |  |  | | | |  | | |  | | |  | | | |  | | | |  | | | |  | | | | |  | | | |  | | | | |  | | | |  | | |  |  |  |  |  |  |  |  |  |  |  |  |  |  |  |  |  |
| **204.1** | |  | 0,00 | | | |  | | |  | | |  | | | | 1,00 | | | | 0,00 | | | |  | | | | | 2,00 | | | | 8,10 | | | | |  | | | |  | | |  |  |  | 1,80 |  |  |  | 1,00 | 1,00 |  |  | 1,00 |  |  |  |  |  |
| **204.2** | |  | 0,00 | | | |  | | |  | | |  | | | | 0,00 | | | | 0,00 | | | |  | | | | | 1,00 | | | | 7,50 | | | | |  | | | |  | | |  |  |  | 1,60 |  |  |  | 2,00 | 1,00 |  |  | 0,00 |  |  |  |  |  |
| **205 .1** | |  |  | | | |  | | |  | | |  | | | |  | | | |  | | | |  | | | | |  | | | |  | | | | |  | | | |  | | |  |  |  |  |  |  |  |  |  |  |  |  |  |  |  |  |  |
| **205.2** | |  |  | | | |  | | |  | | |  | | | |  | | | |  | | | |  | | | | |  | | | |  | | | | |  | | | |  | | |  |  |  |  |  |  |  |  |  |  |  |  |  |  |  |  |  |
| **206** | |  | 3,00 | | | |  | | | 1,00 | | | 8,00 | | | | 1,00 | | | |  | | | |  | | | | |  | | | | 11,60 | | | | |  | | | | 31,39 | | |  |  |  | 3,32 |  | 4,00 |  |  |  |  |  |  | 0,70 |  |  |  |  |
| **207** | | 266,19 |  | | | |  | | |  | | | 2559,50 | | | | 112,62 | | | | 112,62 | | | |  | | | | |  | | | | 6,90 | | | | | 163,81 | | | |  | | |  | 593,80 |  | 2,46 |  |  | 982,85 | 112,62 | 337,85 |  |  |  |  |  |  |  |  |
| **208** | | 64,00 |  | | | | 24,00 | | |  | | | 405,00 | | | |  | | | | 6,00 | | | | 29,00 | | | | |  | | | | 8,00 | | | | | 32,00 | | | |  | | |  |  |  | 1,80 |  | 40,00 |  | 128,00 | 34,00 |  |  |  |  |  |  |  |  |
| **209.1** | | 0,00 | 3,00 | | | |  | | |  | | | 7,00 | | | | 0,00 | | | | 3,00 | | | |  | | | | |  | | | |  | | | | | 3,00 | | | |  | | |  |  |  |  |  |  |  | 2,00 | 0,00 |  |  |  |  |  |  |  |  |
| **209.2** | | 2,00 | 1,00 | | | |  | | |  | | | 27,00 | | | | 0,00 | | | | 3,00 | | | |  | | | | |  | | | |  | | | | | 1,00 | | | |  | | |  |  |  |  |  |  |  | 1,00 | 2,00 |  |  |  |  |  |  |  |  |
| **209.3** | | 3,00 | 7,00 | | | |  | | |  | | | 30,00 | | | | 0,00 | | | | 8,00 | | | |  | | | | |  | | | |  | | | | | 7,00 | | | |  | | |  |  |  |  |  |  |  | 9,00 | 6,00 |  |  |  |  |  |  |  |  |
| **209.4** | | 10,00 | 5,00 | | | |  | | |  | | | 193,00 | | | | 1,00 | | | | 8,00 | | | |  | | | | |  | | | |  | | | | | 8,00 | | | |  | | |  |  |  |  |  |  |  | 15,00 | 11,00 |  |  |  |  |  |  |  |  |
| **209.5** | | 7,00 | 10,00 | | | |  | | |  | | | 48,00 | | | | 0,00 | | | | 5,00 | | | |  | | | | |  | | | |  | | | | | 8,00 | | | |  | | |  |  |  |  |  |  |  | 10,00 | 1,00 |  |  |  |  |  |  |  |  |
| **210** | | 0,00 |  | | | |  | | | 4,00 | | |  | | | |  | | | |  | | | | 1,00 | | | | |  | | | | 10,50 | | | | |  | | | |  | | | 1284,00 |  |  | 2,20 |  |  |  | 2,00 |  |  |  |  | 2,50 |  |  |  |  |
| **211.1** | |  | 0,00 | | | |  | | |  | | |  | | | |  | | | |  | | | |  | | | | |  | | | | 7,00 | | | | |  | | | |  | | |  |  |  | 2,50 |  |  |  |  |  | 2,00 |  |  |  | 10,40 |  |  |  |
| **211.2** | |  | 1,00 | | | |  | | |  | | |  | | | |  | | | |  | | | |  | | | | |  | | | | 8,00 | | | | |  | | | |  | | |  |  |  | 2,00 |  |  |  |  |  | 2,00 |  |  |  | 9,74 |  |  |  |
| **212** | |  | 1,00 | | | |  | | | 3,00 | | |  | | | |  | | | |  | | | |  | | | | |  | | | |  | | | | |  | | | |  | | | 660,00 | 2,00 |  | 0,96 |  |  |  |  |  |  |  |  |  |  |  |  |  |
| **213** | | 1,00 | 1,00 | | | |  | | |  | | |  | | | |  | | | | 3,00 | | | |  | | | | |  | | | | 8,00 | | | | |  | | | |  | | | 372,00 | 3,00 |  | 1,01 |  |  |  |  |  |  |  |  |  |  |  |  |  |
| **214** | | 6,00 | 12,00 | | | |  | | | 8,00 | | | 9,00 | | | |  | | | | 1,00 | | | |  | | | | |  | | | |  | | | | |  | | | |  | | |  |  |  |  |  |  |  | 6,00 | 5,00 |  |  |  |  |  |  |  |  |
| **215** | |  |  | | | |  | | |  | | |  | | | |  | | | | 4,00 | | | |  | | | | | 4,00 | | | | 10,00 | | | | |  | | | |  | | |  |  |  |  |  |  |  | 47,00 |  |  |  |  | 0,90 |  |  |  |  |
| **216** | |  | 1,00 | | | |  | | |  | | |  | | | |  | | | |  | | | |  | | | | |  | | | | 8,00 | | | | |  | | | |  | | |  |  |  | 2,00 |  | 2,00 |  |  |  | 2,00 |  |  |  | 9,74 |  |  |  |
| **217** | |  |  | | | |  | | |  | | |  | | | |  | | | |  | | | |  | | | | |  | | | |  | | | | |  | | | |  | | |  |  |  |  |  |  |  |  |  |  |  |  |  |  |  |  |  |
| **218** | |  |  | | | |  | | |  | | |  | | | |  | | | |  | | | |  | | | | |  | | | |  | | | | |  | | | |  | | |  |  |  |  |  |  |  |  |  |  |  |  |  |  |  |  |  |
| **219.1** | | 2,00 |  | | | | 1,00 | | |  | | | 4,00 | | | |  | | | |  | | | |  | | | | |  | | | | 10,20 | | | | | 0,00 | | | |  | | | 1878,00 |  |  | 2,60 |  |  | 1,00 |  |  |  |  | 1,00 |  |  |  |  |  |
| **219 .2** | | 1,00 |  | | | | 1,00 | | |  | | | 2,00 | | | |  | | | |  | | | |  | | | | |  | | | | 10,10 | | | | | 0,00 | | | |  | | | 1902,00 |  |  | 2,70 |  |  | 0,00 |  |  |  |  | 1,00 |  |  |  |  |  |
| **220.1** | | 21,00 | 7,00 | | | |  | | |  | | | 28,00 | | | | 0,00 | | | | 6,00 | | | | 12,00 | | | | | 9,00 | | | | 11,00 | | | | | 7,00 | | | |  | | | 780,00 |  |  | 1,50 |  |  |  |  | 18,00 |  |  | 6,00 |  |  |  |  |  |
| **220.2** | | 13,00 | 4,00 | | | |  | | |  | | | 17,00 | | | | 0,00 | | | | 4,00 | | | | 10,00 | | | | | 7,00 | | | | 11,00 | | | | | 3,00 | | | |  | | | 780,00 |  |  | 1,00 |  |  |  |  | 13,00 |  |  | 4,00 |  |  |  |  |  |
| **220.3** | | 8,00 | 3,00 | | | |  | | |  | | | 6,00 | | | | 0,00 | | | | 2,00 | | | | 2,00 | | | | | 2,00 | | | | 12,00 | | | | | 3,00 | | | |  | | | 780,00 |  |  | 2,00 |  |  |  |  | 4,00 |  |  | 2,00 |  |  |  |  |  |
| **220.4** | | 0,00 | 0,00 | | | |  | | |  | | | 5,00 | | | | 0,00 | | | | 0,00 | | | | 0,00 | | | | | 0,00 | | | | 10,00 | | | | | 1,00 | | | |  | | | 660,00 |  |  | 1,00 |  |  |  |  | 1,00 |  |  | 0,00 |  |  |  |  |  |
| **220.5** | | 15,00 | 5,00 | | | |  | | |  | | | 16,00 | | | | 1,00 | | | | 2,00 | | | | 14,00 | | | | | 7,00 | | | | 10,00 | | | | | 5,00 | | | |  | | | 660,00 |  |  | 1,00 |  |  |  |  | 11,00 |  |  | 8,00 |  |  |  |  |  |
| **221** | |  |  | | | |  | | |  | | |  | | | |  | | | |  | | | |  | | | | |  | | | | 7,00 | | | | |  | | | |  | | | 366,00 |  |  | 5,00 |  |  |  |  |  |  |  |  |  |  |  |  |  |
| **222.1** | |  |  | | | |  | | |  | | |  | | | |  | | | | 0,00 | | | |  | | | | | 2,00 | | | |  | | | | | 0,00 | | | |  | | |  |  |  | 1,00 |  |  |  |  |  |  |  |  |  |  |  |  |  |
| **222.2** | |  |  | | | |  | | |  | | |  | | | |  | | | | 4,00 | | | |  | | | | | 2,00 | | | |  | | | | | 0,00 | | | |  | | |  |  |  | 1,00 |  |  |  |  |  |  |  |  |  |  |  |  |  |
| **223** | | 2,00 |  | | | | 4,00 | | |  | | | 3,00 | | | |  | | | | 1,00 | | | |  | | | | |  | | | | 7,10 | | | | | 3,00 | | | |  | | |  |  |  | 1,30 |  |  |  | 1,00 | 3,00 |  |  |  |  |  |  |  |  |
| **224** | |  |  | | | |  | | |  | | |  | | | |  | | | |  | | | |  | | | | |  | | | | 5,22 | | | | |  | | | |  | | |  |  |  | 1,50 |  |  |  |  |  |  |  |  |  |  |  |  |  |
| **225.1** | |  | 2,00 | | | |  | | |  | | |  | | | | 4,00 | | | |  | | | |  | | | | |  | | | | 11,86 | | | | |  | | | |  | | | 848,40 |  |  | 2,93 |  |  |  |  |  | 2,00 |  |  |  |  |  |  |  |
| **225.2** | |  | 0,00 | | | |  | | |  | | |  | | | | 3,00 | | | |  | | | |  | | | | |  | | | | 10,86 | | | | |  | | | |  | | | 865,20 |  |  | 2,99 |  |  |  |  |  | 3,00 |  |  |  |  |  |  |  |
| **Ʃ** | | 2755,19 |  | | | | 1186,00 | | | 536,00 | | | 15726,50 | | | | 424,62 | | | | 945,62 | | | | 632,00 | | | | | 594,00 | | | | 1435,08 | | | | |  | | | |  | | |  |  | 1158,42 |  |  | 314,00 | 1058,85 | 1775,12 | 2023,85 | 79,00 | 0,00 | 105,00 |  |  |  |  |  |
| **mean** | |  | 7,71 | | | |  | | | 8,79 | | |  | | | |  | | | |  | | | |  | | | | |  | | | | 8,59 | | | | | 4,67 | | | | 9,22 | | | 540,81 | 49,79 | 35,10 | 1,80 |  |  | 58,82 |  |  |  |  |  | 1,12 | 10,79 | 29,53 | 35,70 | 24,60 |
| **SD** | |  | 24,19 | | | |  | | | 24,72 | | |  | | | |  | | | |  | | | |  | | | | |  | | | | 3,90 | | | | | 16,95 | | | | 6,30 | | | 439,79 | 134,98 | 15,88 | 1,06 |  |  | 224,15 |  |  |  |  |  | 0,53 | 0,88 | 4,05 | 0,00 | 0,00 |

|  | **CK-MB** | | **rMR0** | **rMR1** | **rMR2** | | **rMR3** | **rMS** | | | **AVR** | **MR** | **MMG** | | | **PMG** | | **LVEF (%)** | | **LA** | **MTV** | **MOA** | **LVDD** | | **LVDV** | | **LoC** | | | | |  |
| --- | --- | --- | --- | --- | --- | --- | --- | --- | --- | --- | --- | --- | --- | --- | --- | --- | --- | --- | --- | --- | --- | --- | --- | --- | --- | --- | --- | --- | --- | --- | --- | --- |
| **1** |  | | 467,00 | 21,00 | 3,00 | | 0,00 |  | | |  |  |  | | |  | |  | |  |  |  |  | |  | |  | | | | |  |
| **3** |  | | 0,00 | 0,00 | 0,00 | | 0,00 |  | | |  |  | 3,00 | | | 7,00 | | 55,00 | |  |  |  |  | |  | |  | | | | |  |
| **4** |  | |  |  |  | |  |  | | |  |  |  | | |  | |  | |  |  |  |  | |  | |  | | | | |  |
| **5** |  | | 94,00 | 33,00 | 18,00 | | 4,00 |  | | |  |  |  | | |  | |  | |  |  |  |  | |  | |  | | | | |  |
| **7** | 26,20 | |  |  |  | |  |  | | |  |  |  | | |  | | 58,80 | | 34,30 |  |  |  | |  | |  | | | | |  |
| **8** |  | |  |  |  | |  |  | | |  |  |  | | |  | |  | |  |  |  |  | |  | |  | | | | |  |
| **9** |  | |  |  |  | |  |  | | |  |  |  | | |  | |  | |  |  | 0,60 |  | |  | |  | | | | |  |
| **13** |  | |  |  |  | |  |  | | |  |  |  | | |  | |  | |  |  |  |  | |  | |  | | | | |  |
| **14.1** |  | | 28,00 |  |  | |  |  | | |  |  |  | | |  | |  | |  |  |  |  | |  | |  | | | | |  |
| **14.2** |  | | 62,00 |  |  | |  |  | | |  |  |  | | |  | |  | |  |  |  |  | |  | |  | | | | |  |
| **14.3** |  | | 82,00 |  |  | |  |  | | |  |  |  | | |  | |  | |  |  |  |  | |  | |  | | | | |  |
| **15.1** | 40,00 | |  | 3,00 |  | |  |  | | |  |  |  | | |  | | 50,30 | |  |  |  |  | |  | |  | | | | |  |
| **15.2** | 33,40 | |  | 0,00 |  | |  |  | | |  |  |  | | |  | | 49,10 | |  |  |  |  | |  | |  | | | | |  |
| **18.1** |  | |  |  |  | |  |  | | |  |  |  | | |  | |  | |  |  |  |  | |  | |  | | | | |  |
| **18.2** |  | |  |  |  | |  |  | | |  |  |  | | |  | |  | |  |  |  |  | |  | |  | | | | |  |
| **18.3** |  | |  |  |  | |  |  | | |  |  |  | | |  | |  | |  |  |  |  | |  | |  | | | | |  |
| **20** |  | |  |  |  | | 7,00 |  | | | 117,00 |  |  | | |  | | 60,00 | |  |  |  |  | |  | |  | | | | |  |
| **21** |  | | 73,00 | 2,00 | 0,00 | | 0,00 |  | | |  |  |  | | |  | |  | |  |  |  |  | |  | |  | | | | |  |
| **26** |  | | 86,00 | 10,00 | 1,00 | | 2,00 |  | | |  |  | 1,00 | | |  | |  | |  |  |  |  | |  | | 10,00 | | | | |  |
| **29** |  | |  |  |  | |  |  | | |  |  | 4,60 | | |  | | 56,80 | |  |  |  |  | |  | |  | | | | |  |
| **30** |  | |  |  |  | |  |  | | |  |  |  | | |  | |  | |  |  |  |  | |  | |  | | | | |  |
| **31.1** |  | | 58,00 | 11,00 | 2,00 | | 0,00 |  | | |  |  |  | | |  | | 59,90 | |  |  |  |  | |  | |  | | | | |  |
| **31.2** |  | | 25,00 | 4,00 | 1,00 | | 1,00 |  | | |  |  |  | | |  | | 59,80 | |  |  |  |  | |  | |  | | | | |  |
| **35** |  | |  |  |  | |  |  | | |  |  |  | | |  | |  | |  |  |  |  | |  | |  | | | | |  |
| **38** |  | | 33,00 | 7,00 | 0,00 | | 0,00 |  | | |  |  |  | | |  | | 51,40 | | 40,90 |  |  | 48,60 | |  | |  | | | | |  |
| **40** |  | |  |  |  | |  |  | | |  |  |  | | |  | |  | |  |  |  |  | |  | |  | | | | |  |
| **41** |  | | 107,00 | 2,00 | 0,00 | | 0,00 |  | | |  |  |  | | |  | | 55,00 | |  |  |  |  | |  | |  | | | | |  |
| **42** |  | |  |  |  | |  |  | | |  |  |  | | |  | |  | |  |  |  |  | |  | |  | | | | |  |
| **43** |  | |  |  |  | |  |  | | |  |  |  | | |  | |  | |  |  |  |  | |  | |  | | | | |  |
| **44.1** |  | |  |  |  | |  |  | | |  |  |  | | |  | |  | |  |  |  |  | |  | |  | | | | |  |
| **44.2** |  | |  |  |  | |  |  | | |  |  |  | | |  | |  | |  |  |  |  | |  | |  | | | | |  |
| **45** |  | |  |  |  | |  |  | | |  |  | 3,40 | | |  | | 60,00 | | 38,00 |  |  | 31,00 | |  | |  | | | | |  |
| **46** |  | | 150,00 |  |  | |  |  | | |  |  | 2,93 | | |  | |  | |  |  |  |  | |  | | 9,22 | | | | |  |
| **47** |  | |  |  |  | |  |  | | |  |  |  | | |  | |  | |  |  |  |  | |  | |  | | | | |  |
| **48** |  | |  |  |  | |  |  | | |  |  |  | | |  | |  | |  |  |  |  | |  | |  | | | | |  |
| **51** |  | |  | 2,00 | 0,00 | | 0,00 |  | | |  |  | 4,10 | | |  | |  | |  |  |  |  | |  | |  | | | | |  |
| **53.1** |  | | 38,00 | 228,00 | 10,00 | | 5,00 |  | | |  |  | 3,40 | | |  | | 56,80 | |  |  |  |  | |  | |  | | | | |  |
| **53.2** |  | | 18,00 | 91,00 | 4,00 | | 3,00 |  | | |  |  | 3,60 | | |  | | 58,30 | |  |  |  |  | |  | |  | | | | |  |
| **53.3** |  | | 15,00 | 62,00 | 2,00 | | 0,00 |  | | |  |  | 3,40 | | |  | | 57,60 | |  |  |  |  | |  | |  | | | | |  |
| **55.1** |  | |  |  |  | |  |  | | |  |  |  | | |  | | 58,18 | |  |  |  |  | |  | |  | | | | |  |
| **55.2** |  | |  |  |  | |  |  | | |  |  |  | | |  | | 57,00 | |  |  |  |  | |  | |  | | | | |  |
| **56.1** |  | |  |  |  | |  |  | | |  |  |  | | |  | |  | |  |  |  |  | |  | |  | | | | |  |
| **56.2** |  | |  |  |  | |  |  | | |  |  |  | | |  | |  | |  |  |  |  | |  | |  | | | | |  |
| **57** |  | |  |  |  | |  |  | | |  |  |  | | |  | |  | |  |  |  |  | |  | |  | | | | |  |
| **63.1** |  | | 42,00 | 41,00 | 2,00 | |  |  | | |  |  |  | | |  | |  | |  |  |  |  | |  | |  | | | | |  |
| **63.2** |  | | 43,00 | 37,00 | 5,00 | |  |  | | |  |  |  | | |  | |  | |  |  |  |  | |  | |  | | | | |  |
| **65** |  | |  |  |  | |  |  | | |  |  |  | | |  | |  | |  |  |  |  | |  | |  | | | | |  |
| **66** |  | |  | 810,00 | 64,00 | | 8,00 |  | | |  |  |  | | |  | |  | |  |  |  |  | |  | |  | | | | |  |
| **67** |  | |  |  |  | |  |  | | |  |  |  | | |  | |  | |  |  |  |  | |  | |  | | | | |  |
| **68** | 47,00 | |  |  |  | |  |  | | |  |  |  | | |  | |  | |  |  |  |  | |  | |  | | | | |  |
| **71** |  | |  |  |  | |  |  | | |  |  |  | | |  | |  | |  |  |  |  | |  | |  | | | | |  |
| **72** |  | |  |  |  | |  |  | | |  |  |  | | |  | |  | |  |  |  |  | |  | |  | | | | |  |
| **73** |  | |  |  |  | |  |  | | |  |  |  | | |  | |  | |  |  |  |  | |  | |  | | | | |  |
| **74** |  | | 73,00 | 131,00 | 9,00 | |  |  | | |  |  | 3,00 | | |  | | 62,00 | |  |  |  |  | |  | |  | | | | |  |
| **75** |  | |  |  |  | |  |  | | |  |  |  | | |  | |  | |  |  |  |  | |  | |  | | | | |  |
| **77** |  | |  |  |  | |  |  | | |  |  |  | | |  | |  | |  |  |  |  | |  | |  | | | | |  |
| **78.1** |  | |  |  |  | |  |  | | |  |  |  | | |  | |  | |  |  |  |  | |  | |  | | | | |  |
| **78.2** |  | |  |  |  | |  |  | | |  |  |  | | |  | |  | |  |  |  |  | |  | |  | | | | |  |
| **79** |  | |  |  |  | |  |  | | |  |  |  | | |  | |  | |  |  |  |  | |  | |  | | | | |  |
| **80** |  | |  |  | 7,00 | |  |  | | |  |  |  | | |  | |  | |  |  |  |  | |  | |  | | | | |  |
| **82** |  | |  |  |  | |  |  | | |  |  |  | | |  | |  | |  |  |  |  | |  | |  | | | | |  |
| **83** |  | |  |  |  | |  |  | | |  |  |  | | |  | |  | |  |  |  |  | |  | |  | | | | |  |
| **86** |  | |  |  |  | |  |  | | |  |  |  | | |  | |  | |  |  |  |  | |  | |  | | | | |  |
| **87.1** |  | |  |  |  | |  |  | | |  |  |  | | |  | |  | |  |  |  |  | |  | |  | | | | |  |
| **87.2** |  | |  |  |  | |  |  | | |  |  |  | | |  | |  | |  |  |  |  | |  | |  | | | | |  |
| **93** |  | |  | 15,00 | 2,00 | |  |  | | |  |  |  | | |  | | 61,50 | | 38,70 |  |  | 48,90 | |  | |  | | | | |  |
| **98** |  | | 110,00 | 82,00 | 4,00 | | 0,00 |  | | |  |  |  | | |  | | 58,30 | | 37,80 |  |  | 48,00 | |  | |  | | | | |  |
| **99.1** |  | |  |  |  | |  |  | | |  |  |  | | |  | |  | |  |  |  |  | |  | |  | | | | |  |
| **99.2** |  | |  |  |  | |  |  | | |  |  |  | | |  | |  | |  |  |  |  | |  | |  | | | | |  |
| **101.1** |  | |  |  |  | |  |  | | |  |  |  | | |  | |  | |  |  |  |  | |  | |  | | | | |  |
| **101.2** |  | |  |  |  | |  |  | | |  |  |  | | |  | |  | |  |  |  |  | |  | |  | | | | |  |
| **102** |  | |  | 24,00 | 3,00 | |  |  | | |  |  |  | | |  | |  | |  |  |  |  | |  | |  | | | | |  |
| **103** |  | |  |  |  | |  |  | | |  |  |  | | |  | |  | |  |  |  |  | |  | |  | | | | |  |
| **104** |  | |  |  |  | |  |  | | |  |  |  | | |  | |  | |  |  |  |  | |  | |  | | | | |  |
| **105** |  | |  |  |  | |  |  | | |  |  |  | | |  | |  | |  |  |  |  | |  | |  | | | | |  |
| **107** |  | |  |  | 0,00 | |  |  | | |  |  |  | | |  | |  | |  |  |  |  | |  | |  | | | | |  |
| **108** |  | |  |  |  | |  |  | | |  |  |  | | |  | |  | |  |  |  |  | |  | |  | | | | |  |
| **110** |  | | 5,00 |  |  | |  |  | | |  |  |  | | |  | | 55,00 | |  |  |  |  | |  | |  | | | | |  |
| **111** |  | |  |  | 17,00 | |  |  | | |  |  |  | | |  | | 52,00 | |  |  |  |  | |  | |  | | | | |  |
| **112** |  | |  |  |  | |  |  | | |  |  |  | | |  | |  | |  |  |  |  | |  | |  | | | | |  |
| **113.1** |  | |  |  |  | |  |  | | |  |  |  | | |  | |  | |  |  |  |  | |  | |  | | | | |  |
| **113.2** |  | |  |  |  | |  |  | | |  |  |  | | |  | |  | |  |  |  |  | |  | |  | | | | |  |
| **115.1** |  | |  |  |  | |  |  | | |  |  |  | | |  | |  | |  |  |  |  | |  | |  | | | | |  |
| **115.2** |  | |  |  |  | |  |  | | |  |  |  | | |  | |  | |  |  |  |  | |  | |  | | | | |  |
| **115.3** |  | |  |  |  | |  |  | | |  |  |  | | |  | |  | |  |  |  |  | |  | |  | | | | |  |
| **116.1** |  | |  |  |  | |  |  | | |  |  | |  |  | | |  | |  |  |  |  | |  | |  | | | | | |
| **116.2** |  | |  |  |  | |  |  | | |  |  |  | | |  | |  | |  |  |  |  | |  | |  | | | | | |
| **117.1** |  | |  |  |  | |  |  | | |  |  |  | | |  | | 57,53 | |  |  |  | 55,55 | |  | |  | | | | | |
| **117.2** |  | |  |  |  | |  |  | | |  |  |  | | |  | | 57,63 | |  |  |  | 55,94 | |  | |  | | | | | |
| **118** |  | |  |  |  | |  |  | | |  |  |  | | |  | |  | |  |  |  |  | |  | |  | | | | | |
| **119.1** |  | |  |  |  | |  |  | | |  |  |  | | |  | |  | |  |  |  |  | |  | |  | | | | | |
| **119.2** |  | |  |  |  | |  |  | | |  |  |  | | |  | |  | |  |  |  |  | |  | |  | | | | | |
| **121.1** | 1394,40 | |  |  |  | |  |  | | |  |  |  | | |  | |  | |  |  |  |  | |  | |  | | | | | |
| **121.2** | 1775,60 | |  |  |  | |  |  | | |  |  |  | | |  | |  | |  |  |  |  | |  | |  | | | | | |
| **122.1** | 36,00 | |  |  |  | |  |  | | |  |  |  | | |  | |  | |  |  |  |  | |  | |  | | | | | |
| **122.2** | 33,00 | |  |  |  | |  |  | | |  |  |  | | |  | |  | |  |  |  |  | |  | |  | | | | | |
| **123.1** |  | |  |  |  | |  |  | | |  |  |  | | |  | |  | |  |  |  |  | |  | |  | | | | | |
| **123.2** |  | |  |  |  | |  |  | | |  |  |  | | |  | |  | |  |  |  |  | |  | |  | | | | | |
| **127** |  | |  |  |  | |  |  | | |  |  | 2,30 | | | 6,33 | | 51,40 | | 44,70 | 1,22 |  |  | |  | |  | | | | | |
| **128** |  | |  |  |  | |  |  | | |  |  |  | | |  | |  | |  |  |  |  | |  | |  | | | | | |
| **129** |  | | 152,00 | 1,00 |  | |  |  | | |  |  |  | | |  | |  | |  |  |  |  | |  | |  | | | | | |
| **130** |  | |  |  | 0,00 | |  |  | | |  |  |  | | |  | | 60,00 | |  |  |  |  | |  | |  | | | | | |
| **131.1** |  | |  |  |  | |  |  | | |  |  |  | | |  | |  | |  |  |  |  | |  | |  | | | | | |
| **131.2** |  | |  |  |  | |  |  | | |  |  |  | | |  | |  | |  |  |  |  | |  | |  | | | | | |
| **132 .1** |  | |  |  | 0,00 | |  |  | | |  |  |  | | |  | |  | |  |  |  |  | |  | |  | | | | | |
| **132 .2** |  | |  |  | 11,00 | |  |  | | |  |  |  | | |  | |  | |  |  |  |  | |  | |  | | | | | |
| **133** |  | |  |  |  | |  |  | | |  |  |  | | |  | |  | |  |  |  |  | |  | |  | | | | | |
| **134.1** |  | |  |  |  | |  |  | | |  |  |  | | |  | |  | |  |  |  |  | |  | |  | | | | | |
| **134.2** |  | |  |  |  | |  |  | | |  |  |  | | |  | |  | |  |  |  |  | |  | |  | | | | | |
| **135 .1** |  | |  |  | 2,00 | | 1,00 |  | | |  |  |  | | |  | |  | |  |  |  |  | |  | |  | | | | | |
| **135.2** |  | |  |  | 3,00 | | 1,00 |  | | |  |  |  | | |  | |  | |  |  |  |  | |  | |  | | | | | |
| **136.1** |  | | 120,00 | 37,00 | 5,00 | | 1,00 |  | | |  |  | |  |  | | |  | |  |  |  |  | |  | |  | | | | | |
| **136.2** |  | | 153,00 | 32,00 | 7,00 | | 0,00 |  | | |  |  |  | | |  | |  | |  |  |  |  | |  | |  | | | | | |
| **136.3** |  | | 105,00 | 34,00 | 0,00 | | 0,00 |  | | |  |  | |  |  | | |  | |  |  |  |  | |  | |  | | | | | |
| **136.4** |  | | 20,00 | 6,00 | 0,00 | | 0,00 |  | | |  |  |  | | |  | |  | |  |  |  |  | |  | |  | | | | | |
| **136.5** |  | | 15,00 | 1,00 | 0,00 | | 0,00 |  | | |  |  | |  |  | | |  | |  |  |  |  | |  | |  | | | | | |
| **137** |  |  | |  | |  |  | |  |  | | | |  |  | |  | |  | |  |  | |  | |  | |  |  |  |  |  |
| **138** |  |  | |  | |  |  | |  |  | | | |  |  | |  | |  | |  |  | |  | |  | |  |  | |  |  |
| **139.1** |  |  | | 6,00 | | 1,00 | 1,00 | |  |  | | | |  |  | |  | |  | |  |  | |  | |  | |  |  | |  |  |
| **139.2** |  |  | | 13,00 | | 0,00 | 0,00 | |  |  | | | |  |  | |  | |  | |  |  | |  | |  | |  |  | |  |  |
| **139.3** |  |  | | 222,00 | | 19,00 | 1,00 | |  |  | | | |  |  | |  | |  | |  |  | |  | |  | |  |  | |  |  |
| **139.4** |  |  | | 387,00 | | 40,00 | 4,00 | |  |  | | | |  |  | |  | |  | |  |  | |  | |  | |  |  | |  |  |
| **140** |  |  | |  | |  |  | |  |  | | | |  |  | |  | |  | |  |  | |  | |  | |  |  | |  |  |
| **141** |  |  | |  | |  |  | |  |  | | | |  |  | |  | |  | |  |  | |  | |  | |  |  | |  |  |
| **142.1** |  |  | |  | |  |  | |  |  | | | |  |  | |  | |  | |  |  | |  | |  | |  |  | |  |  |
| **142.2** |  |  | |  | |  |  | |  |  | | | |  |  | |  | |  | |  |  | |  | |  | |  |  | |  |  |
| **143.1** |  | 7,00 | | 0,00 | | 0,00 | 0,00 | |  |  | | | |  |  | |  | | 55,00 | |  |  | |  | |  | |  |  | |  |  |
| **143.2** |  | 155,00 | | 19,00 | | 1,00 | 0,00 | |  |  | | | |  |  | |  | | 53,00 | |  |  | |  | |  | |  |  | |  |  |
| **144.1** |  | 7,00 | | 1,00 | | 0,00 | 0,00 | |  |  | | | |  |  | |  | | 55,00 | |  |  | |  | |  | |  |  | |  |  |
| **144.2** |  | 163,00 | | 20,00 | | 0,00 | 0,00 | |  |  | | | |  |  | |  | | 53,00 | |  |  | |  | |  | |  |  | |  |  |
| **145** |  |  | |  | |  |  | |  |  | | | |  |  | |  | | 52,17 | |  |  | |  | |  | |  |  | |  |  |
| **146** |  |  | |  | |  |  | |  |  | | | |  |  | |  | |  | |  |  | |  | |  | |  |  | |  |  |
| **147** |  |  | |  | |  |  | |  |  | | | |  |  | |  | |  | |  |  | |  | |  | |  |  | |  |  |
| **148 .1** |  |  | |  | |  |  | |  |  | | | |  |  | |  | | 55,00 | |  |  | |  | |  | |  |  | |  |  |
| **148.2** |  |  | |  | |  |  | |  |  | | | |  |  | |  | | 55,00 | |  |  | |  | |  | |  |  | |  |  |
| **149.1** |  |  | | 7,00 | |  | 22,00 | |  |  | | | |  | 3,50 | |  | |  | |  |  | |  | |  | |  |  | |  |  |
| **149.2** |  |  | | 10,00 | |  | 15,00 | |  |  | | | |  | 3,00 | |  | |  | |  |  | |  | |  | |  |  | |  |  |
| **149.3** |  |  | |  | |  |  | |  |  | | | |  |  | |  | |  | |  |  | |  | |  | |  |  | |  |  |
| **149.4** |  |  | |  | |  |  | |  |  | | | |  |  | |  | |  | |  |  | |  | |  | |  |  | |  |  |
| **150** |  |  | |  | |  |  | |  |  | | | |  |  | |  | | 53,60 | |  |  | |  | |  | |  |  | |  |  |
| **151** |  |  | |  | |  |  | |  |  | | | |  |  | |  | |  | |  |  | |  | |  | |  |  | |  |  |
| **152 .1** |  |  | |  | |  |  | |  |  | | | |  |  | |  | |  | |  |  | |  | |  | |  |  | |  |  |
| **152.2** |  |  | |  | |  |  | |  |  | | | |  |  | |  | |  | |  |  | |  | |  | |  |  | |  |  |
| **152.3** |  |  | |  | |  |  | |  |  | | | |  |  | |  | |  | |  |  | |  | |  | |  |  | |  |  |
| **153.1** |  |  | |  | | 11,00 | 1,00 | |  |  | | | |  |  | |  | |  | |  |  | |  | |  | |  |  | |  |  |
| **153.2** |  |  | |  | | 18,00 | 5,00 | |  |  | | | |  |  | |  | |  | |  |  | |  | |  | |  |  | |  |  |
| **155.1** |  |  | |  | |  |  | |  |  | | | |  |  | |  | |  | |  |  | |  | |  | |  |  | |  |  |
| **155.2** |  |  | |  | |  |  | |  |  | | | |  |  | |  | |  | |  |  | |  | |  | |  |  | |  |  |
| **156.1** |  |  | | 7,00 | |  |  | |  |  | | | |  |  | |  | |  | |  |  | |  | |  | |  |  | |  |  |
| **156.2** |  |  | | 8,00 | |  |  | |  |  | | | |  |  | |  | |  | |  |  | |  | |  | |  |  | |  |  |
| **157.1** |  |  | |  | |  |  | |  |  | | | |  |  | |  | | 61,90 | | 34,00 |  | |  | | 43,70 | |  |  | |  |  |
| **157.2** |  |  | |  | |  |  | |  |  | | | |  |  | |  | | 58,80 | | 34,30 |  | |  | | 43,50 | |  |  | |  |  |
| **158.1** |  |  | |  | | 43,00 | 44,00 | |  |  | | | |  |  | |  | |  | |  |  | |  | |  | |  |  | |  |  |
| **158.2** |  |  | |  | | 154,00 | 149,00 | |  |  | | | |  |  | |  | |  | |  |  | |  | |  | |  |  | |  |  |
| **160** |  | 28,00 | | 6,00 | | 0,00 |  | |  |  | | | |  |  | |  | |  | |  |  | |  | |  | |  |  | |  |  |
| **161** |  |  | |  | |  |  | |  |  | | | |  |  | |  | |  | |  |  | |  | |  | |  |  | |  |  |
| **162.1** |  |  | |  | |  |  | |  |  | | | |  |  | |  | |  | |  |  | |  | |  | |  |  | |  |  |
| **162 .2** |  |  | |  | |  |  | |  |  | | | |  |  | |  | |  | |  |  | |  | |  | |  |  | |  |  |
| **163.1** |  | 149,00 | | 7,00 | | 0,00 |  | |  |  | | | |  |  | |  | |  | |  |  | |  | |  | |  |  | |  |  |
| **163.2** |  | 140,00 | | 14,00 | | 2,00 |  | |  |  | | | |  |  | |  | |  | |  |  | |  | |  | |  |  | |  |  |
| **164** |  | 21,00 | | 20,00 | | 6,00 |  | |  |  | | | |  | 3,40 | |  | |  | |  |  | |  | |  | |  |  | |  |  |
| **169.1** |  |  | |  | |  |  | |  |  | | | |  |  | |  | |  | |  |  | |  | |  | |  |  | |  |  |
| **169.2** |  |  | |  | |  |  | |  |  | | | |  |  | |  | |  | |  |  | |  | |  | |  |  | |  |  |
| **170.1** |  |  | |  | |  |  | |  |  | | | |  |  | |  | |  | |  |  | |  | |  | |  |  | |  |  |
| **170.2** |  |  | |  | |  |  | |  |  | | | |  |  | |  | |  | |  |  | |  | |  | |  |  | |  |  |
| **170.3** |  |  | |  | |  |  | |  |  | | | |  |  | |  | |  | |  |  | |  | |  | |  |  | |  |  |
| **170.4** |  |  | |  | |  |  | |  |  | | | |  |  | |  | |  | |  |  | |  | |  | |  |  | |  |  |
| **171.1** |  |  | |  | |  |  | |  |  | | | |  |  | |  | |  | |  |  | |  | |  | |  |  | |  |  |
| **171.2** |  |  | |  | |  |  | |  |  | | | |  |  | |  | |  | |  |  | |  | |  | |  |  | |  |  |
| **172.1** |  |  | |  | |  |  | |  |  | | | |  |  | |  | |  | |  |  | |  | |  | |  |  | |  |  |
| **172.2** |  |  | |  | |  |  | |  |  | | | |  |  | |  | |  | |  |  | |  | |  | |  |  | |  |  |
| **173** |  |  | |  | |  |  | |  |  | | | |  |  | |  | |  | |  |  | |  | |  | |  |  | |  |  |
| **174** |  |  | |  | | 29,00 |  | |  |  | | | |  |  | |  | |  | |  |  | |  | |  | |  |  | |  |  |
| **177** |  |  | |  | |  |  | |  |  | | | |  |  | |  | |  | |  |  | |  | |  | |  |  | |  |  |
| **177.2** |  |  | |  | |  |  | |  |  | | | |  |  | |  | |  | |  |  | |  | |  | |  |  | |  |  |
| **177.3** |  |  | |  | |  |  | |  |  | | | |  |  | |  | |  | |  |  | |  | |  | |  |  | |  |  |
| **178** |  |  | |  | |  |  | |  |  | | | |  |  | |  | |  | |  |  | |  | |  | |  |  | |  |  |
| **179** |  |  | |  | |  |  | |  |  | | | |  |  | |  | | 60,92 | | 39,92 |  | |  | | 46,83 | |  |  | |  |  |
| **180** |  |  | |  | |  |  | |  |  | | | |  |  | |  | |  | |  |  | |  | |  | |  |  | |  |  |
| **181** |  |  | |  | |  |  | |  |  | | | |  |  | |  | |  | |  |  | |  | |  | |  |  | |  |  |
| **182** |  |  | |  | |  |  | |  |  | | | |  |  | |  | |  | |  |  | |  | |  | |  |  | |  |  |
| **183.1** |  |  | |  | |  |  | |  |  | | | |  |  | |  | |  | |  |  | |  | |  | |  |  | |  |  |
| **183.2** |  |  | |  | |  |  | |  |  | | | |  |  | |  | |  | |  |  | |  | |  | |  |  | |  |  |
| **184.1** |  |  | |  | | 2,00 |  | |  |  | | | |  | 3,10 | |  | | 55,00 | |  |  | |  | |  | |  |  | |  |  |
| **184.2** |  |  | |  | | 4,00 |  | |  |  | | | |  | 2,70 | |  | | 52,00 | |  |  | |  | |  | |  |  | |  |  |
| **184.3** |  |  | |  | | 0,00 |  | |  |  | | | |  | 3,00 | |  | | 57,00 | |  |  | |  | |  | |  |  | |  |  |
| **185** |  |  | |  | |  |  | |  |  | | | |  |  | |  | |  | |  |  | |  | |  | |  |  | |  |  |
| **186.1** |  |  | |  | | 6,00 |  | |  |  | | | |  | 3,00 | |  | | 56,30 | |  |  | |  | |  | |  |  | |  |  |
| **186.2** |  |  | |  | | 2,00 |  | |  |  | | | |  | 3,20 | |  | | 58,20 | |  |  | |  | |  | |  |  | |  |  |
| **187 .1** |  |  | |  | |  |  | |  |  | | | |  |  | |  | |  | |  |  | |  | |  | |  |  | |  |  |
| **187.2** |  |  | |  | |  |  | |  |  | | | |  |  | |  | |  | |  |  | |  | |  | |  |  | |  |  |
| **188** |  |  | | 100,00 | | 1,00 | 0,00 | |  |  | | | |  | 3,00 | |  | |  | | 41,50 |  | |  | | 42,70 | |  | 10,00 | |  |  |
| **189.1** |  |  | |  | |  |  | |  |  | | | |  |  | |  | |  | |  |  | |  | |  | |  |  | |  |  |
| **189 .2** |  |  | |  | |  |  | |  |  | | | |  |  | |  | |  | |  |  | |  | |  | |  |  | |  |  |
| **190.1** |  |  | |  | |  |  | |  |  | | | |  |  | |  | |  | |  |  | |  | |  | |  |  | |  |  |
| **190 .2** |  |  | |  | |  |  | |  |  | | | |  |  | |  | |  | |  |  | |  | |  | |  |  | |  |  |
| **191.1** |  |  | |  | |  |  | |  |  | | | |  |  | |  | |  | |  |  | |  | |  | |  |  | |  |  |
| **191.2** |  |  | |  | |  |  | |  |  | | | |  |  | |  | |  | |  |  | |  | |  | |  |  | |  |  |
| **191.3** |  |  | |  | |  |  | |  |  | | | |  |  | |  | |  | |  |  | |  | |  | |  |  | |  |  |
| **192.1** |  |  | |  | |  |  | |  |  | | | |  |  | |  | |  | |  |  | |  | |  | |  |  | |  |  |
| **192.2** |  |  | |  | |  |  | |  |  | | | |  |  | |  | |  | |  |  | |  | |  | |  |  | |  |  |
| **193.1** |  |  | |  | |  |  | |  |  | | | |  |  | |  | |  | |  |  | |  | |  | |  |  | |  |  |
| **193.2** |  |  | |  | |  |  | |  |  | | | |  |  | |  | |  | |  |  | |  | |  | |  |  | |  |  |
| **194.1** |  |  | |  | |  |  | |  |  | | | |  |  | |  | |  | |  |  | |  | |  | |  |  | |  |  |
| **194.2** |  |  | |  | |  |  | |  |  | | | |  |  | |  | |  | |  |  | |  | |  | |  |  | |  |  |
| **195.1** |  |  | |  | |  |  | |  |  | | | |  |  | |  | |  | |  |  | |  | |  | |  |  | |  |  |
| **195.2** |  |  | |  | |  |  | |  |  | | | |  |  | |  | |  | |  |  | |  | |  | |  |  | |  |  |
| **196** |  |  | |  | |  |  | |  |  | | | |  |  | |  | |  | |  |  | |  | |  | |  |  | |  |  |
| **197.1** | 46,90 |  | |  | |  |  | |  |  | | | |  |  | |  | |  | |  |  | |  | |  | |  |  | |  |  |
| **197.2** | 49,60 |  | |  | |  |  | |  |  | | | |  |  | |  | |  | |  |  | |  | |  | |  |  | |  |  |
| **198.1** |  |  | |  | |  |  | |  |  | | | |  |  | |  | |  | |  |  | |  | |  | |  |  | |  |  |
| **198.2** |  |  | |  | |  |  | |  |  | | | |  |  | |  | |  | |  |  | |  | |  | |  |  | |  |  |
| **199.1** |  |  | |  | |  |  | |  |  | | | |  |  | |  | |  | |  |  | |  | |  | |  |  | |  |  |
| **199.2** |  |  | |  | |  |  | |  |  | | | |  |  | |  | |  | |  |  | |  | |  | |  |  | |  |  |
| **199.3** |  |  | |  | |  |  | |  |  | | | |  |  | |  | |  | |  |  | |  | |  | |  |  | |  |  |
| **199.4** |  |  | |  | |  |  | |  |  | | | |  |  | |  | |  | |  |  | |  | |  | |  |  | |  |  |
| **199.5** |  |  | |  | |  |  | |  |  | | | |  |  | |  | |  | |  |  | |  | |  | |  |  | |  |  |
| **200.1** |  |  | |  | |  |  | |  |  | | | |  |  | |  | |  | |  |  | |  | |  | |  |  | |  |  |
| **200.2** |  |  | |  | |  |  | |  |  | | | |  |  | |  | |  | |  |  | |  | |  | |  |  | |  |  |
| **201** |  |  | |  | |  |  | |  |  | | | |  |  | |  | |  | |  |  | |  | |  | |  |  | |  |  |
| **202** |  |  | |  | |  |  | |  |  | | | |  |  | |  | | 53,88 | |  |  | |  | | 55,72 | |  |  | |  |  |
| **203** |  |  | |  | |  |  | |  |  | | | |  |  | |  | |  | |  |  | |  | |  | |  |  | |  |  |
| **204.1** |  |  | |  | |  |  | |  |  | | | |  | 2,40 | |  | | 49,00 | | 41,00 |  | |  | | 50,00 | |  |  | |  |  |
| **204.2** |  |  | |  | |  |  | |  |  | | | |  | 2,70 | |  | | 58,70 | | 40,00 |  | |  | | 50,00 | |  |  | |  |  |
| **205 .1** |  |  | |  | |  |  | |  |  | | | |  |  | |  | |  | |  |  | |  | |  | |  |  | |  |  |
| **205.2** |  |  | |  | |  |  | |  |  | | | |  |  | |  | |  | |  |  | |  | |  | |  |  | |  |  |
| **206** |  | 33,00 | | 67,00 | | 5,00 |  | |  |  | | | |  |  | |  | | 53,40 | |  |  | |  | |  | |  |  | |  |  |
| **207** |  |  | |  | |  |  | |  |  | | | |  |  | |  | |  | |  |  | |  | |  | |  |  | |  |  |
| **208** |  |  | |  | |  |  | |  |  | | | |  |  | |  | |  | |  |  | |  | |  | |  |  | |  |  |
| **209.1** |  |  | |  | |  |  | |  |  | | | |  |  | |  | |  | |  |  | |  | |  | |  |  | |  |  |
| **209.2** |  |  | |  | |  |  | |  |  | | | |  |  | |  | |  | |  |  | |  | |  | |  |  | |  |  |
| **209.3** |  |  | |  | |  |  | |  |  | | | |  |  | |  | |  | |  |  | |  | |  | |  |  | |  |  |
| **209.4** |  |  | |  | |  |  | |  |  | | | |  |  | |  | |  | |  |  | |  | |  | |  |  | |  |  |
| **209.5** |  |  | |  | |  |  | |  |  | | | |  |  | |  | |  | |  |  | |  | |  | |  |  | |  |  |
| **210** |  |  | |  | |  |  | |  |  | | | |  |  | |  | | 58,80 | | 45,60 |  | |  | | 53,40 | |  |  | |  |  |
| **211.1** |  |  | |  | |  |  | |  |  | | | |  |  | |  | |  | |  |  | |  | |  | |  |  | |  |  |
| **211.2** |  |  | |  | |  |  | |  |  | | | |  |  | |  | |  | |  |  | |  | |  | |  |  | |  |  |
| **212** |  |  | |  | |  |  | |  |  | | | |  |  | |  | | 58,50 | |  |  | |  | |  | |  |  | |  |  |
| **213** |  |  | |  | |  |  | |  |  | | | |  |  | |  | |  | |  |  | |  | |  | |  |  | |  |  |
| **214** |  |  | |  | |  |  | |  |  | | | |  |  | |  | |  | |  |  | |  | |  | |  |  | |  |  |
| **215** |  |  | |  | | 4,00 |  | |  |  | | | |  |  | |  | |  | |  |  | |  | |  | |  |  | |  |  |
| **216** |  |  | |  | |  |  | |  |  | | | |  |  | |  | |  | |  |  | |  | |  | |  |  | |  |  |
| **217** |  |  | |  | |  |  | |  |  | | | |  |  | |  | |  | |  |  | |  | |  | |  |  | |  |  |
| **218** |  |  | |  | |  |  | |  |  | | | |  |  | |  | |  | |  |  | |  | |  | |  |  | |  |  |
| **219.1** |  |  | |  | |  |  | |  |  | | | |  |  | |  | |  | |  |  | |  | |  | |  |  | |  |  |
| **219 .2** |  |  | |  | |  |  | |  |  | | | |  |  | |  | |  | |  |  | |  | |  | |  |  | |  |  |
| **220.1** |  |  | | 32,00 | | 4,00 | 0,00 | |  |  | | | |  |  | |  | | 55,00 | |  |  | |  | |  | |  |  | |  |  |
| **220.2** |  |  | | 22,00 | | 1,00 | 0,00 | |  |  | | | |  |  | |  | | 52,00 | |  |  | |  | |  | |  |  | |  |  |
| **220.3** |  |  | | 10,00 | | 1,00 | 0,00 | |  |  | | | |  |  | |  | | 55,00 | |  |  | |  | |  | |  |  | |  |  |
| **220.4** |  |  | | 0,00 | | 2,00 | 0,00 | |  |  | | | |  |  | |  | | 58,00 | |  |  | |  | |  | |  |  | |  |  |
| **220.5** |  |  | | 45,00 | | 2,00 | 0,00 | |  |  | | | |  |  | |  | | 55,00 | |  |  | |  | |  | |  |  | |  |  |
| **221** |  |  | |  | |  |  | |  |  | | | |  |  | |  | |  | |  |  | |  | |  | |  |  | |  |  |
| **222.1** |  |  | |  | |  |  | |  |  | | | |  |  | |  | |  | |  |  | |  | |  | |  |  | |  |  |
| **222.2** |  |  | |  | |  |  | |  |  | | | |  |  | |  | |  | |  |  | |  | |  | |  |  | |  |  |
| **223** |  |  | |  | |  |  | |  |  | | | |  | 4,20 | | 8,50 | |  | |  |  | | 1,83 | |  | |  |  | |  |  |
| **224** |  |  | |  | |  |  | |  |  | | | |  |  | |  | | 50,63 | |  |  | |  | |  | |  |  | |  |  |
| **225.1** |  |  | |  | |  |  | |  |  | | | |  |  | |  | | 55,56 | |  |  | |  | |  | |  |  | |  |  |
| **225.2** |  |  | |  | |  |  | |  |  | | | |  |  | |  | | 54,55 | |  |  | |  | |  | |  |  | |  |  |
| **Ʃ** |  |  | | 2750,00 | | 540,00 | 275,00 | | 0,00 | 117,00 | | | | 0,00 |  | |  | |  | |  |  | |  | |  | |  |  | |  |  |
| **mean** | 348,21 |  | |  | |  |  | |  |  | | | |  | 3,13 | | 7,28 | | 55,99 | | 39,29 | 1,22 | | 1,22 | | 48,13 | |  | 9,74 | |  |  |
| **SD** | 624,28 |  | |  | |  |  | |  |  | | | |  | 0,70 | | 0,91 | | 3,26 | | 3,53 | 0,00 | | 0,62 | | 6,41 | |  | 0,37 | |  |  |
